# Supplementary material for: Enhanced IFNα Signaling Promotes Ligand-Independent Activation of ERα to Promote Aromatase Inhibitor Resistance in Breast Cancer
Source: Cancers (Basel). 2021 Oct 13;13(20):5130. doi: 10.3390/cancers13205130 (PMC8534010; doi:10.3390/cancers13205130)
Supplement: Supplementary file 1 [file cancers-13-05130-s001.zip › cancers-1384109-supplementary/cancers-1384109-0-supplementary.pdf]

Figure S1

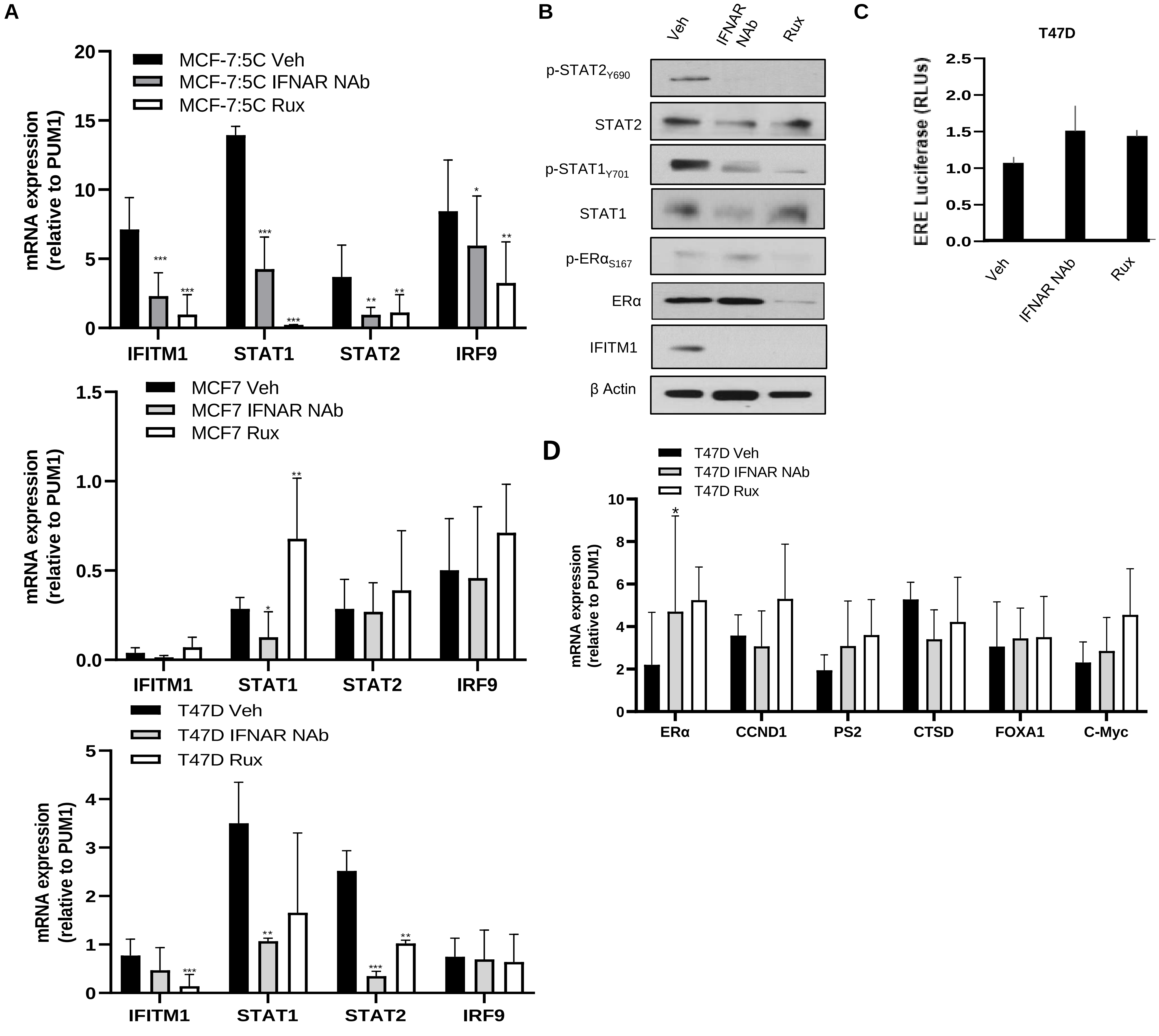

Figure S1. IFNα signaling inhibition on ERα in T47D cells. (**a**) T47D, MCF-7, and MCF-7:5C cells were treated for 48 hours with IFNAR NAb or Rux (as indicated) and analyzed by RT-PCR for IFITM1, STAT1, STAT2, and IRF9 expression. (**b**) T47D, cells were treated for 48 hours with IFNAR NAb or Rux (as indicated) and immunoblotted for p-ERα, ERα, p-STAT2, STAT2, p-STAT1, STAT1, and IFITM1 expression. (**c**) Luciferase assay was used to determine the activity of the ERE promoter in T47D cells treated with IFNAR NAb or Rux. (**d**) T47D cells were treated for 48 hours with IFNAR NAb or Rux (as indicated) and analyzed by RT-PCR for ER-regulated gene expression. *p<0.05, **p< 0.01 and ***p< 0.001

Figure S2

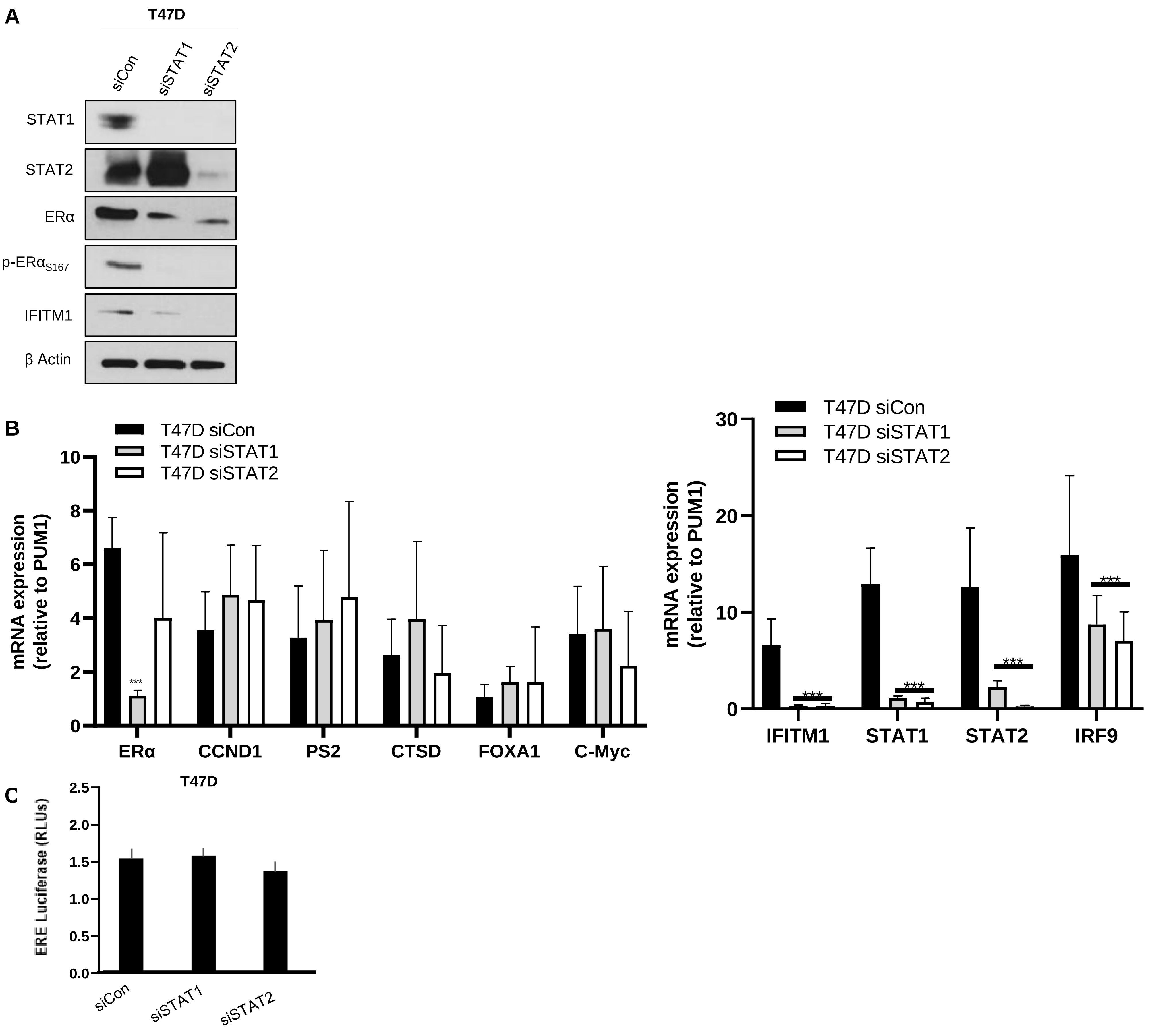

Figure S2. STAT1 and STAT2 signaling inhibition on ERα in T47D cells. (a) T47D cells were transiently transfected for 48 hours with siRNA against STAT1 or STAT2 (as indicated) and immunoblotted for p-ERα, ERα, STAT2, STAT1, and IFITM1 expression. (**b**) T47D cells were transiently transfected for 48 hours with siRNA against STAT1 or STAT2 (as indicated) and were analyzed by RT-PCR. **(c)** T47D cells were transiently transfected for the ERE reporter construct and siRNA against STAT1 or STAT2 treated for 48 hours. Then Luciferase and Renilla activities were measured 24 h later using the Dual-Luciferase® reporter assay kit. ***$p < 0.001$

Figure S3

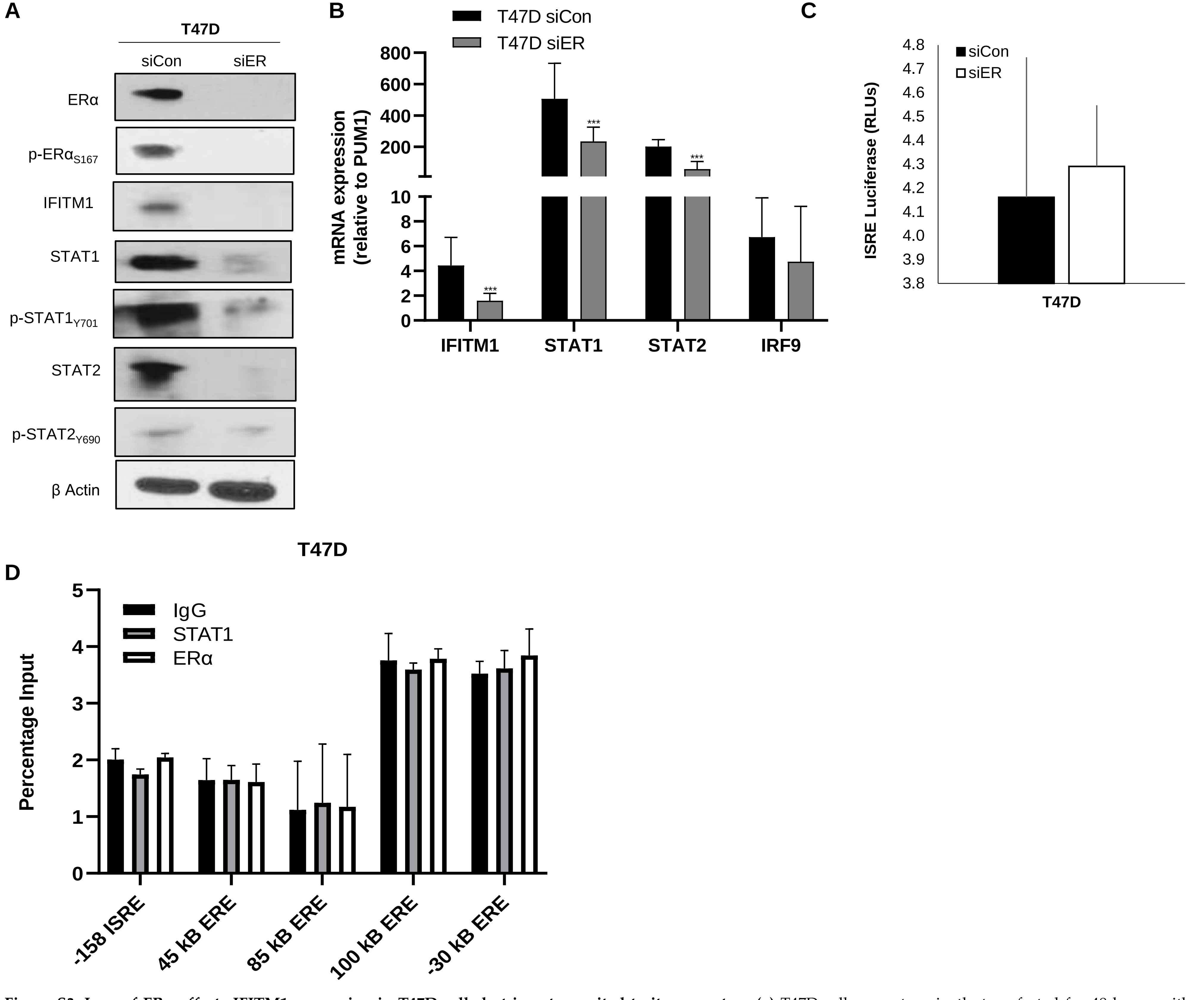

Figure S3. Loss of ERα effects IFITM1 expression in T47D cells but is not recruited to its promoter. **(a)** T47D cells were transiently transfected for 48 hours with siRNA against ERα and immunoblotted for p-ERα, ERα, p-STAT2, STAT2, p-STAT1, STAT1, and IFITM1 expression. (**b**) T47D cells were transiently transfected for 48 hours with siRNA against ERα and IFITM1, STAT1, STAT2 and IRF9 levels were analyzed by RT-PCR. **(c)** T47D cells were transiently transfected for the ISRE reporter construct and siRNA against ERα for 48 hours. Then Luciferase and Renilla activities were measured 24 h later using the Dual-Luciferase® reporter assay kit. **(d)** Fixed T47D cell lysates were subjected to chromatin immunoprecipitation (ChIP) with antibodies against ERα, STAT1 or species-specific IgG control. qPCR was performed on the isolated DNA using primers designed to amplify the ERE and ISRE regulatory regions. DNA gels were run to analyze the amount of DNA that was amplified by qPCR. Recruitment of the indicated proteins to the ERE and ISRE site was compared to input DNA and displayed as mean ± SD of technical triplicates in two independent experiments. ***$p < 0.001$

Table S1

Primer	Left	Right
ISRE	TTGGTCCCTGGCTAATTCAC	TTGGGGAAGGAAGTGTTGAG
+ 30 kB IFITM1 promoter	ACGGAAGTATGACCTAGGAAAGAAG	CCAGTACCTAACCTGGTAATGAAGC
-45 kB IFITM1 promoter	CAGCTTCCTGGACACTCTGC	GTCGGTTCGGGAATGTTTTC
-85 kB IFITM1 promoter	CTGTCTGTCCACTCACTGTGG	CTGTCTGTCCACTCACTGTGG
-100 kB IFITM1 promoter	ATTCAGACCCTAGACTTTGCTGAC	ACATGTCACGAGCAGAGTGC
SP1	GCCTCCAGACCATTAACCTCAGT	GCTCCATGATCACCTGGGGCA
SRC1	CATGGTCAGGCAAAAACCTT	CTTGCCGATTTTGGTGTAT
SRC3	TTCAGGAAAGGTTGTCAATATAGATACA	AATACACCTTCGGATTATATCTTCAAA
CITED1	AGGATGCCAACCAAGAGATG	GTTTAGTGGGAGGGGTGGTT
GATA3	ACCACAACCACACTCTGGAGGA	TCGGTTTCTGGTCTGGATGCCT
CBP	TGCCAAGTTGCCCATTGTG	TTGTTGGTTTCGCTTGTCACT
P300	TTCAAACGCCGAGTCTTCTT	GTTGAGCTGCTGTTGGCATA
CCND1	GCGGAGGAGAACAAACAGAT	GAGGGCGGATTGGAAATGA
PS2	ATGGCCACCATGGAGAACAAGG	CTAAAATTCACACTCCTCTTCTGG
CTSD	GCCAGGACCCTGTGTCG	GCACGTTGTTGACGGAGATG
FOXA1	ACAGGGTTGGATGGTTGTGT	TGTTGCTGACAGGGACAGAG
C-Myc	TACCCTCTCAACGACAGCAG	TCTTGACATTCTCCTCGGTG
ERα	AAGAGGGTGCCAGGCTTTGT	CAGGATCTCTAGCCAGGCACAT
IFITM1	GGATTTCGGCTTGTCCCGAG	CCATGTGGAAGGGAGGGCTC
STAT1	CCGCCATGTTTACAGCAGAT	GTCCCCTAGGACCTCCTCAT
STAT2	GCAGCACCATTTGCGGAA	ACAGGTGTTTCGAGAACTGGC
IRF9	TTCTGTCCCTGGTGTAGAGCCT	TTTCAGGACACGATTATCACGG
IRF3	AAGACCCTCACGACCCACATAA	GGCCAACACCATGTTACCCAGT
IFNα	CTTGAAGGACAGACATGACTTTGGA	GGATGGTTTCAGCCTTTTGGA
IFNβ	GTCTCCTCCAAATTGCTCTC	ACAGGAGCTTCTGACACTGA
IFIT1	TCTCAGAGGAGCCTGGCTAA	CCAGACTATCCTTGACCTGATGA
OAS1	TGAGGTCCAGGCTCCACGCT	GCAGGTCGGTGCACTCCTCG
PLSCR1	CATTCACCGGGCTCTCTAC	GGCAGCTGGGCAATCTTGCA
PUM1	TCACCGAGGCCCCTCTGAACCCTA	GGCAGTAATCTCCTTCTGCATCCT
