# Supplementary material for: Enhanced IFNα Signaling Promotes Ligand-Independent Activation of ERα to Promote Aromatase Inhibitor Resistance in Breast Cancer
Source: Cancers (Basel). 2021 Oct 13;13(20):5130. doi: 10.3390/cancers13205130 (PMC8534010; doi:10.3390/cancers13205130)
Supplement: Supplementary file 1 [file cancers-13-05130-s001.zip › cancers-1384109-supplementary/cancers-1384109-western blot/ER paper WBs/WB0002.pdf]

Thanks COVID-19!

5/21/20 Need to take out two more vials of 5C cells.
Ran cell line western

marker | T47D p112 | T47D p113 | MCF7 p209 | MCF7 p210 | 5C p128 | 5C p129 | 5C Sicon | mark[illegible]

5/22/20 Imaged WB - everything looks good with cells.

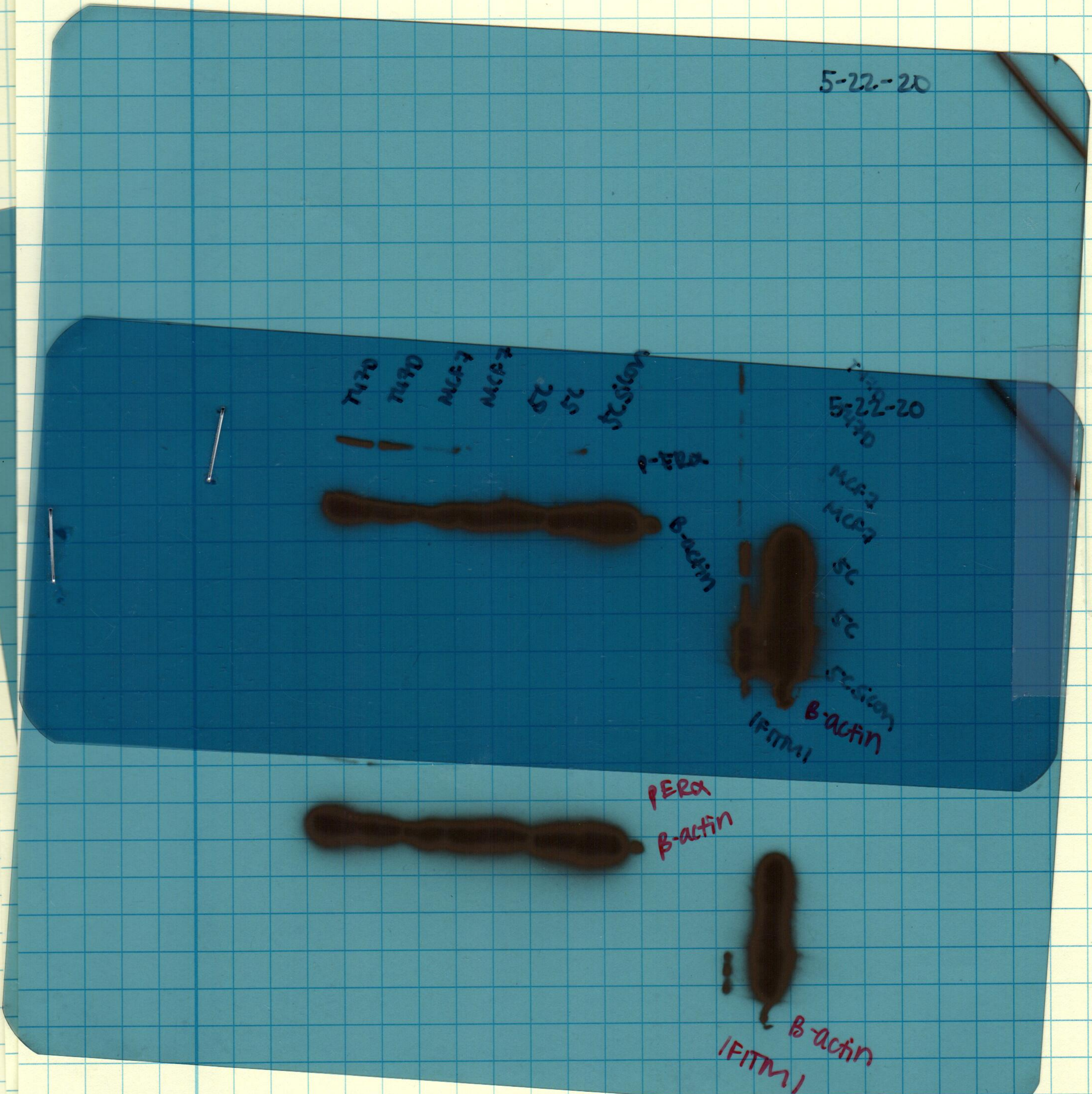

Changed media on cells.
Meeting with Dr. Wambi.
