# Supplementary material for: Enhanced IFNα Signaling Promotes Ligand-Independent Activation of ERα to Promote Aromatase Inhibitor Resistance in Breast Cancer
Source: Cancers (Basel). 2021 Oct 13;13(20):5130. doi: 10.3390/cancers13205130 (PMC8534010; doi:10.3390/cancers13205130)
Supplement: Supplementary file 1 [file cancers-13-05130-s001.zip › cancers-1384109-supplementary/cancers-1384109-western blot/ER paper WBs/WB0004.pdf]

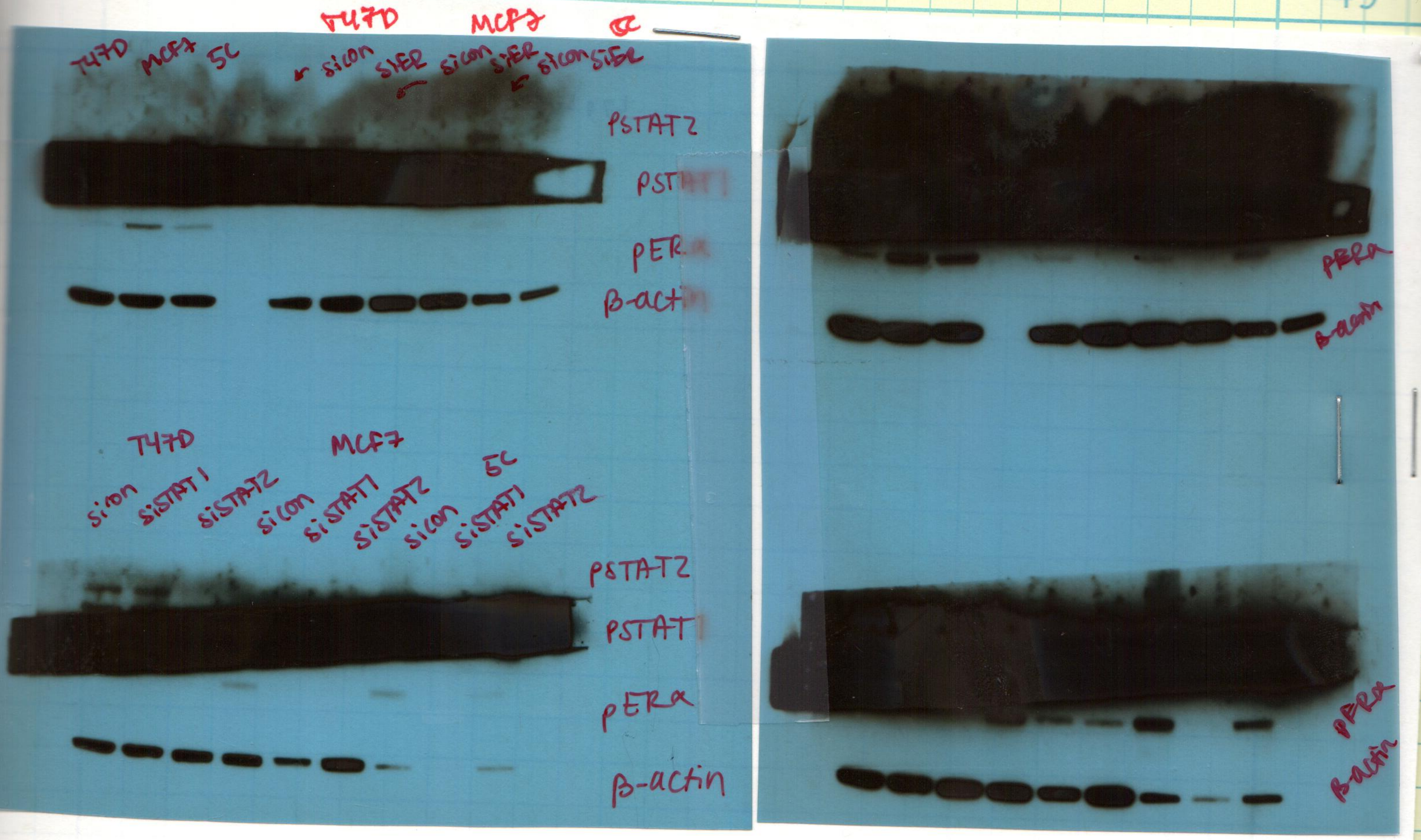

Imaged WBs ⮥

Replaced 1° Abs to reblot tomorrow.

Ask Shri for Fulbright rec letter.

Treated cells with E_2 (1nm, 2ul/2mL) and IFNα (20 u/mL, 400uL/2mL)

Changed media on cells.

2-2020

Compiled luciferase data from CSF media tests

Set up PCR template to run tomorrow

Plated 6-well yellow T47D and MCF7 plates

T47D y

con	E2	IFNα

MCF7 y

con	E2	IFNα

T47D y − $\frac{78}{4}$ x10 x 10,000 cells = 1,950,000 cells/mL

$\frac{1,300,000 \text{ cells}}{1,950,000 \text{ cells/mL}} \times \frac{1000 \text{ uL}}{\text{mL}}$ = 667 uL in 13 mL media

MCF7 y − $\frac{178}{4}$ x10 x 10,000 cells = 4,450,000 cells/mL

$\frac{1,300,000 \text{ cells}}{4,450,000 \text{ cells/mL}} \times \frac{1000 \text{ uL}}{\text{mL}}$ = 292 uL in 13 mL media
