# Supplementary material for: Enhanced IFNα Signaling Promotes Ligand-Independent Activation of ERα to Promote Aromatase Inhibitor Resistance in Breast Cancer
Source: Cancers (Basel). 2021 Oct 13;13(20):5130. doi: 10.3390/cancers13205130 (PMC8534010; doi:10.3390/cancers13205130)

$\frac{1,300,000}{4,400,000}$ = 295 uL cells in 13 mL media

T47D + MCF7 yellow

sicon ↓	siSTAT1	siSTAT2
	si ER	E2

con	ICI	E2+ ICI
Rux	Ita	IFNα

5C

sicon ↓	siIFITM1 ↓	con
		E2

8/21/2020 Transfection and drug treatment for 6-wells
Re-imaged westerns
Changed media on cells
Ran western:

				T47D		MCF7		5C	
T47D	MCF7	5C	marker	sicon	siER	sicon	siER	sicon	siER

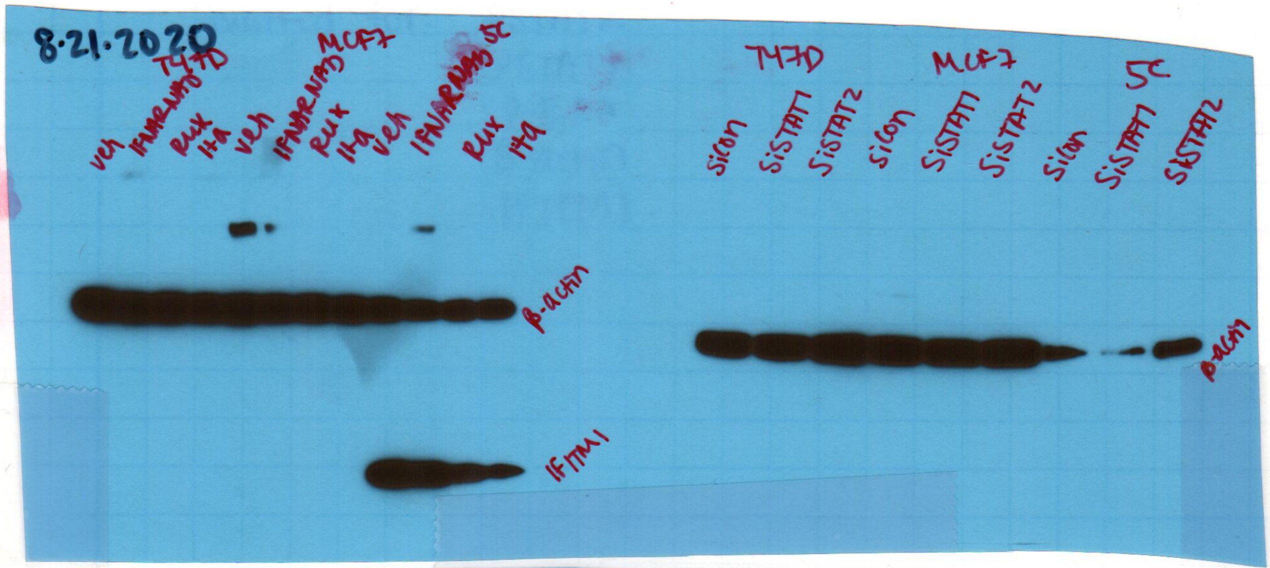

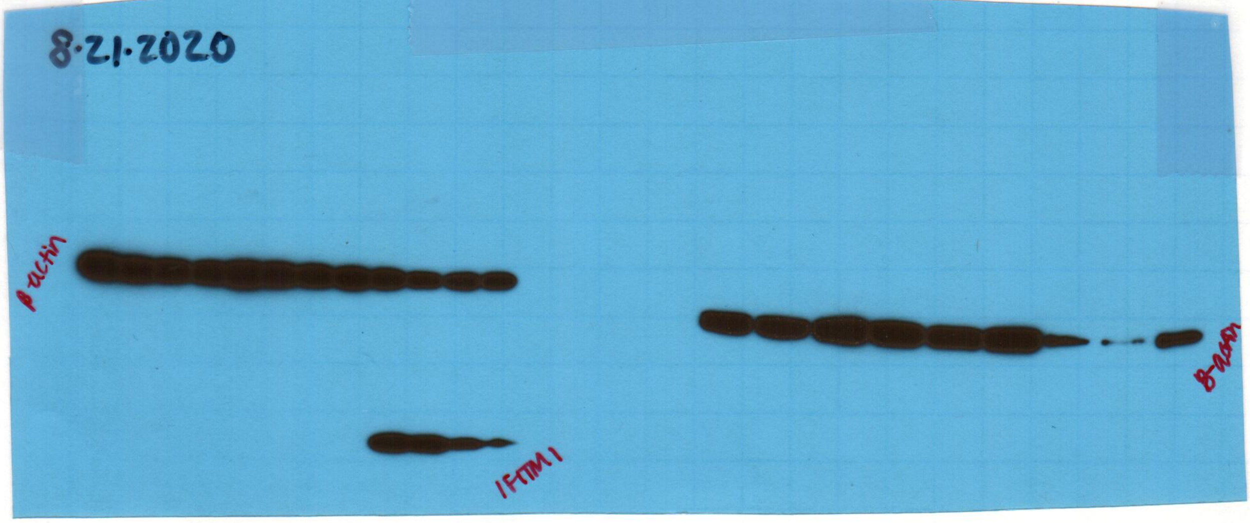

Supplement: Supplementary file 1 [file cancers-13-05130-s001.zip › cancers-1384109-supplementary/cancers-1384109-western blot/ER paper WBs/WB0007.pdf]
