# Supplementary material for: Enhanced IFNα Signaling Promotes Ligand-Independent Activation of ERα to Promote Aromatase Inhibitor Resistance in Breast Cancer
Source: Cancers (Basel). 2021 Oct 13;13(20):5130. doi: 10.3390/cancers13205130 (PMC8534010; doi:10.3390/cancers13205130)
Supplement: Supplementary file 1 [file cancers-13-05130-s001.zip › cancers-1384109-supplementary/cancers-1384109-western blot/ER paper WBs/WB0008.pdf]

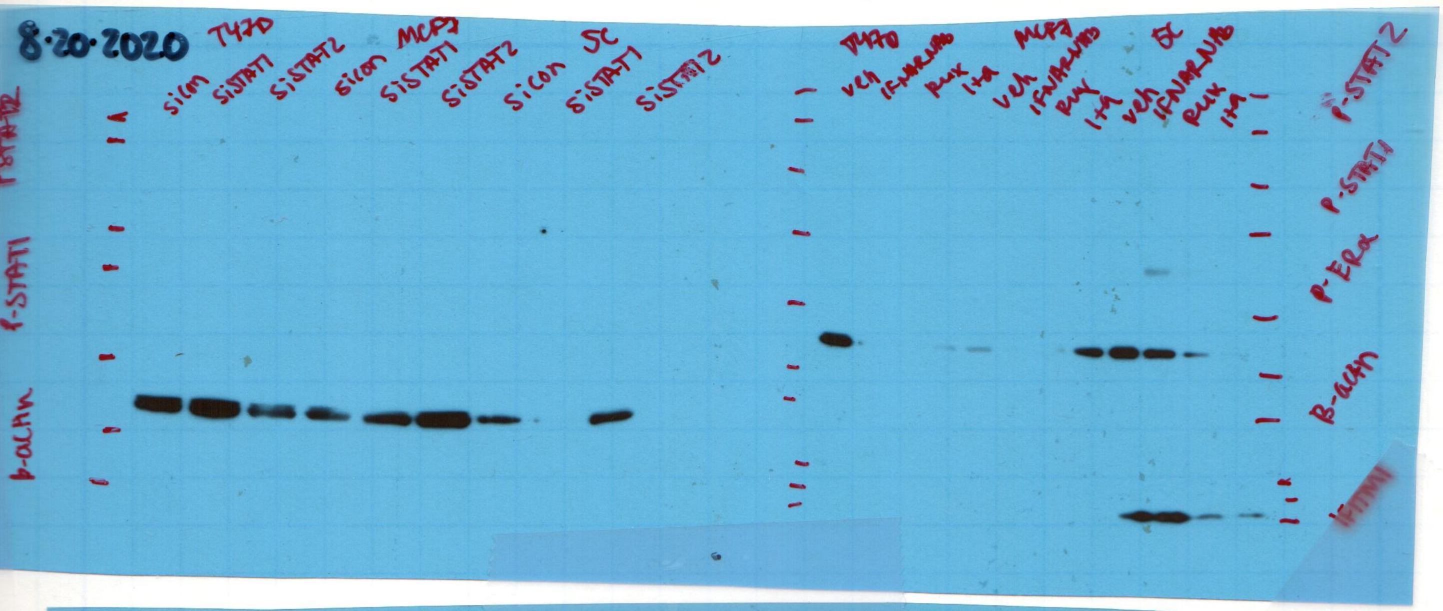

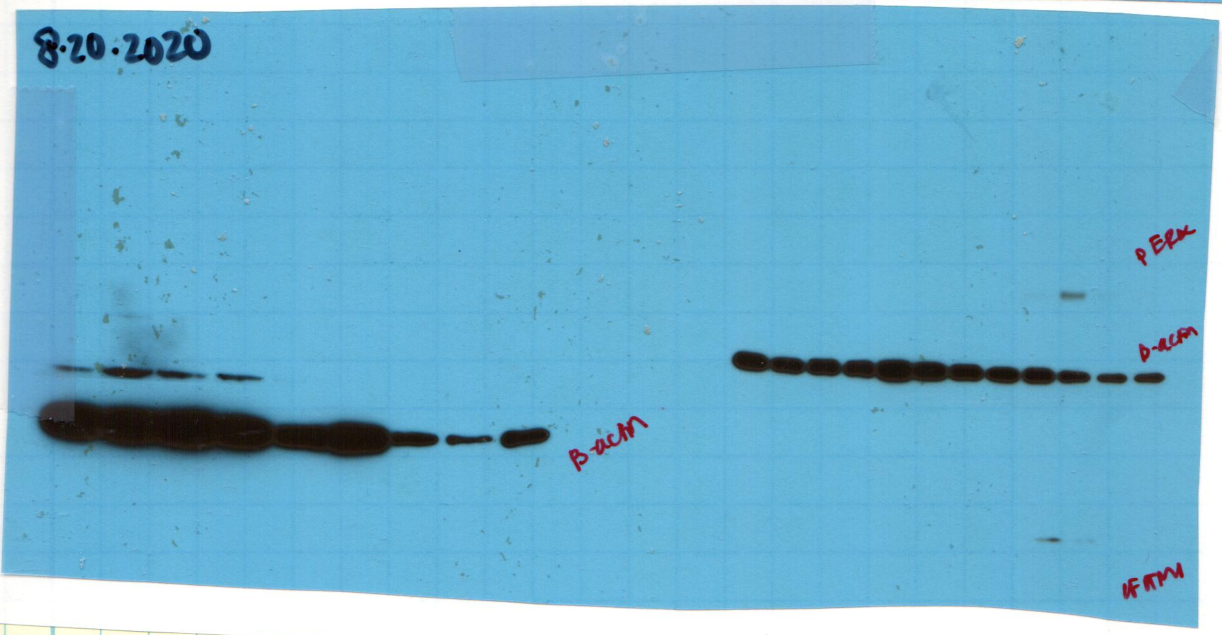

.2020 collected samples from 6-wells (p.54)
washed and added 2° Abs to westerns

.2020 imaged westerns ⟶
IFITM1 isn't showing up... what?

.2020 Ran qRT-PCR
24h 5C sicon
siER
siSTAT1
siSTAT2
veh
Rux
EZ
IFNα
ita
} need to analyze data.
