# Supplementary material for: Enhanced IFNα Signaling Promotes Ligand-Independent Activation of ERα to Promote Aromatase Inhibitor Resistance in Breast Cancer
Source: Cancers (Basel). 2021 Oct 13;13(20):5130. doi: 10.3390/cancers13205130 (PMC8534010; doi:10.3390/cancers13205130)
Supplement: Supplementary file 1 [file cancers-13-05130-s001.zip › cancers-1384109-supplementary/cancers-1384109-western blot/ER paper WBs/WB0010.pdf]

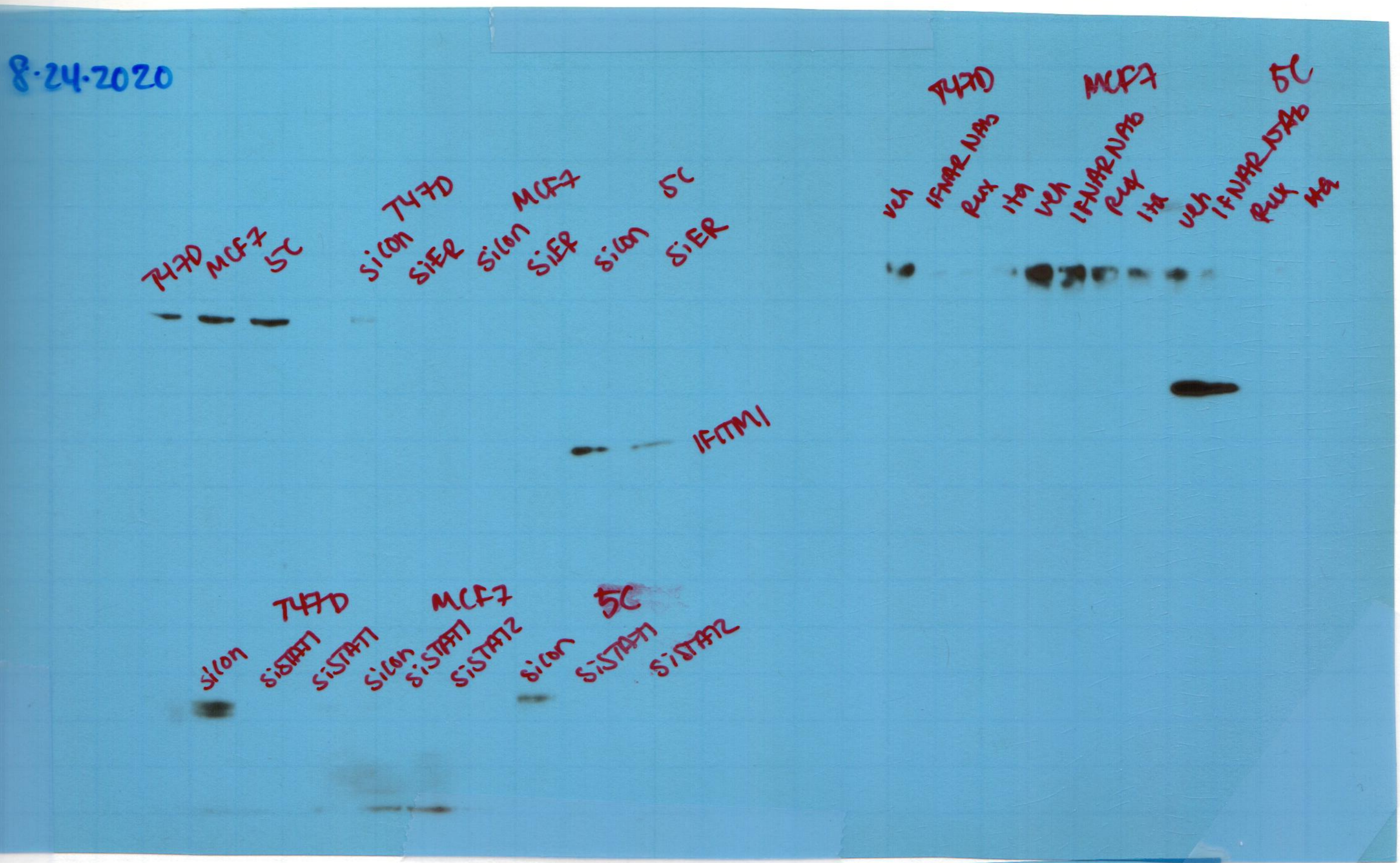
8·24·2020
T47D MCF7 5C
T47D MCF7 5C
sicon siER sicon siER sicon siER
IFITM1
T47D MCF7 5C
veh IFNAR NAb flux itg veh IFNAR NAb flux itg veh IFNAR NAb flux itg
T47D MCF7 5C
sicon siSTAT1 siSTAT1 sicon siSTAT1 siSTAT2 sicon siSTAT1 siSTAT2

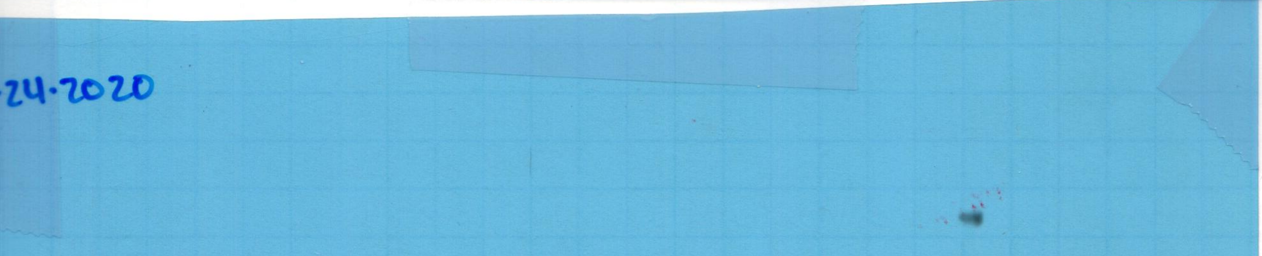
24·2020
