# Supplementary material for: Enhanced IFNα Signaling Promotes Ligand-Independent Activation of ERα to Promote Aromatase Inhibitor Resistance in Breast Cancer
Source: Cancers (Basel). 2021 Oct 13;13(20):5130. doi: 10.3390/cancers13205130 (PMC8534010; doi:10.3390/cancers13205130)
Supplement: Supplementary file 1 [file cancers-13-05130-s001.zip › cancers-1384109-supplementary/cancers-1384109-western blot/ER paper WBs/WB0011.pdf]

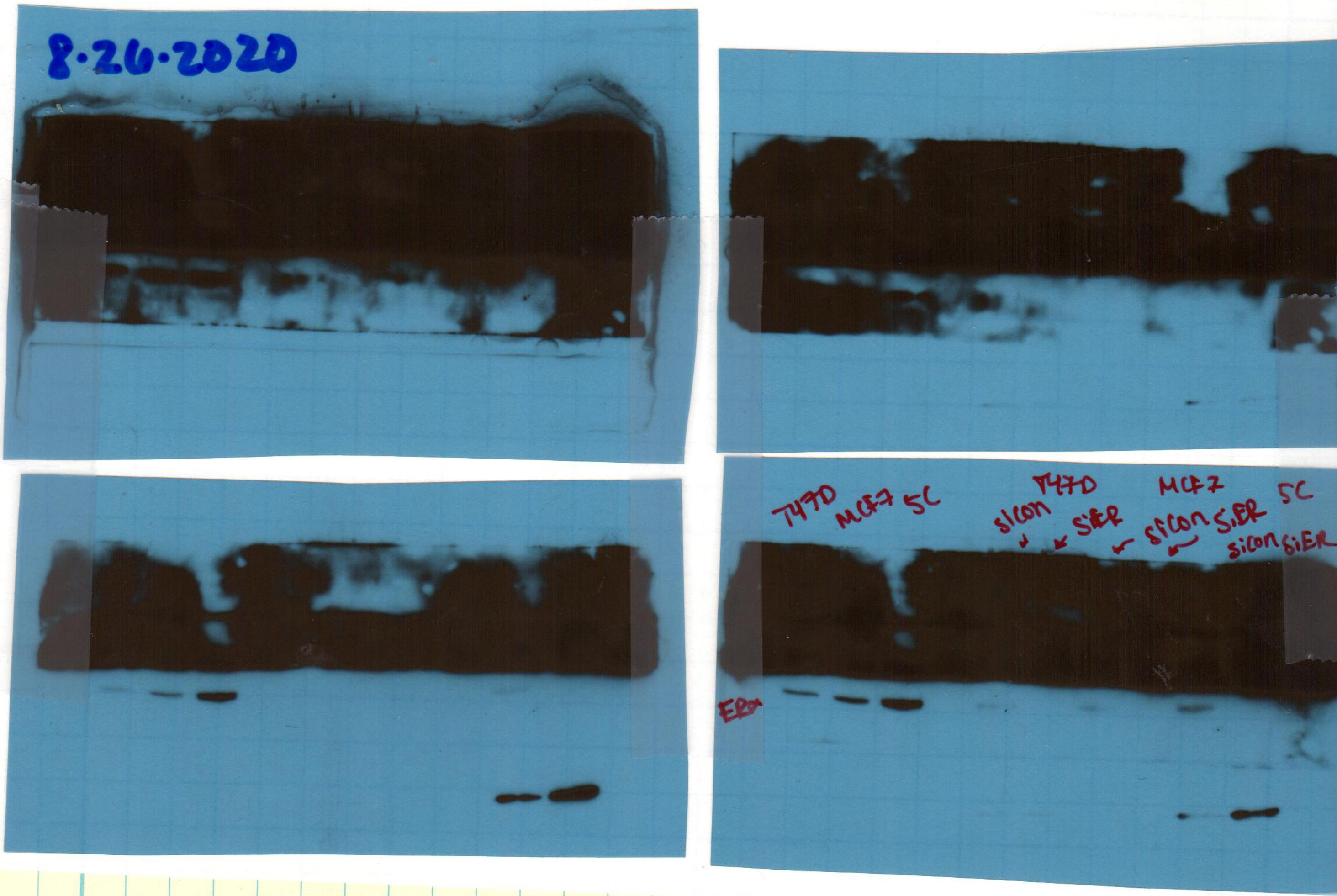

8·26·2020 Re-imaged westerns
5% BSA blacks out blot but allows P-ERα to show up.
for PSTATS

Re-run qRT-PCR

24h 5C sicon
siER
siSTAT1
veh
Rux
E2
Ita

8·27·2020 [illegible]

Analyzed qRT-PCR data

[illegible] Passaged cells

Protein assay and RNA isolation

T47D
MCF7
5C Fox Chase CSF
5C 2x CSF May 2020

sicon
siIFITM1

Read article for class
