# Supplementary material for: Enhanced IFNα Signaling Promotes Ligand-Independent Activation of ERα to Promote Aromatase Inhibitor Resistance in Breast Cancer
Source: Cancers (Basel). 2021 Oct 13;13(20):5130. doi: 10.3390/cancers13205130 (PMC8534010; doi:10.3390/cancers13205130)
Supplement: Supplementary file 1 [file cancers-13-05130-s001.zip › cancers-1384109-supplementary/cancers-1384109-western blot/ER paper WBs/Western Scans - Lab Notebook 3 IN BINDER/WB0014.pdf]

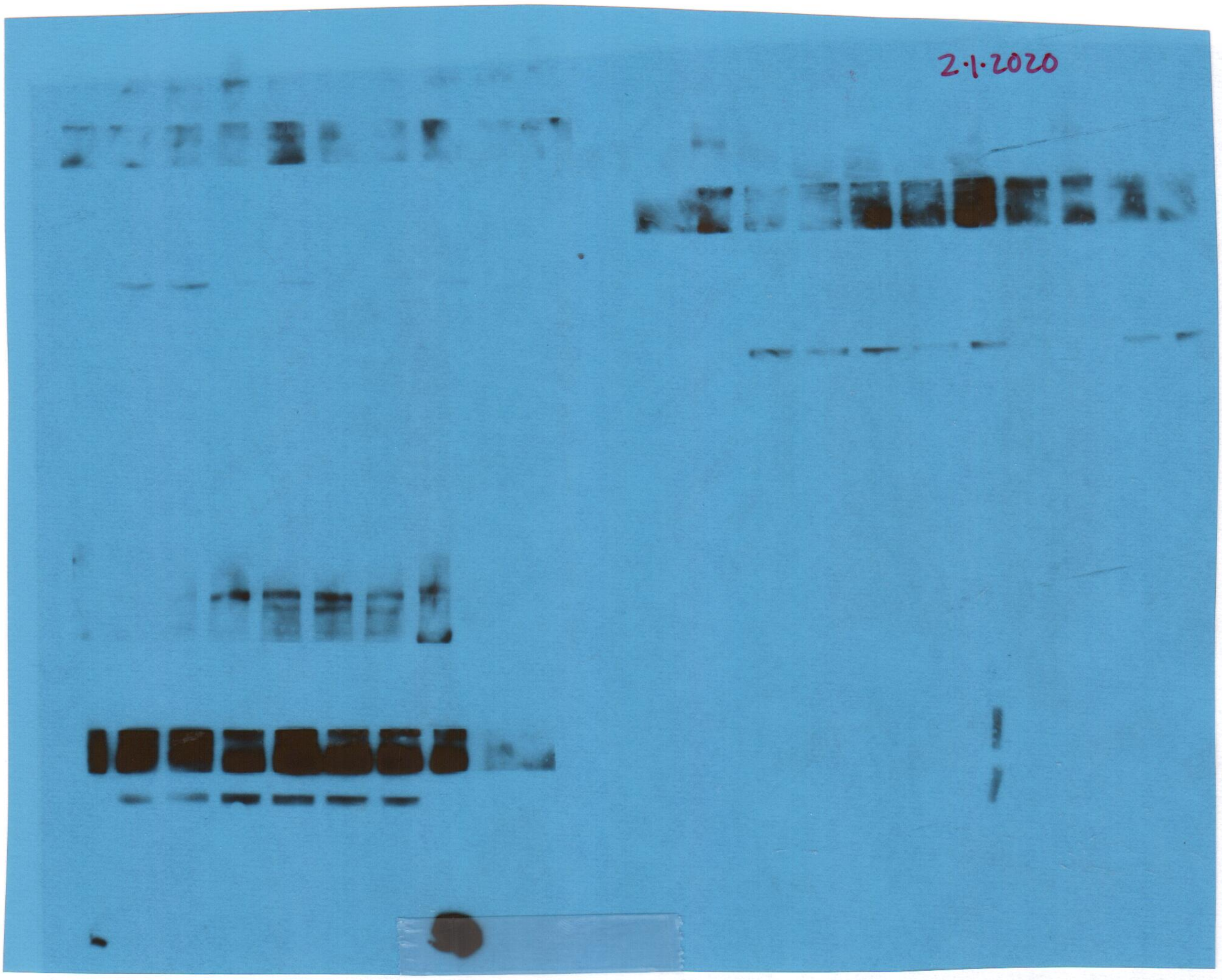
2.1.2020

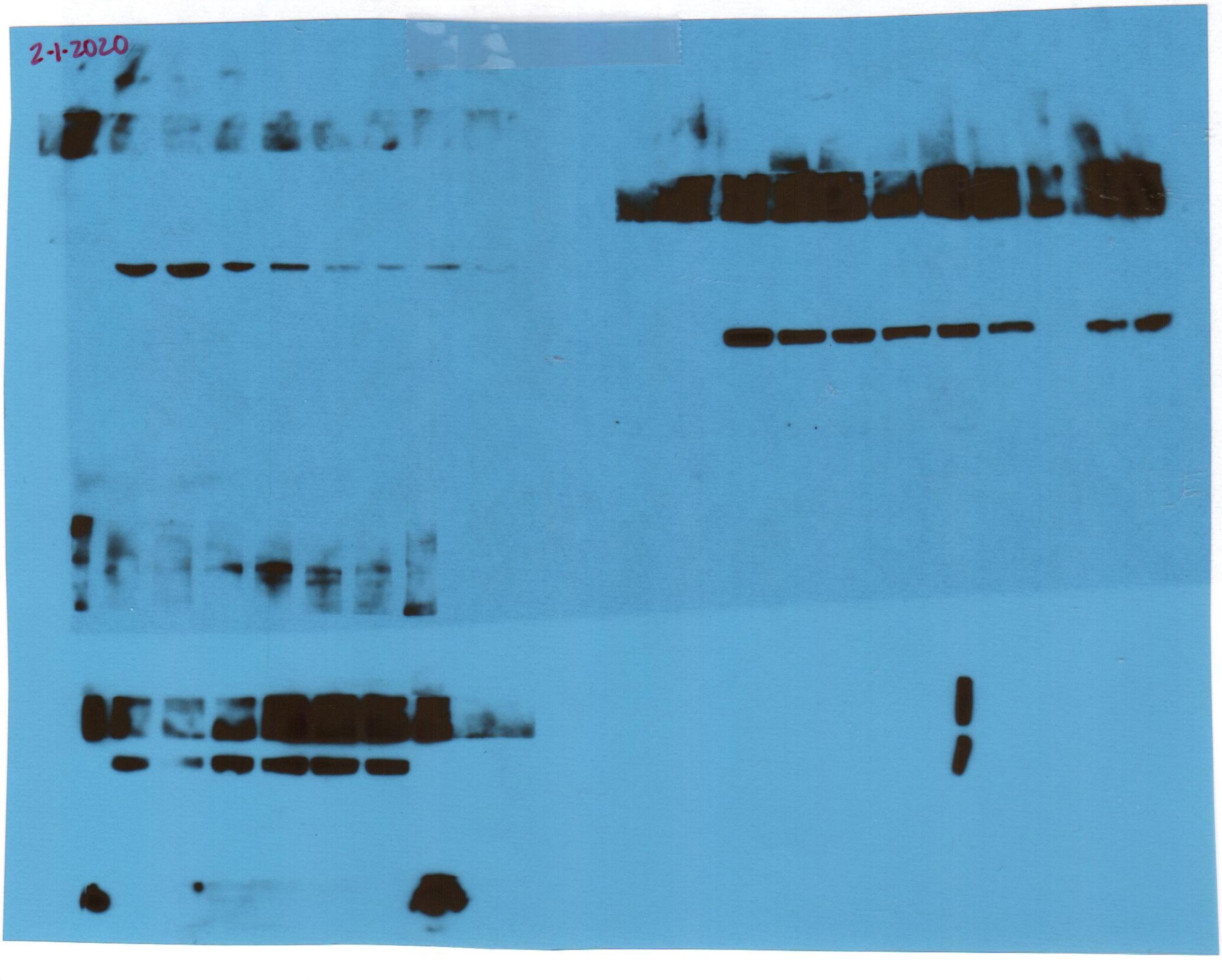
2.1.2020
