# Supplementary material for: Enhanced IFNα Signaling Promotes Ligand-Independent Activation of ERα to Promote Aromatase Inhibitor Resistance in Breast Cancer
Source: Cancers (Basel). 2021 Oct 13;13(20):5130. doi: 10.3390/cancers13205130 (PMC8534010; doi:10.3390/cancers13205130)
Supplement: Supplementary file 1 [file cancers-13-05130-s001.zip › cancers-1384109-supplementary/cancers-1384109-western blot/ER paper WBs/Western Scans - Lab Notebook 3 IN BINDER/WB0026.pdf]

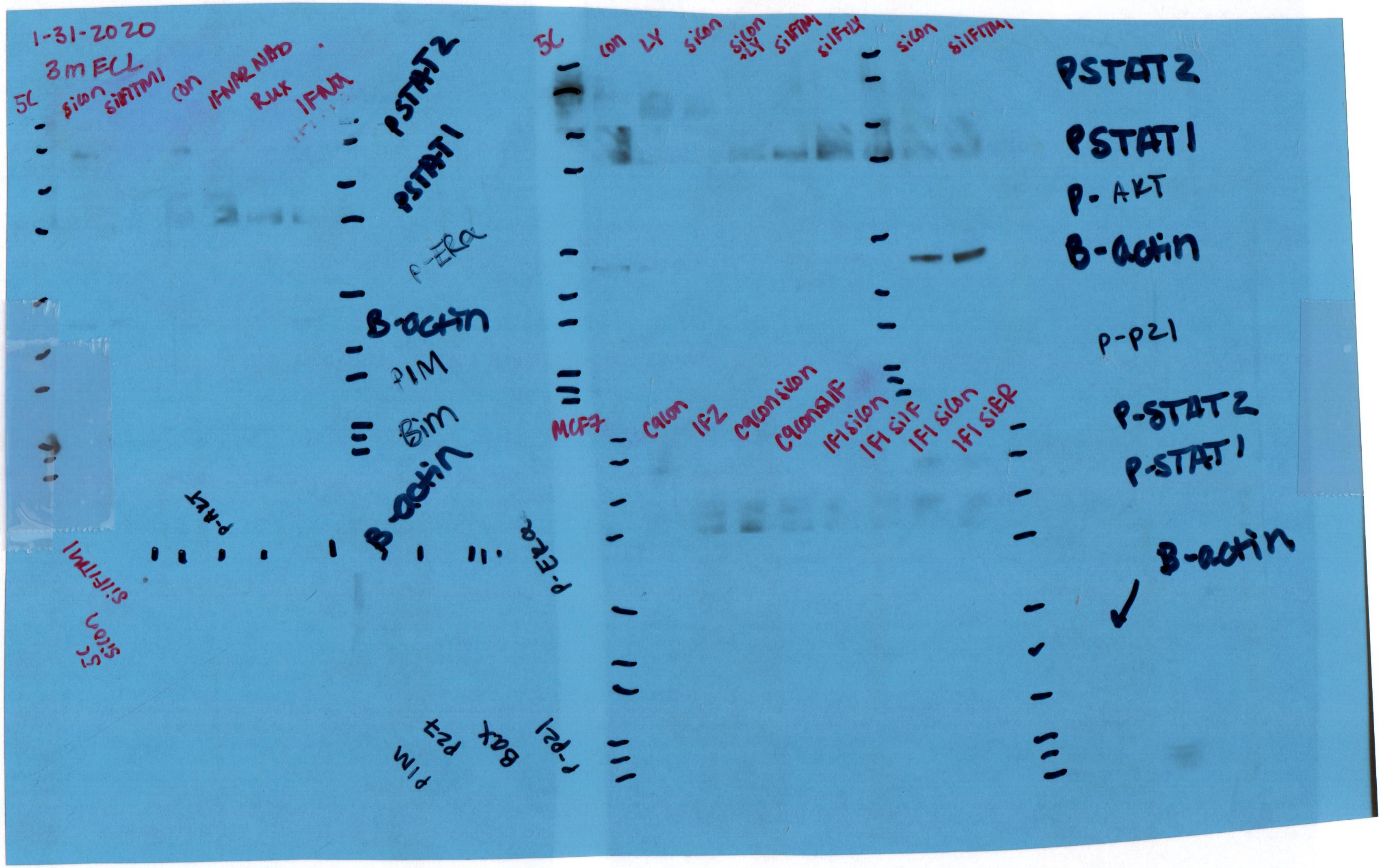
1-31-2020
8m ECL
5C
sicon
siIFITM1
con
IFNAR NAb
Rux
IFNAR
PSTAT2
PSTAT1
P-ERα
B-actin
PIM
Bim
B-actin
P-AKT
P-ERα
5C
sicon
siIFITM1
PIM
P27
Bax
P-P21
5C
con
LY
sicon
sicon LY
siIFITM
siIFLY
sicon
siIFITM1
MCF7
C9con
IF2
C9consicon
C9consiIF
IF1sicon
IF1siIF
IF1sicon
IF1siER
PSTAT2
PSTAT1
P-AKT
B-actin
P-P21
P-STAT2
P-STAT1
B-actin
