# Supplementary material for: Enhanced IFNα Signaling Promotes Ligand-Independent Activation of ERα to Promote Aromatase Inhibitor Resistance in Breast Cancer
Source: Cancers (Basel). 2021 Oct 13;13(20):5130. doi: 10.3390/cancers13205130 (PMC8534010; doi:10.3390/cancers13205130)

2/26/2021

| | 1 | 2 | 3 | 4 | 5 | 6 | 7 | 8 | 9 | 10 | 11 | 12 | 13 | 14 | 15 | 16 | 17 | 18 | 19 | 20 | 21 | 22 | 23 | 24 |
|---|---|---|---|---|---|---|---|---|---|---|---|---|---|---|---|---|---|---|---|---|---|---|---|---|
| | MCF7 siCon | | | MCF7 siSTAT1 | | | MCF7 siSTAT2 | | | 5C veh | | | 5C Rux | | | 5C IFNAR Nab | | | | | | | | |
| A | PUM1 | PUM1 | PUM1 | PUM1 | PUM1 | PUM1 | PUM1 | PUM1 | PUM1 | PUM1 | PUM1 | PUM1 | PUM1 | PUM1 | PUM1 | PUM1 | PUM1 | PUM1 | | | | | | |
| B | IFITM1 | IFITM1 | IFITM1 | IFITM1 | IFITM1 | IFITM1 | IFITM1 | IFITM1 | IFITM1 | IFITM1 | IFITM1 | IFITM1 | IFITM1 | IFITM1 | IFITM1 | IFITM1 | IFITM1 | IFITM1 | | | | | | |
| C | CCND1 | CCND1 | CCND1 | CCND1 | CCND1 | CCND1 | CCND1 | CCND1 | CCND1 | CCND1 | CCND1 | CCND1 | CCND1 | CCND1 | CCND1 | CCND1 | CCND1 | CCND1 | | | | | | |
| D | ps2 | ps2 | ps2 | ps2 | ps2 | ps2 | ps2 | ps2 | ps2 | ps2 | ps2 | ps2 | ps2 | ps2 | ps2 | ps2 | ps2 | ps2 | | | | | | |
| E | CTSD | CTSD | CTSD | CTSD | CTSD | CTSD | CTSD | CTSD | CTSD | CTSD | CTSD | CTSD | CTSD | CTSD | CTSD | CTSD | CTSD | CTSD | | | | | | |
| F | FOXA1 | FOXA1 | FOXA1 | FOXA1 | FOXA1 | FOXA1 | FOXA1 | FOXA1 | FOXA1 | FOXA1 | FOXA1 | FOXA1 | FOXA1 | FOXA1 | FOXA1 | FOXA1 | FOXA1 | FOXA1 | | | | | | |
| G | c-myc | c-myc | c-myc | c-myc | c-myc | c-myc | c-myc | c-myc | c-myc | c-myc | c-myc | c-myc | c-myc | c-myc | c-myc | c-myc | c-myc | c-myc | | | | | | |
| H | ER | ER | ER | ER | ER | ER | ER | ER | ER | ER | ER | ER | ER | ER | ER | ER | ER | ER | | | | | | |
| | 5C siCon | | | 5C siSTAT1 | | | 5C siSTAT2 | | | MCF7 veh | | | MCF7 ITA | | | MCF7 Rux | | | | | | | | |
| I | PUM1 | PUM1 | PUM1 | PUM1 | PUM1 | PUM1 | PUM1 | PUM1 | PUM1 | PUM1 | PUM1 | PUM1 | PUM1 | PUM1 | PUM1 | PUM1 | PUM1 | PUM1 | | | | | | |
| J | IFITM1 | IFITM1 | IFITM1 | IFITM1 | IFITM1 | IFITM1 | IFITM1 | IFITM1 | IFITM1 | IFITM1 | IFITM1 | IFITM1 | IFITM1 | IFITM1 | IFITM1 | IFITM1 | IFITM1 | IFITM1 | | | | | | |
| K | STAT1 | STAT1 | STAT1 | STAT1 | STAT1 | STAT1 | STAT1 | STAT1 | STAT1 | CTSD | CTSD | CTSD | CTSD | CTSD | CTSD | | | | | | | | | |
| L | STAT2 | STAT2 | STAT2 | STAT2 | STAT2 | STAT2 | STAT2 | STAT2 | STAT2 | C-Myc | C-Myc | C-Myc | C-Myc | C-Myc | C-Myc | | | | | | | | | |
| M | IRF9 | IRF9 | IRF9 | IRF9 | IRF9 | IRF9 | IRF9 | IRF9 | IRF9 | | | | | | | | | | | | | | | |
| N | | | | | | | | | | | | | | | | | | | | | | | | |
| O | | | | | | | | | | | | | | | | | | | | | | | | |
| P | | | | | | | | | | | | | | | | | | | | | | | | |

| 5C ITA | | |
|---|---|---|
| PUM1 | PUM1 | PUM1 |
| IFITM1 | IFITM1 | IFITM1 |
| CCND1 | CCND1 | CCND1 |
| ps2 | ps2 | ps2 |
| CTSD | CTSD | CTSD |
| FOXA1 | FOXA1 | FOXA1 |
| c-myc | c-myc | c-myc |
| ER | ER | ER |

| MCF7 veh | | | MCF7 IFNAR Nab | | |
|---|---|---|---|---|---|
| PUM1 | PUM1 | PUM1 | PUM1 | PUM1 | PUM1 |
| IFITM1 | IFITM1 | IFITM1 | IFITM1 | IFITM1 | IFITM1 |
| CCND1 | CCND1 | CCND1 | CCND1 | CCND1 | CCND1 |
| ps2 | ps2 | ps2 | ps2 | ps2 | ps2 |
| CTSD | CTSD | CTSD | CTSD | CTSD | CTSD |
| FOXA1 | FOXA1 | FOXA1 | FOXA1 | FOXA1 | FOXA1 |
| c-myc | c-myc | c-myc | c-myc | c-myc | c-myc |
| ER | ER | ER | ER | ER | ER |

| MCF7 siCon | | | MCF7 siSTAT1 | | | MCF7 siSTAT2 | | |
|---|---|---|---|---|---|---|---|---|
| STAT1 | STAT1 | STAT1 | STAT1 | STAT1 | STAT1 | STAT1 | STAT1 | STAT1 |
| STAT2 | STAT2 | STAT2 | STAT2 | STAT2 | STAT2 | STAT2 | STAT2 | STAT2 |
| IRF9 | IRF9 | IRF9 | IRF9 | IRF9 | IRF9 | IRF9 | IRF9 | IRF9 |

| 2 primers | each primer uL | x | uL |
|---|---|---|---|
| Sybr Green | 5 | 40 | 200 |
| Sterile H20 | 3.9 | | 156 |
| Primers | 0.1 | | 4 |
| cDNA | 1 | | |

| 2 primers | each primer uL | x | uL |
|---|---|---|---|
| Sybr Green | 5 | 27 | 135 |
| Sterile H20 | 3.9 | | 105.3 |
| Primers | 0.1 | | 2.7 |
| cDNA | 1 | | |

| 4 primers | each primer uL | x | uL |
|---|---|---|---|
| Sybr Green | 5 | 21 | 105 |
| Sterile H20 | 3.9 | | 81.9 |
| Primers | 0.1 | | 2.1 |
| cDNA | 1 | | |

1270

| Total | uL | x | uL |
|---|---|---|---|
| Sybr Green | 5 | ~~100~~ 254 | ~~500~~ |
| Sterile H20 | 3.9 | | ~~390~~ ← 990.6 |
| Primers | 0.1 | | ~~10~~ |
| cDNA | 1 | | |

| | |
|---|---|
| PUM1 | 40 wells |
| IFITM1 | 40 wells |
| CCND1 | 21 |
| PS2 | 21 |
| CTSD | 27 |
| FOXA1 | 21 |
| Cmyc | 27 |
| ER | 21 |
| STAT1 | 12 |
| STAT2 | 12 |
| IRF9 | 12 |

| 3 primers | each primer uL | x | uL |
|---|---|---|---|
| Sybr Green | 5 | 12 | 60 |
| Sterile H20 | 3.9 | | 46.8 |
| Primers | 0.1 | | 1.2 |
| cDNA | 1 | | |

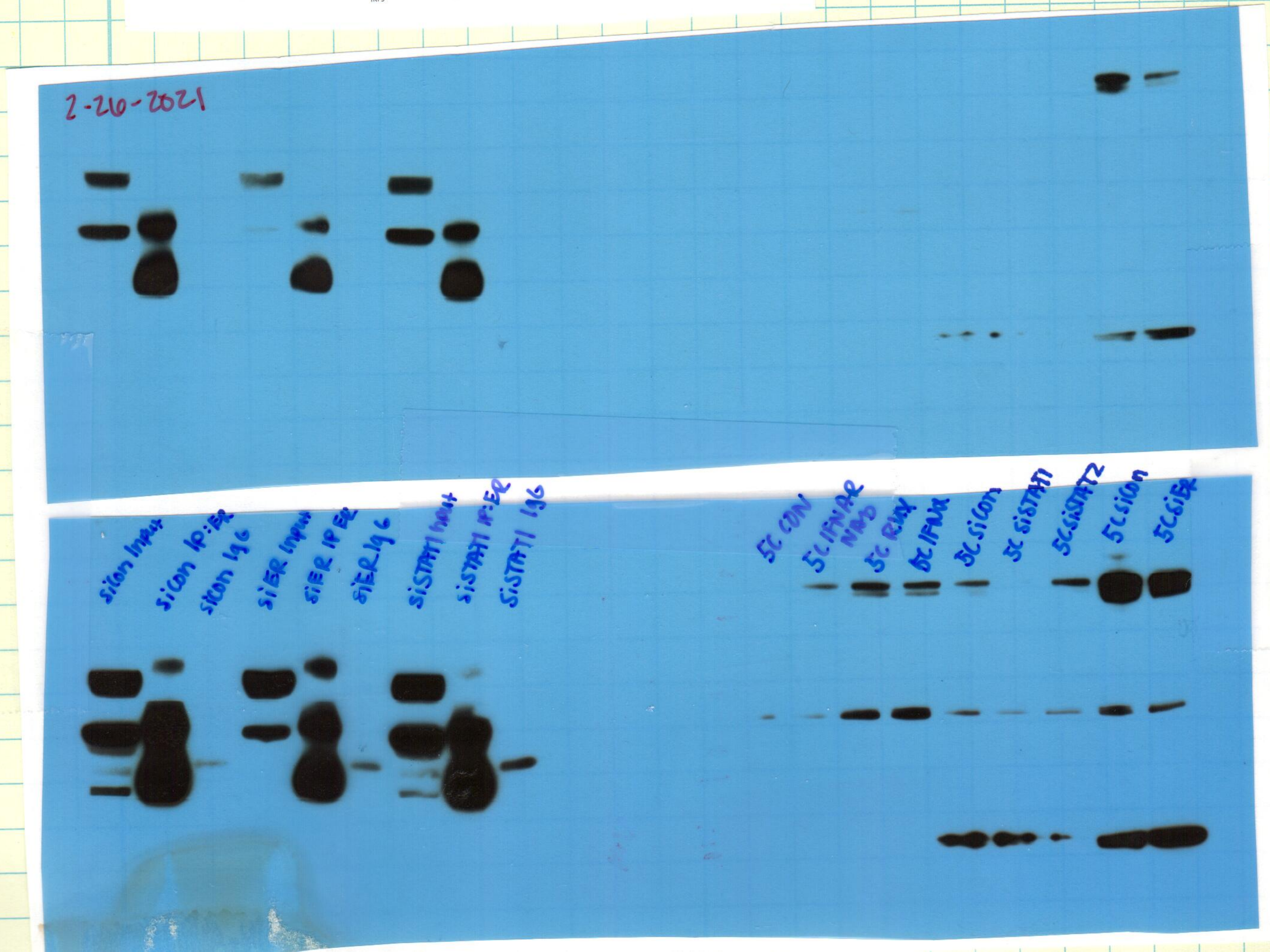

Supplement: Supplementary file 1 [file cancers-13-05130-s001.zip › cancers-1384109-supplementary/cancers-1384109-western blot/ER paper WBs/Western Scans - Lab Notebook 4/WB0005.pdf]
