# Supplementary material for: Enhanced IFNα Signaling Promotes Ligand-Independent Activation of ERα to Promote Aromatase Inhibitor Resistance in Breast Cancer
Source: Cancers (Basel). 2021 Oct 13;13(20):5130. doi: 10.3390/cancers13205130 (PMC8534010; doi:10.3390/cancers13205130)
Supplement: Supplementary file 1 [file cancers-13-05130-s001.zip › cancers-1384109-supplementary/cancers-1384109-western blot/ER paper WBs/Western Scans - Lab Notebook 4/WB0009.pdf]

[illegible]n western:

MCF7 con IFNAR NAb RUX MARKER sicon siSTAT1 siSTAT2 sicon siER MARKER

pSTAT2 STAT2
pSTAT1 STAT1

pERα ERα

IFITM1
β-actin.

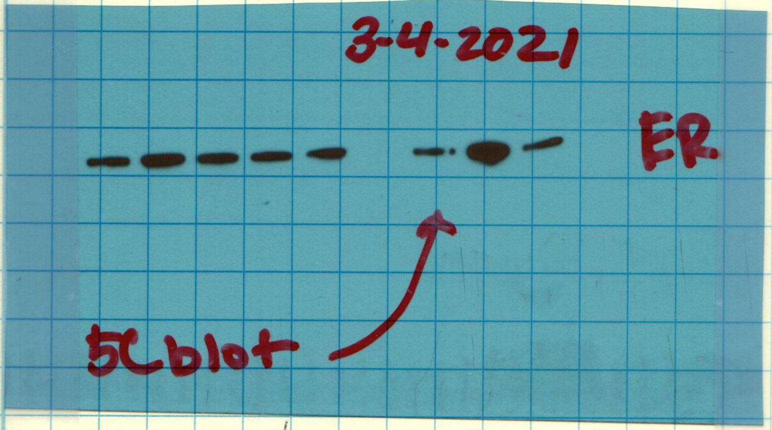

Too low protein on this blot
nothing but β-actin showed up.

			Count 1	Count 2	
149	sicon	1	9	11	in 500 uL
		2	31	25	in 200 uL
		3	39	40	↓
149	siXBP1	1	17	28	
		2	40	51	
		3	27	19	
468	sicon	1	93	68	
		2	98	97	
		3	134	83	
468	siXBP1	1	98	94	
		2	85	88	
		3	133	106	
