# Supplementary material for: Enhanced IFNα Signaling Promotes Ligand-Independent Activation of ERα to Promote Aromatase Inhibitor Resistance in Breast Cancer
Source: Cancers (Basel). 2021 Oct 13;13(20):5130. doi: 10.3390/cancers13205130 (PMC8534010; doi:10.3390/cancers13205130)
Supplement: Supplementary file 1 [file cancers-13-05130-s001.zip › cancers-1384109-supplementary/cancers-1384109-western blot/ER paper WBs/Western Scans - Lab Notebook 4/WB0014.pdf]

8-24-2021

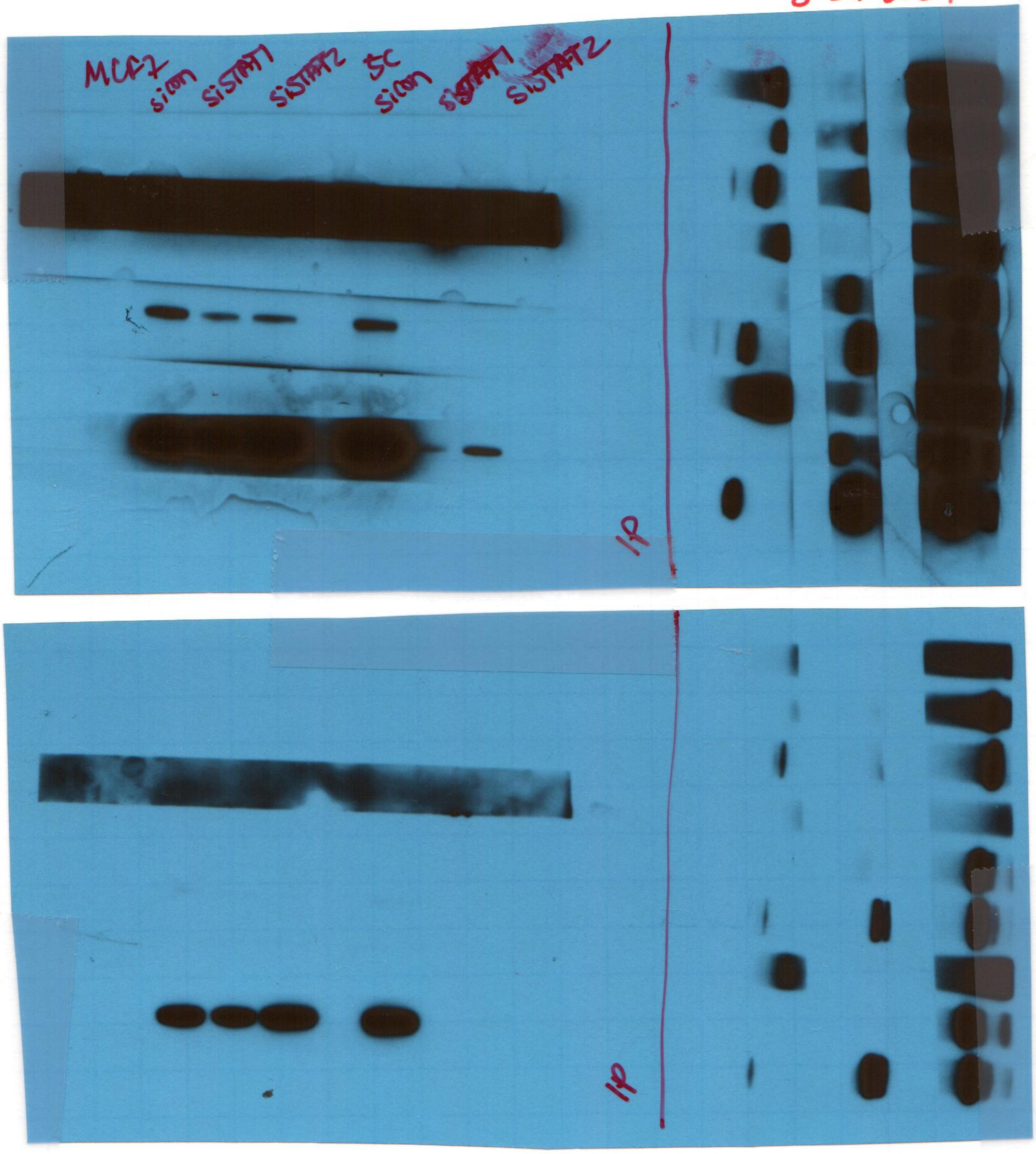

/25/2021

		Count 1	Count 2
149 cap sicon DMSO	1	21	20
	2	43	55
	3	56	63
149 cap sicon ATO	1	72	59
	2	85	80
	3	74	73
149 cap SIXBP1 DMSO	1	122	96
	2	94	68
	3	64	65
149 cap SIXBP1 ATO	1	107	81
	2	59	54
	3	128	124
149 KOP sicon DMSO	1	59	32
	2	20	34
	3	27	30
149 KOP sicon ATO	1	23	30
	2	26	29
	3	18	31

[illegible]
333 uL
