# Supplementary material for: Enhanced IFNα Signaling Promotes Ligand-Independent Activation of ERα to Promote Aromatase Inhibitor Resistance in Breast Cancer
Source: Cancers (Basel). 2021 Oct 13;13(20):5130. doi: 10.3390/cancers13205130 (PMC8534010; doi:10.3390/cancers13205130)

2.4 ug/ul

20_4_2021	Cell Line	Sample #	con. (ug/u	Sample B.	Protein	D.W.	Total
	1411 PolyT KO Tumor - 14w	1	3.2	7.50	***22.64***	***-0.14***	30.00
	1500 PolyT WT MG - 8w	2	3.4	7.50	***21.36***	***1.14***	30.00
	1496 WT MG	3	8.0	7.50	***8.97***	***13.53***	30.00
	1497 KO MG	4	8.0	7.50	***8.97***	***13.53***	30.00
	1501 PolyT KO MG - 8w	5	3.9	7.50	***18.32***	***4.18***	30.00
	1512 PolyT WT Tumor 8w	6	3.2	7.50	***22.78***	***-0.28***	30.00

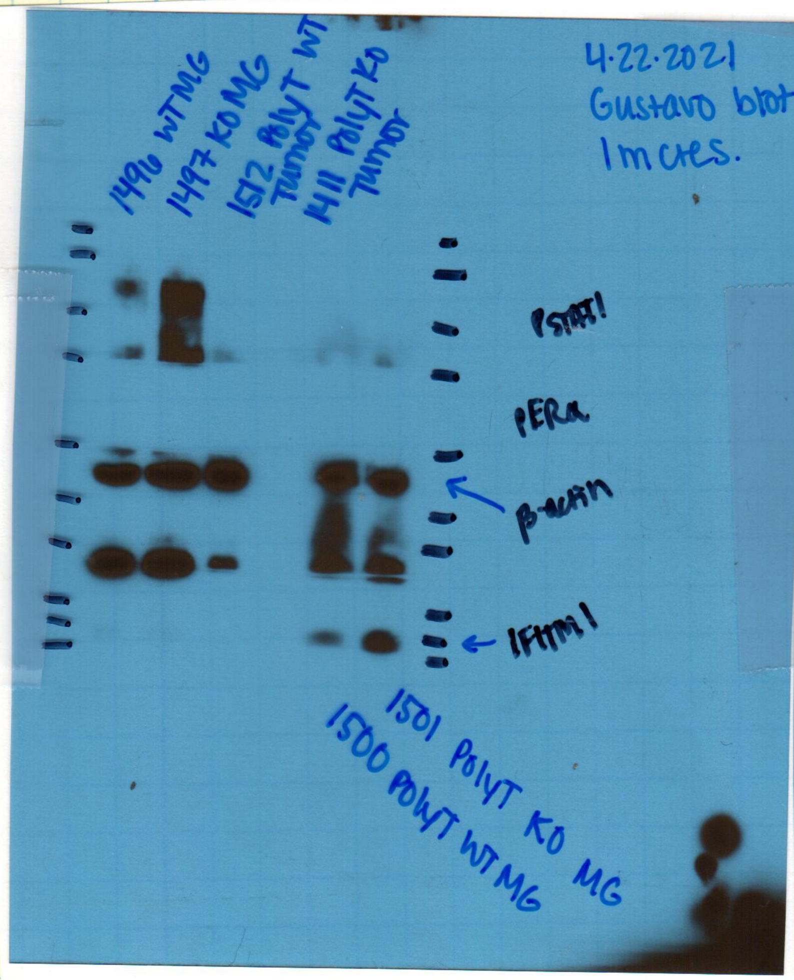

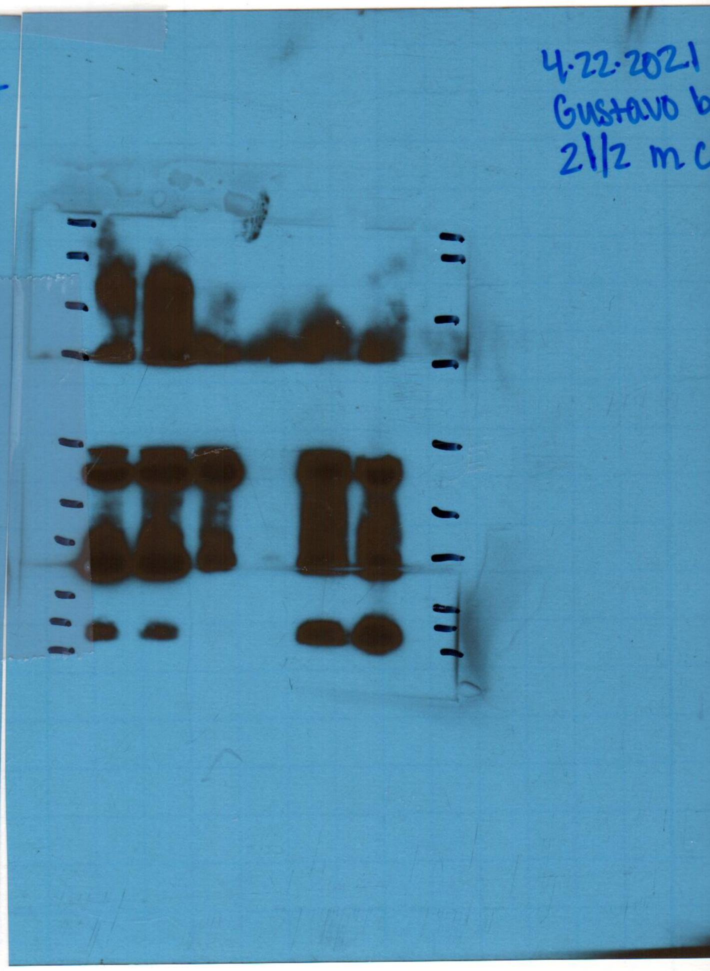

Supplement: Supplementary file 1 [file cancers-13-05130-s001.zip › cancers-1384109-supplementary/cancers-1384109-western blot/ER paper WBs/Western Scans - Lab Notebook 4/WB0021.pdf]
