# Supplementary material for: Enhanced IFNα Signaling Promotes Ligand-Independent Activation of ERα to Promote Aromatase Inhibitor Resistance in Breast Cancer
Source: Cancers (Basel). 2021 Oct 13;13(20):5130. doi: 10.3390/cancers13205130 (PMC8534010; doi:10.3390/cancers13205130)

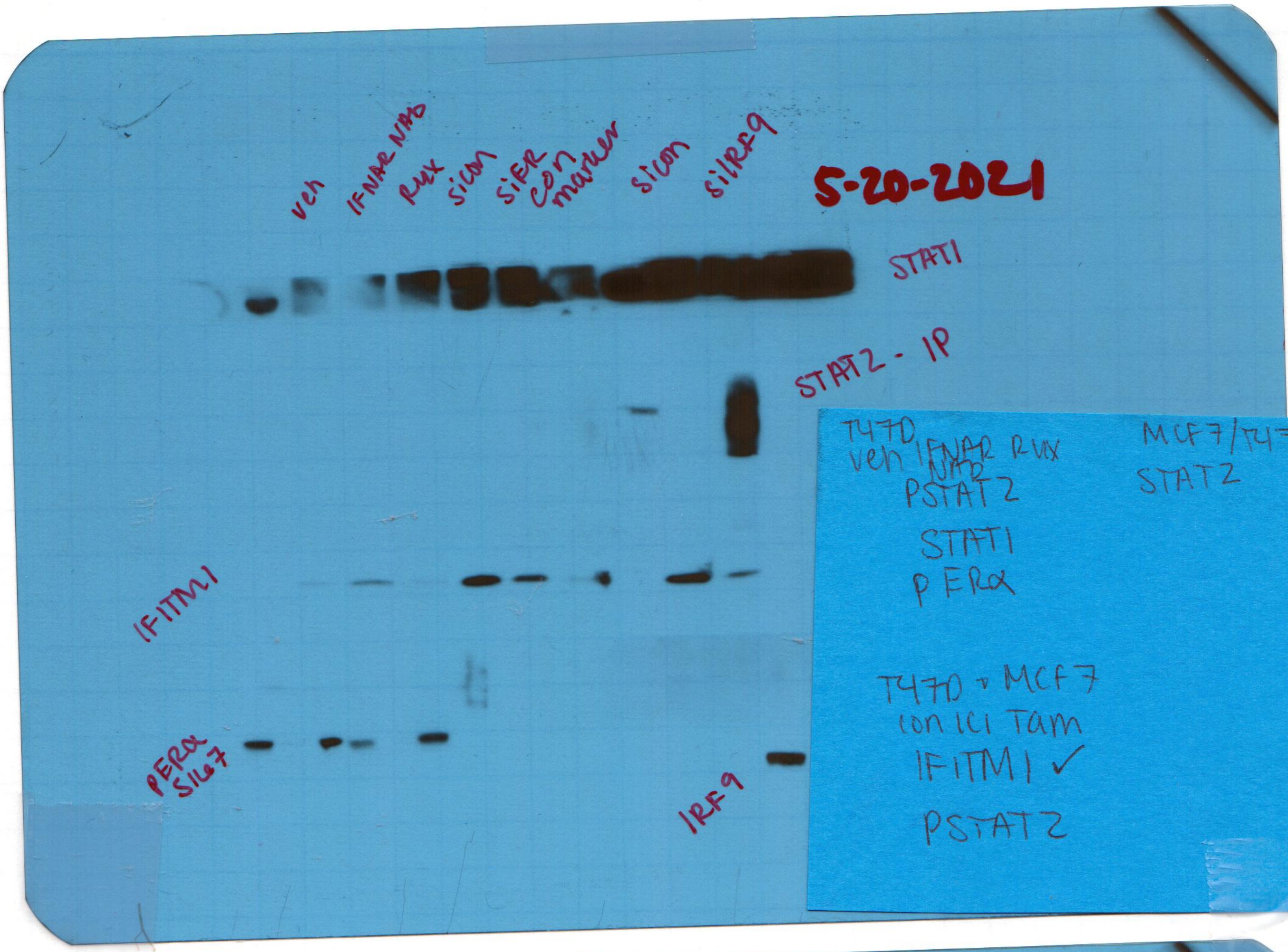

T47D
veh IFNAR NAb Rux
PSTAT2
STAT1
p ERα

MCF7/T47D IP
STAT2

T47D + MCF7
con ICI Tam
IFITM1 ✓
PSTAT2

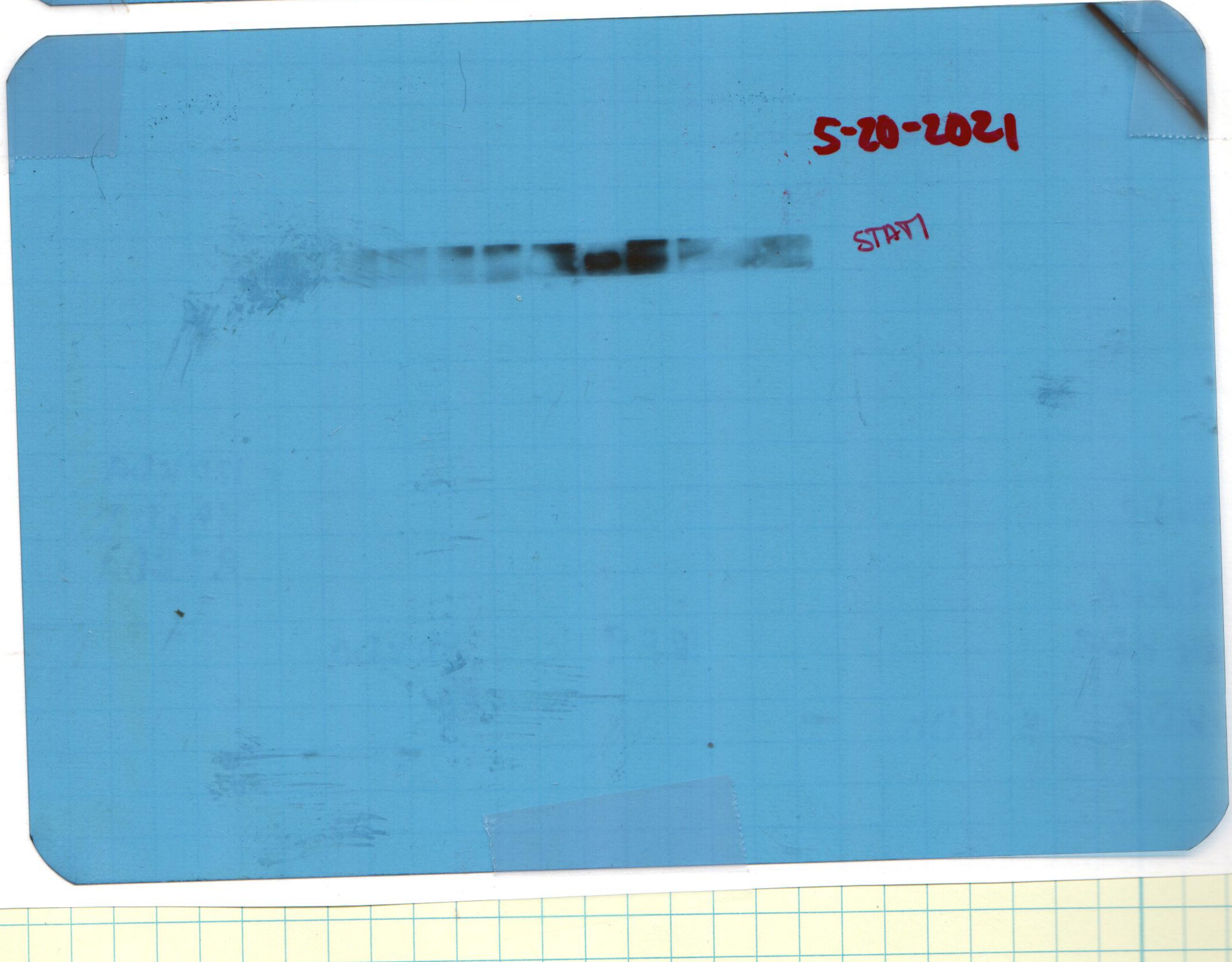

Supplement: Supplementary file 1 [file cancers-13-05130-s001.zip › cancers-1384109-supplementary/cancers-1384109-western blot/ER paper WBs/Western Scans - Lab Notebook 4/WB0035.pdf]
