# Supplementary figures and images for: Enhanced IFNα Signaling Promotes Ligand-Independent Activation of ERα to Promote Aromatase Inhibitor Resistance in Breast Cancer
Source: Cancers (Basel). 2021 Oct 13;13(20):5130. doi: 10.3390/cancers13205130 (PMC8534010; doi:10.3390/cancers13205130)

3.17.20

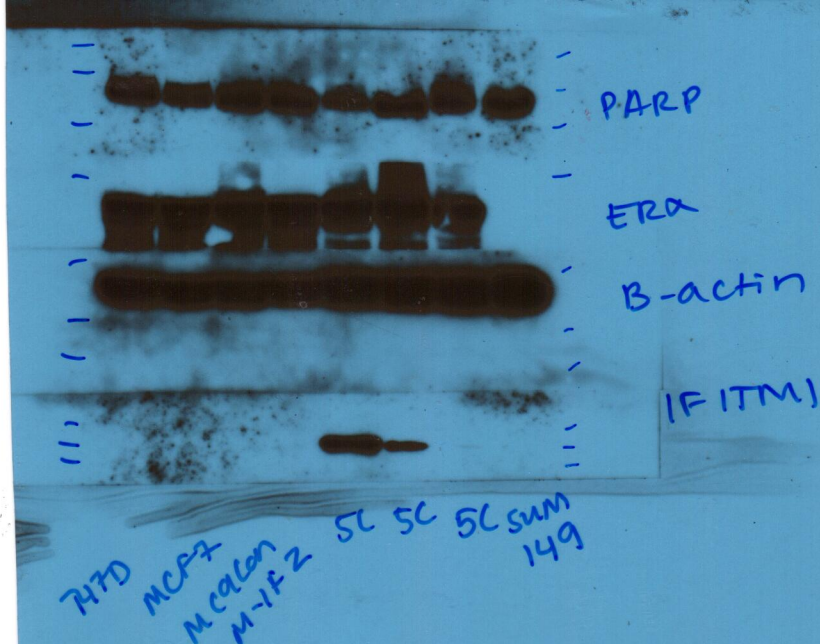

3.17.20

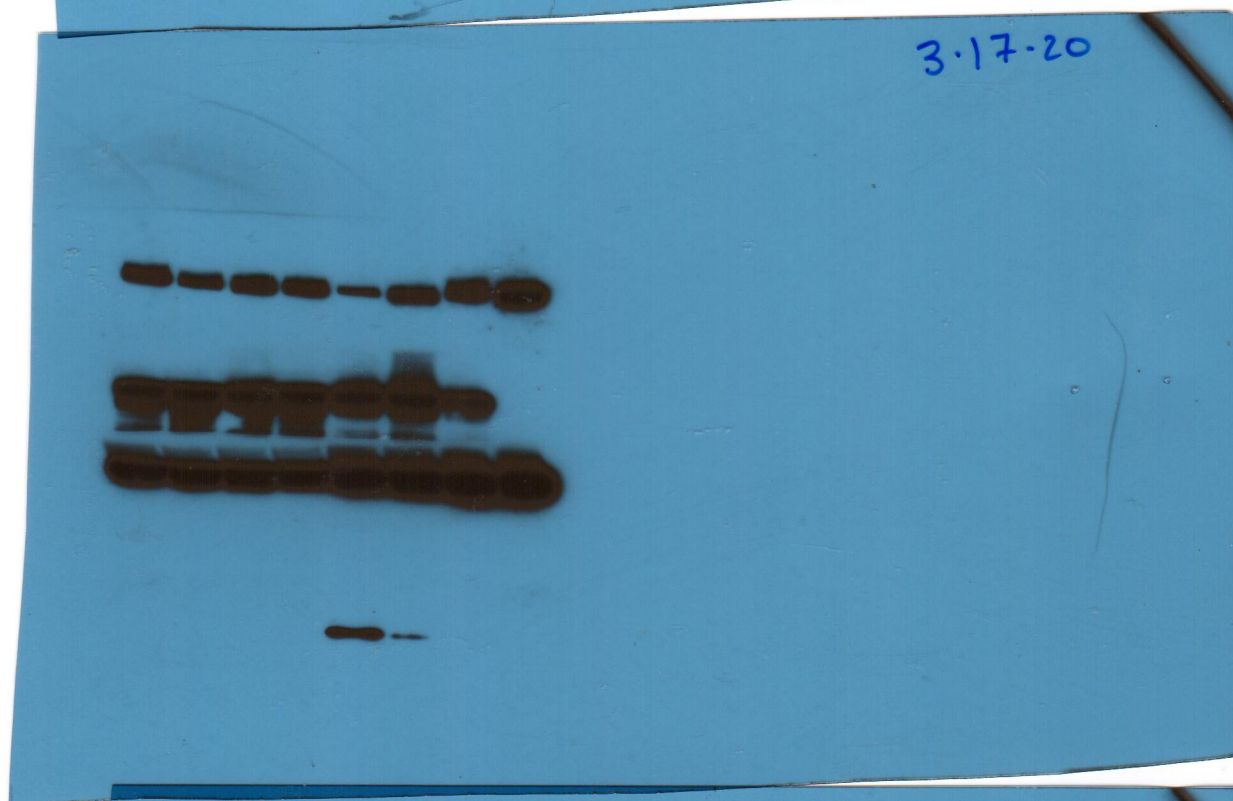

3.17.20

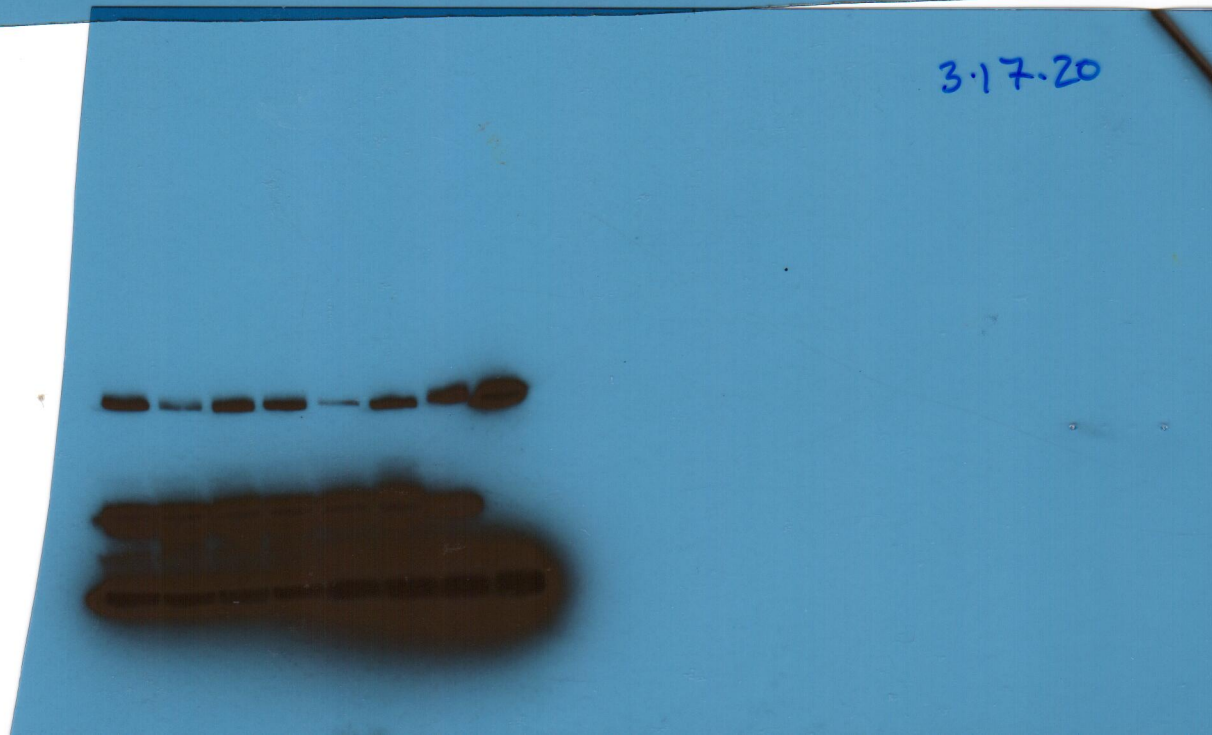

Supplement: Supplementary file 1 [file cancers-13-05130-s001.zip › cancers-1384109-supplementary/cancers-1384109-western blot/ER paper WBs/WB0001.pdf]

8-11-2020

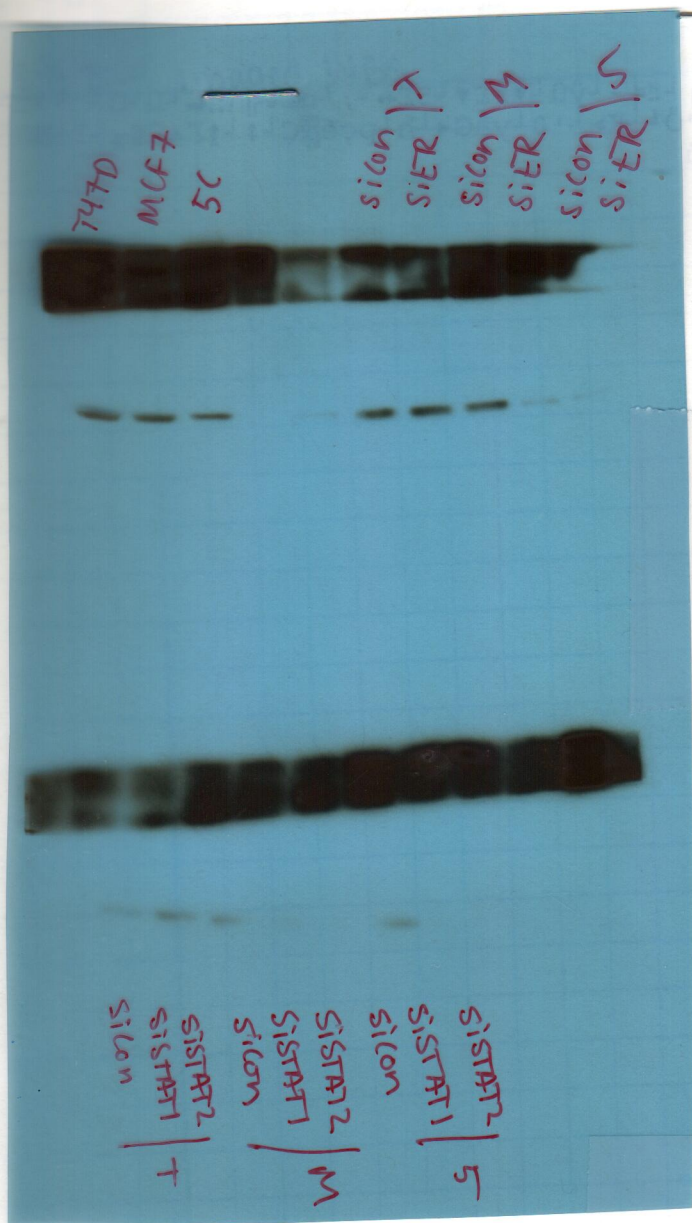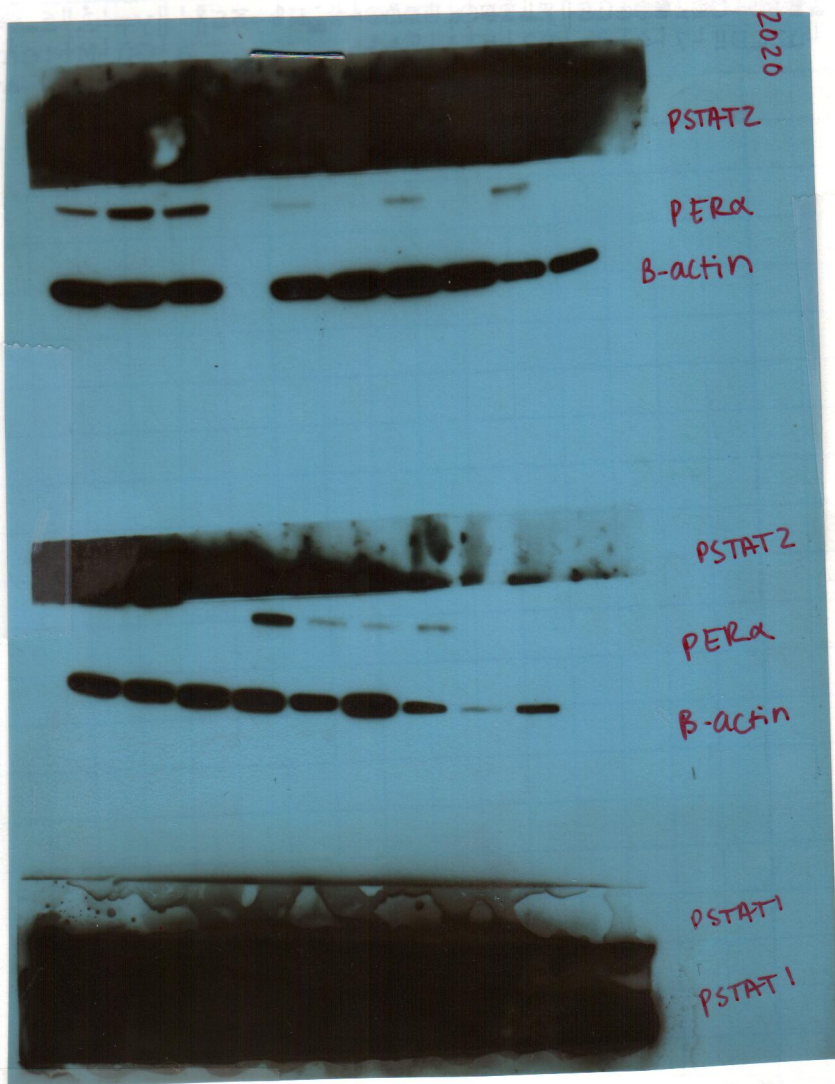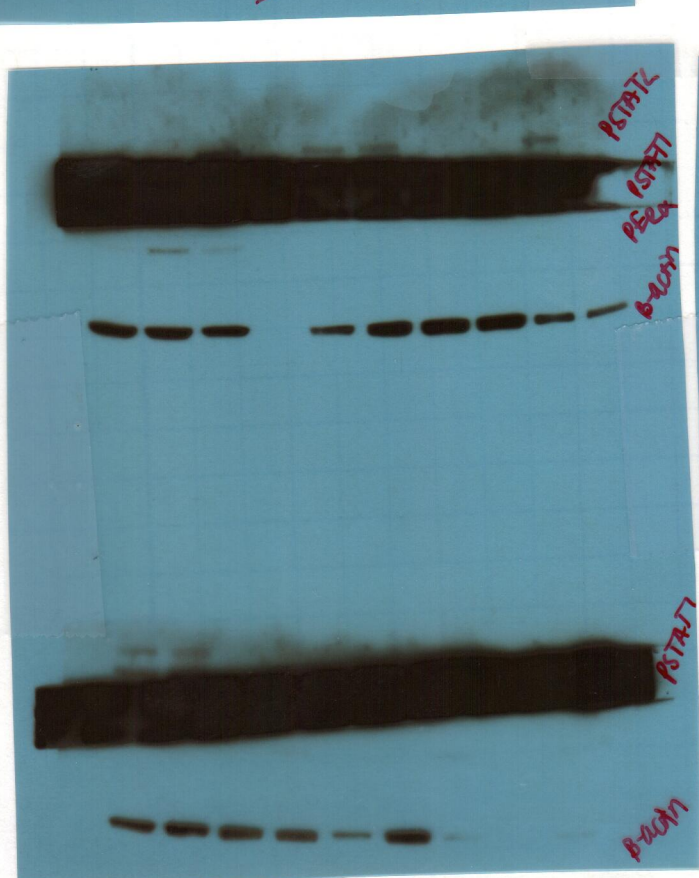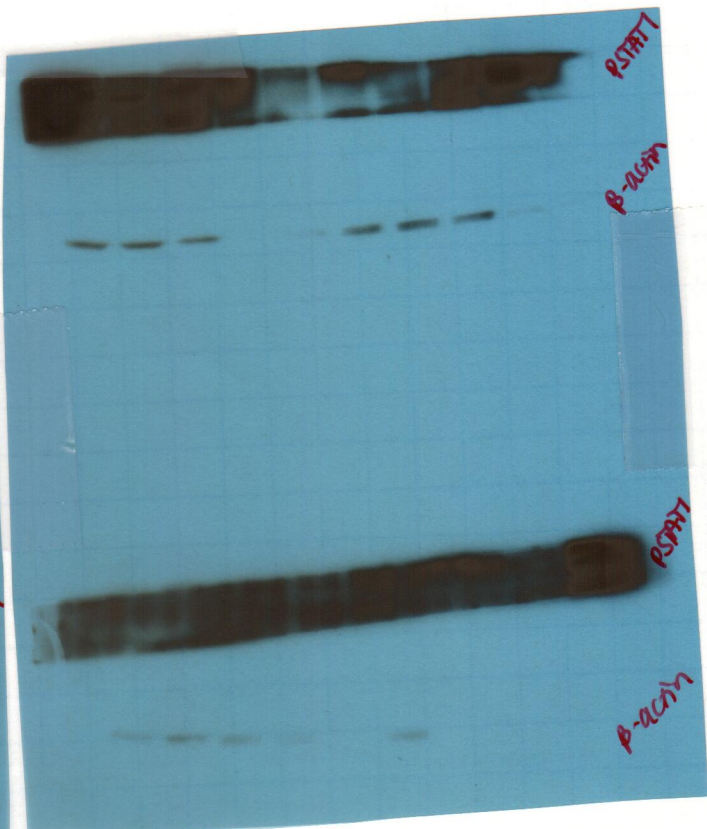

Supplement: Supplementary file 1 [file cancers-13-05130-s001.zip › cancers-1384109-supplementary/cancers-1384109-western blot/ER paper WBs/WB0003.pdf]

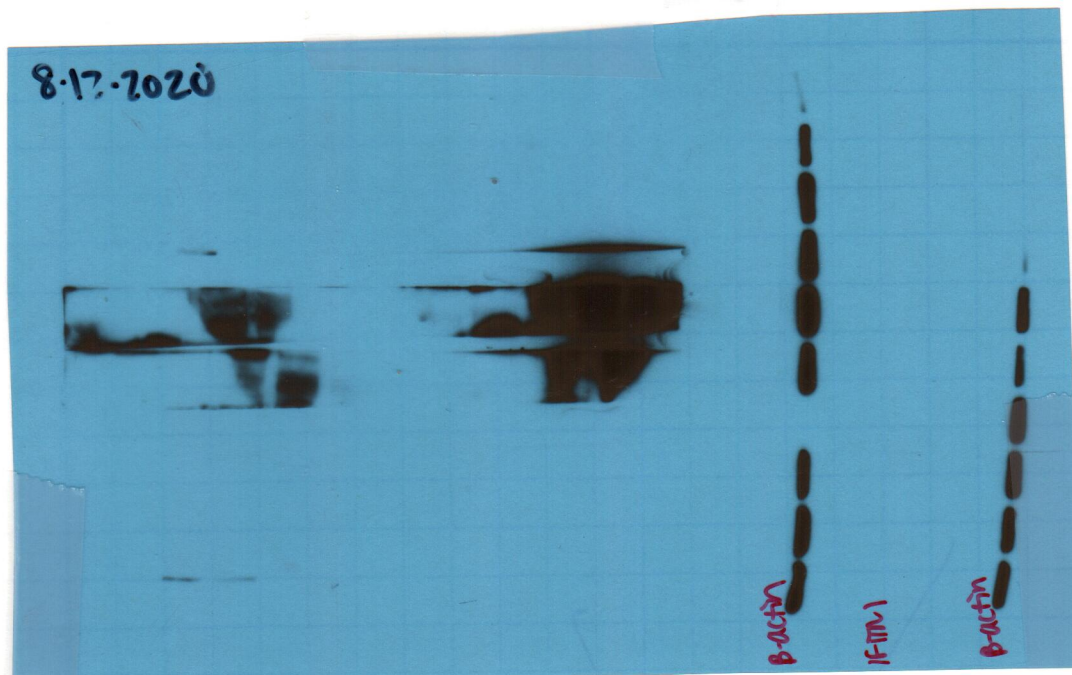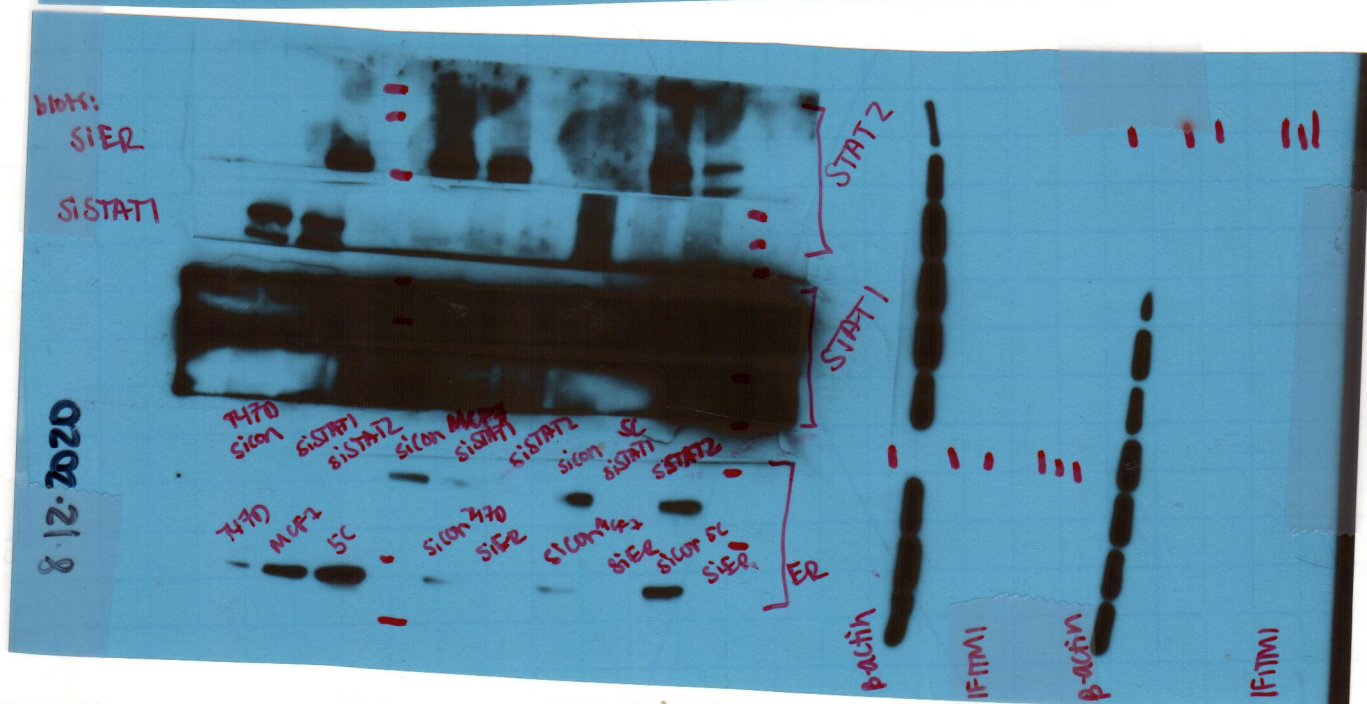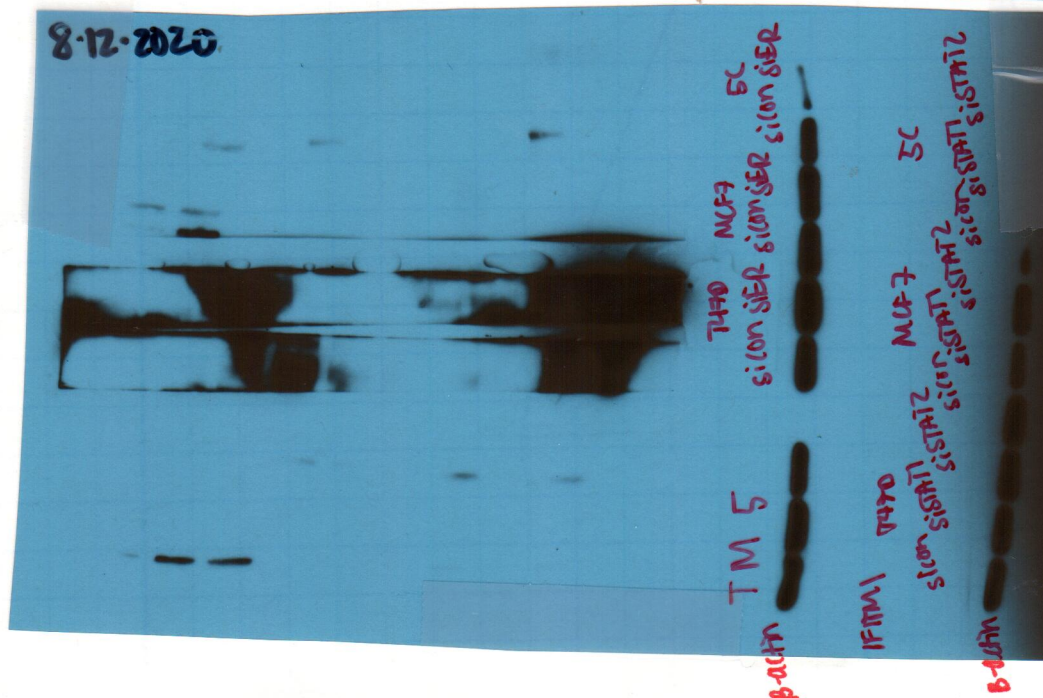

Supplement: Supplementary file 1 [file cancers-13-05130-s001.zip › cancers-1384109-supplementary/cancers-1384109-western blot/ER paper WBs/WB0005.pdf]

0202-31-8

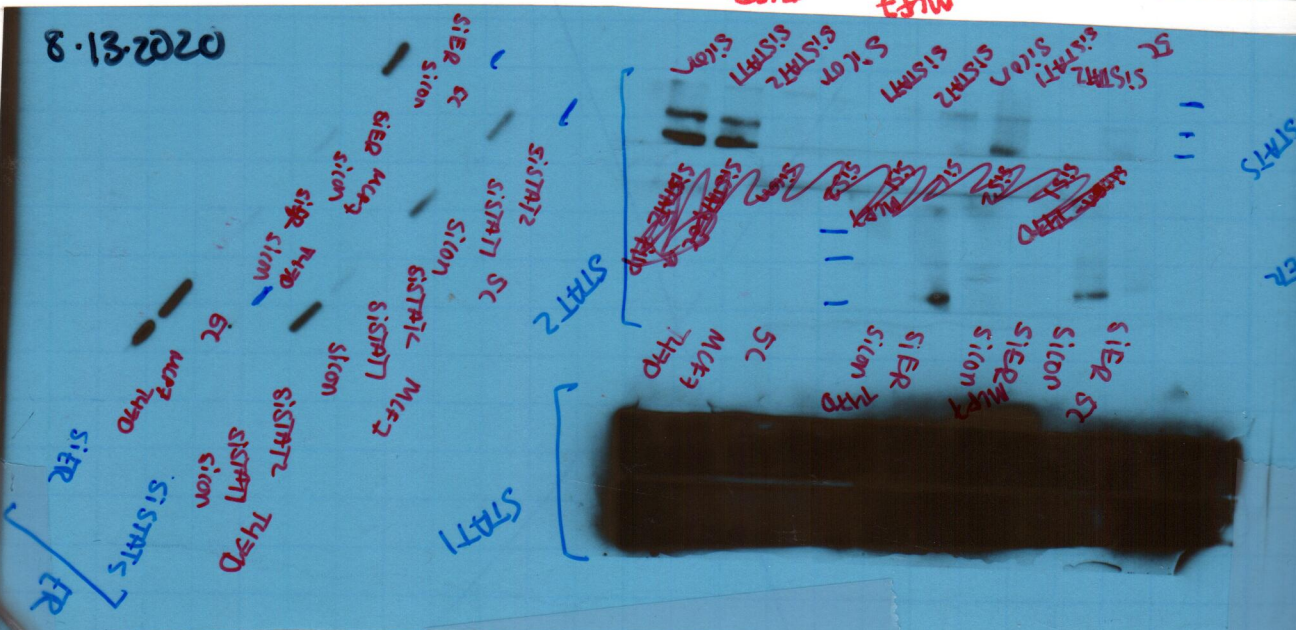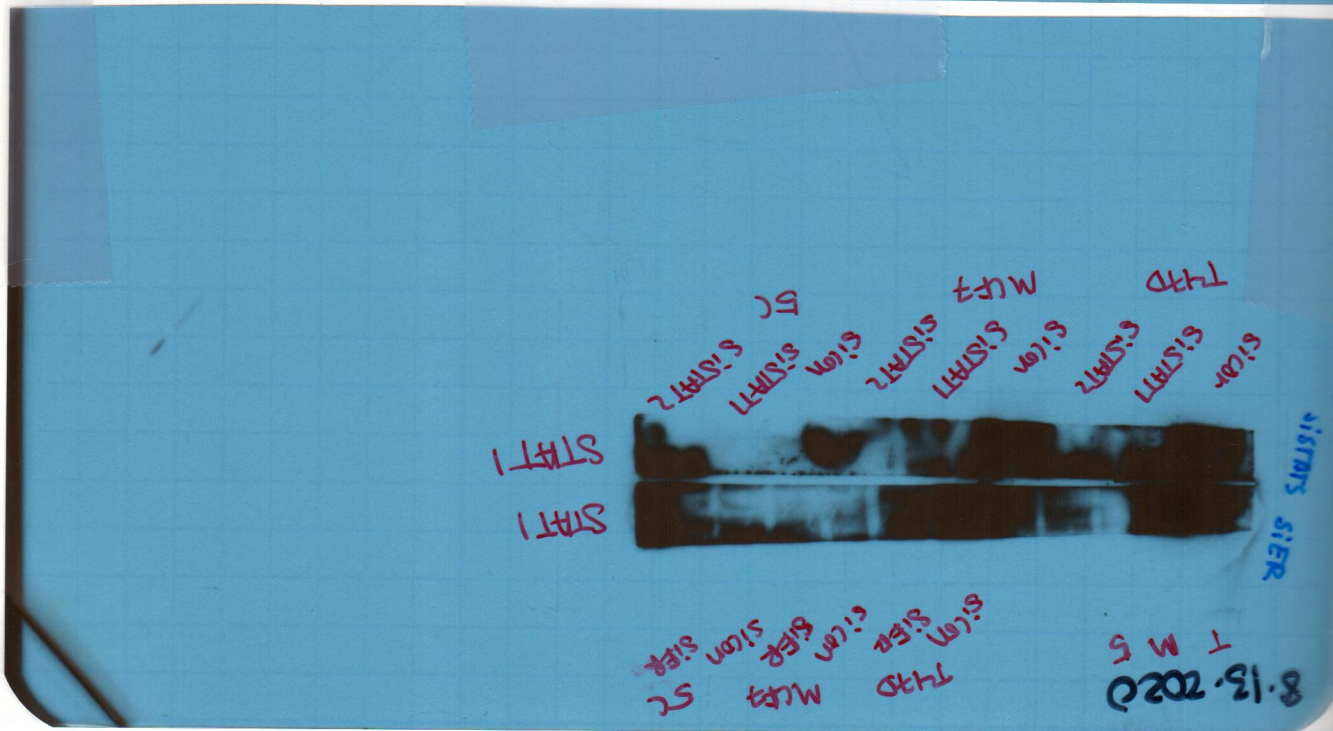

Supplement: Supplementary file 1 [file cancers-13-05130-s001.zip › cancers-1384109-supplementary/cancers-1384109-western blot/ER paper WBs/WB0006.pdf]

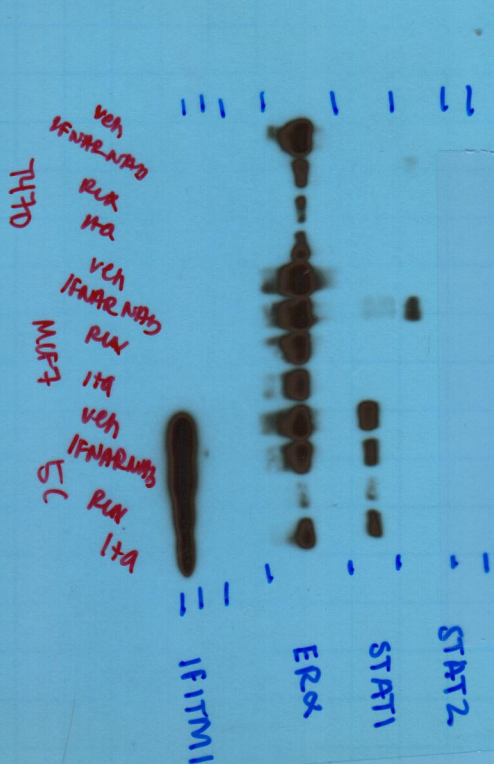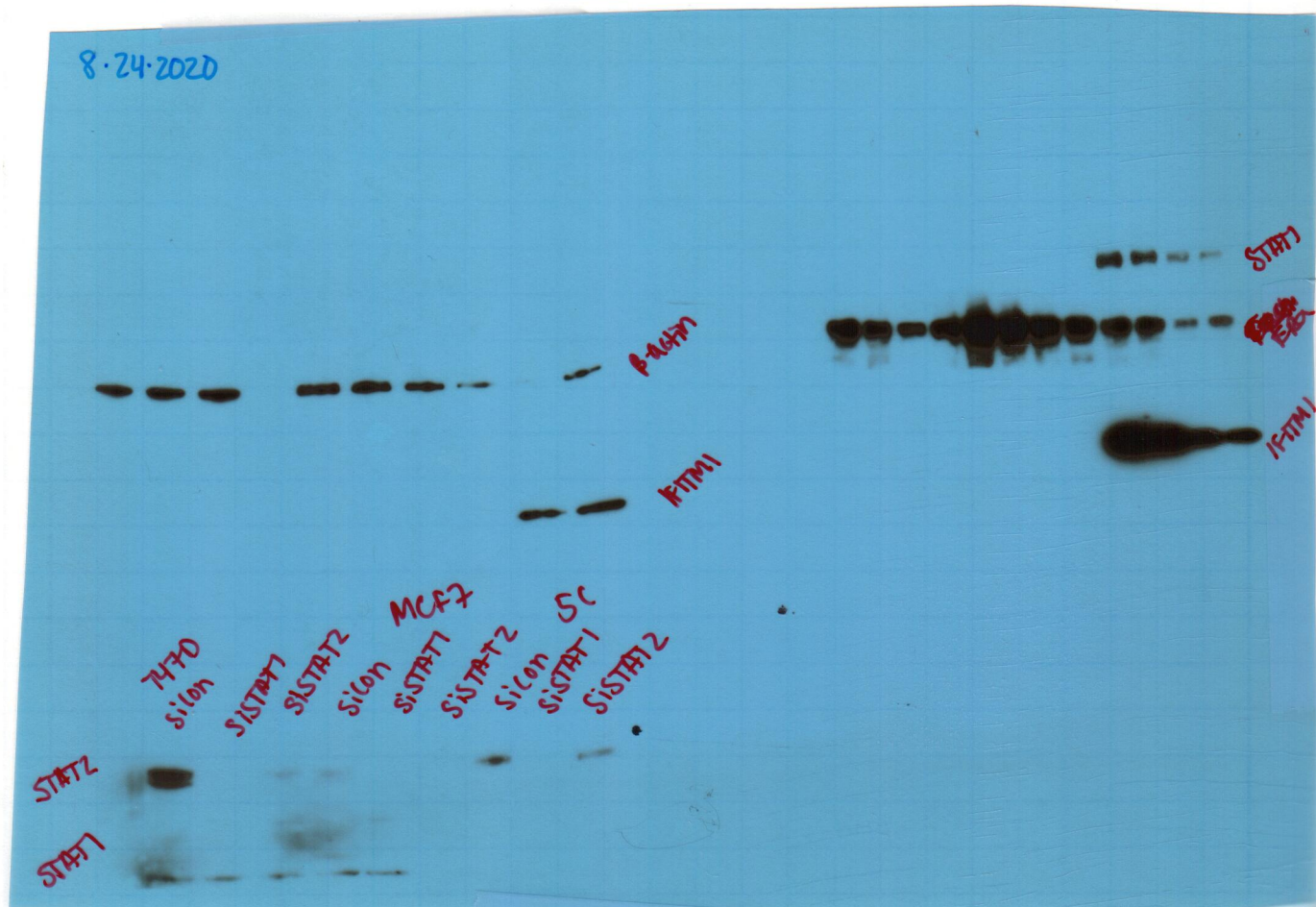

Supplement: Supplementary file 1 [file cancers-13-05130-s001.zip › cancers-1384109-supplementary/cancers-1384109-western blot/ER paper WBs/WB0009.pdf]

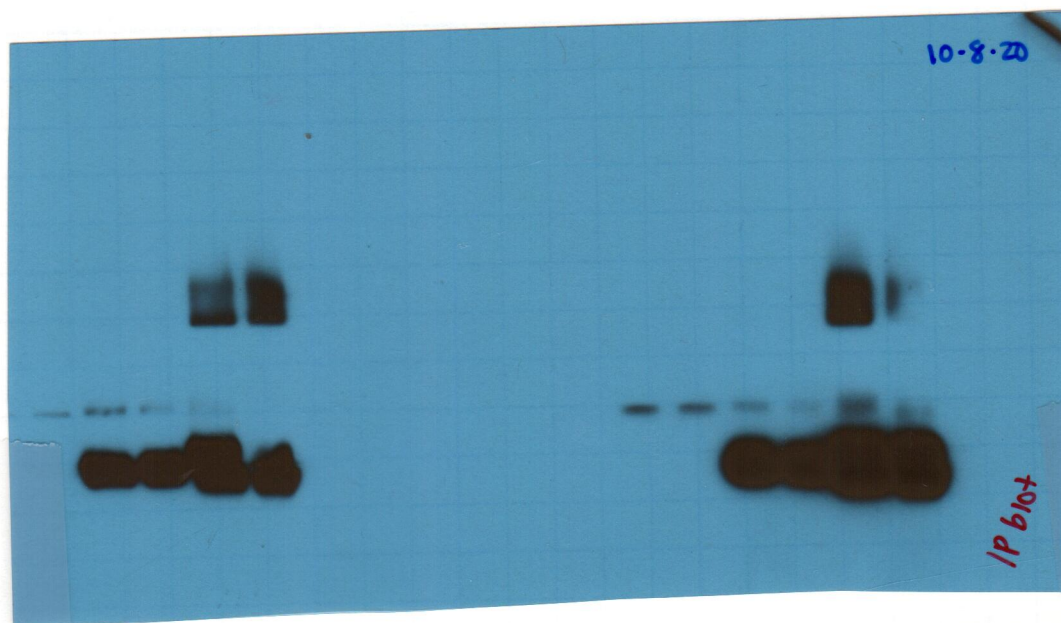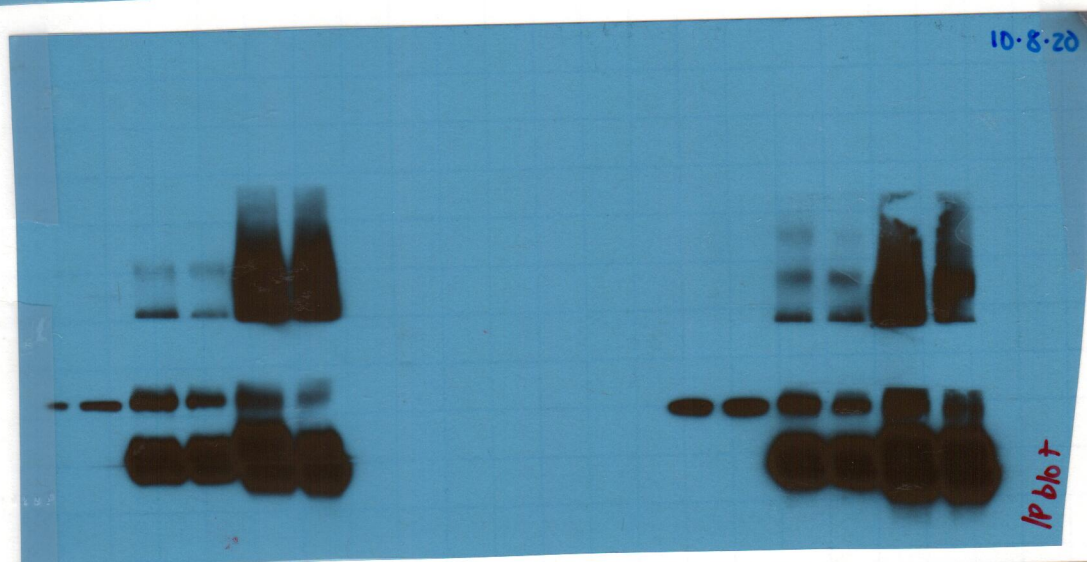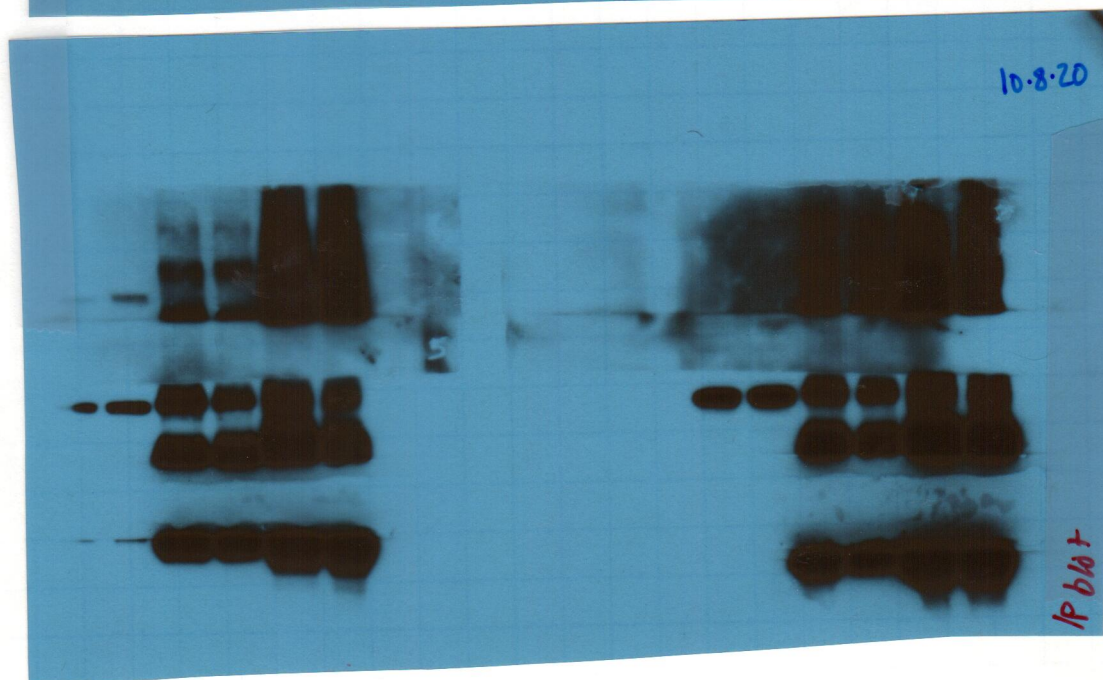

Supplement: Supplementary file 1 [file cancers-13-05130-s001.zip › cancers-1384109-supplementary/cancers-1384109-western blot/ER paper WBs/WB0013.pdf]

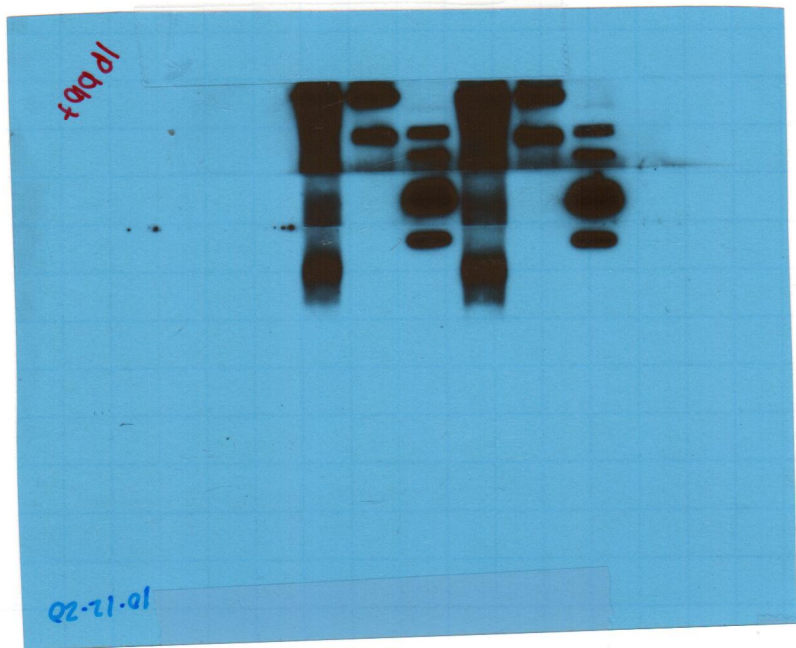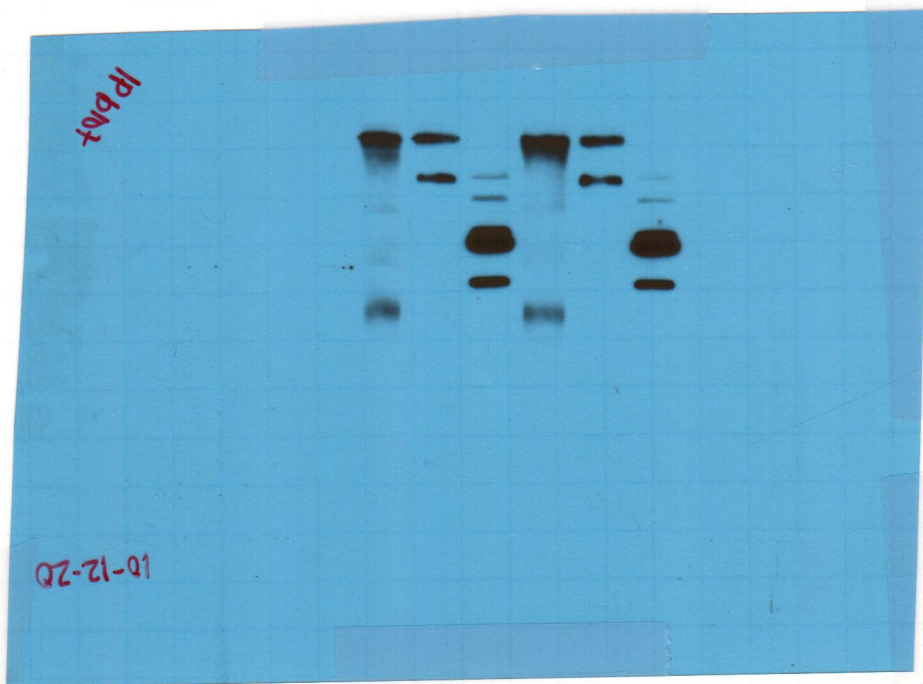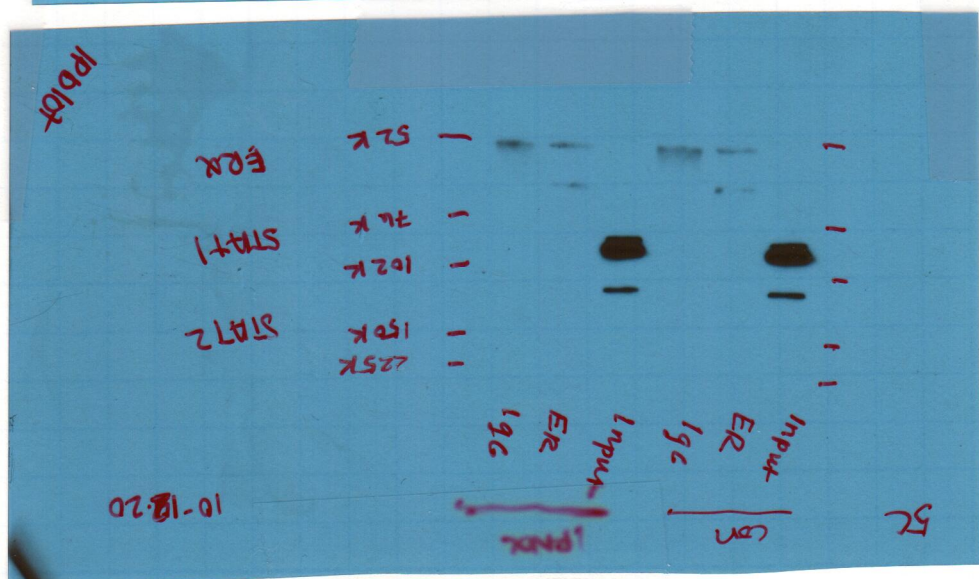

Supplement: Supplementary file 1 [file cancers-13-05130-s001.zip › cancers-1384109-supplementary/cancers-1384109-western blot/ER paper WBs/WB0014.pdf]

10-14-2020

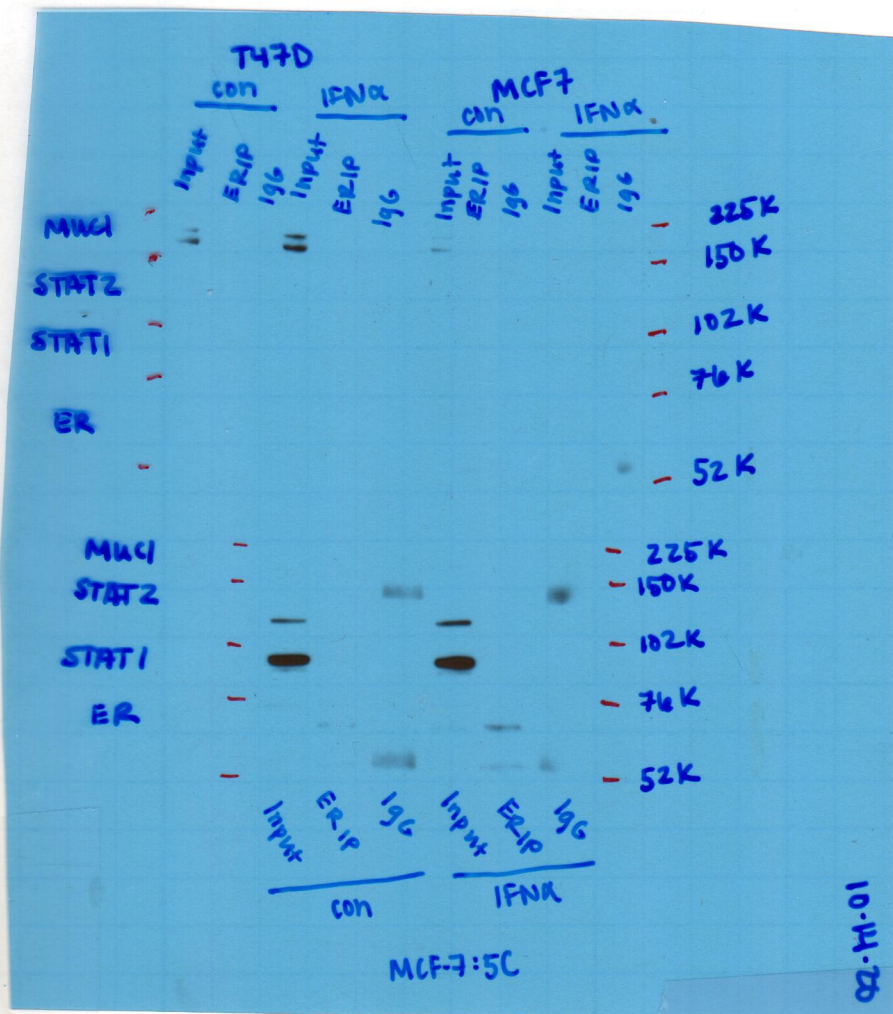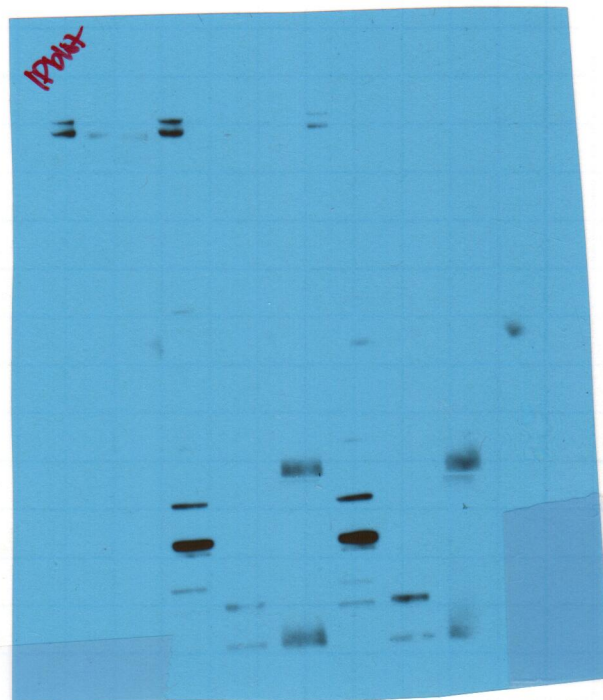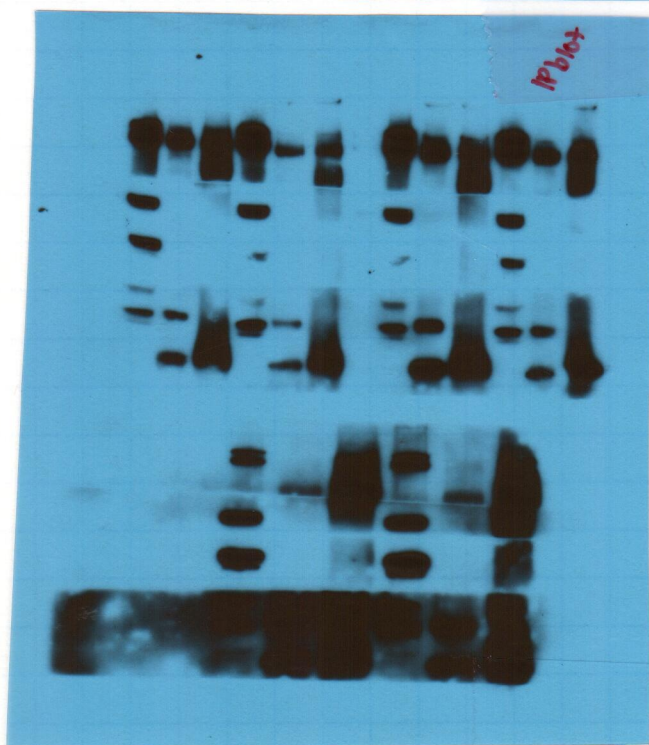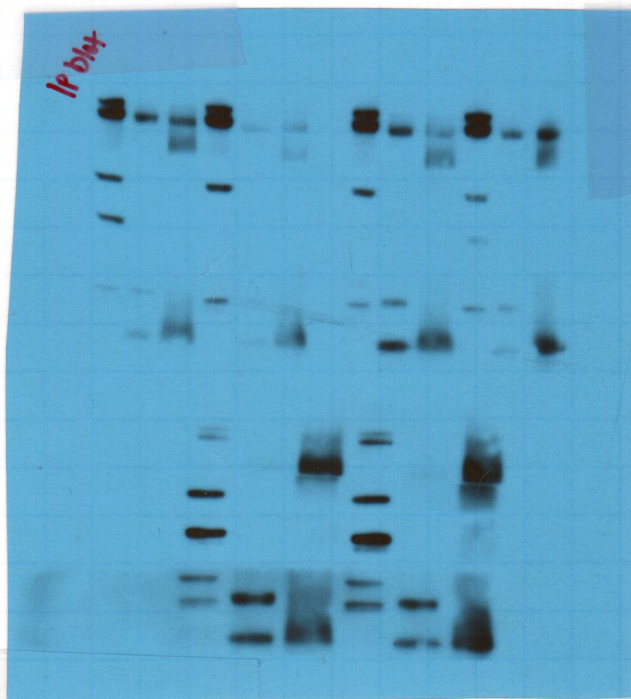

Supplement: Supplementary file 1 [file cancers-13-05130-s001.zip › cancers-1384109-supplementary/cancers-1384109-western blot/ER paper WBs/WB0015.pdf]

12-4-2019

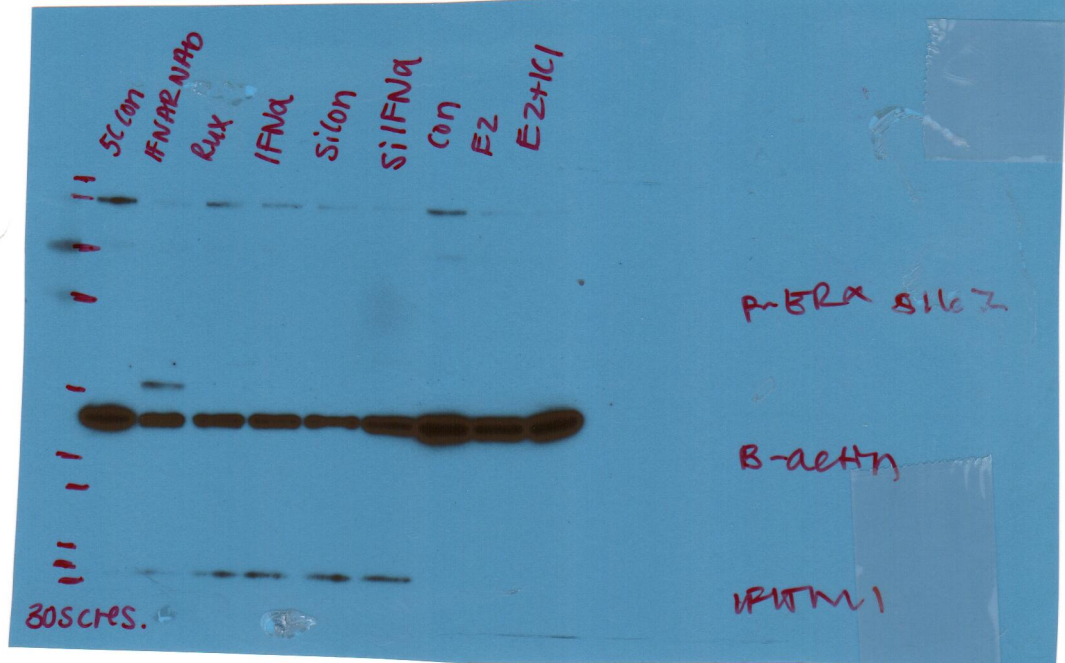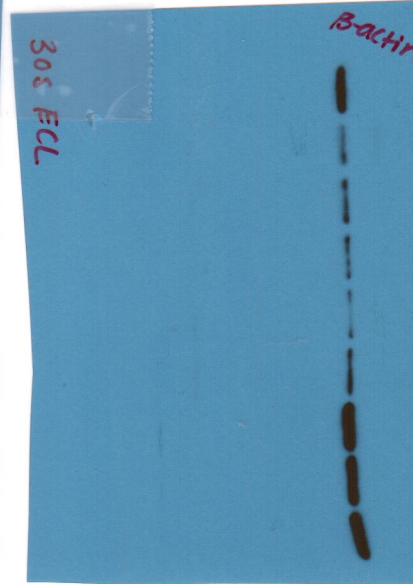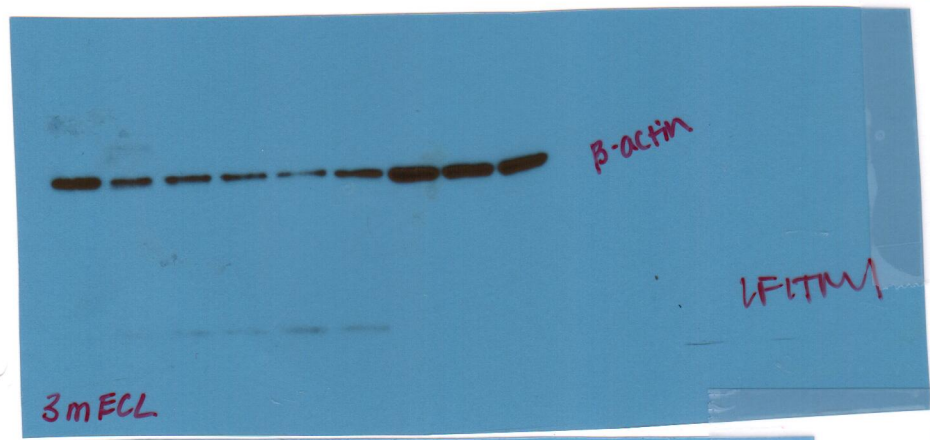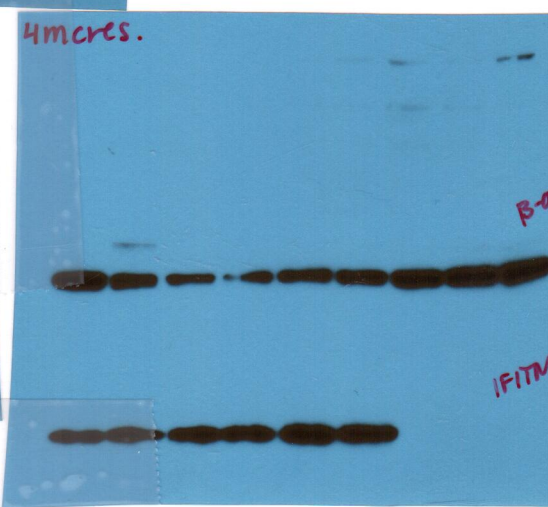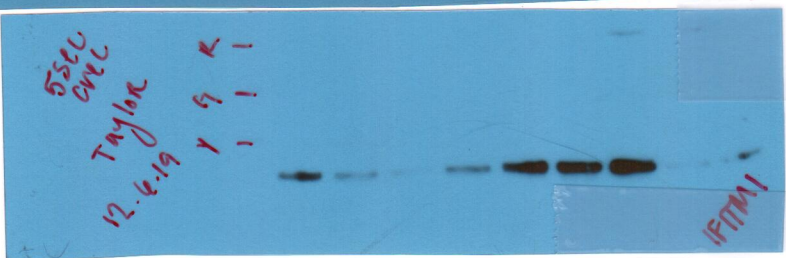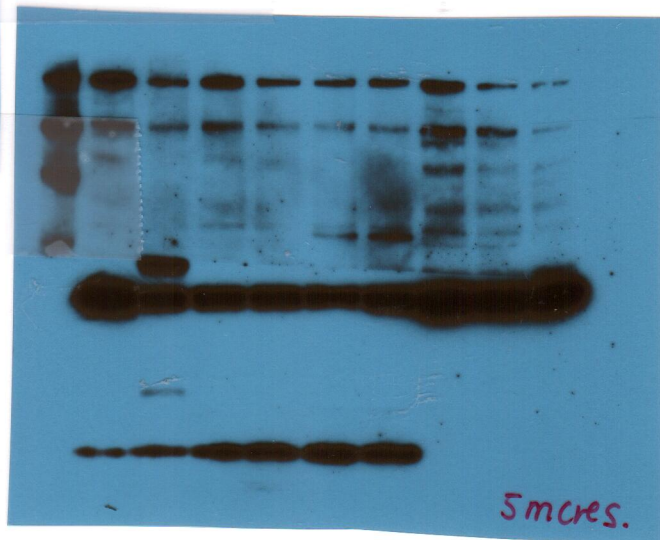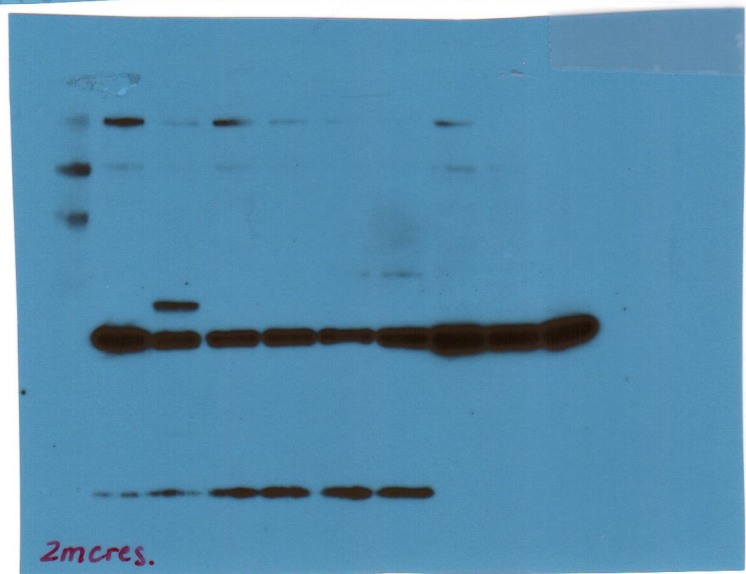

Supplement: Supplementary file 1 [file cancers-13-05130-s001.zip › cancers-1384109-supplementary/cancers-1384109-western blot/ER paper WBs/Western Scans - Lab Notebook 3 IN BINDER/WB.pdf]

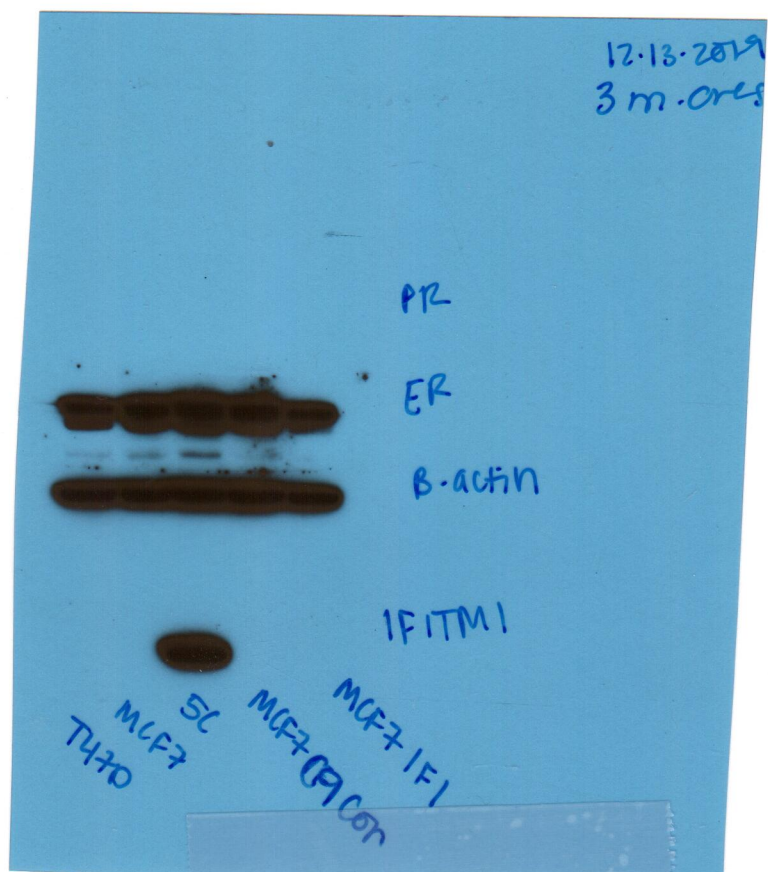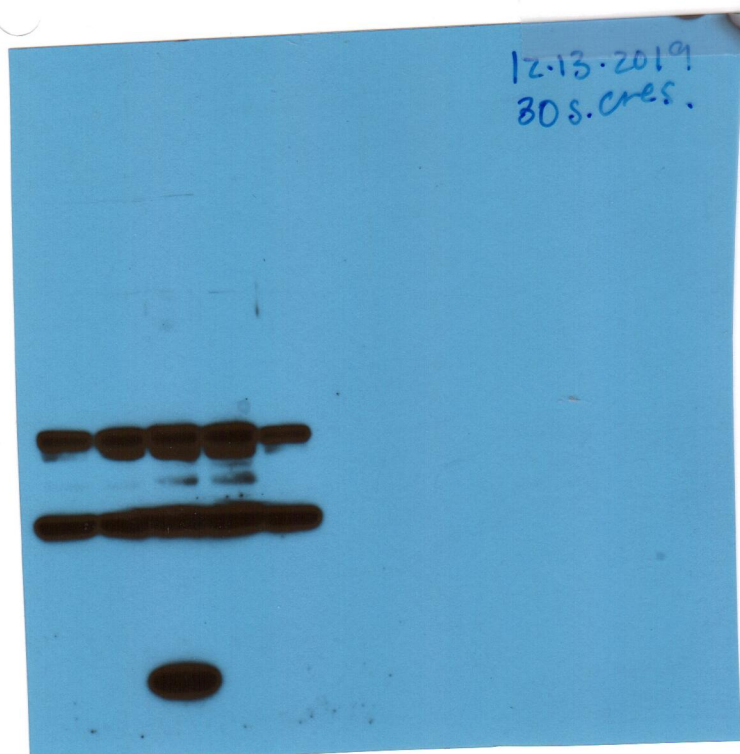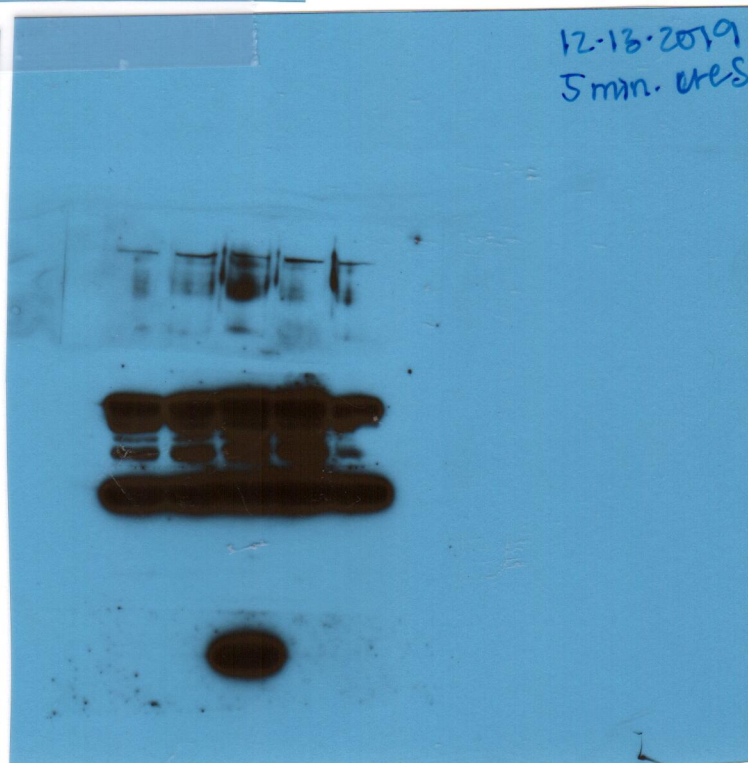

Supplement: Supplementary file 1 [file cancers-13-05130-s001.zip › cancers-1384109-supplementary/cancers-1384109-western blot/ER paper WBs/Western Scans - Lab Notebook 3 IN BINDER/WB0001.pdf]

1-15-2020

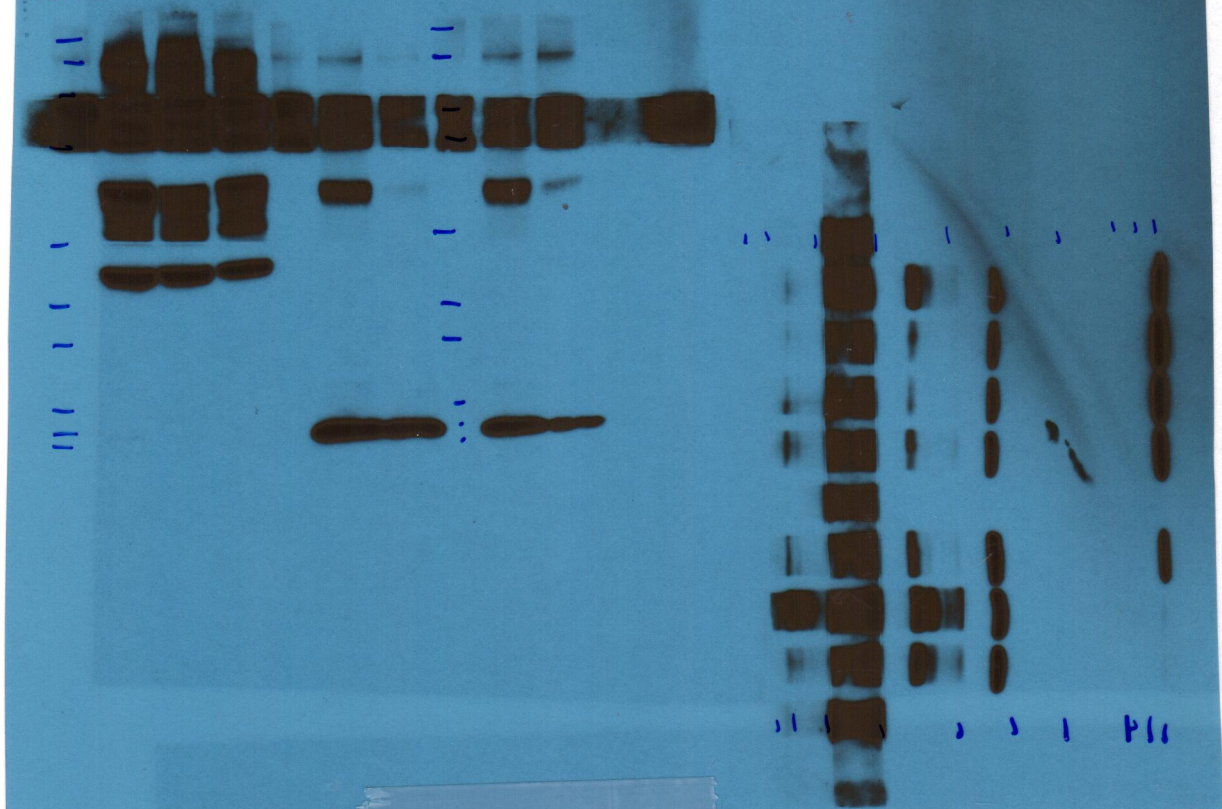

1-15-2020

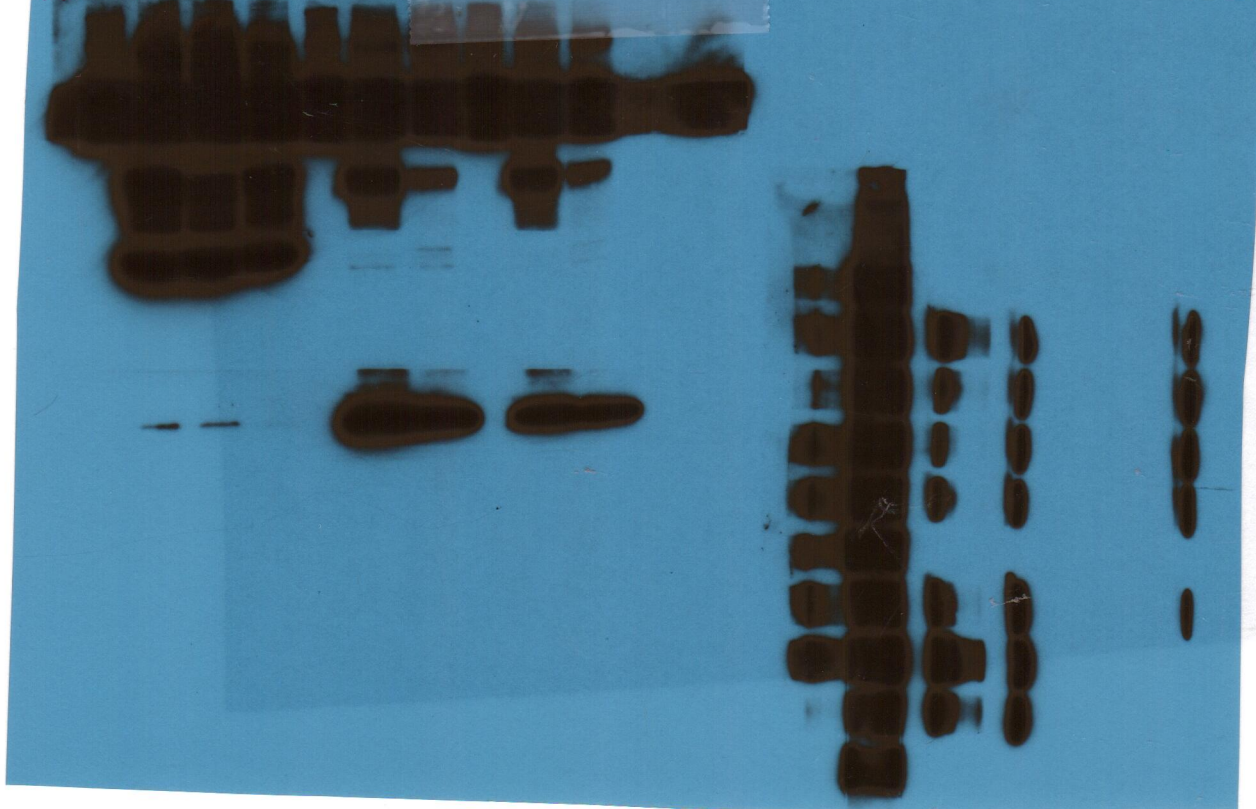

Supplement: Supplementary file 1 [file cancers-13-05130-s001.zip › cancers-1384109-supplementary/cancers-1384109-western blot/ER paper WBs/Western Scans - Lab Notebook 3 IN BINDER/WB0002.pdf]

0202-51-1

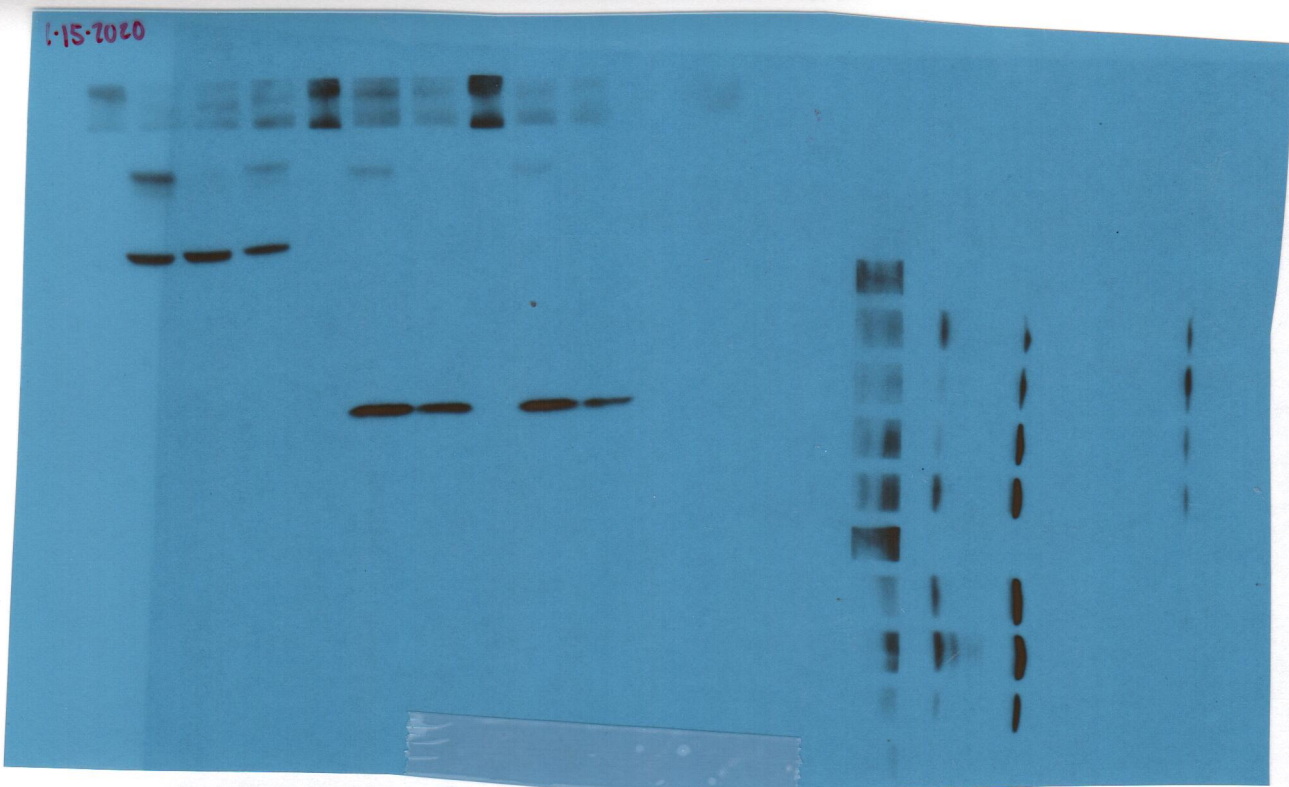

1-15-2026

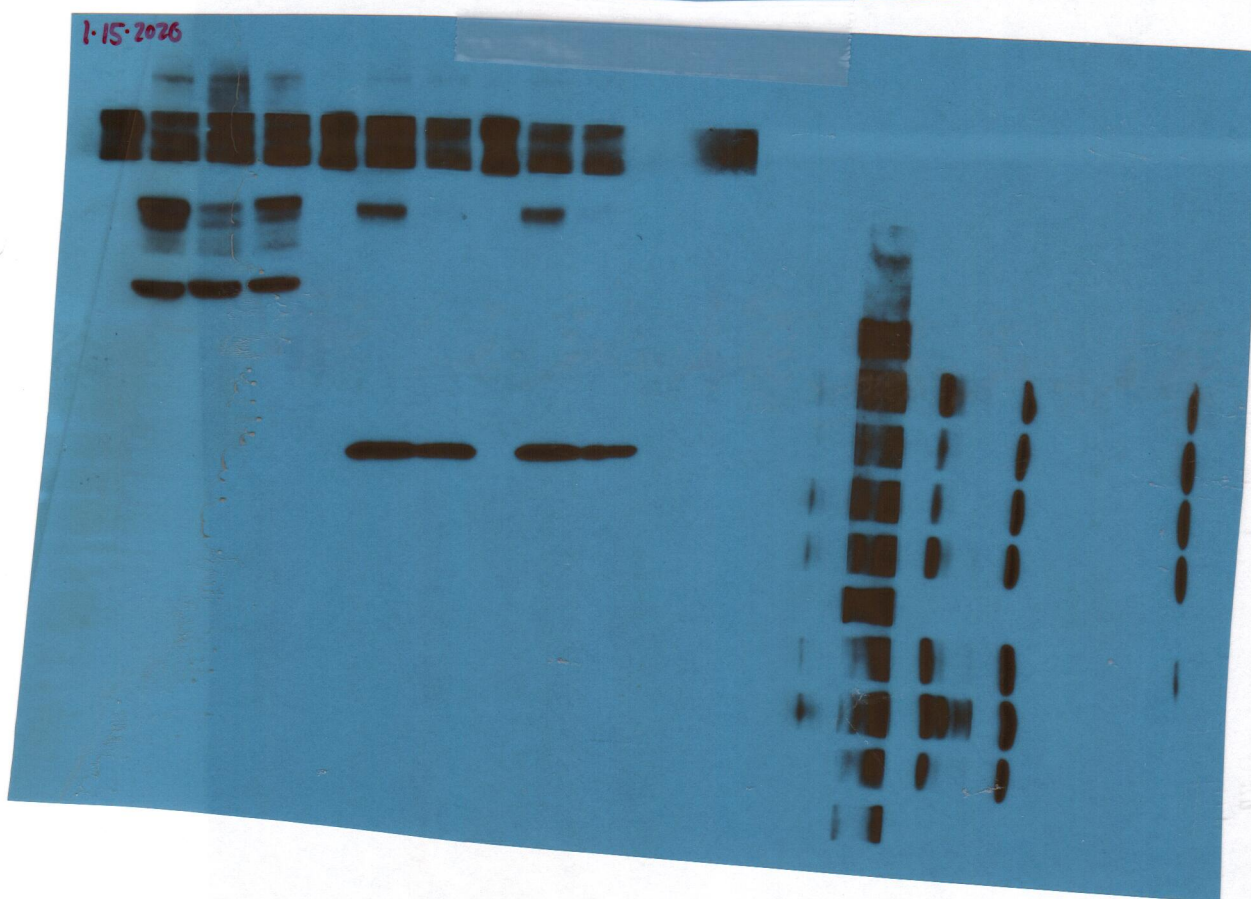

Supplement: Supplementary file 1 [file cancers-13-05130-s001.zip › cancers-1384109-supplementary/cancers-1384109-western blot/ER paper WBs/Western Scans - Lab Notebook 3 IN BINDER/WB0003.pdf]

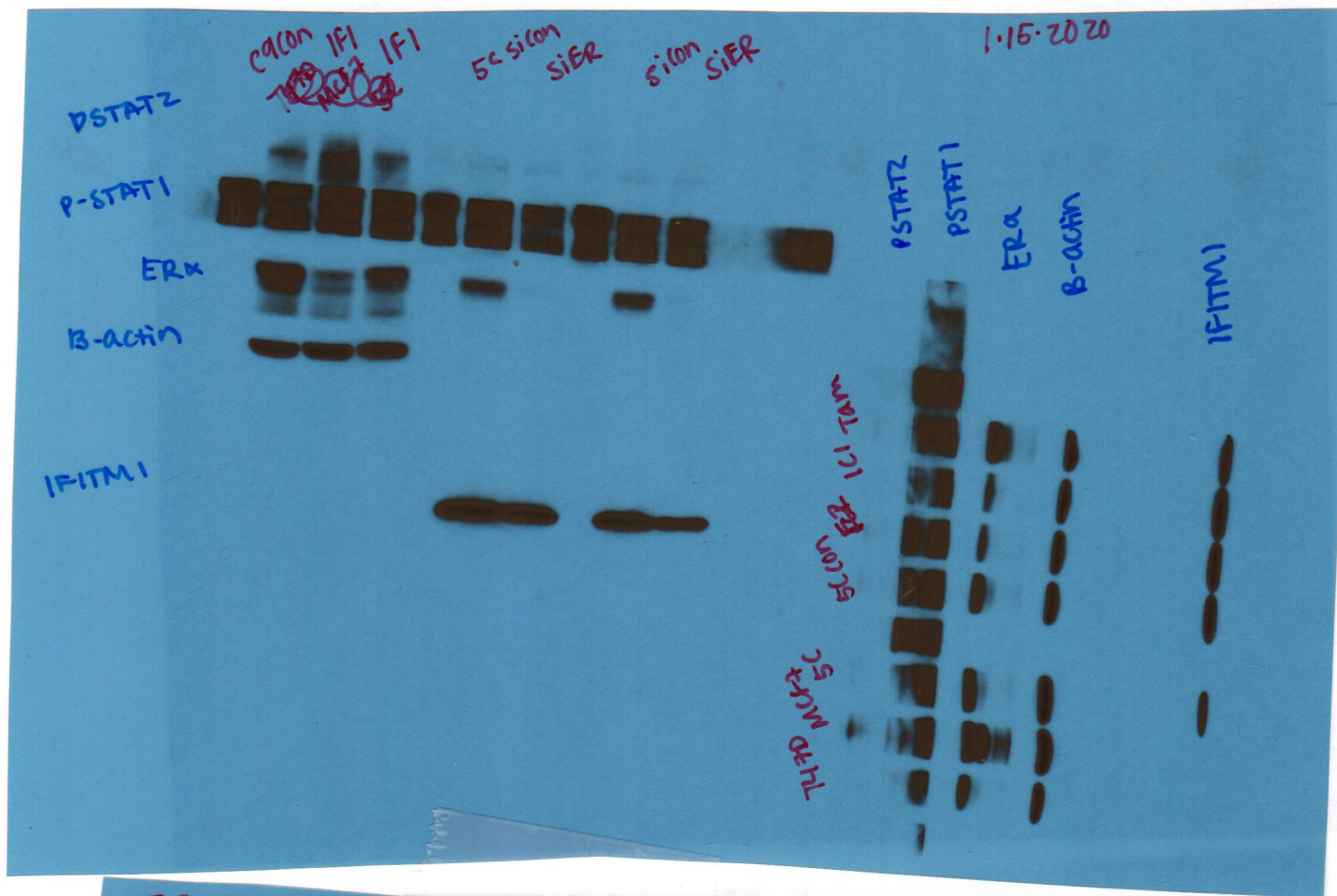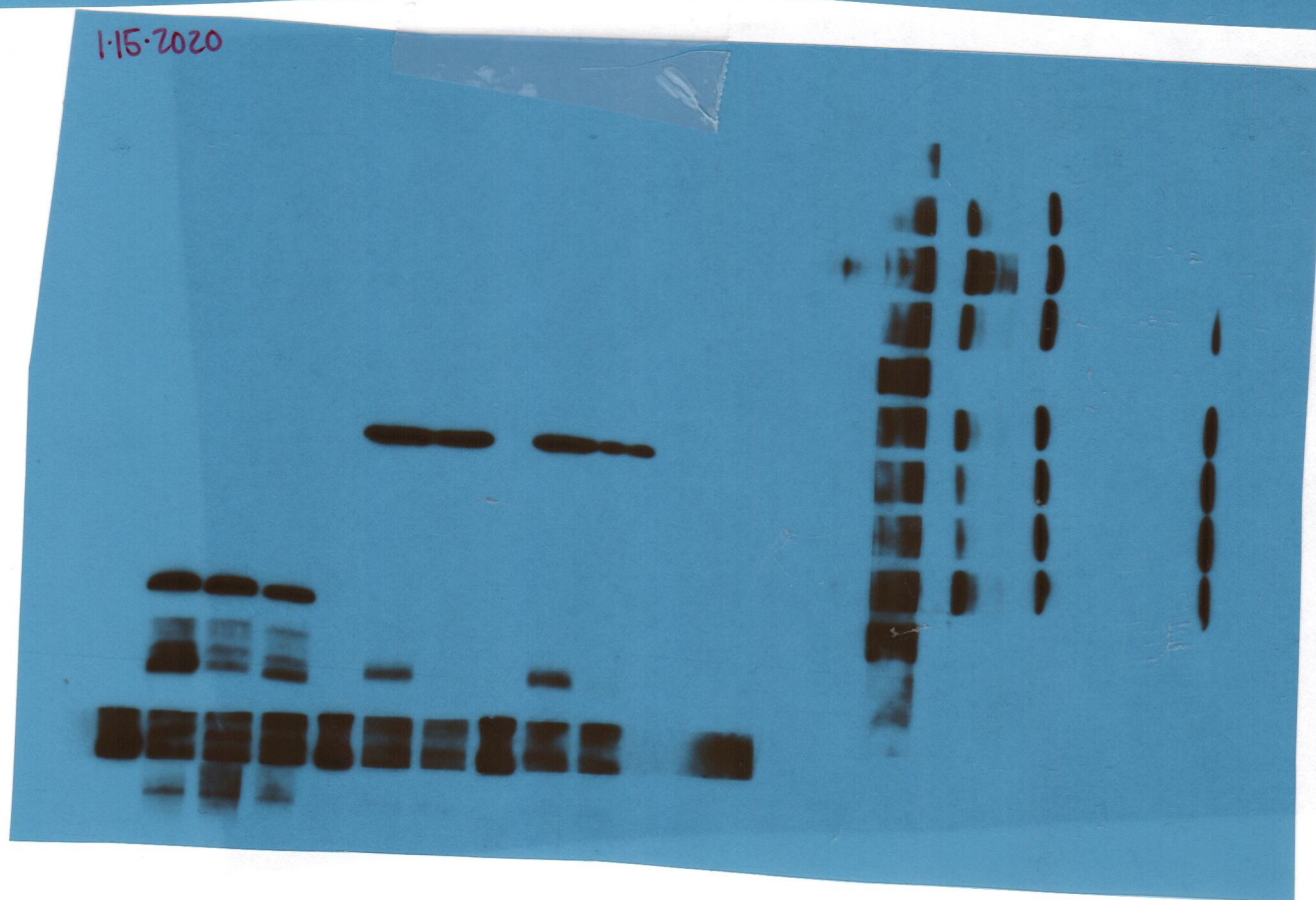

Supplement: Supplementary file 1 [file cancers-13-05130-s001.zip › cancers-1384109-supplementary/cancers-1384109-western blot/ER paper WBs/Western Scans - Lab Notebook 3 IN BINDER/WB0004.pdf]

1-15-2020

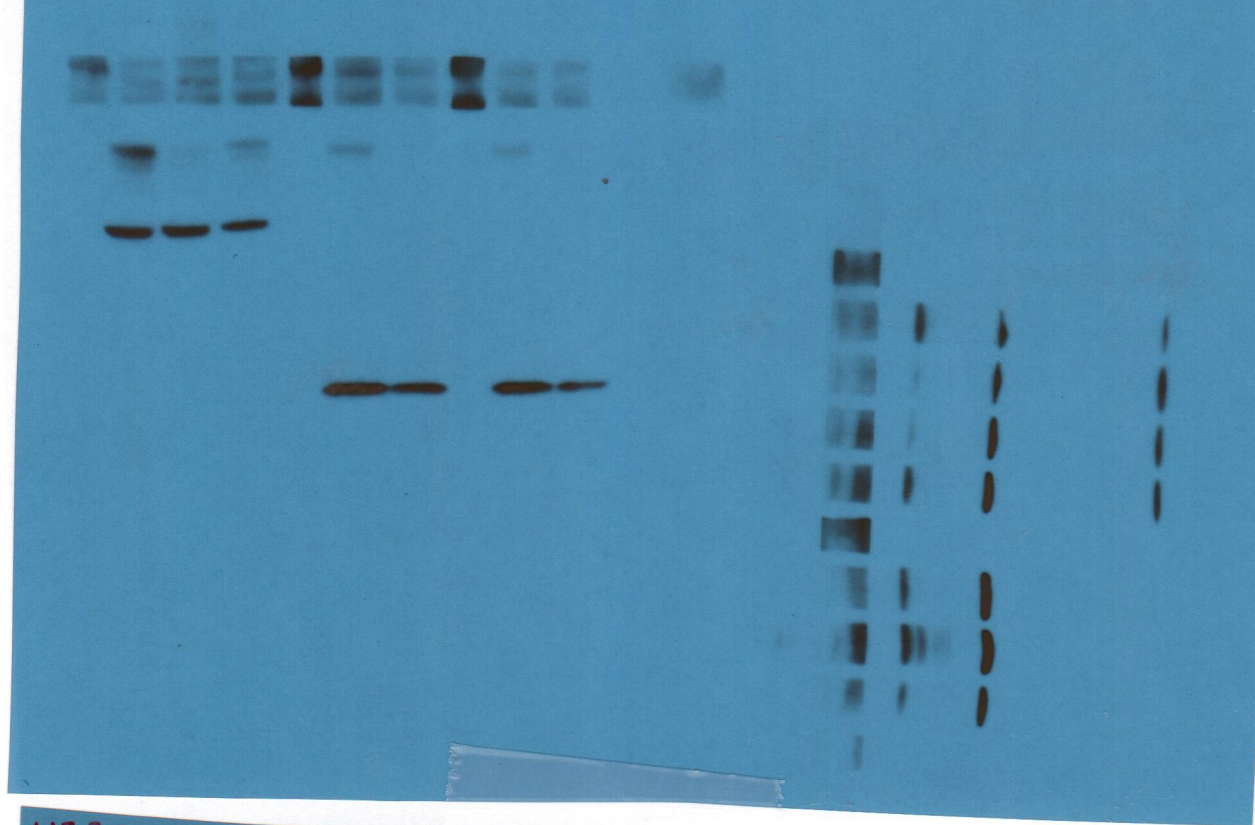

1-15-2020

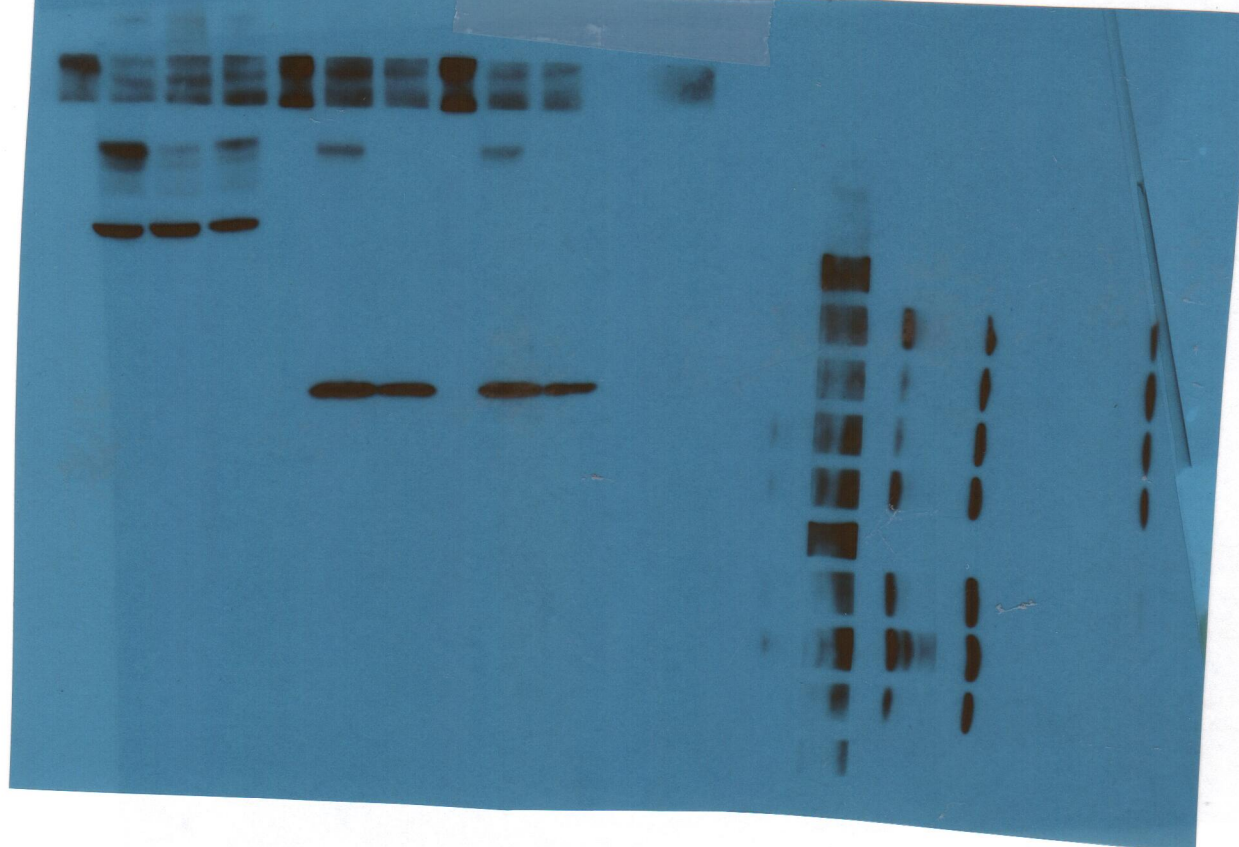

Supplement: Supplementary file 1 [file cancers-13-05130-s001.zip › cancers-1384109-supplementary/cancers-1384109-western blot/ER paper WBs/Western Scans - Lab Notebook 3 IN BINDER/WB0005.pdf]

1-16-2020

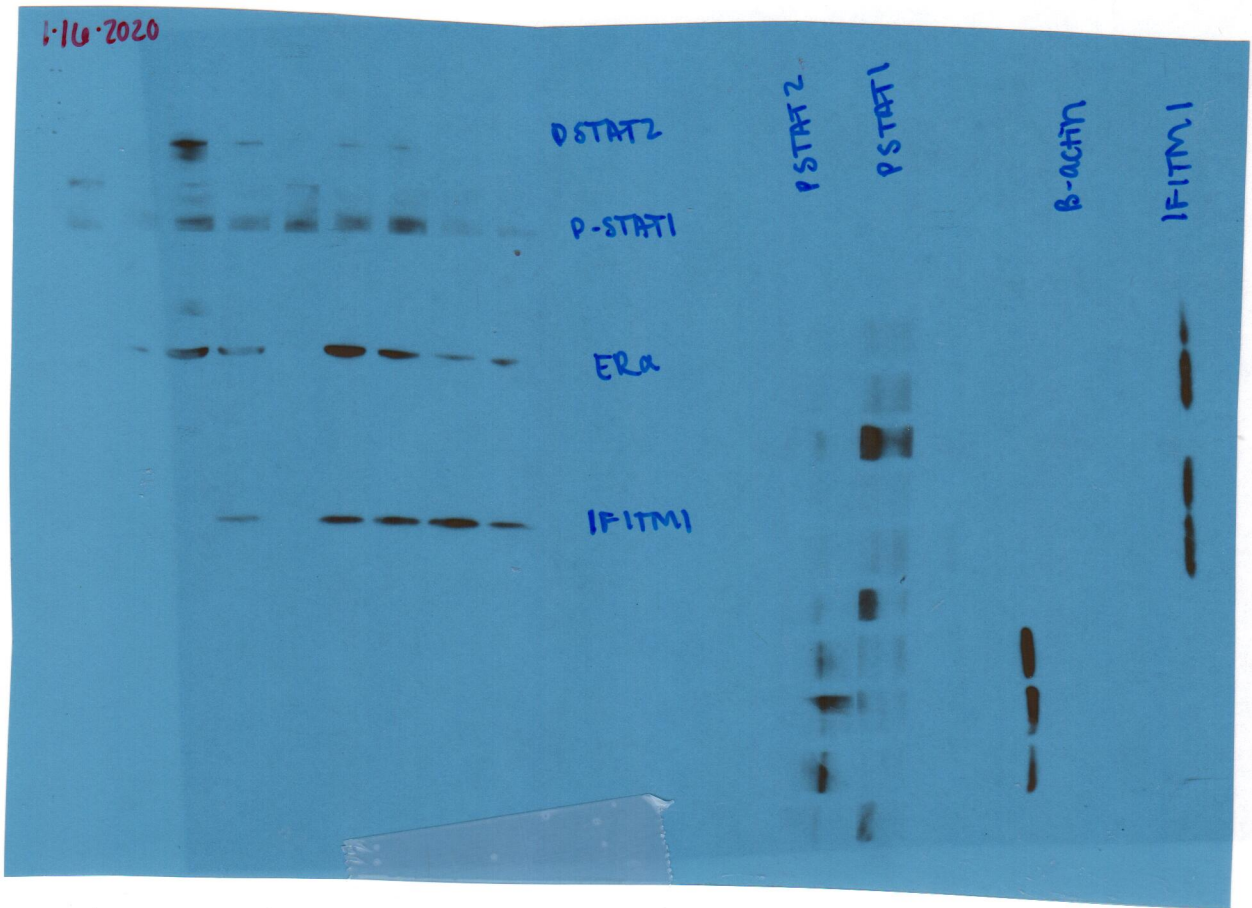

0202-01-1

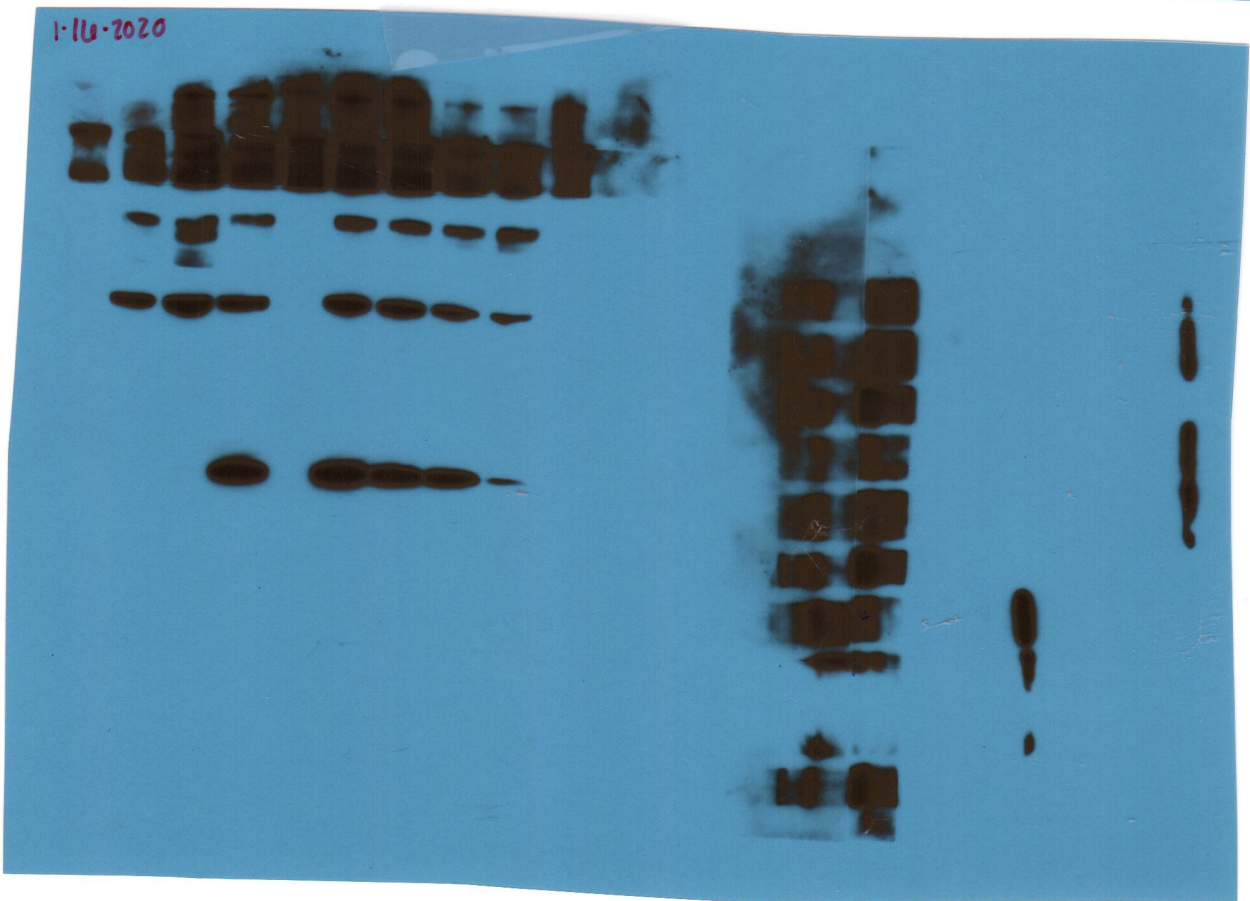

Supplement: Supplementary file 1 [file cancers-13-05130-s001.zip › cancers-1384109-supplementary/cancers-1384109-western blot/ER paper WBs/Western Scans - Lab Notebook 3 IN BINDER/WB0006.pdf]

1.16.2020

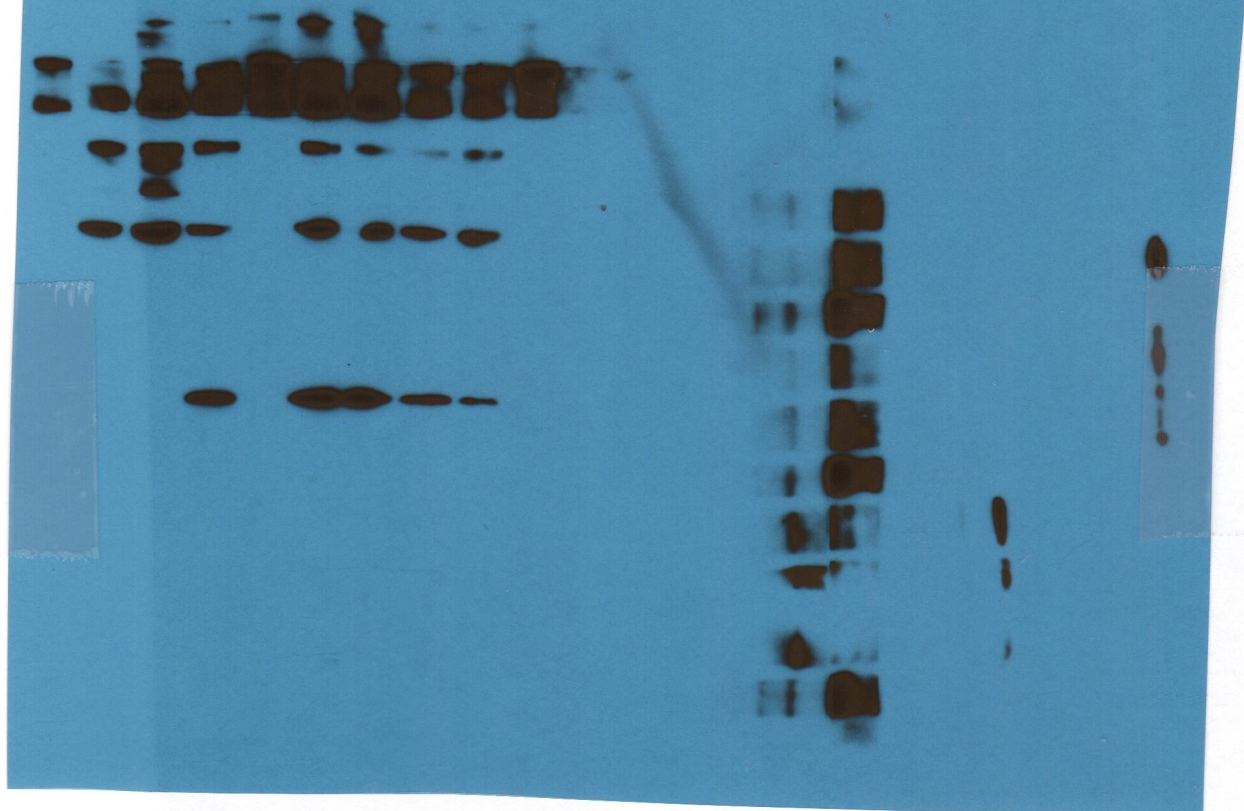

Supplement: Supplementary file 1 [file cancers-13-05130-s001.zip › cancers-1384109-supplementary/cancers-1384109-western blot/ER paper WBs/Western Scans - Lab Notebook 3 IN BINDER/WB0007.pdf]

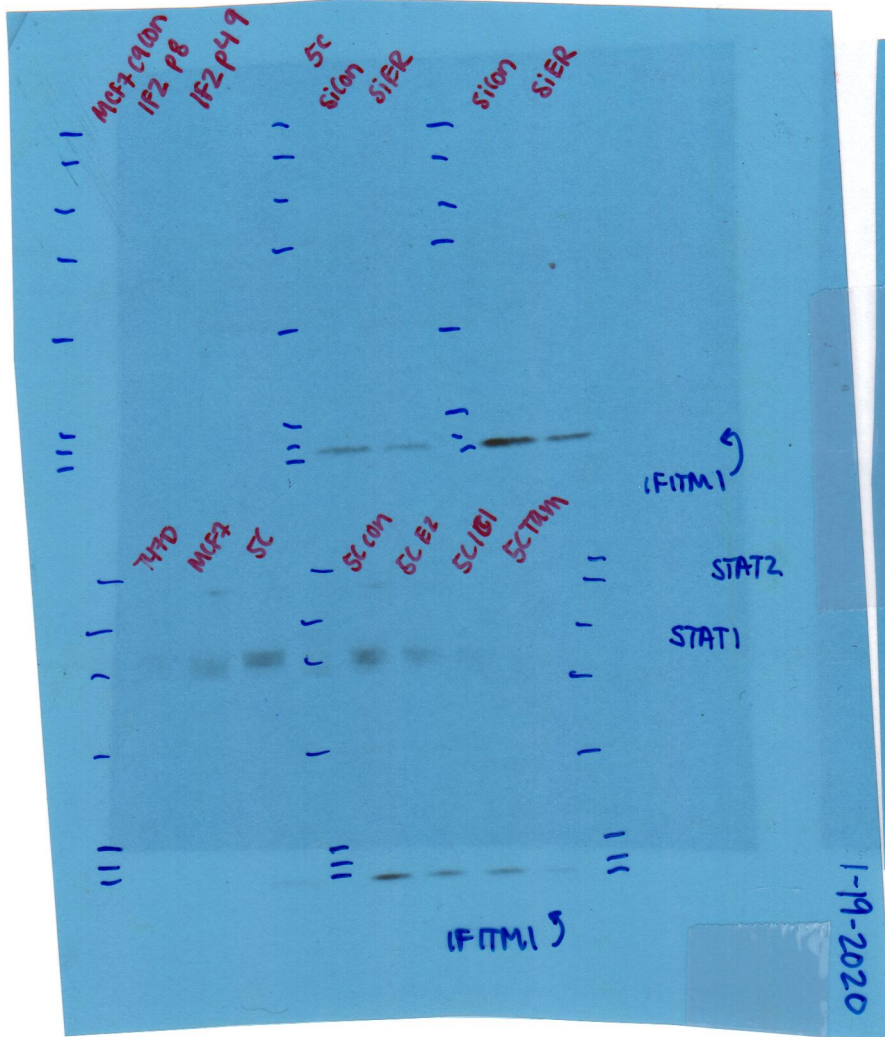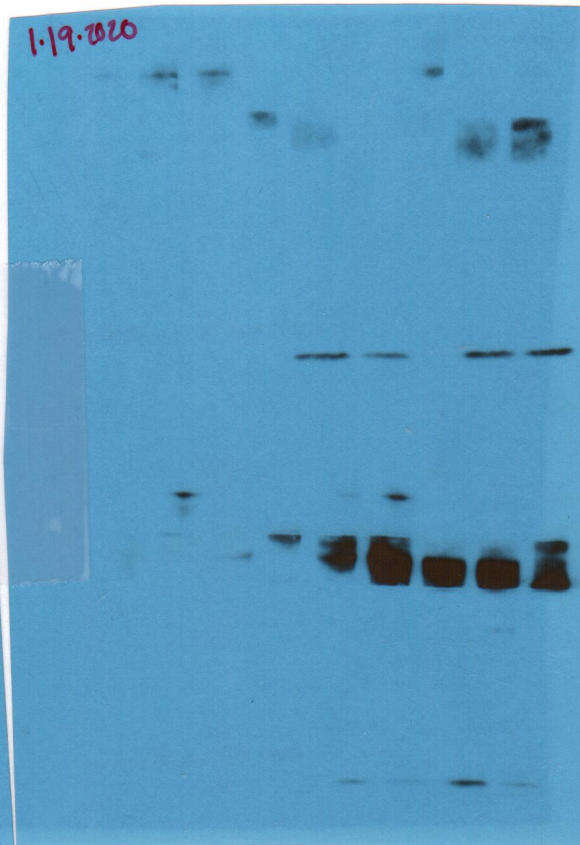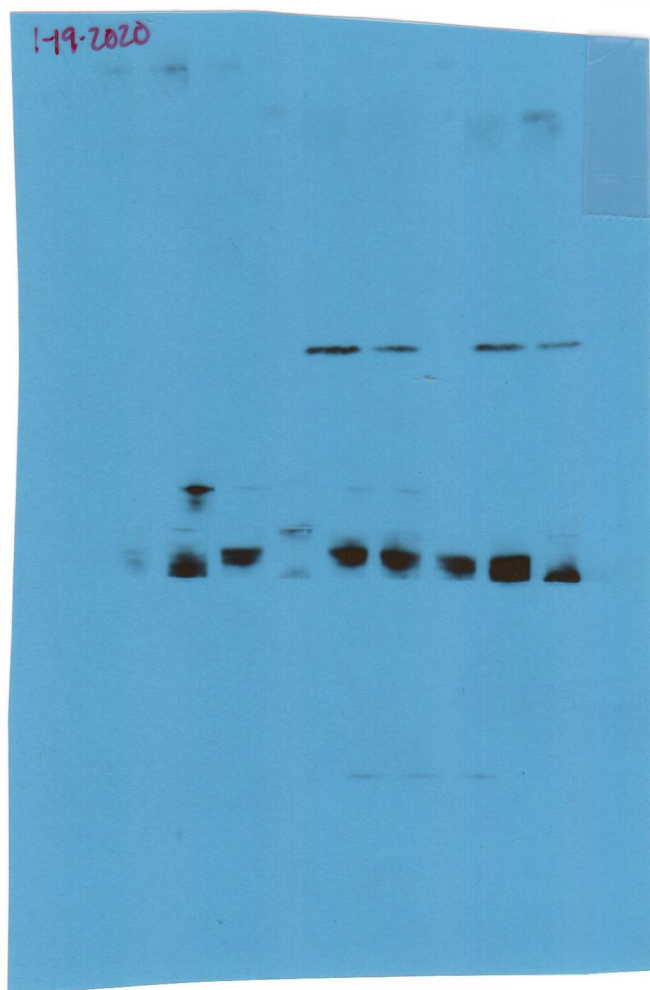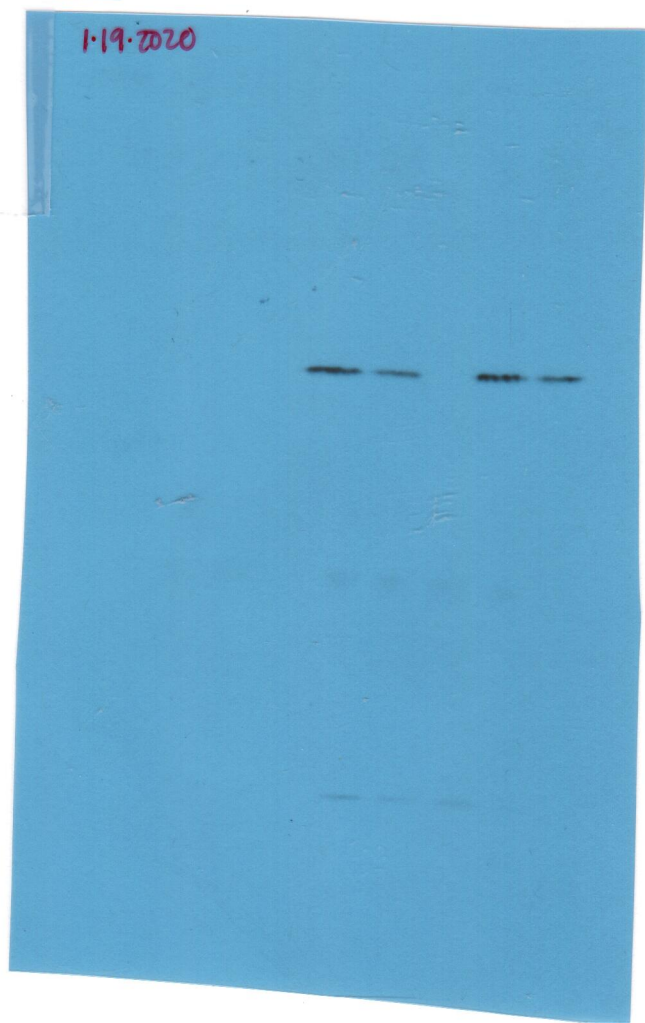

Supplement: Supplementary file 1 [file cancers-13-05130-s001.zip › cancers-1384109-supplementary/cancers-1384109-western blot/ER paper WBs/Western Scans - Lab Notebook 3 IN BINDER/WB0008.pdf]

0202.611

0202.611

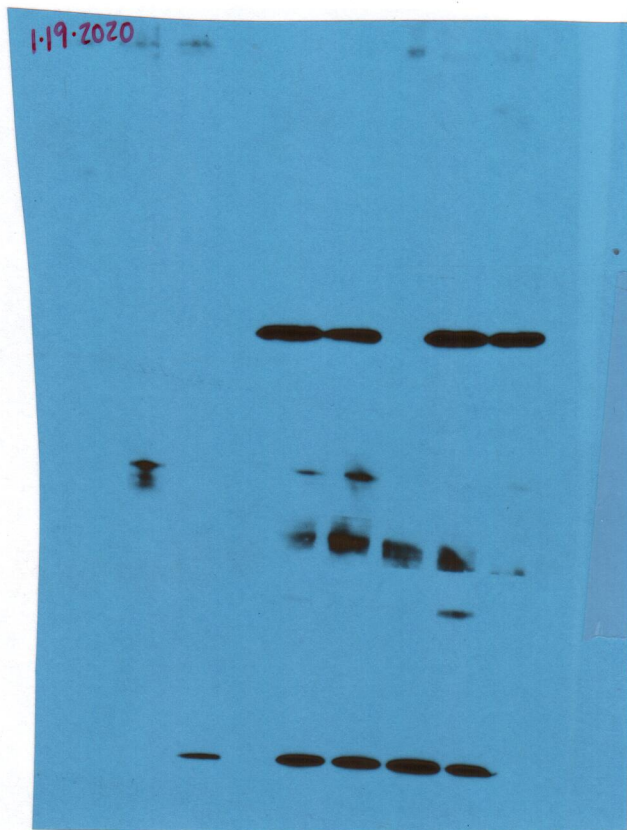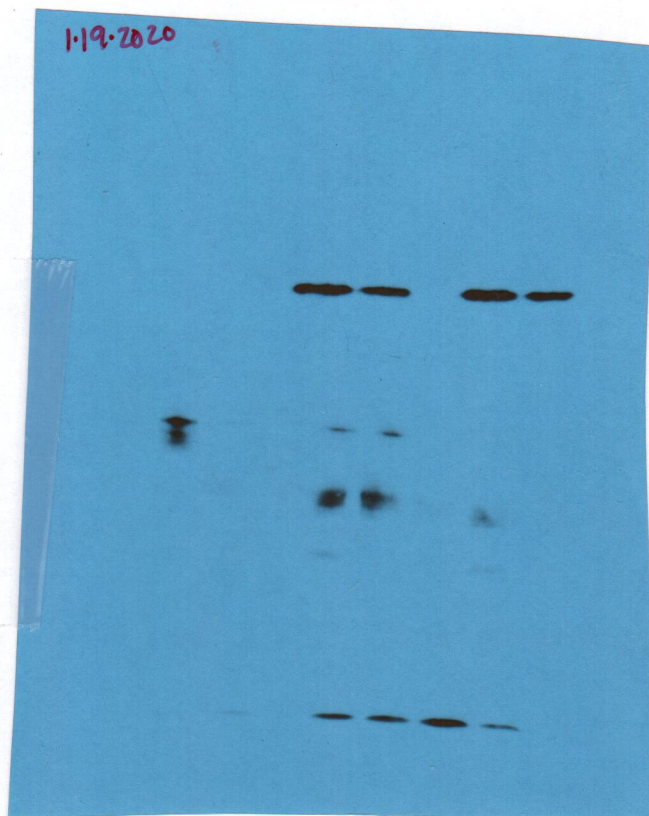

Supplement: Supplementary file 1 [file cancers-13-05130-s001.zip › cancers-1384109-supplementary/cancers-1384109-western blot/ER paper WBs/Western Scans - Lab Notebook 3 IN BINDER/WB0009.pdf]

1-31-2020  
30S ECL

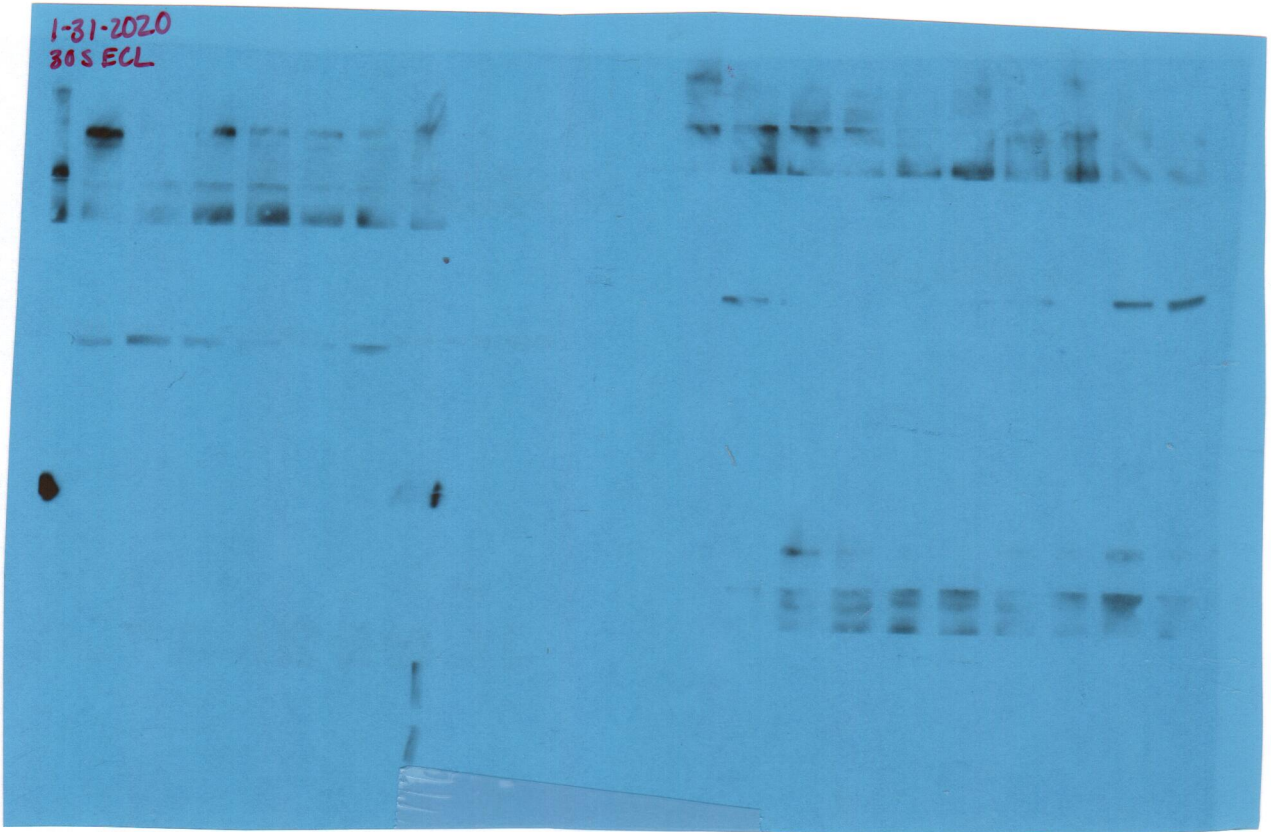

1-31-2020

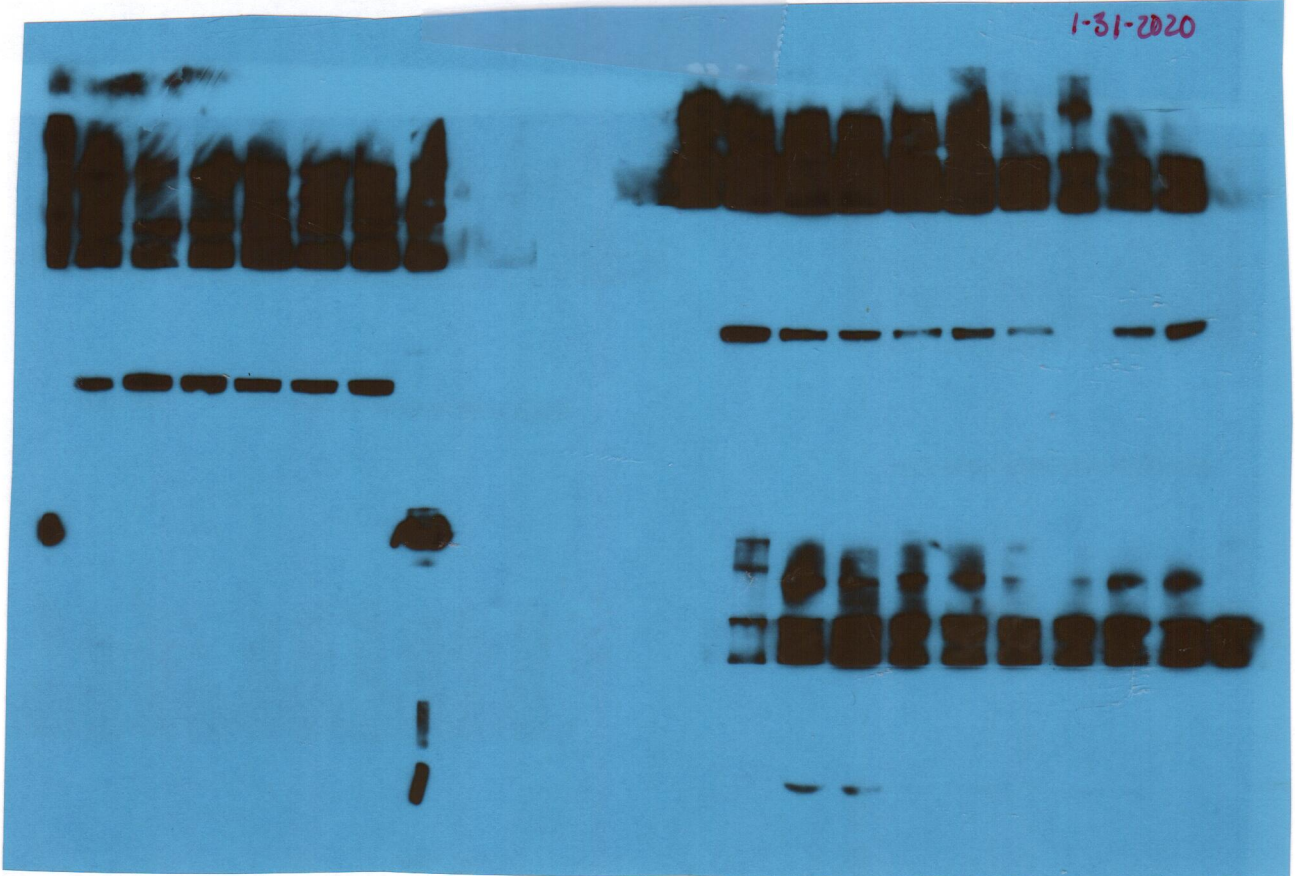

Supplement: Supplementary file 1 [file cancers-13-05130-s001.zip › cancers-1384109-supplementary/cancers-1384109-western blot/ER paper WBs/Western Scans - Lab Notebook 3 IN BINDER/WB0010.pdf]

1-31-2020

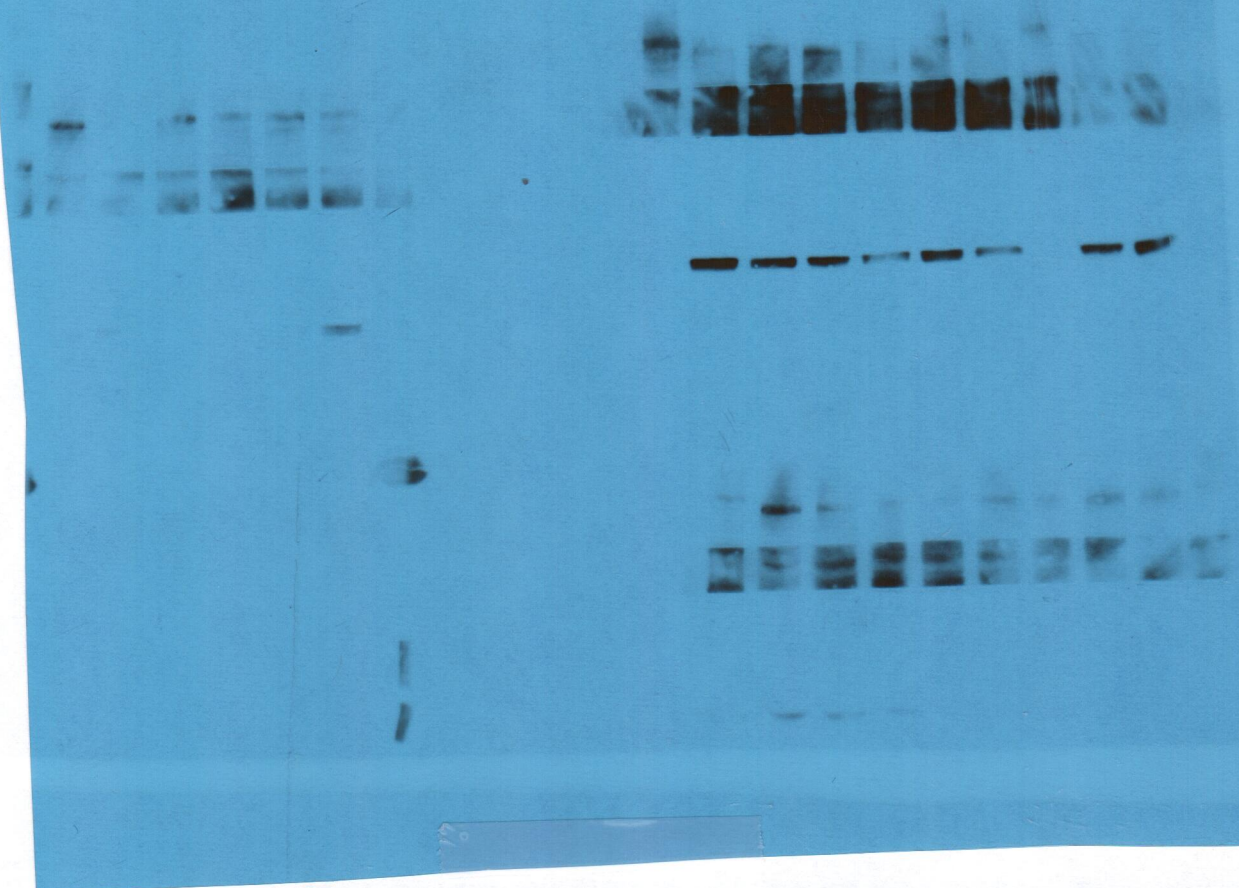

1-31-2020

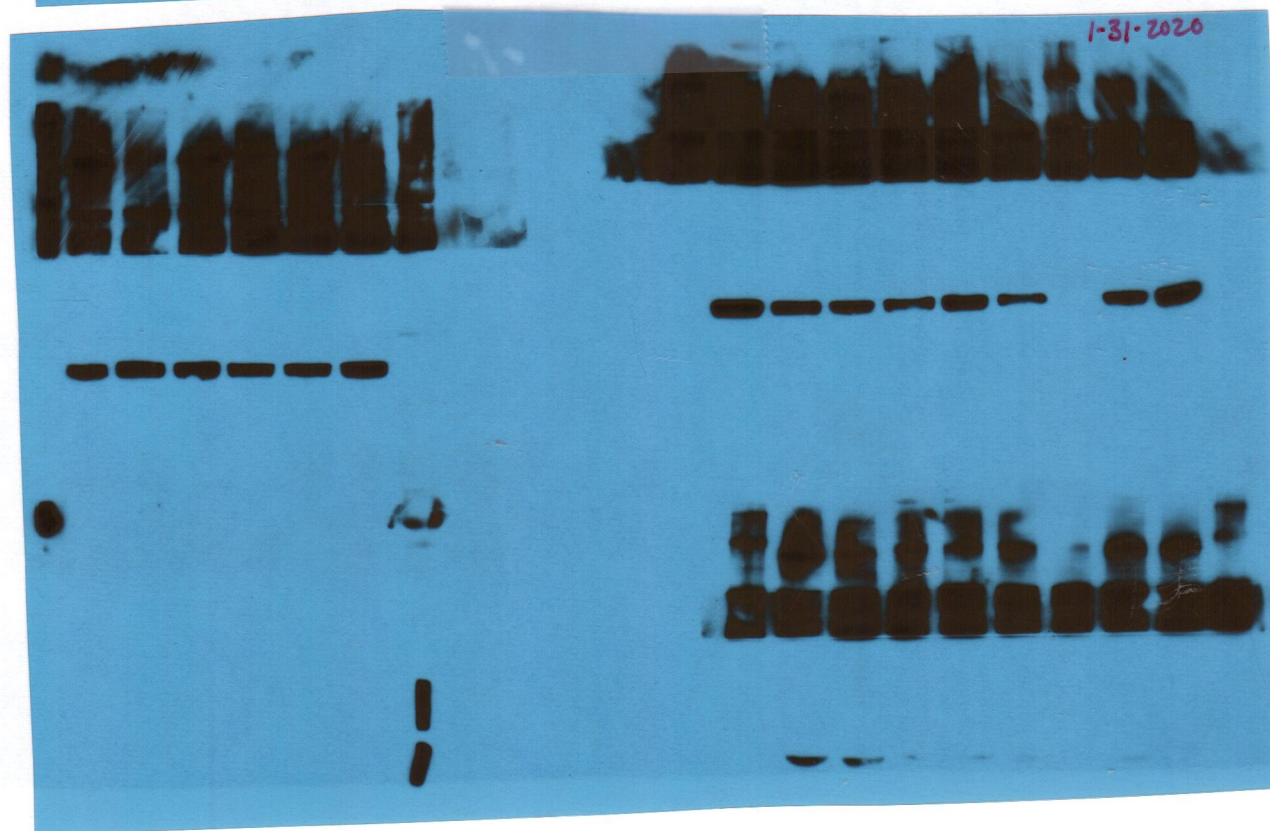

Supplement: Supplementary file 1 [file cancers-13-05130-s001.zip › cancers-1384109-supplementary/cancers-1384109-western blot/ER paper WBs/Western Scans - Lab Notebook 3 IN BINDER/WB0011.pdf]

2-1-2020

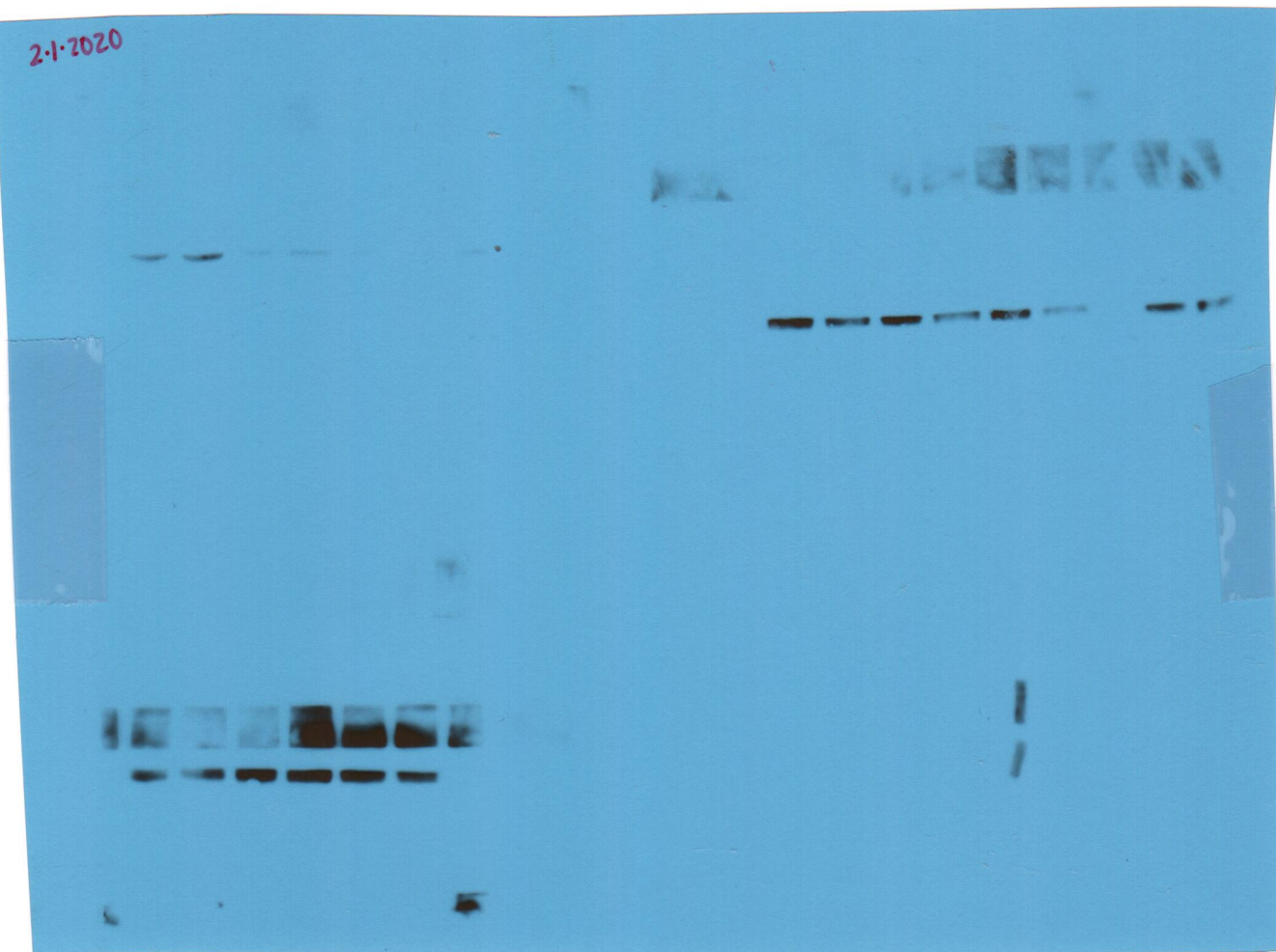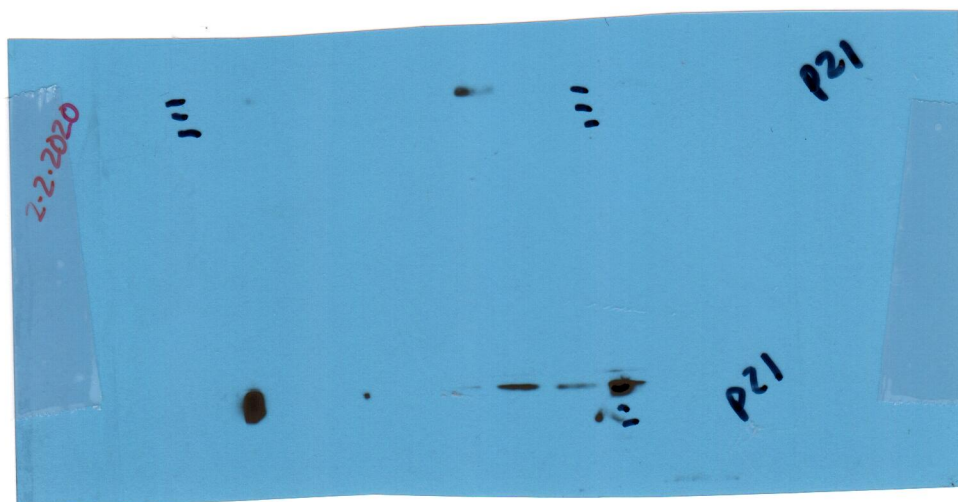

Supplement: Supplementary file 1 [file cancers-13-05130-s001.zip › cancers-1384109-supplementary/cancers-1384109-western blot/ER paper WBs/Western Scans - Lab Notebook 3 IN BINDER/WB0012.pdf]

2-3-2020

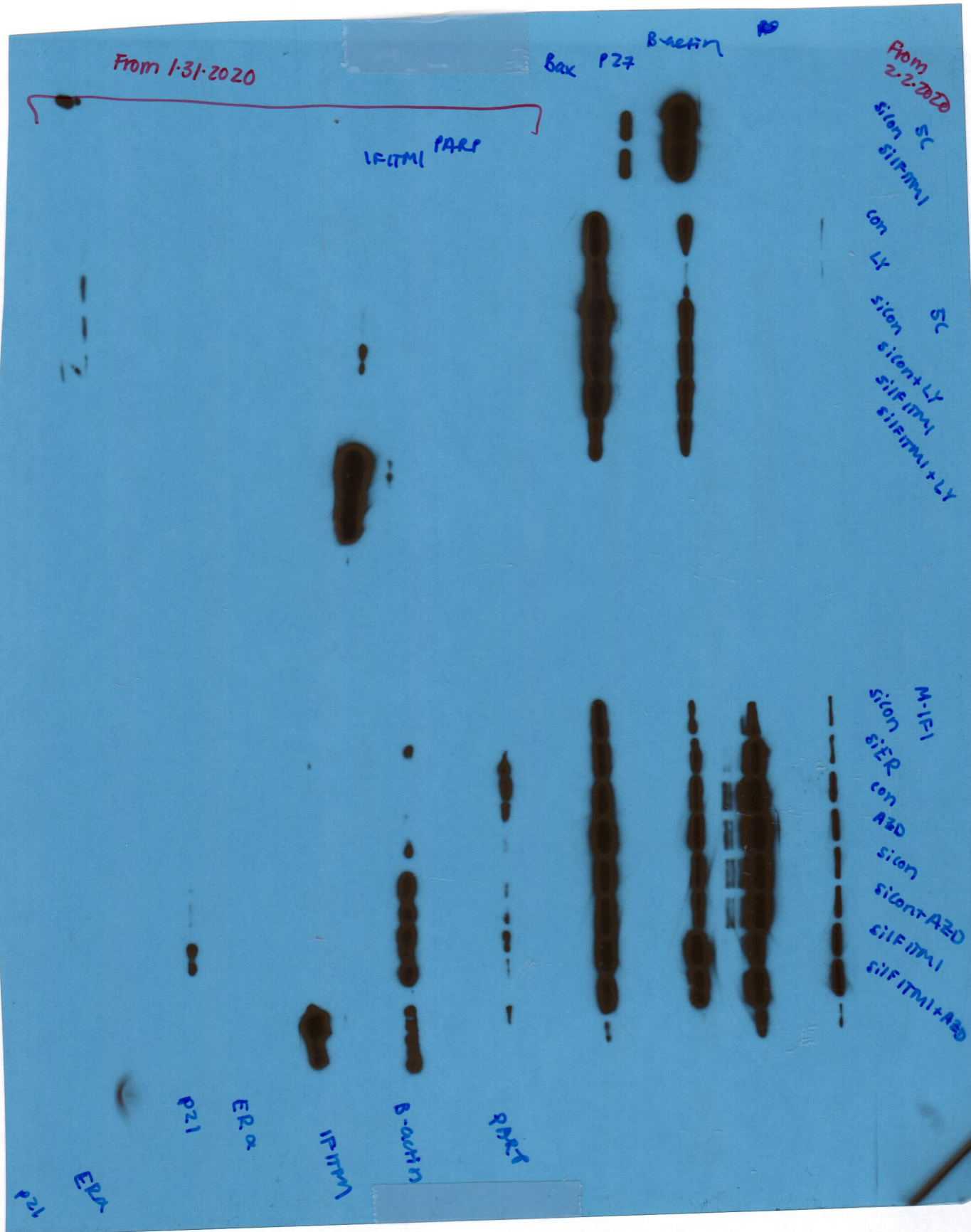

Supplement: Supplementary file 1 [file cancers-13-05130-s001.zip › cancers-1384109-supplementary/cancers-1384109-western blot/ER paper WBs/Western Scans - Lab Notebook 3 IN BINDER/WB0015.pdf]

2-3-2020

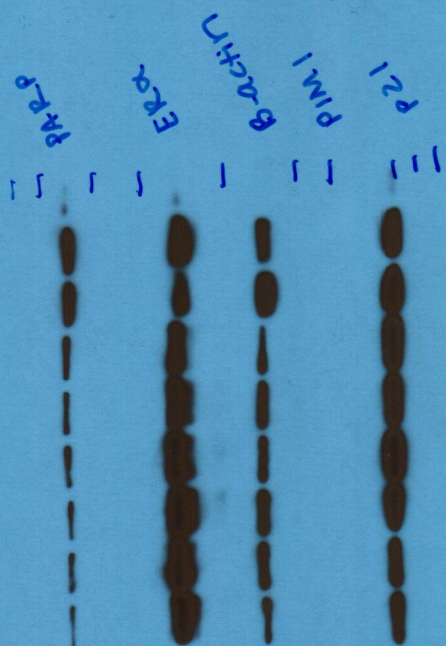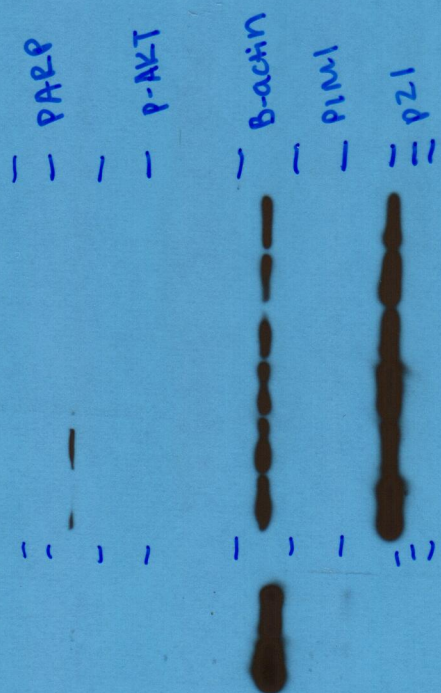

1st  
ERA  
p21

1st  
ERA  
p21

AKT

ERA

p21

Supplement: Supplementary file 1 [file cancers-13-05130-s001.zip › cancers-1384109-supplementary/cancers-1384109-western blot/ER paper WBs/Western Scans - Lab Notebook 3 IN BINDER/WB0016.pdf]

2-3-2020

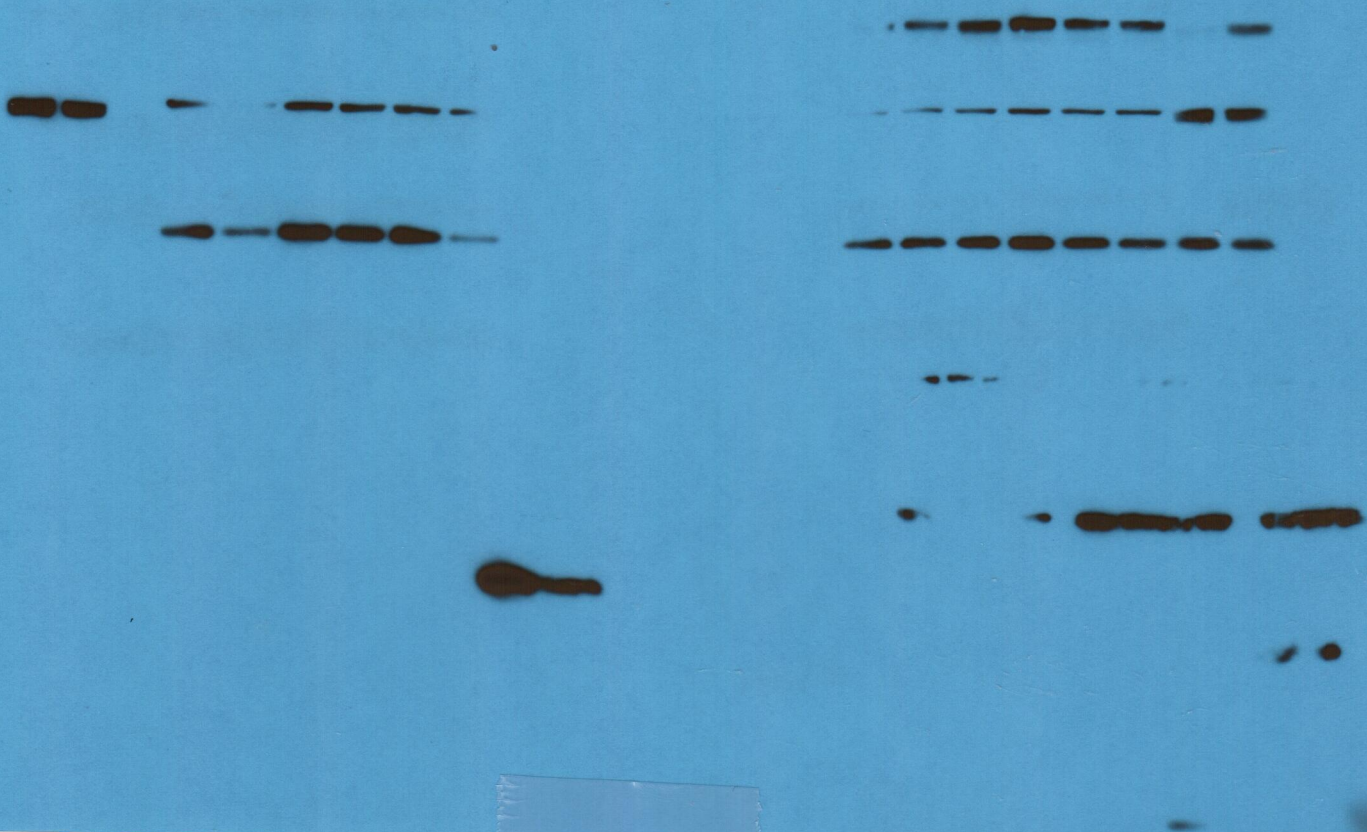

2-3-2020

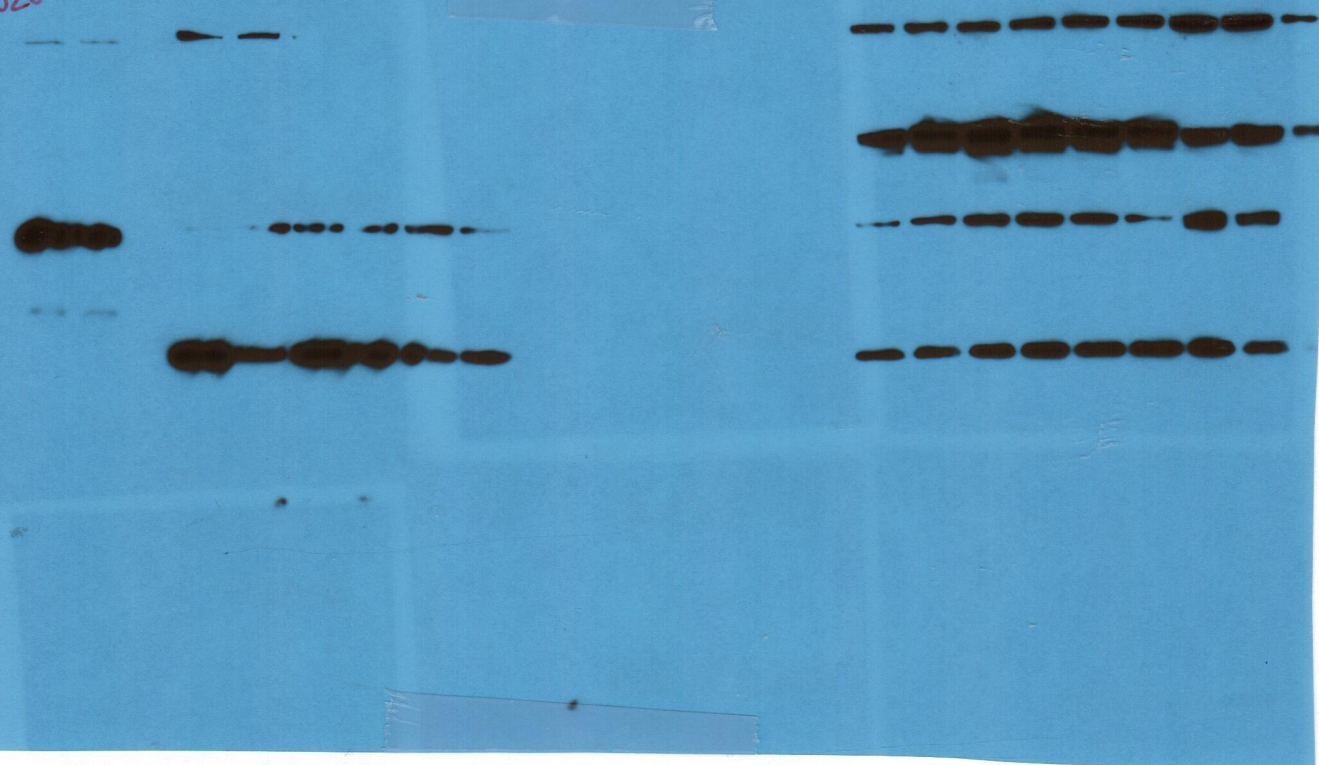

Supplement: Supplementary file 1 [file cancers-13-05130-s001.zip › cancers-1384109-supplementary/cancers-1384109-western blot/ER paper WBs/Western Scans - Lab Notebook 3 IN BINDER/WB0017.pdf]

2-3-2020

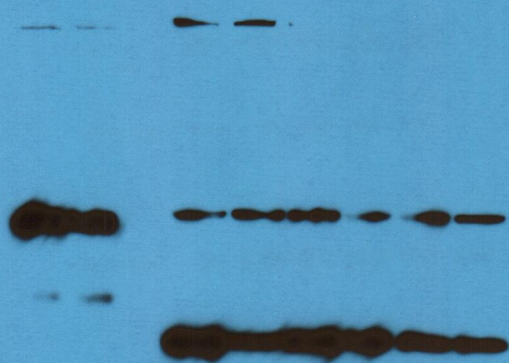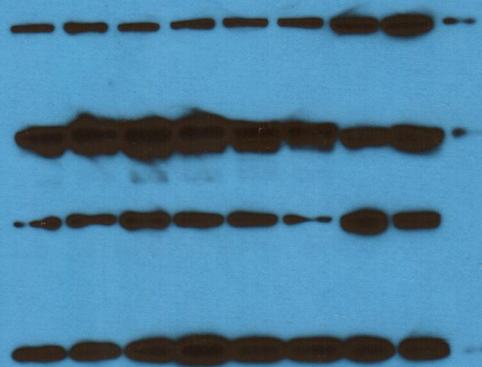

band

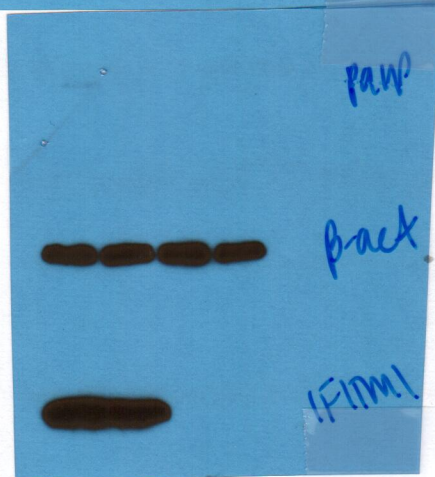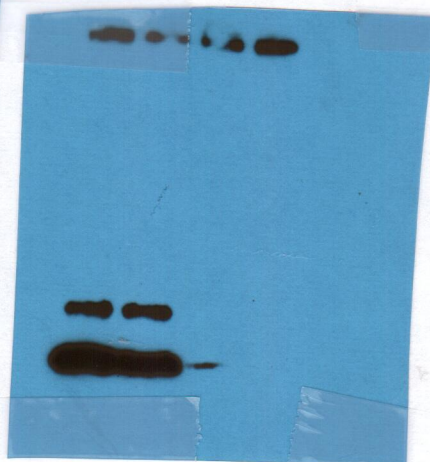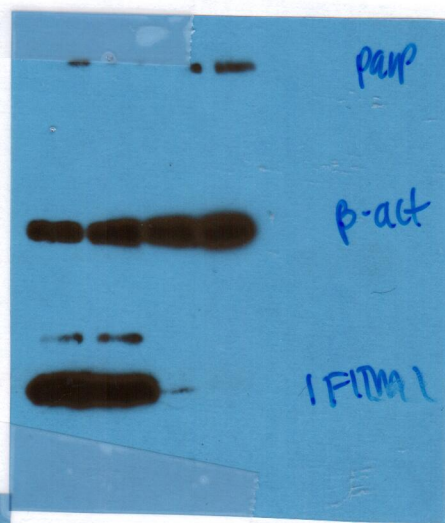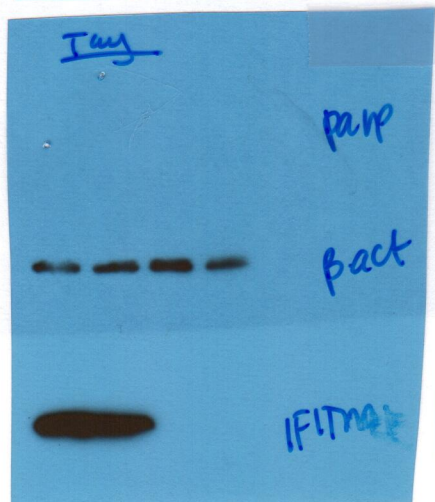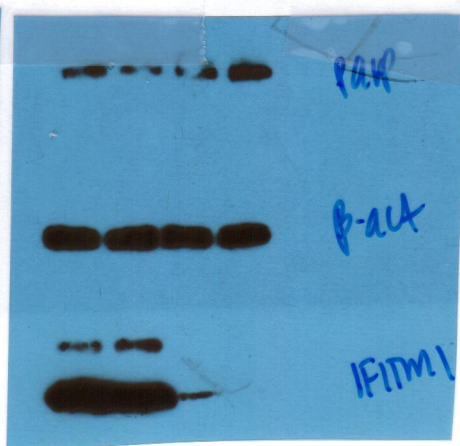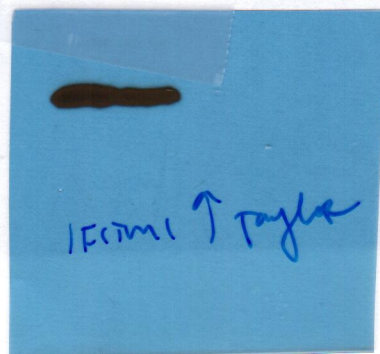

Supplement: Supplementary file 1 [file cancers-13-05130-s001.zip › cancers-1384109-supplementary/cancers-1384109-western blot/ER paper WBs/Western Scans - Lab Notebook 3 IN BINDER/WB0018.pdf]

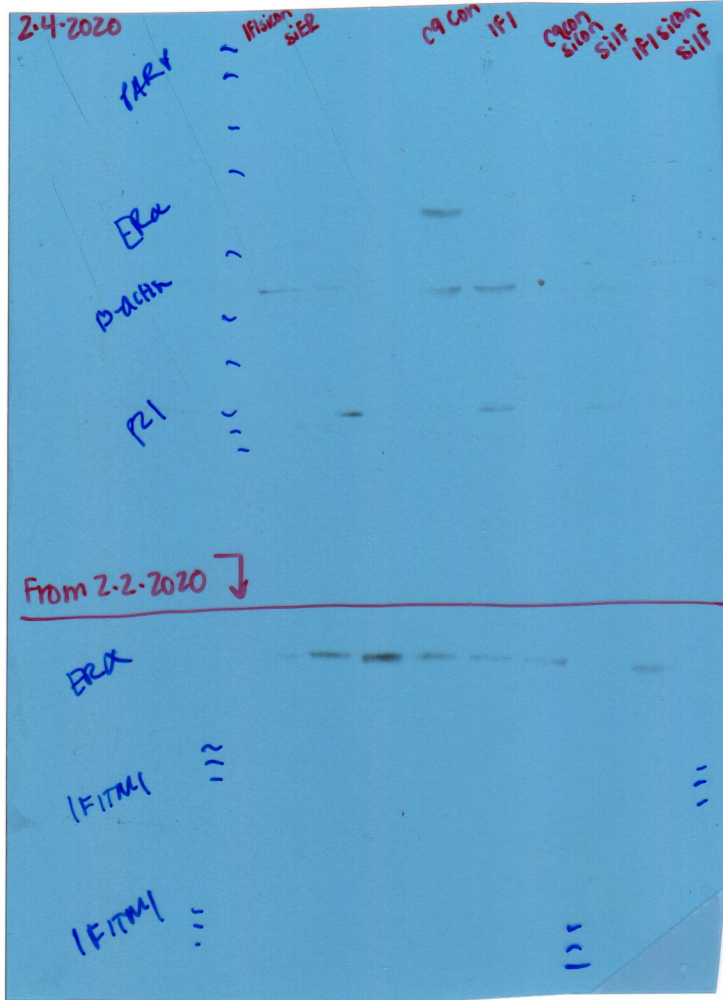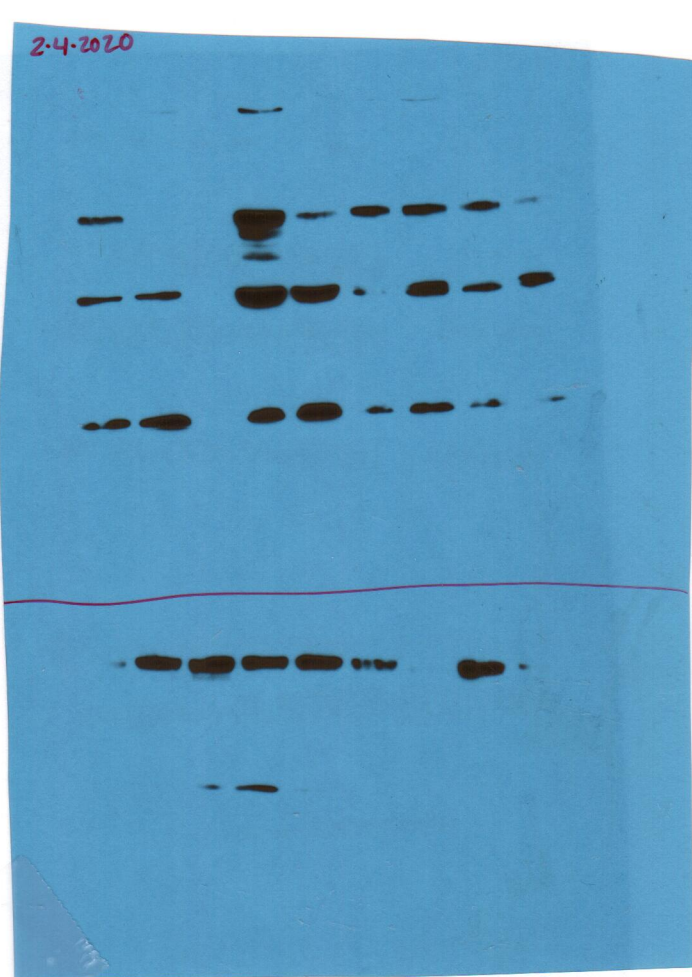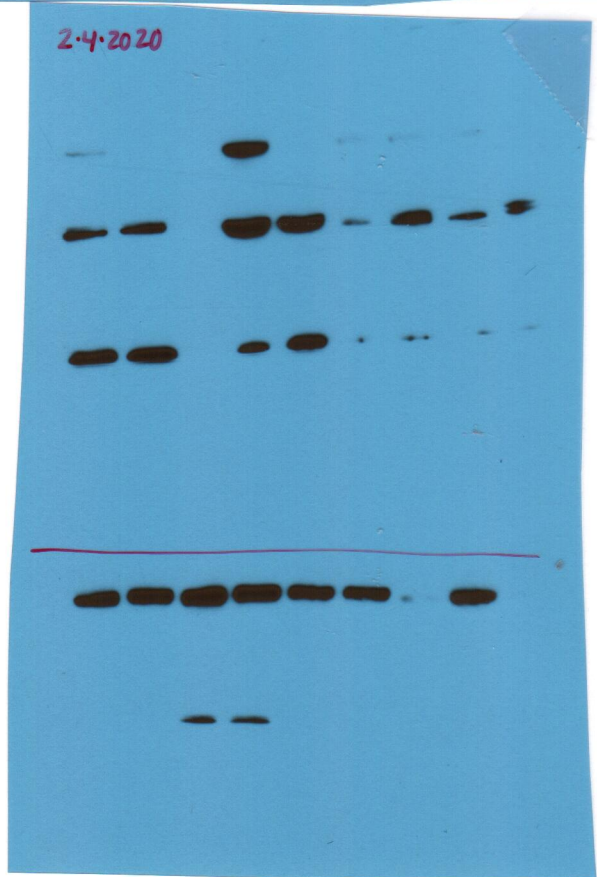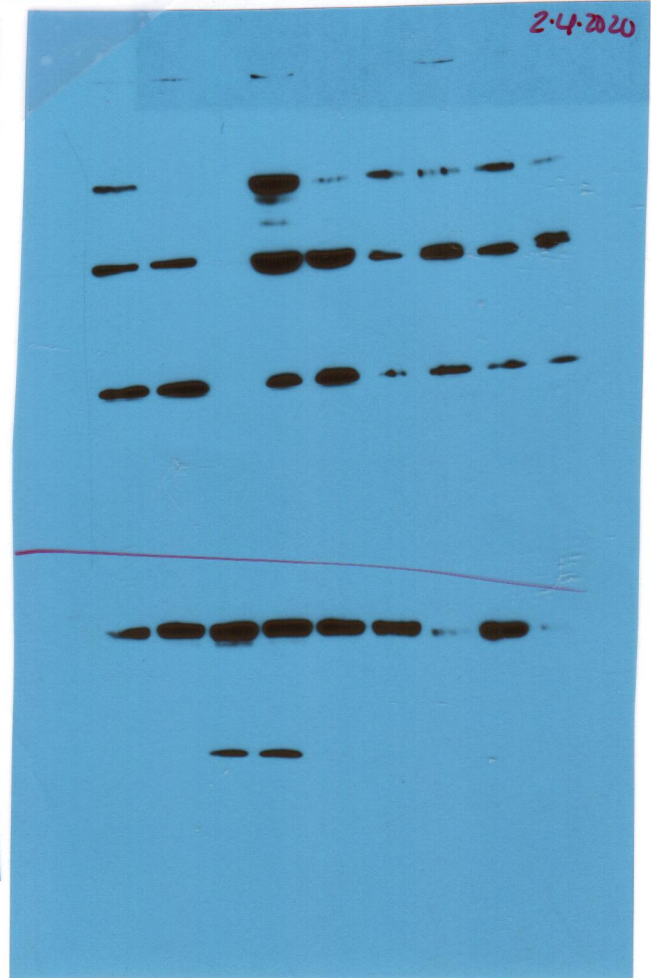

Supplement: Supplementary file 1 [file cancers-13-05130-s001.zip › cancers-1384109-supplementary/cancers-1384109-western blot/ER paper WBs/Western Scans - Lab Notebook 3 IN BINDER/WB0019.pdf]

2-4-2020

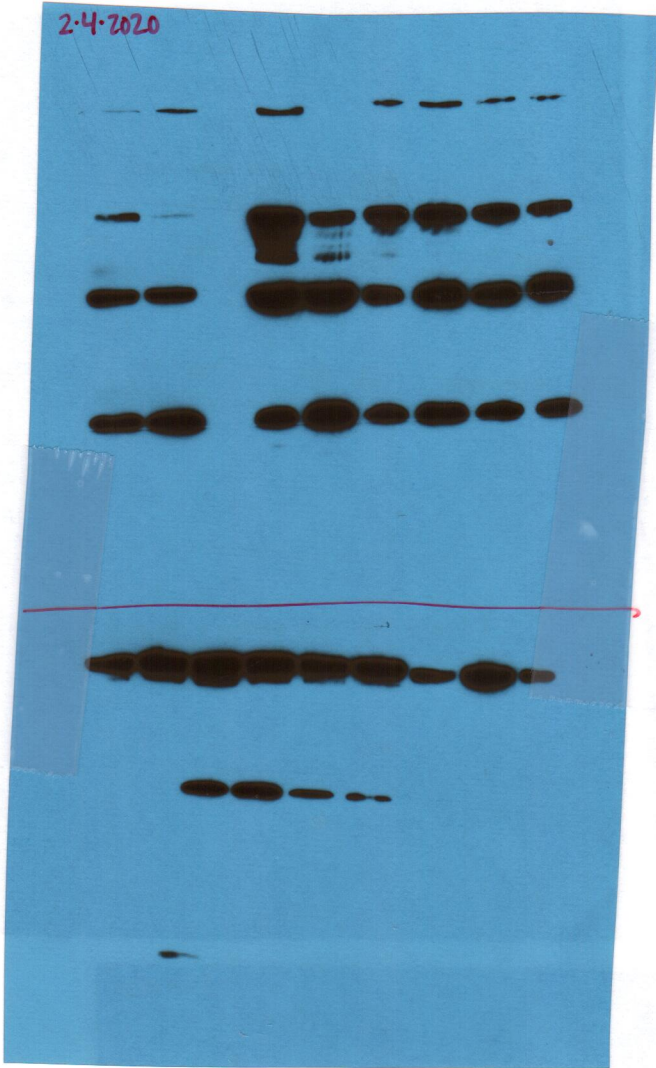

Supplement: Supplementary file 1 [file cancers-13-05130-s001.zip › cancers-1384109-supplementary/cancers-1384109-western blot/ER paper WBs/Western Scans - Lab Notebook 3 IN BINDER/WB0020.pdf]

6.9.2020

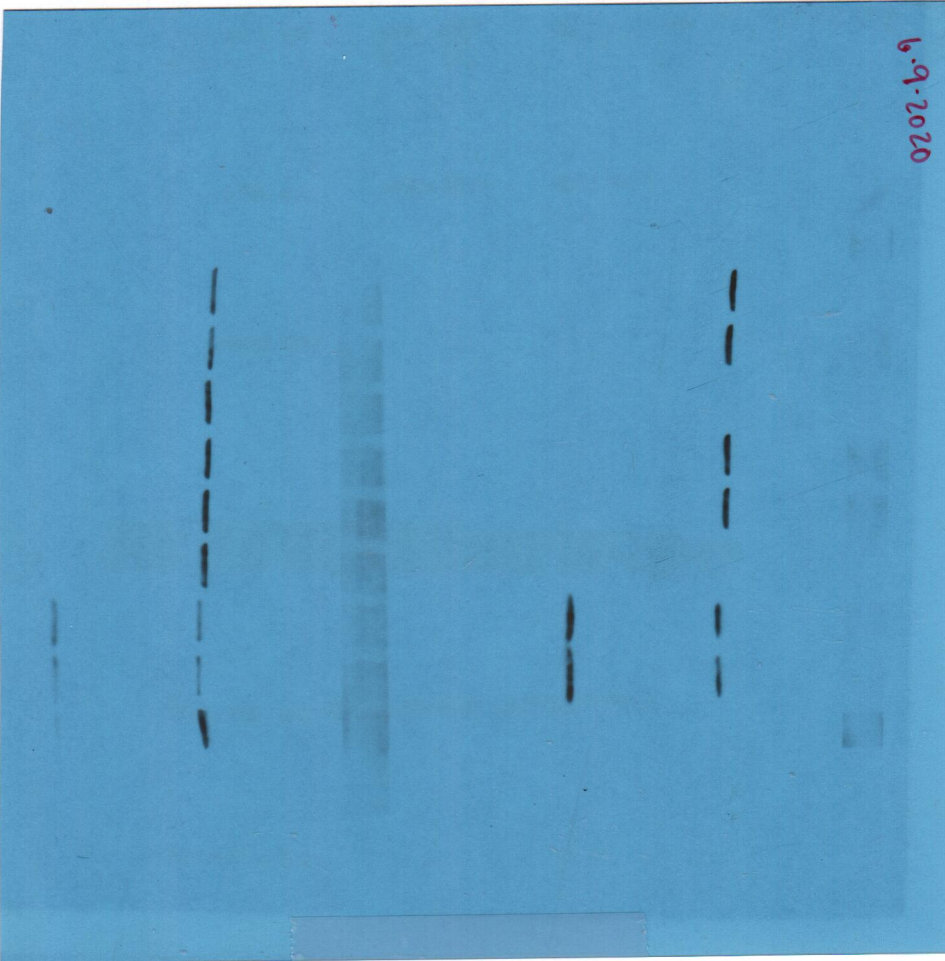

6.9.2026

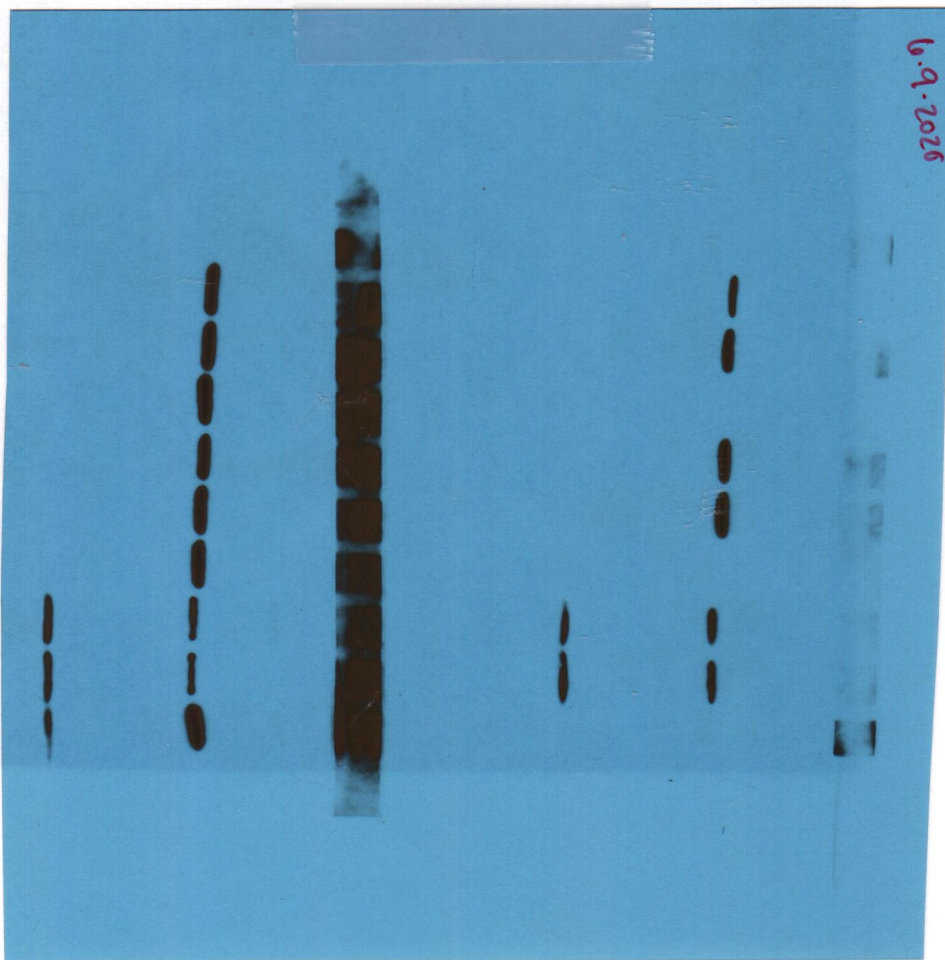

Supplement: Supplementary file 1 [file cancers-13-05130-s001.zip › cancers-1384109-supplementary/cancers-1384109-western blot/ER paper WBs/Western Scans - Lab Notebook 3 IN BINDER/WB0021.pdf]

6.9.2020

S1E2 blot

S1STAT5 blot

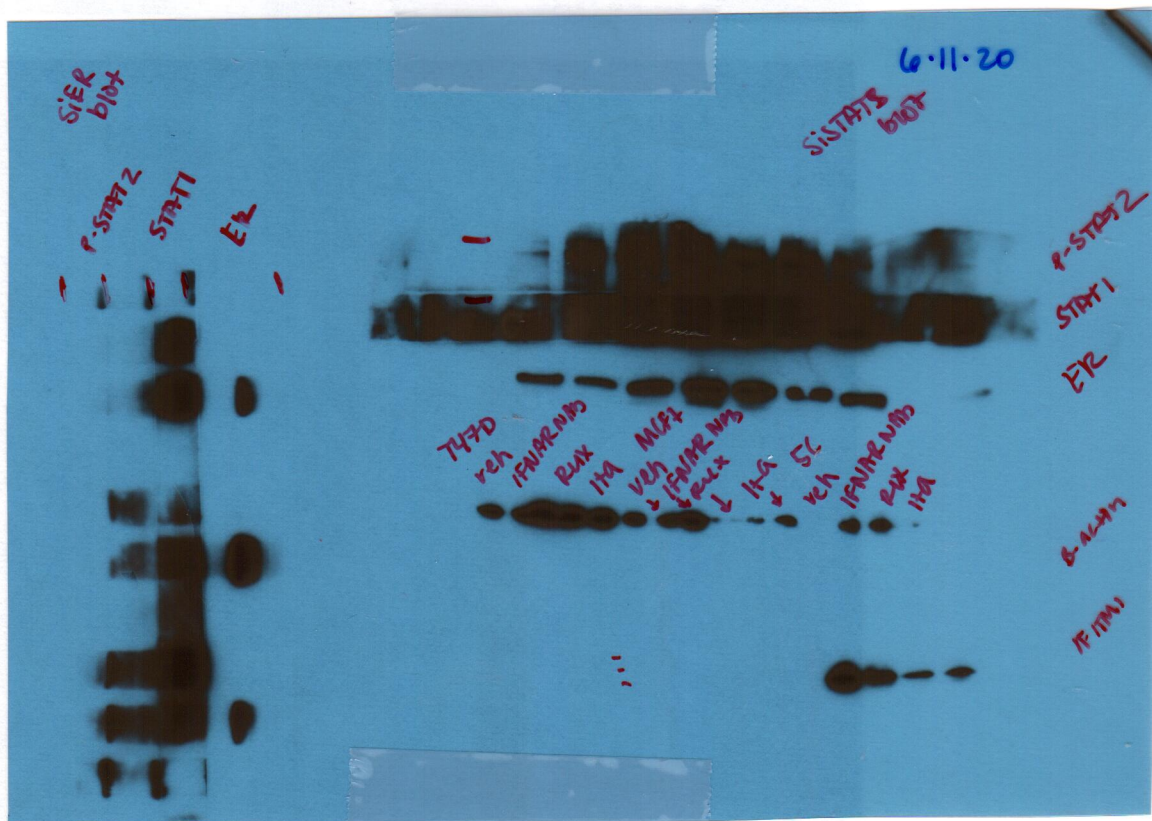

Supplement: Supplementary file 1 [file cancers-13-05130-s001.zip › cancers-1384109-supplementary/cancers-1384109-western blot/ER paper WBs/Western Scans - Lab Notebook 3 IN BINDER/WB0022.pdf]

6.9.2020

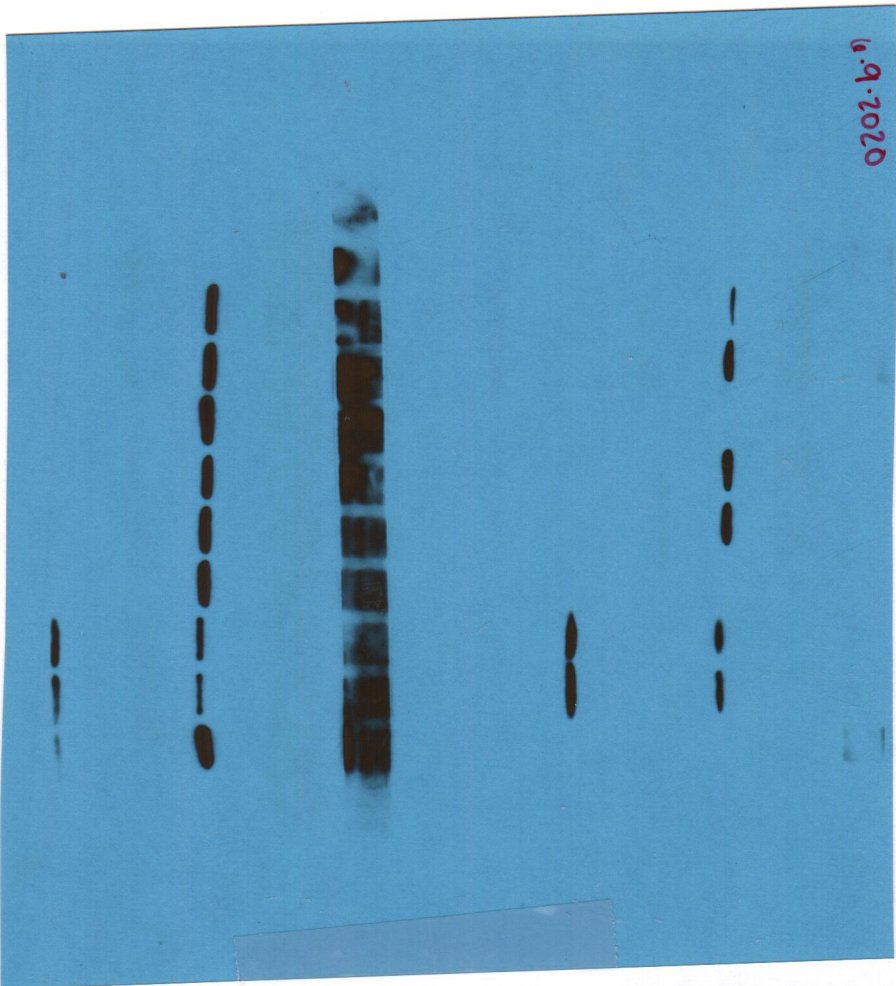

6.9.2020

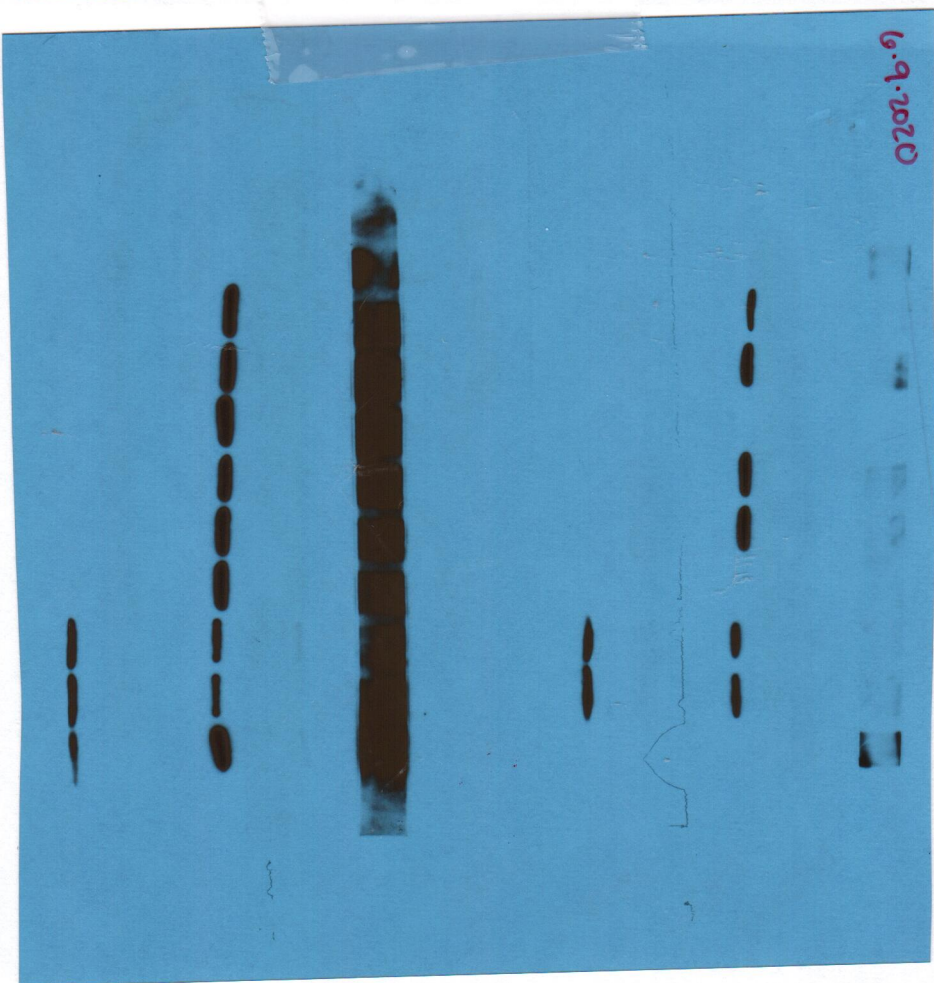

Supplement: Supplementary file 1 [file cancers-13-05130-s001.zip › cancers-1384109-supplementary/cancers-1384109-western blot/ER paper WBs/Western Scans - Lab Notebook 3 IN BINDER/WB0023.pdf]

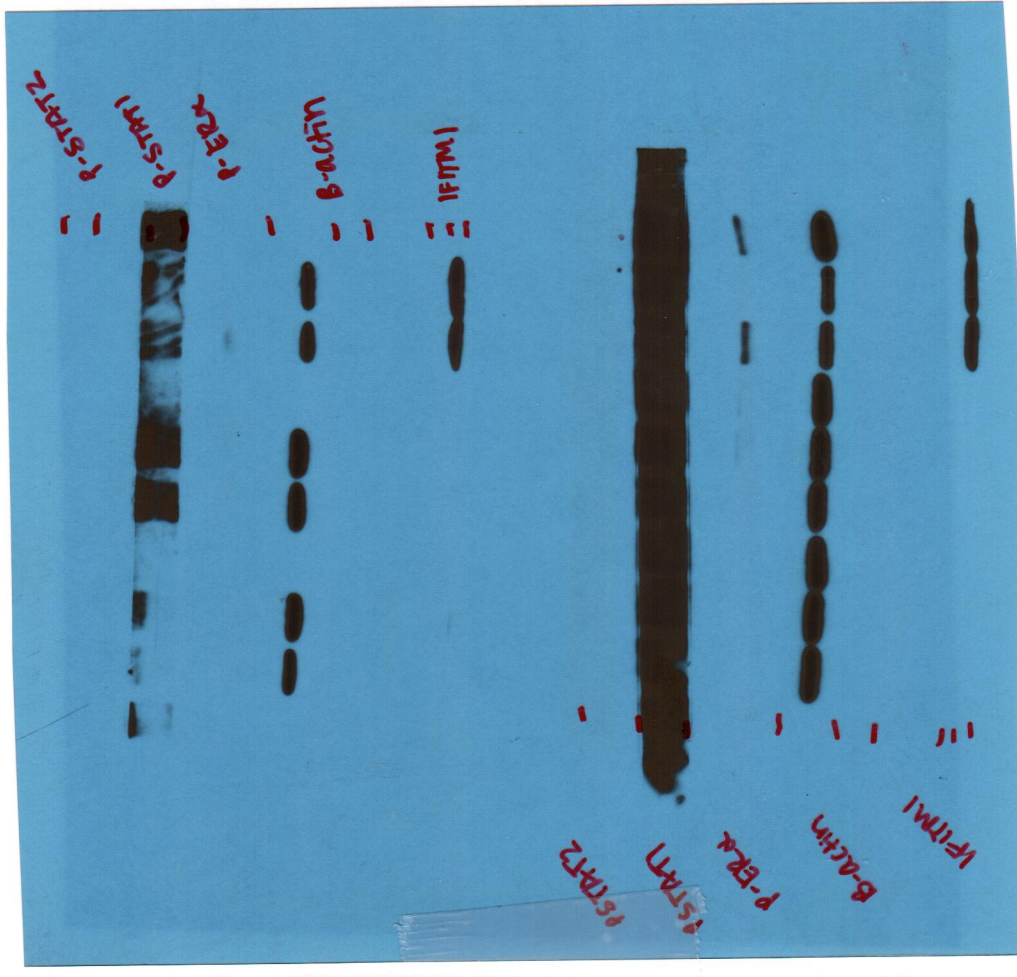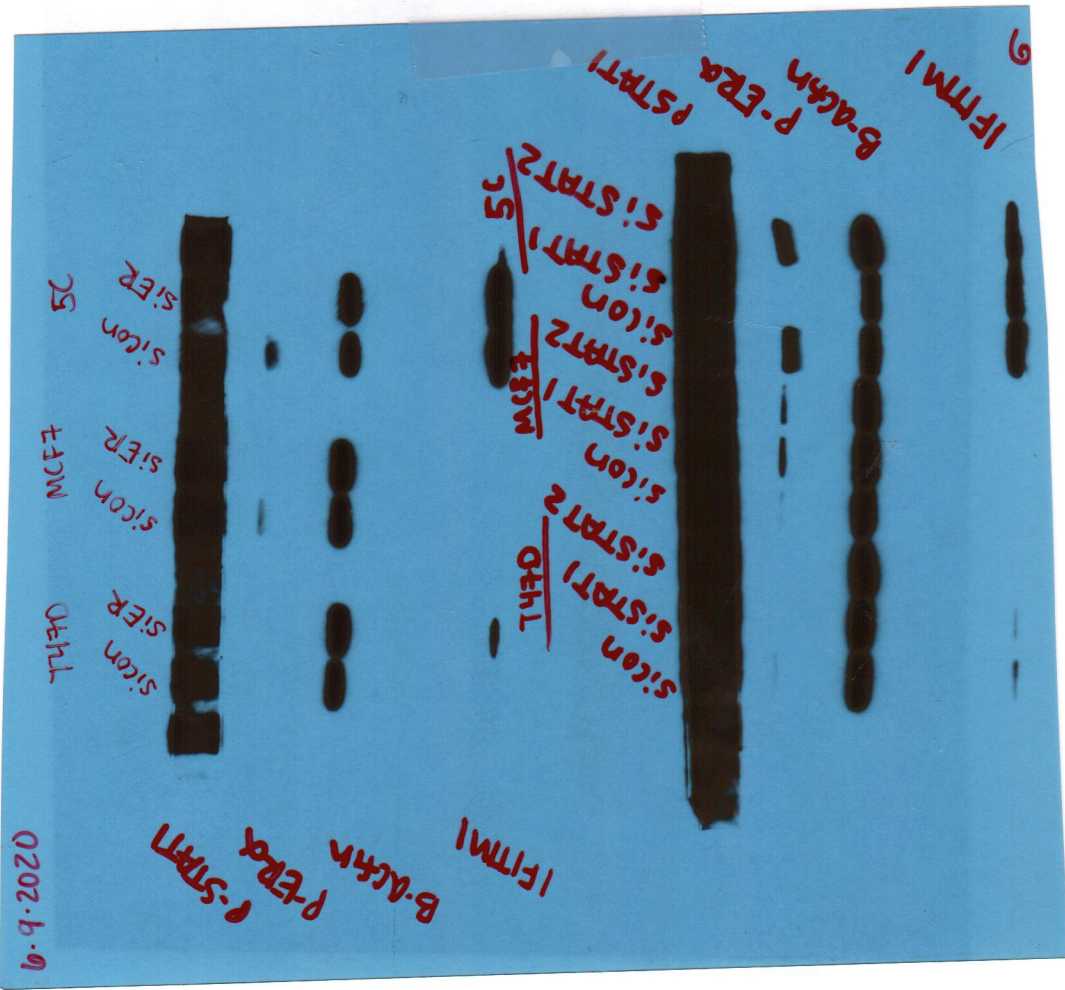

Supplement: Supplementary file 1 [file cancers-13-05130-s001.zip › cancers-1384109-supplementary/cancers-1384109-western blot/ER paper WBs/Western Scans - Lab Notebook 3 IN BINDER/WB0024.pdf]

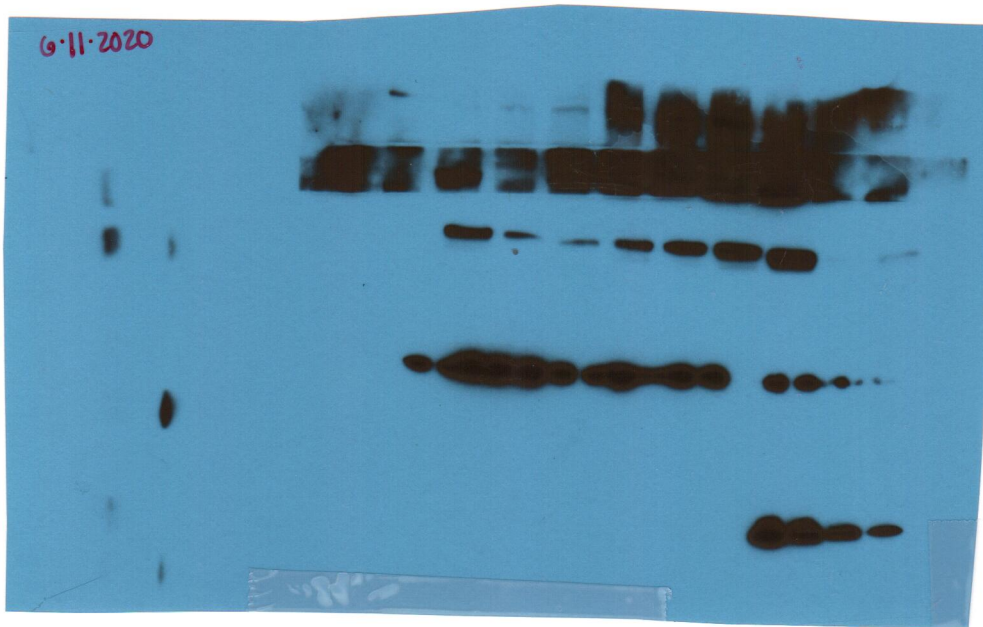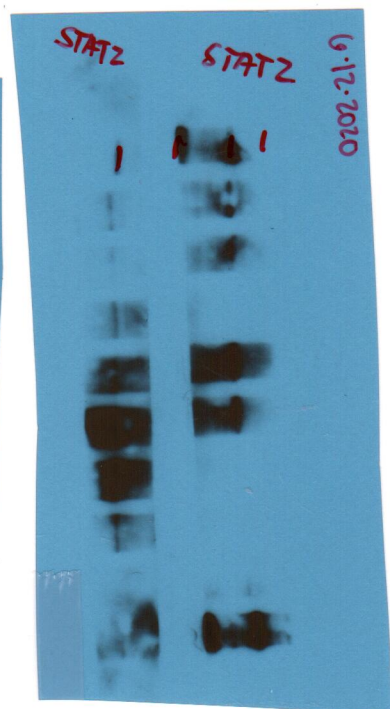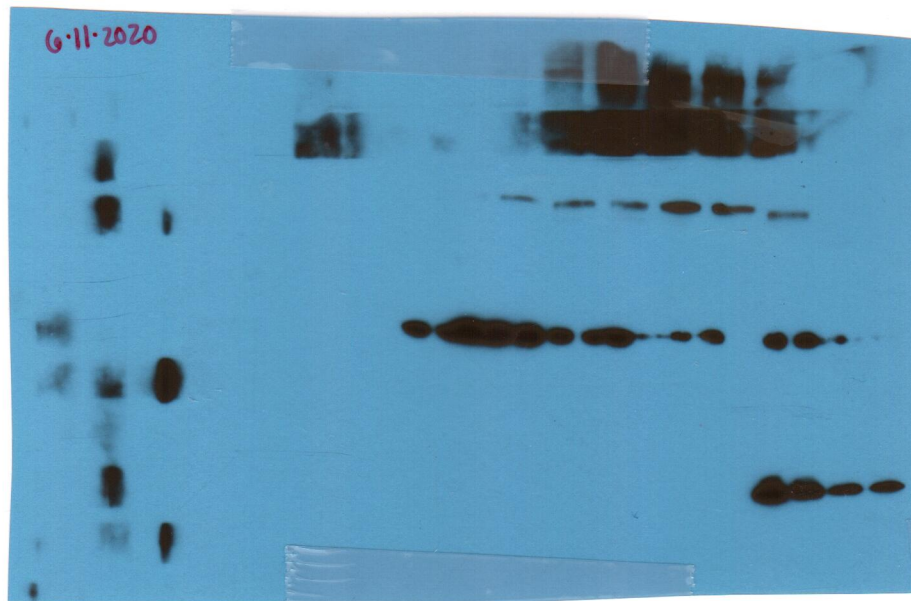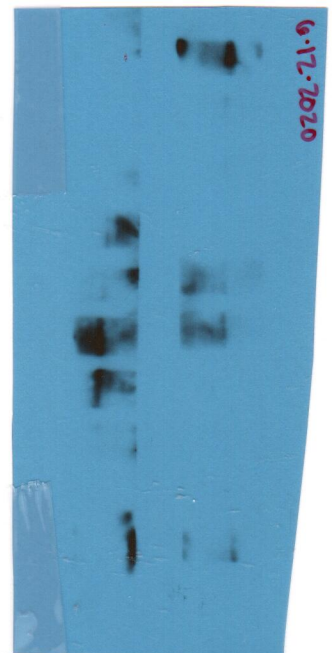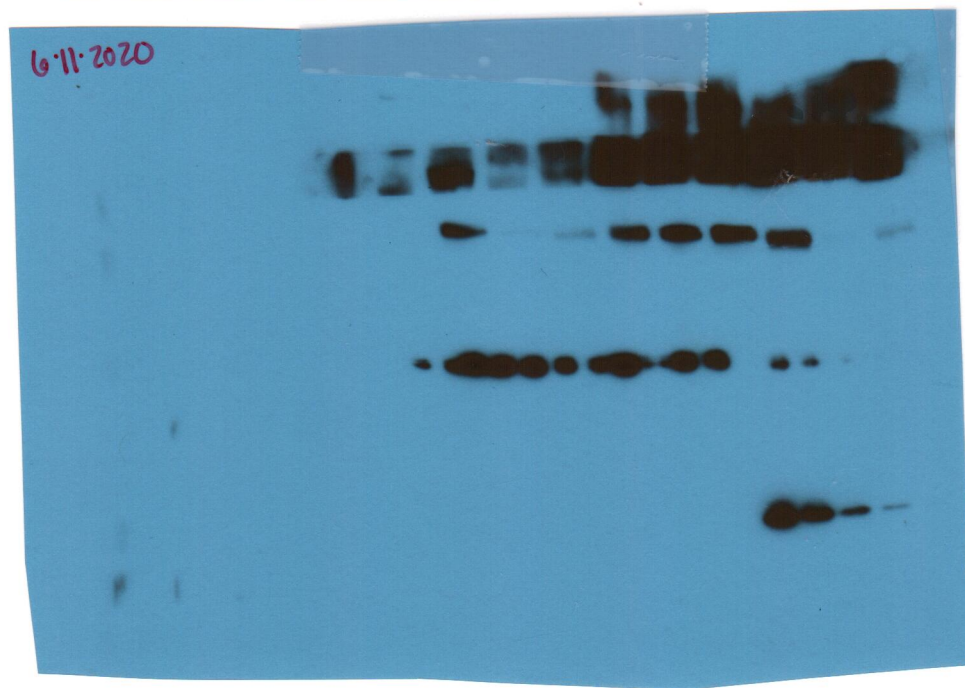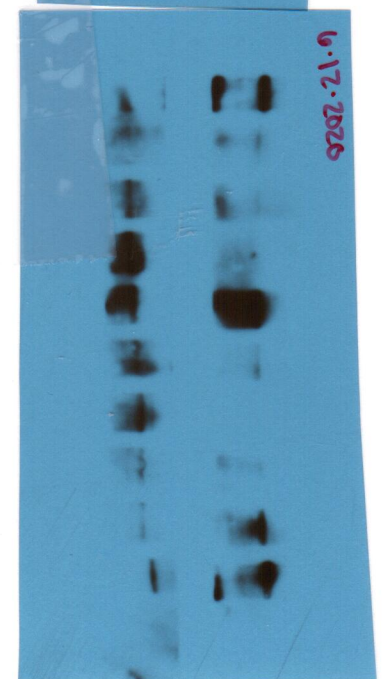

Supplement: Supplementary file 1 [file cancers-13-05130-s001.zip › cancers-1384109-supplementary/cancers-1384109-western blot/ER paper WBs/Western Scans - Lab Notebook 3 IN BINDER/WB0025.pdf]

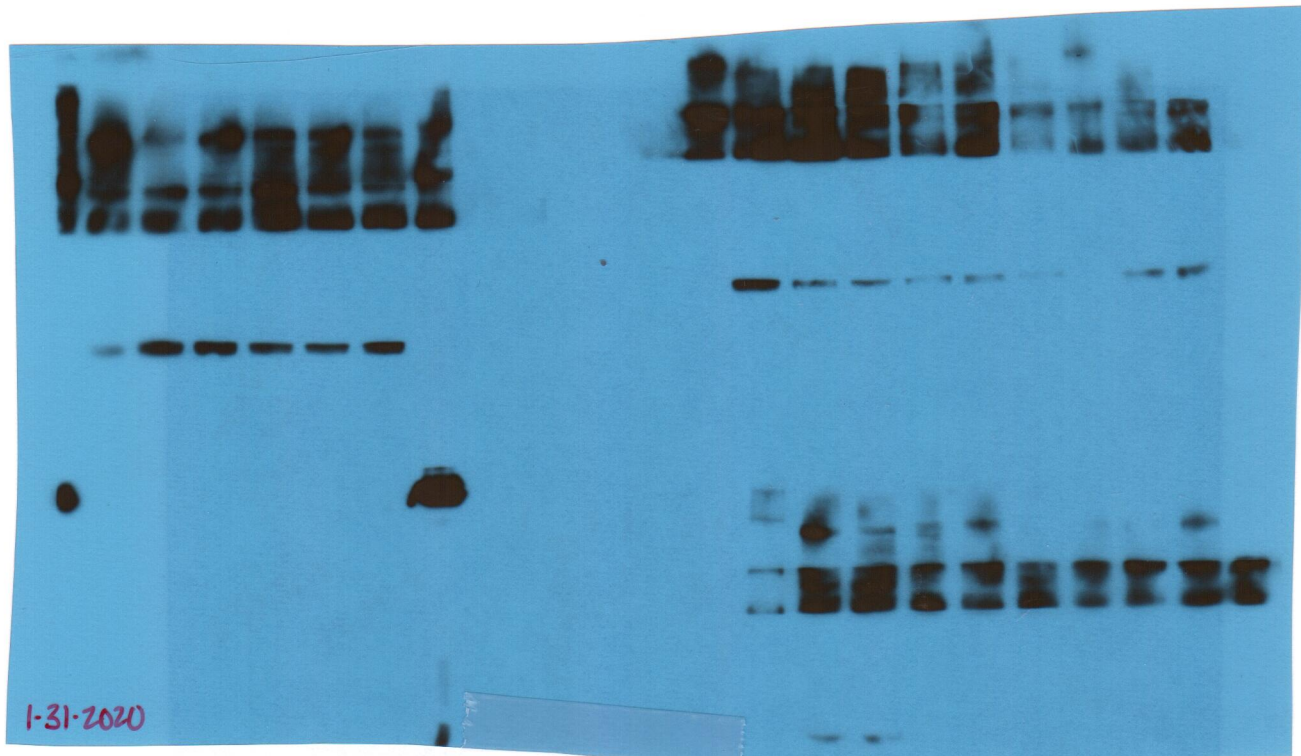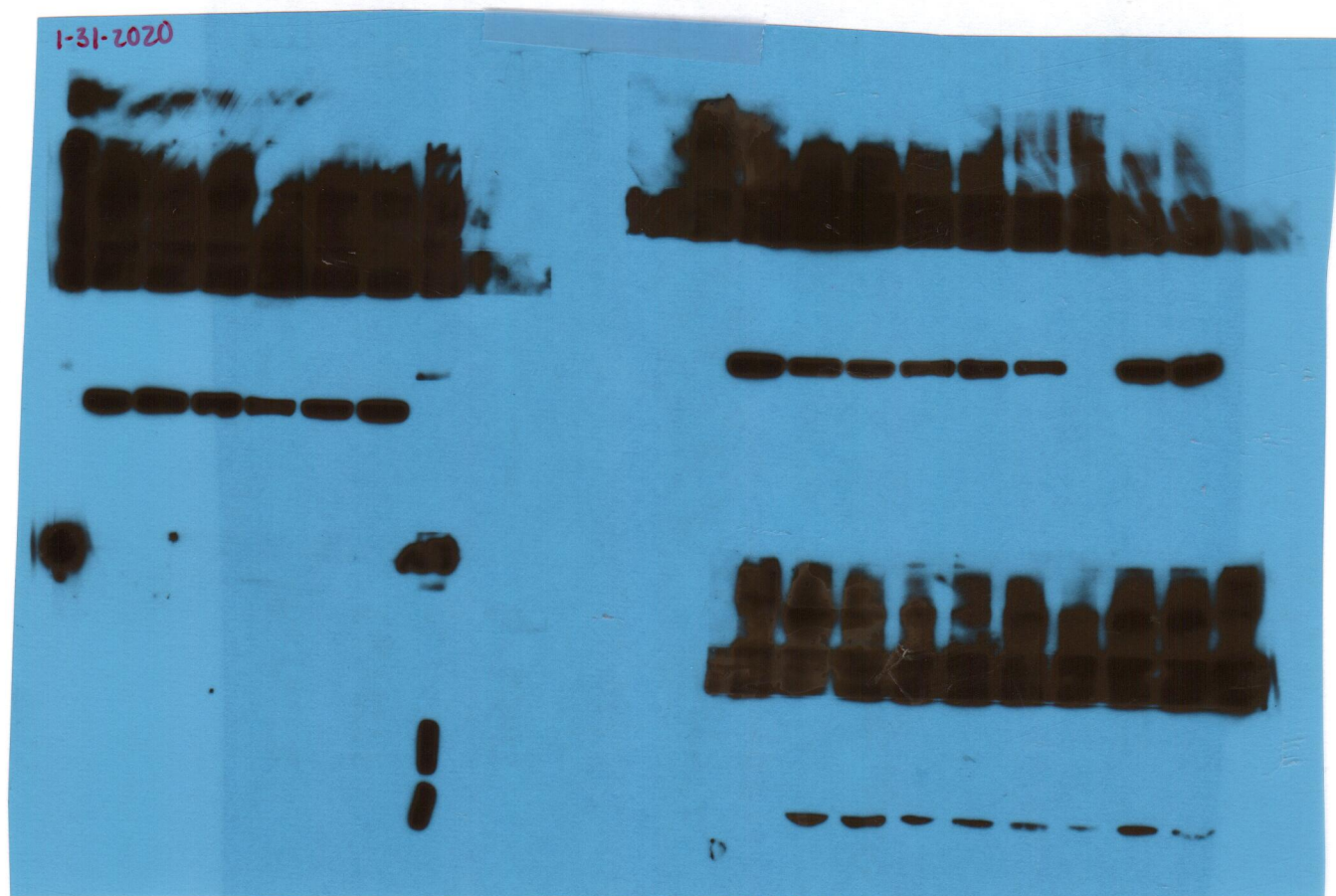

Supplement: Supplementary file 1 [file cancers-13-05130-s001.zip › cancers-1384109-supplementary/cancers-1384109-western blot/ER paper WBs/Western Scans - Lab Notebook 3 IN BINDER/WB0027.pdf]

6-22-2020

THD

STAT2

STAT1

ERK

STAT2 MAP2

STAT1

ERK

STAT2 SL

STAT1

ERK

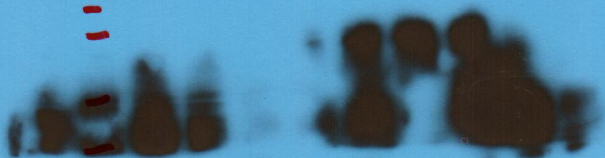

1111

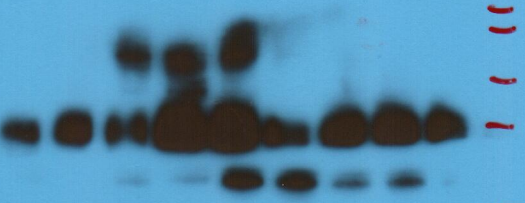

11111111

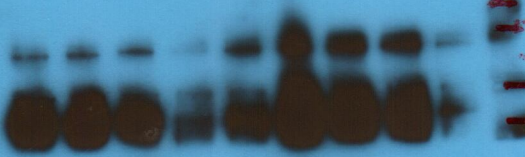

11111111

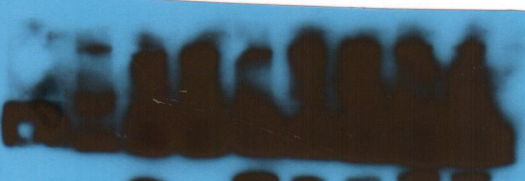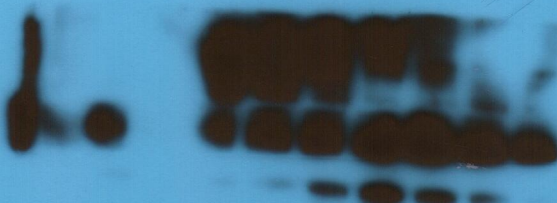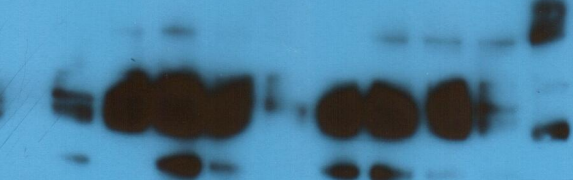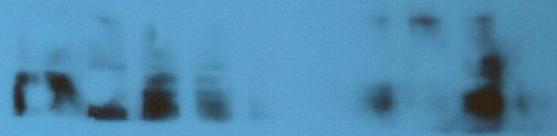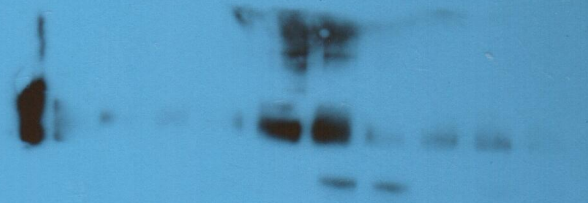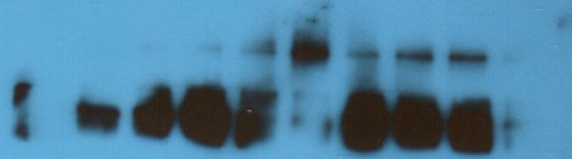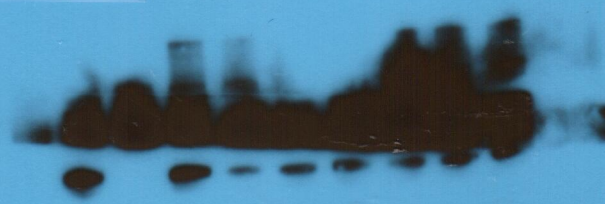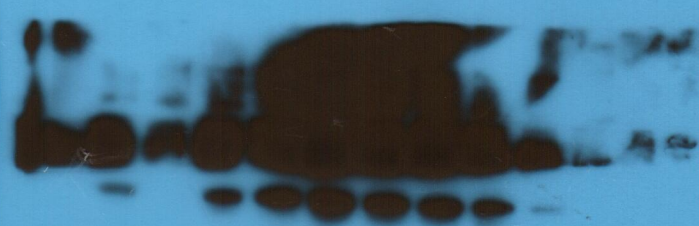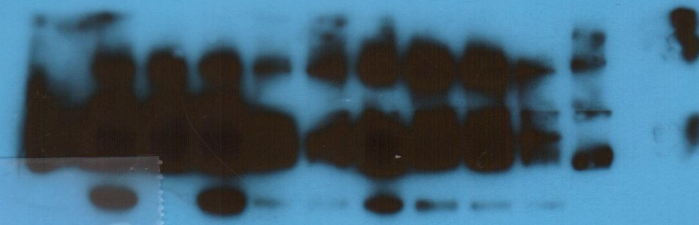

Supplement: Supplementary file 1 [file cancers-13-05130-s001.zip › cancers-1384109-supplementary/cancers-1384109-western blot/ER paper WBs/Western Scans - Lab Notebook 3 IN BINDER/WB0028.pdf]

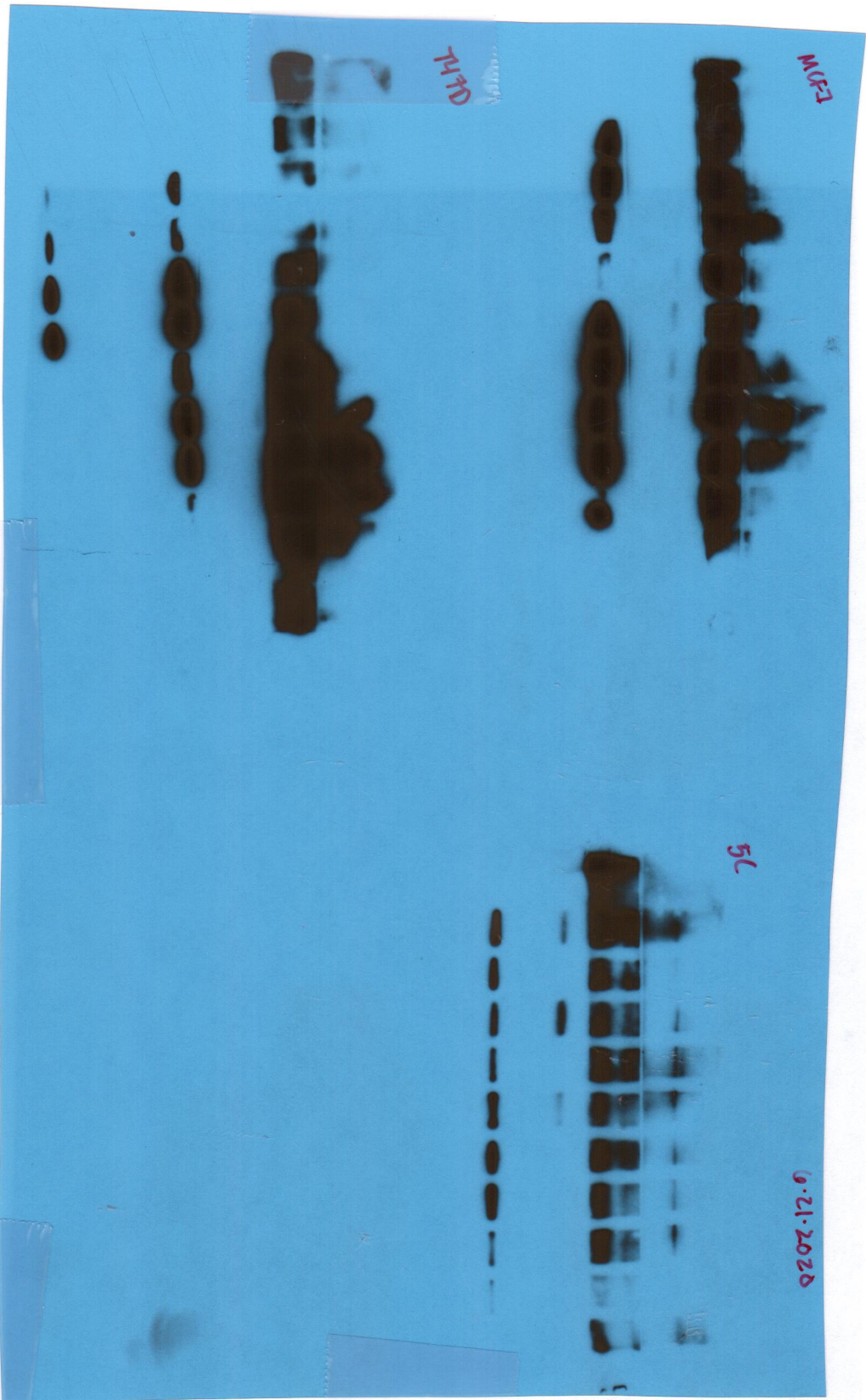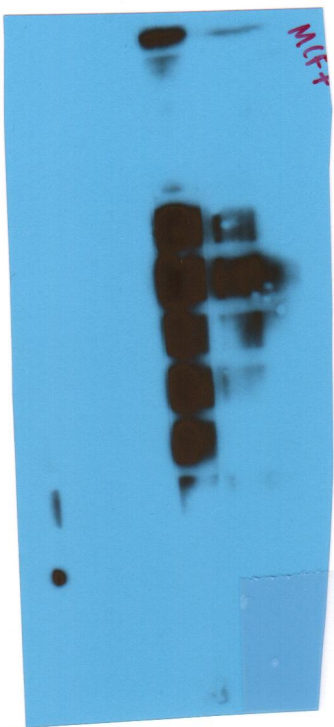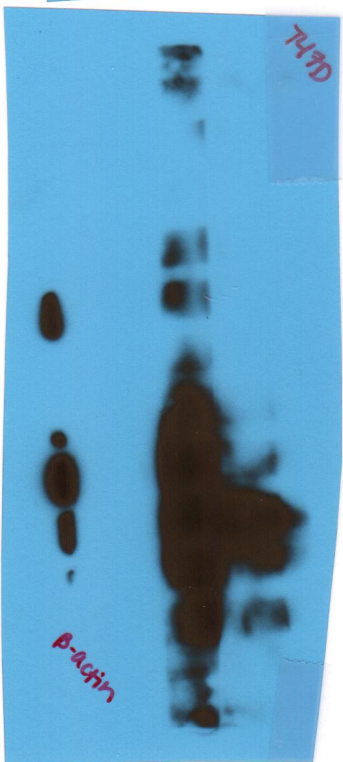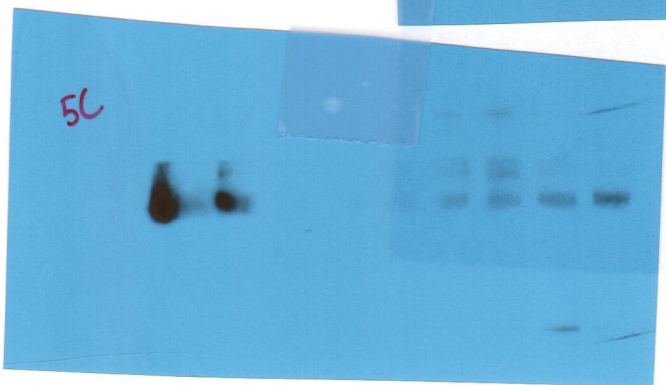

Supplement: Supplementary file 1 [file cancers-13-05130-s001.zip › cancers-1384109-supplementary/cancers-1384109-western blot/ER paper WBs/Western Scans - Lab Notebook 3 IN BINDER/WB0029.pdf]

0202-12.9

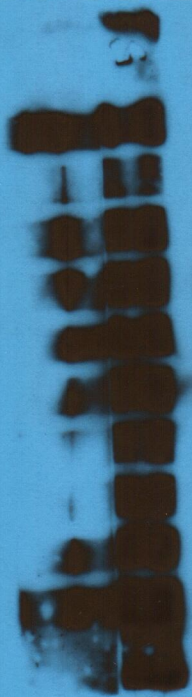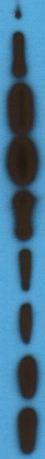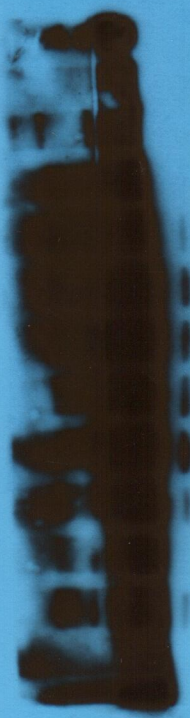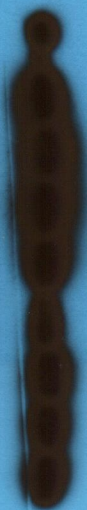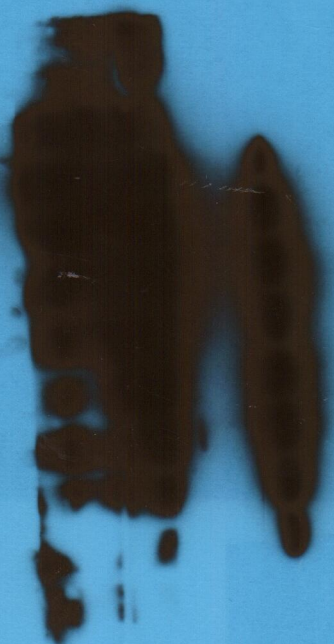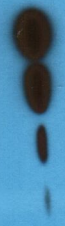

Supplement: Supplementary file 1 [file cancers-13-05130-s001.zip › cancers-1384109-supplementary/cancers-1384109-western blot/ER paper WBs/Western Scans - Lab Notebook 3 IN BINDER/WB0030.pdf]

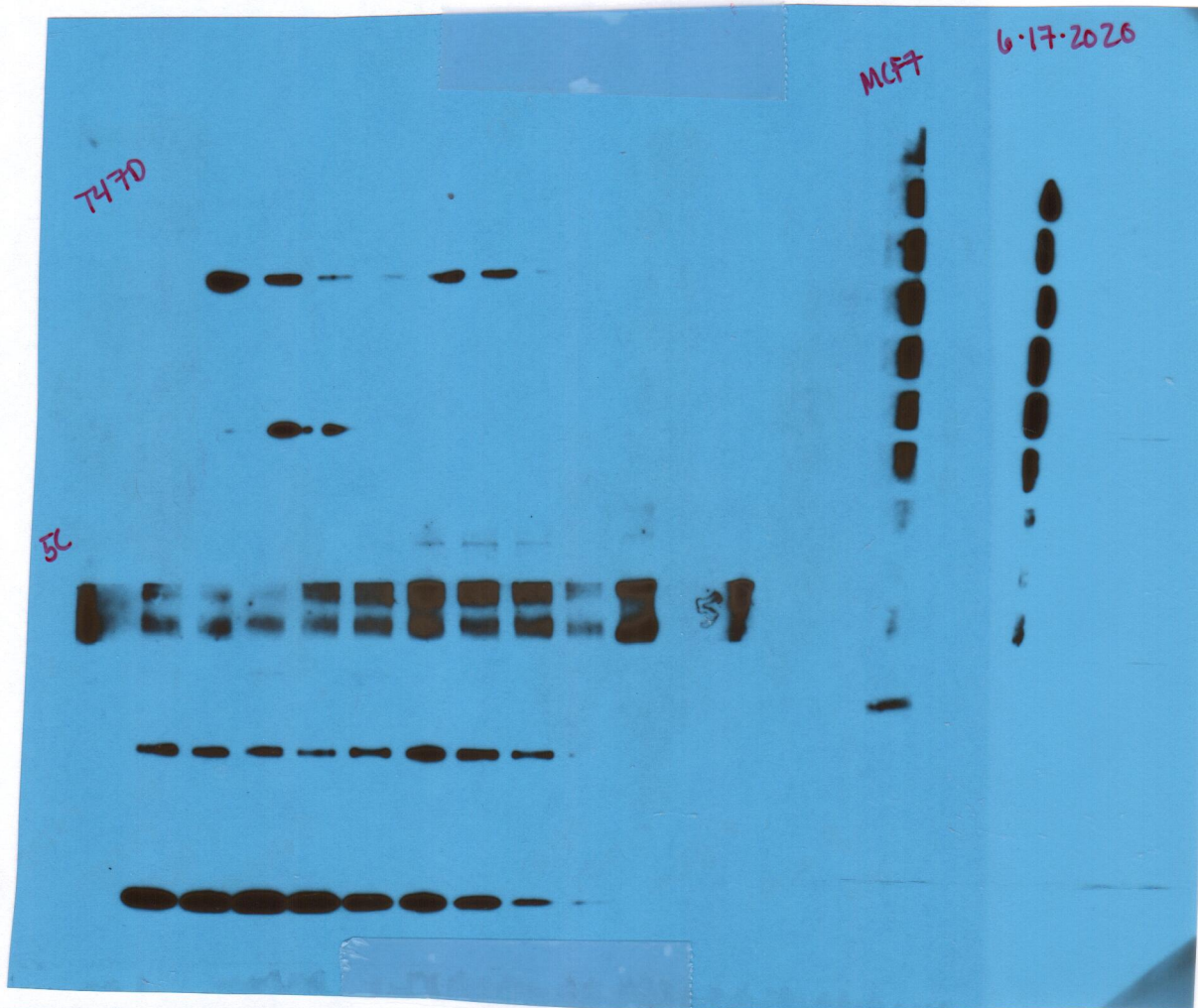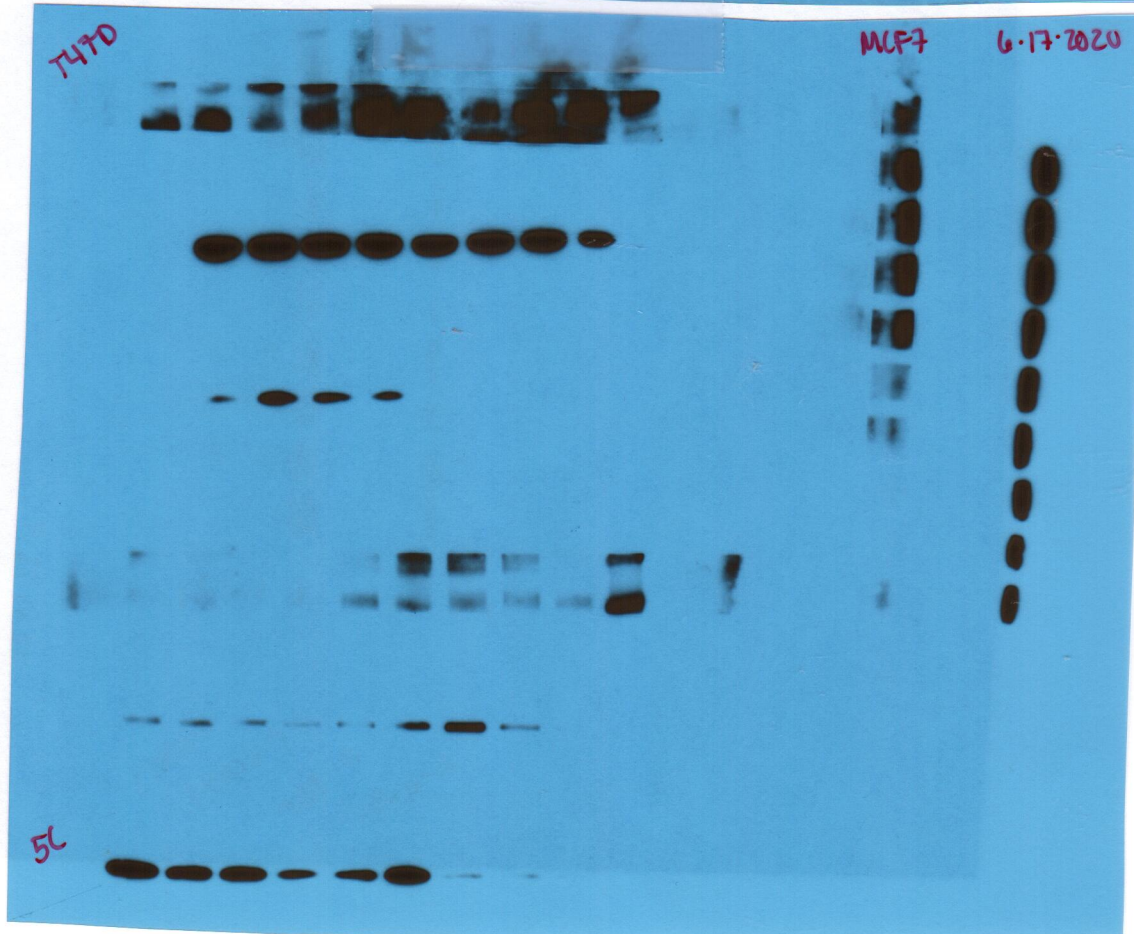

Supplement: Supplementary file 1 [file cancers-13-05130-s001.zip › cancers-1384109-supplementary/cancers-1384109-western blot/ER paper WBs/Western Scans - Lab Notebook 3 IN BINDER/WB0031.pdf]

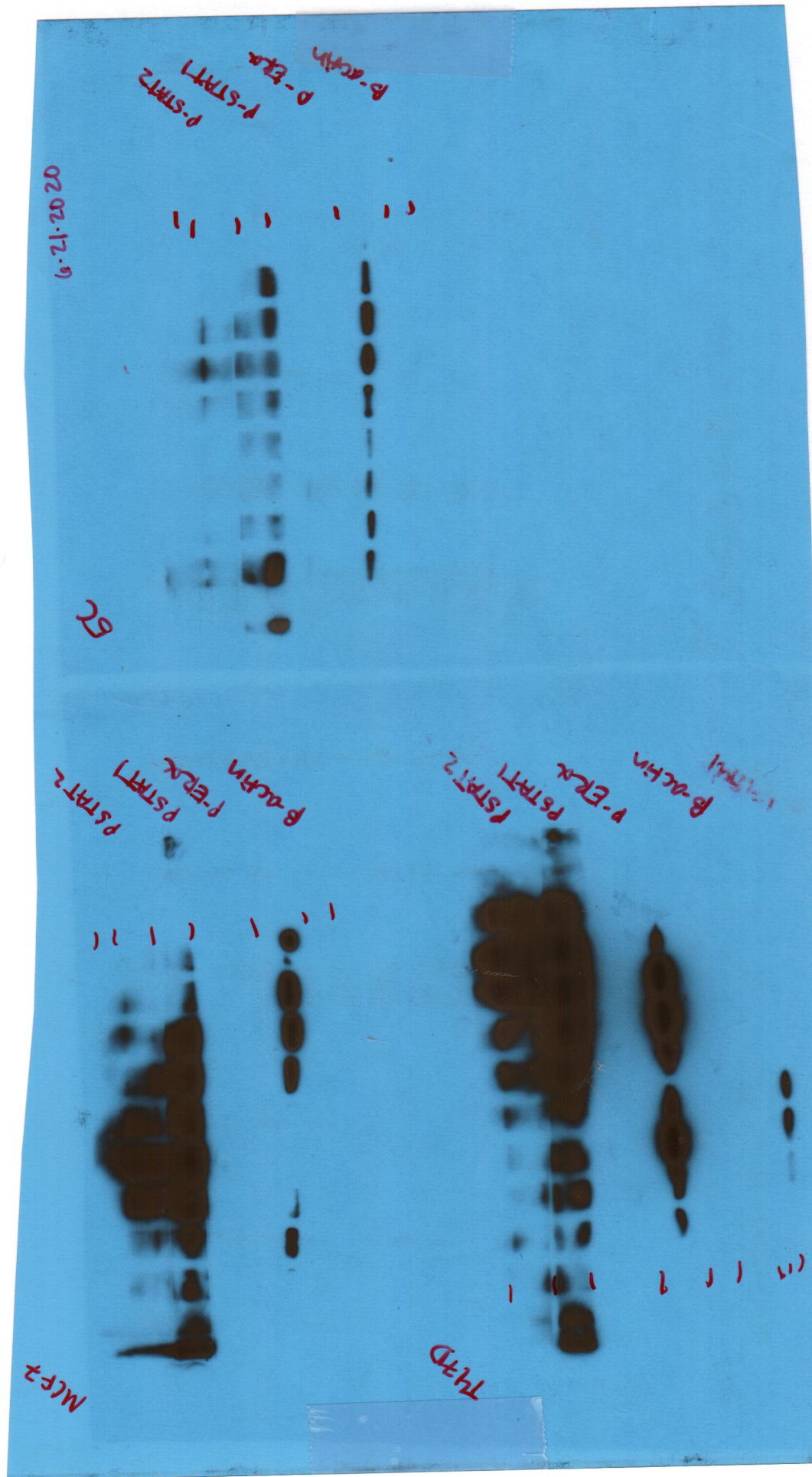

Supplement: Supplementary file 1 [file cancers-13-05130-s001.zip › cancers-1384109-supplementary/cancers-1384109-western blot/ER paper WBs/Western Scans - Lab Notebook 3 IN BINDER/WB0032.pdf]

6.17.2020

7470

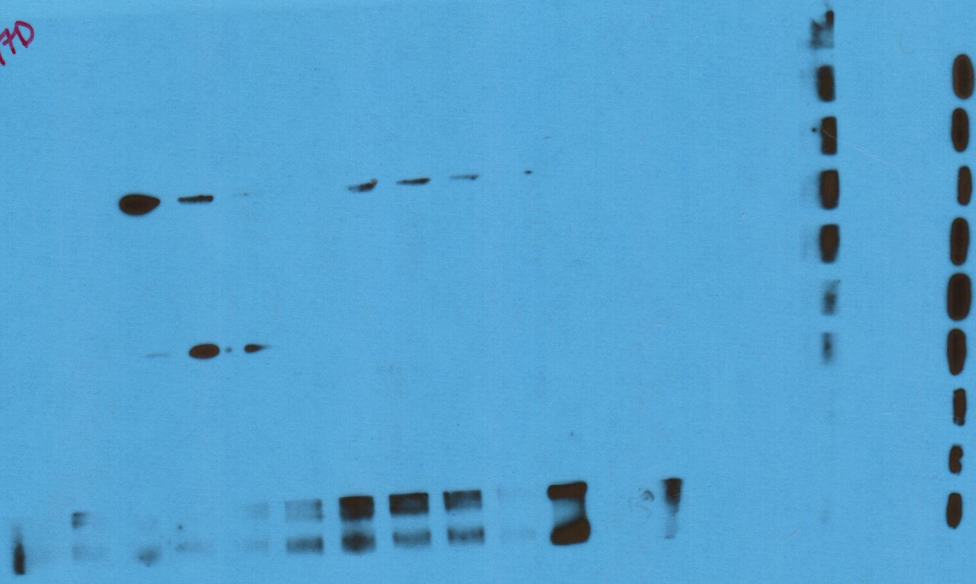

5C

MCF7

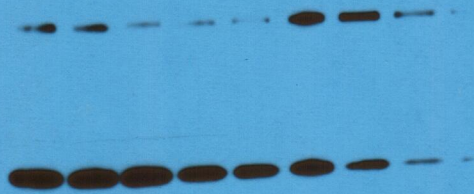

6.17.2020

6.17.2020

Supplement: Supplementary file 1 [file cancers-13-05130-s001.zip › cancers-1384109-supplementary/cancers-1384109-western blot/ER paper WBs/Western Scans - Lab Notebook 3 IN BINDER/WB0033.pdf]

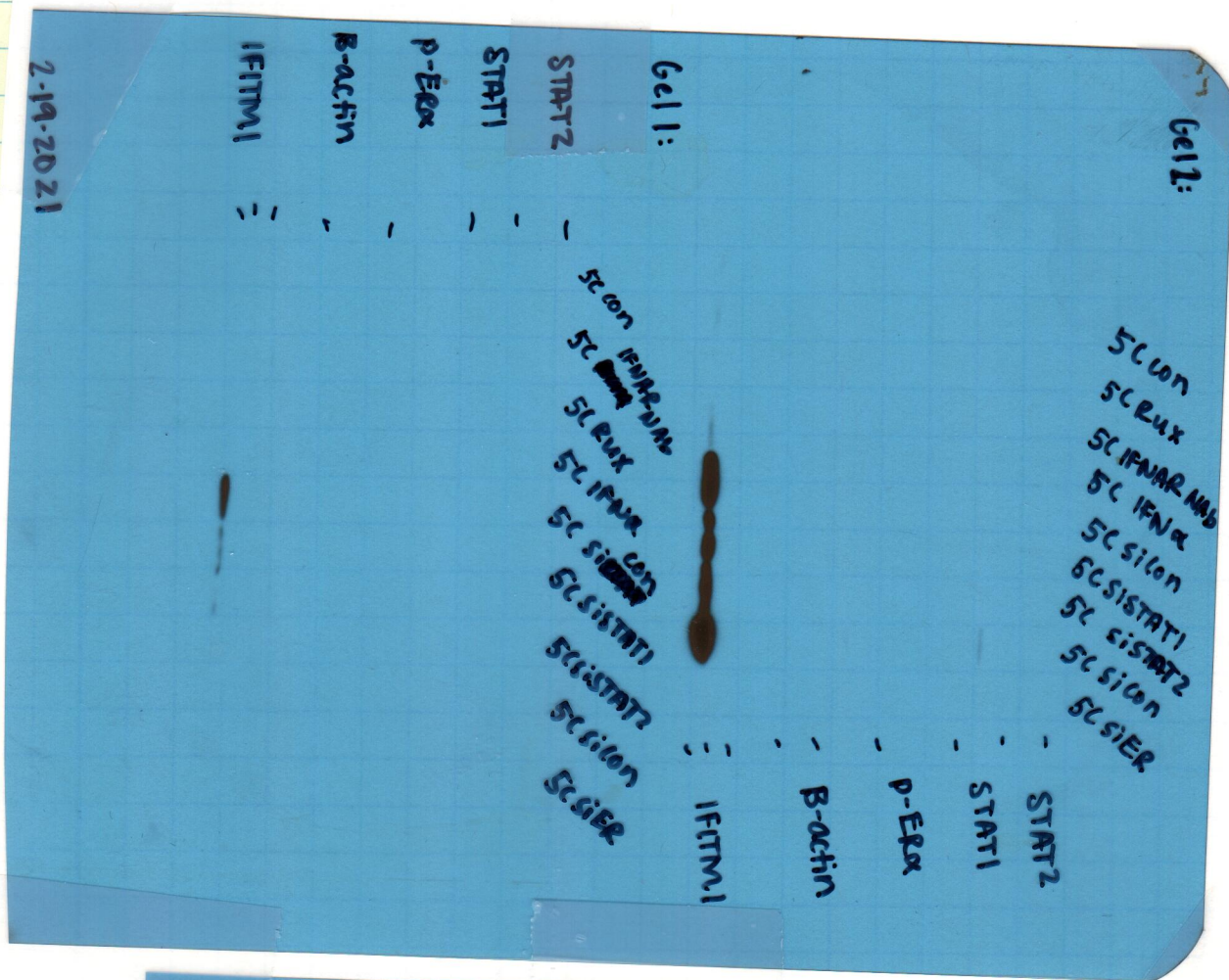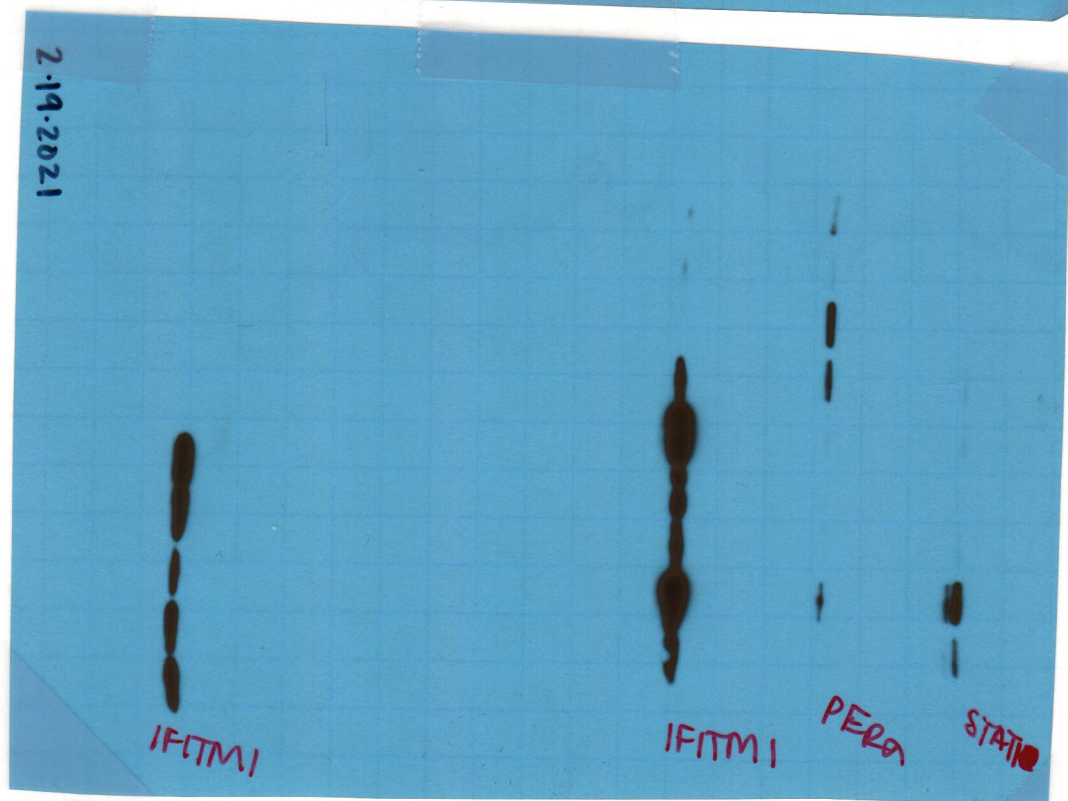

Supplement: Supplementary file 1 [file cancers-13-05130-s001.zip › cancers-1384109-supplementary/cancers-1384109-western blot/ER paper WBs/Western Scans - Lab Notebook 4/WB0003.pdf]

2-26-2021

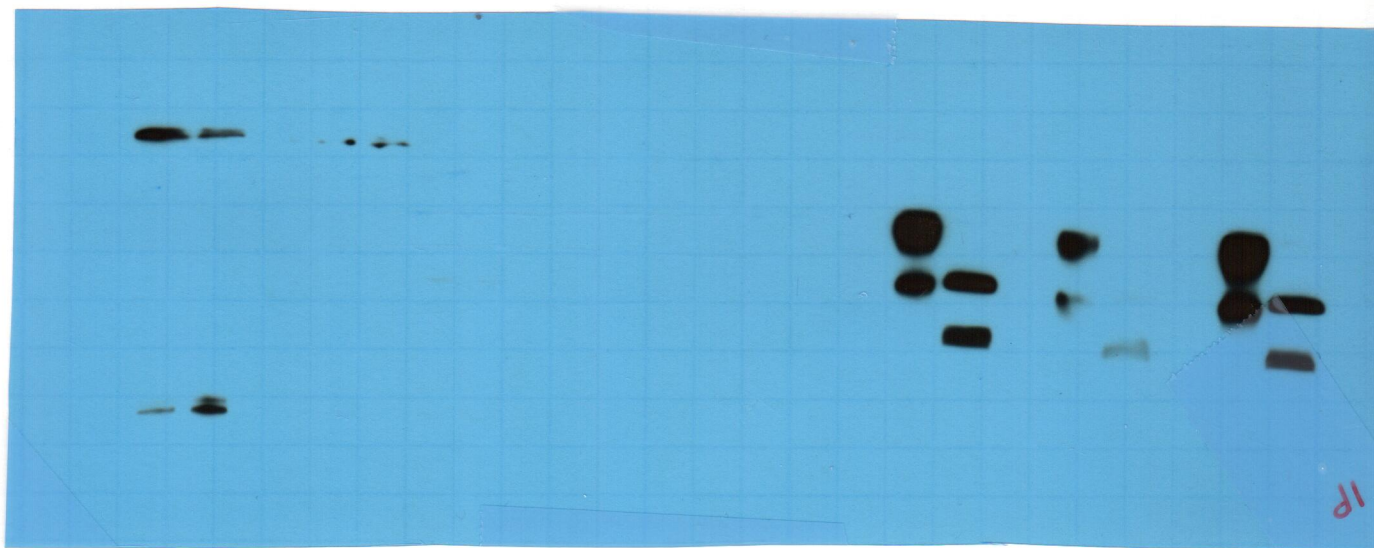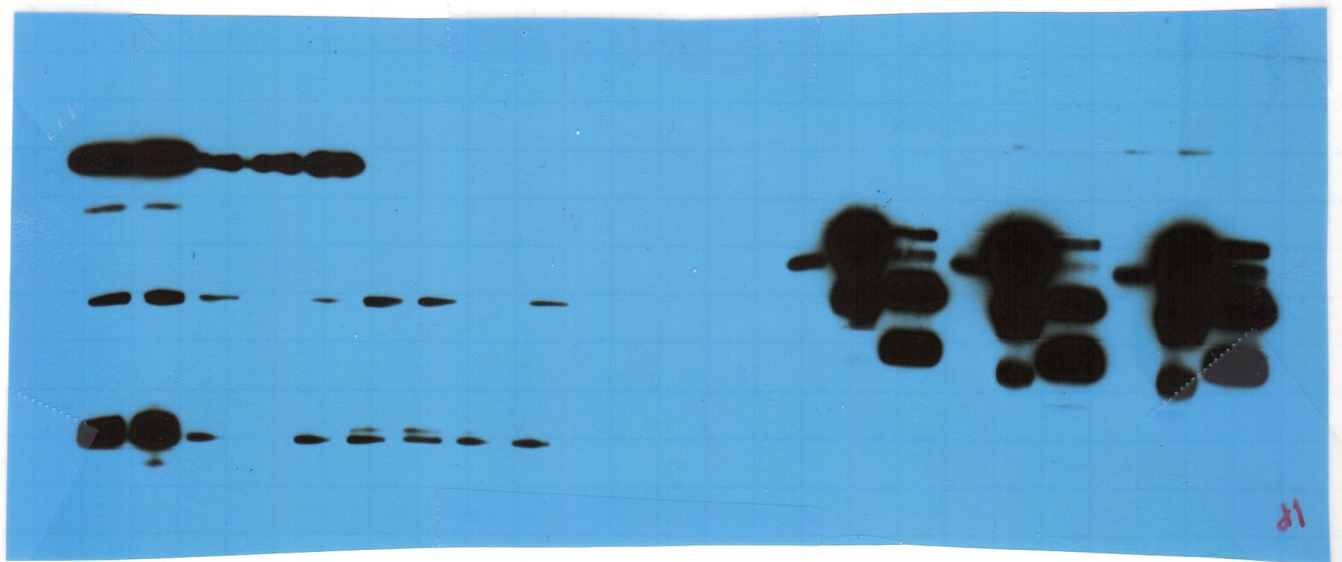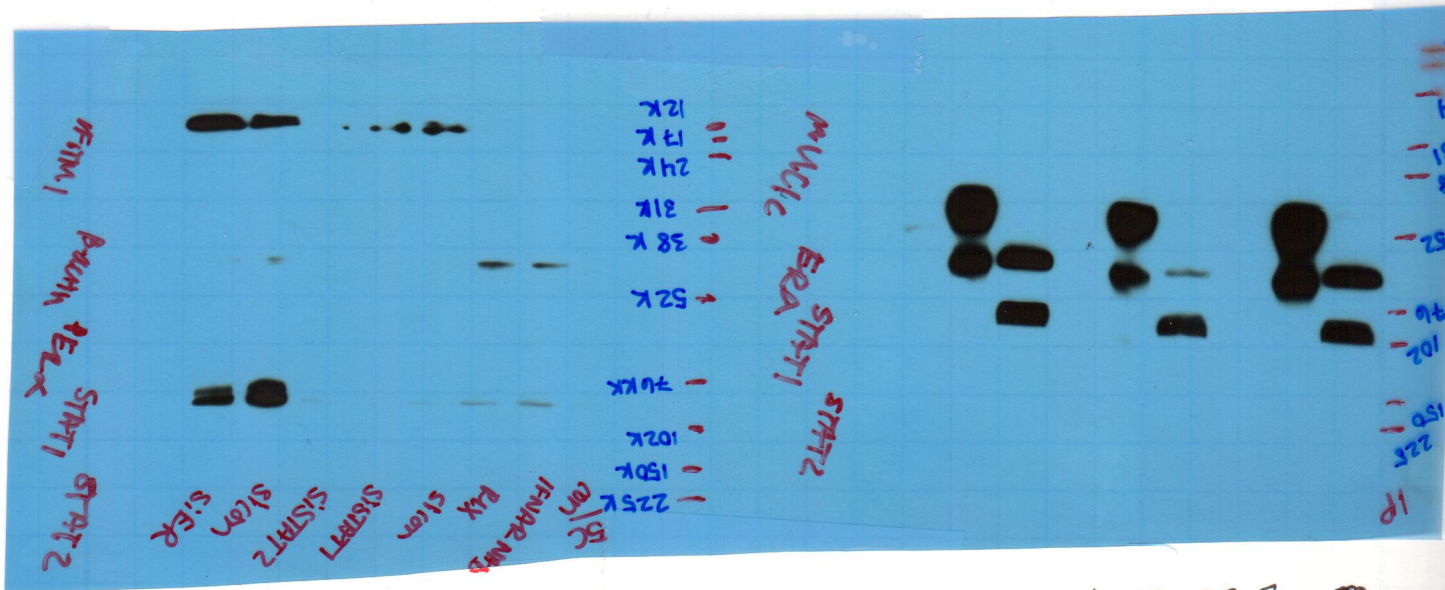

Supplement: Supplementary file 1 [file cancers-13-05130-s001.zip › cancers-1384109-supplementary/cancers-1384109-western blot/ER paper WBs/Western Scans - Lab Notebook 4/WB0006.pdf]

3/2/2021

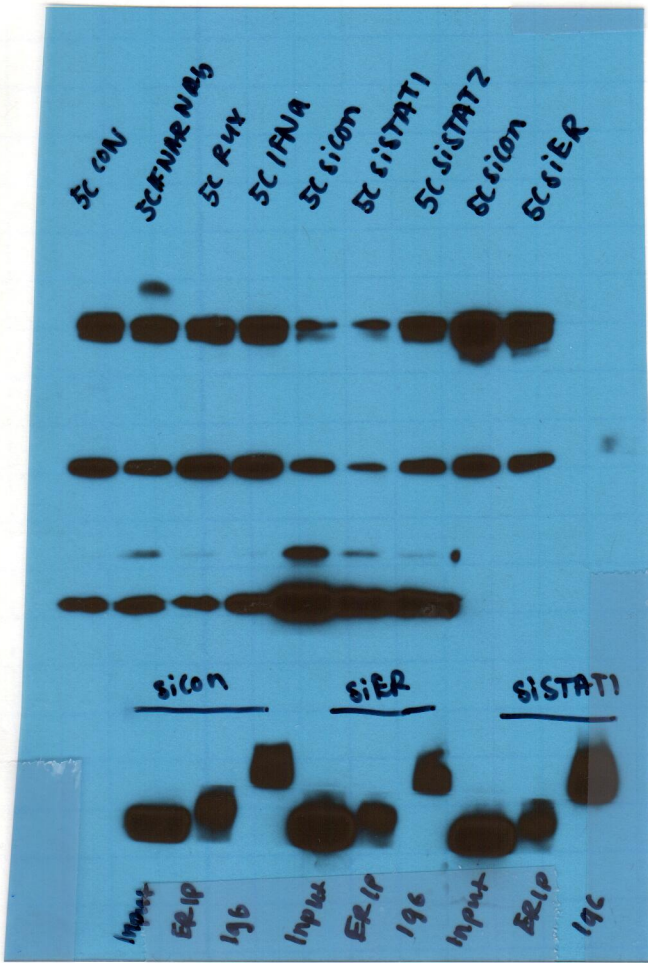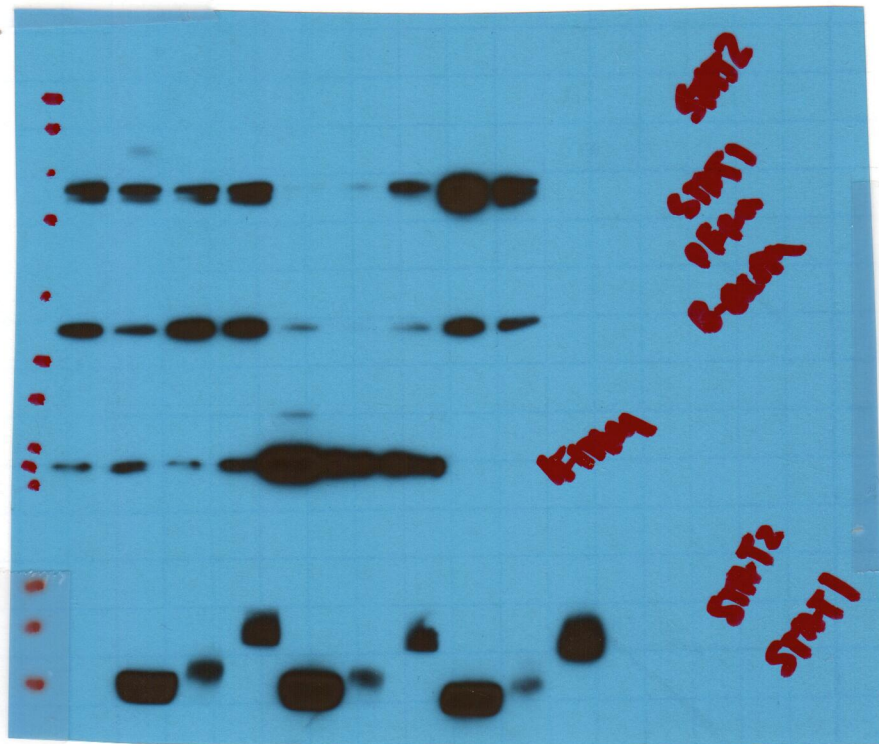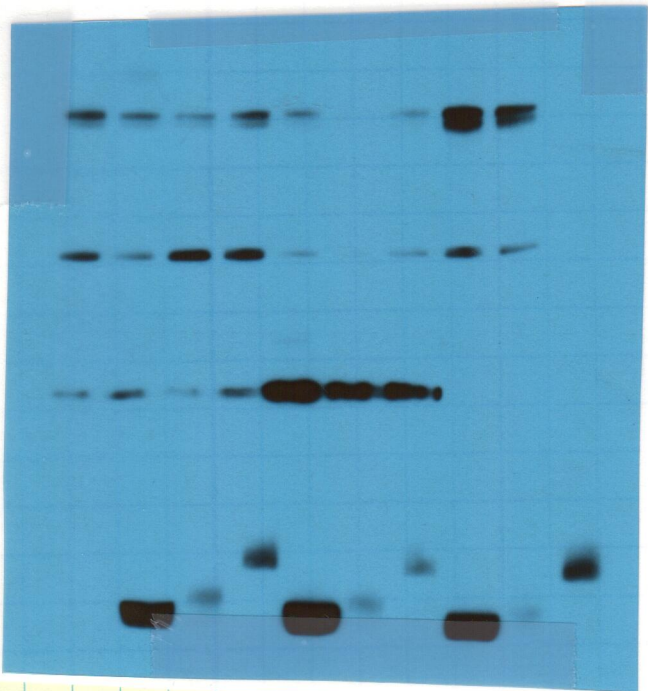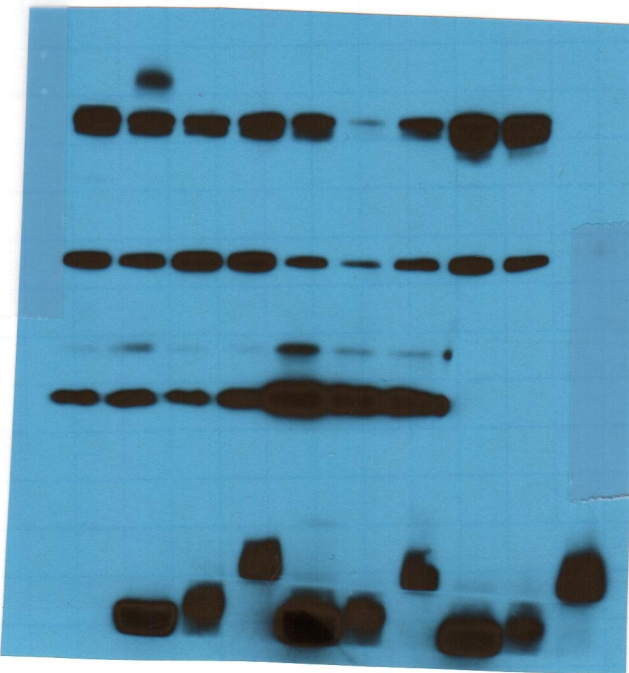

Supplement: Supplementary file 1 [file cancers-13-05130-s001.zip › cancers-1384109-supplementary/cancers-1384109-western blot/ER paper WBs/Western Scans - Lab Notebook 4/WB0007.pdf]

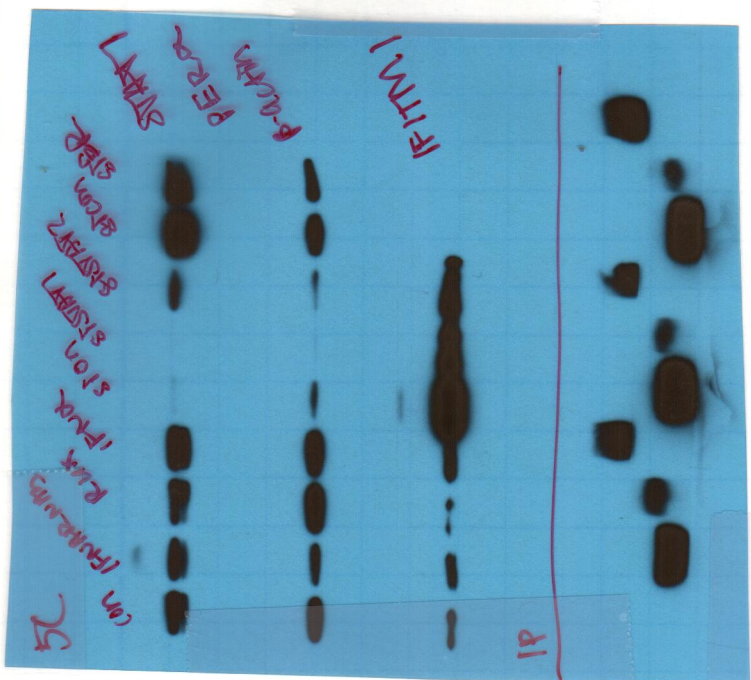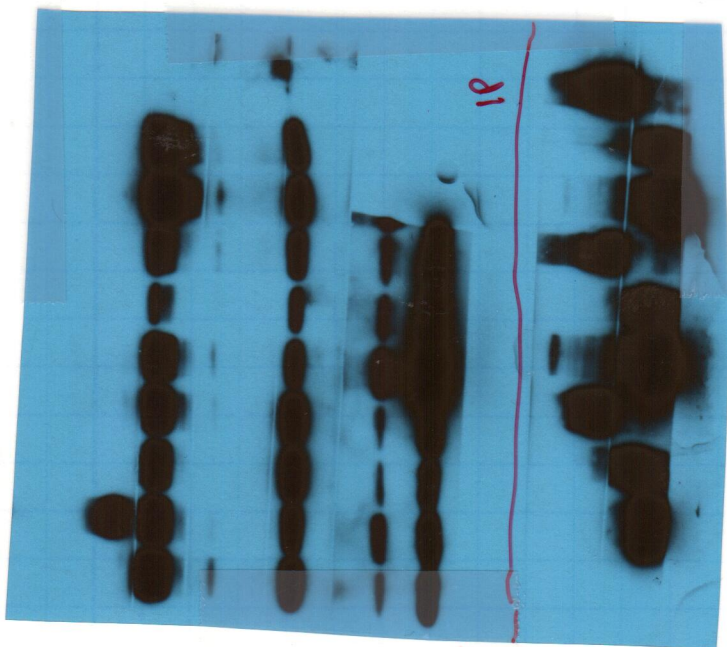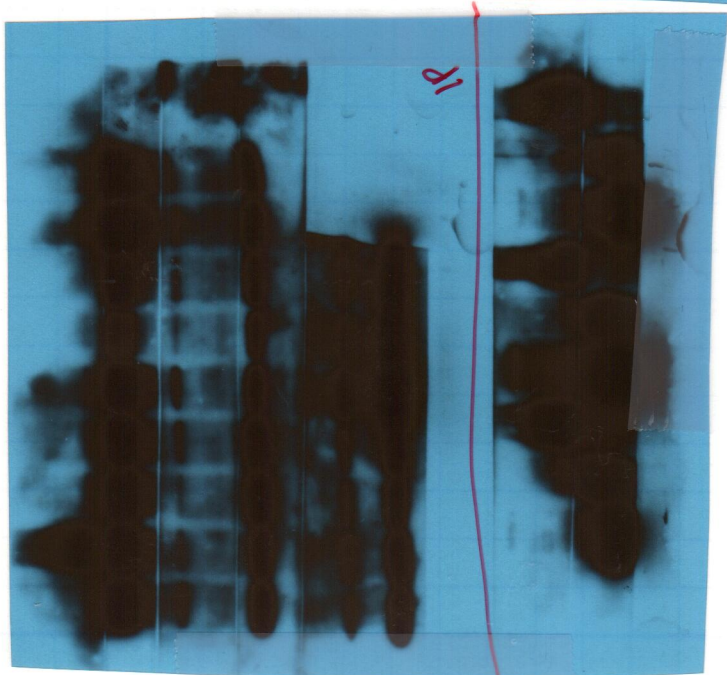

Supplement: Supplementary file 1 [file cancers-13-05130-s001.zip › cancers-1384109-supplementary/cancers-1384109-western blot/ER paper WBs/Western Scans - Lab Notebook 4/WB0008.pdf]

3-9-2021

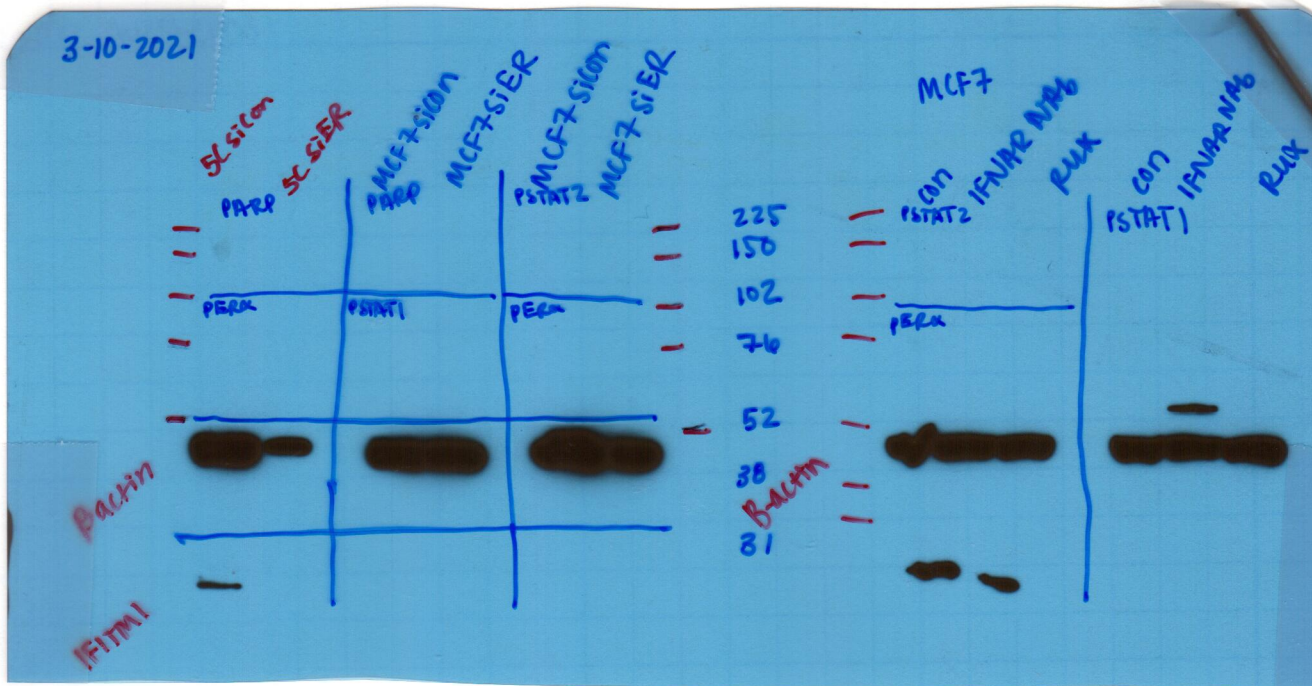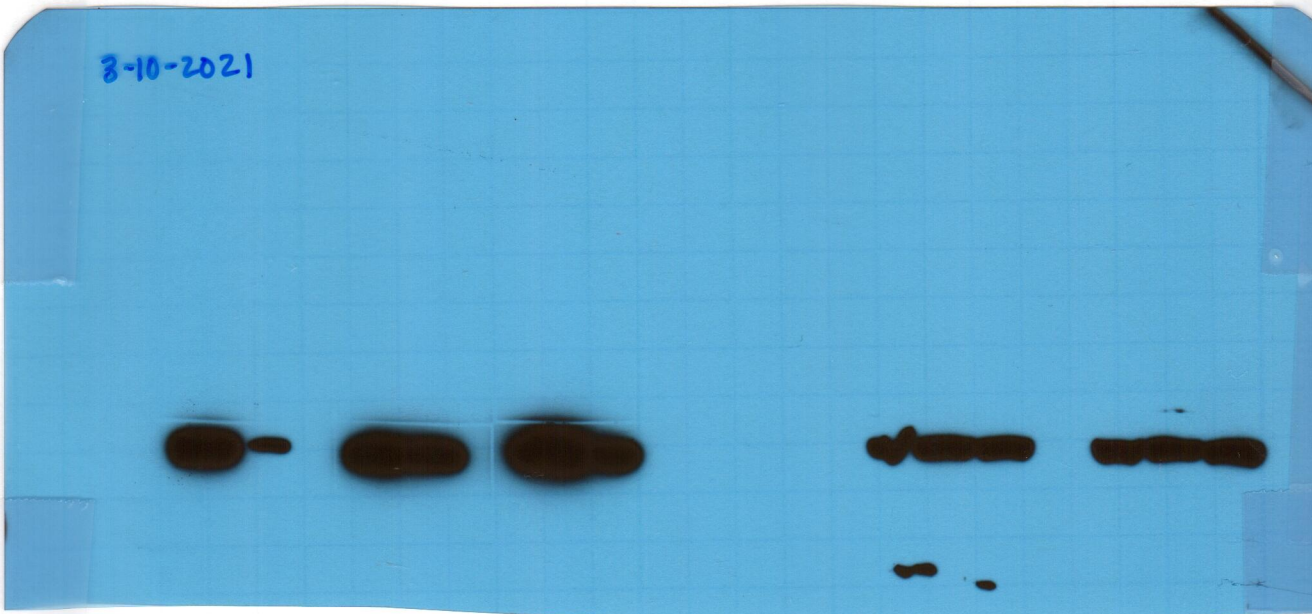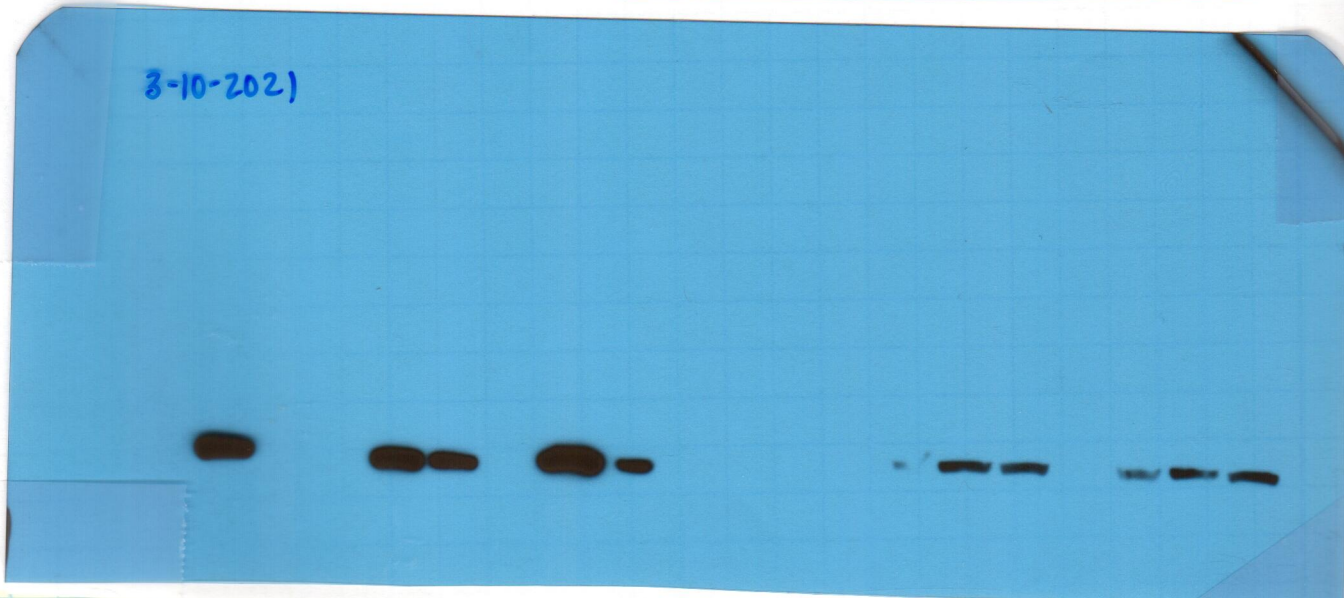

Supplement: Supplementary file 1 [file cancers-13-05130-s001.zip › cancers-1384109-supplementary/cancers-1384109-western blot/ER paper WBs/Western Scans - Lab Notebook 4/WB0010.pdf]

33

Can 925-800:

[illegible]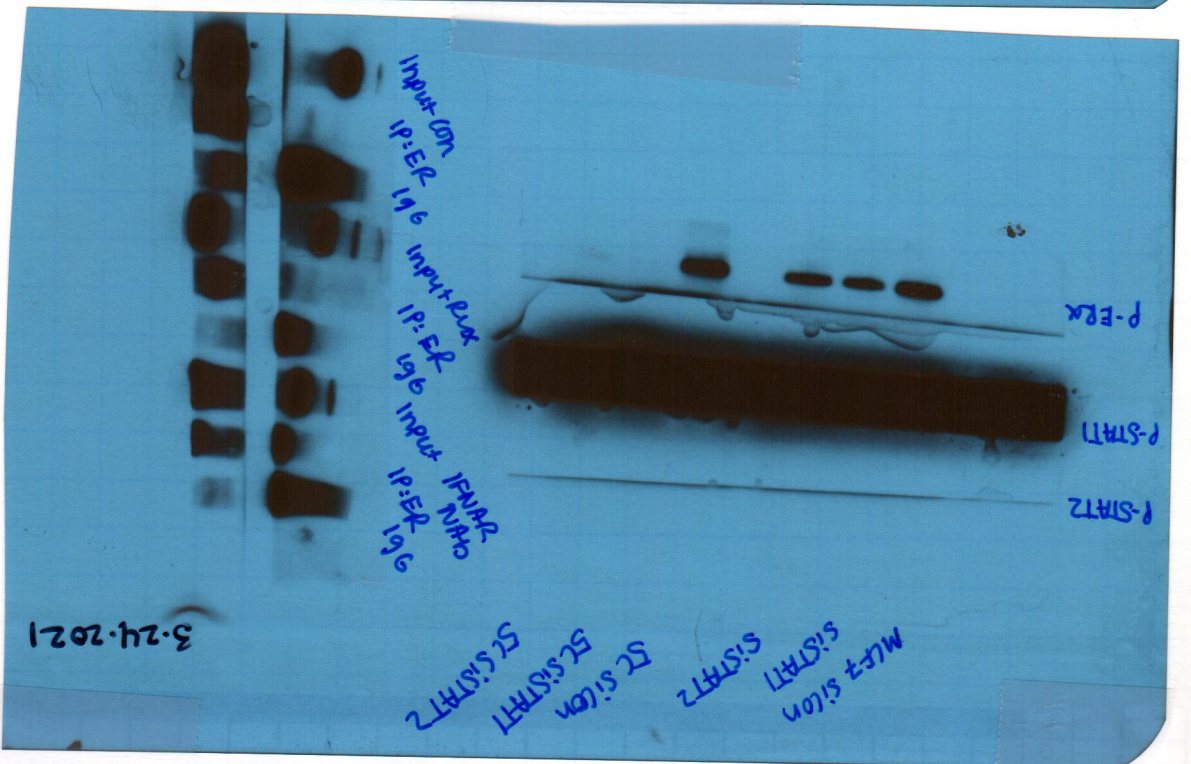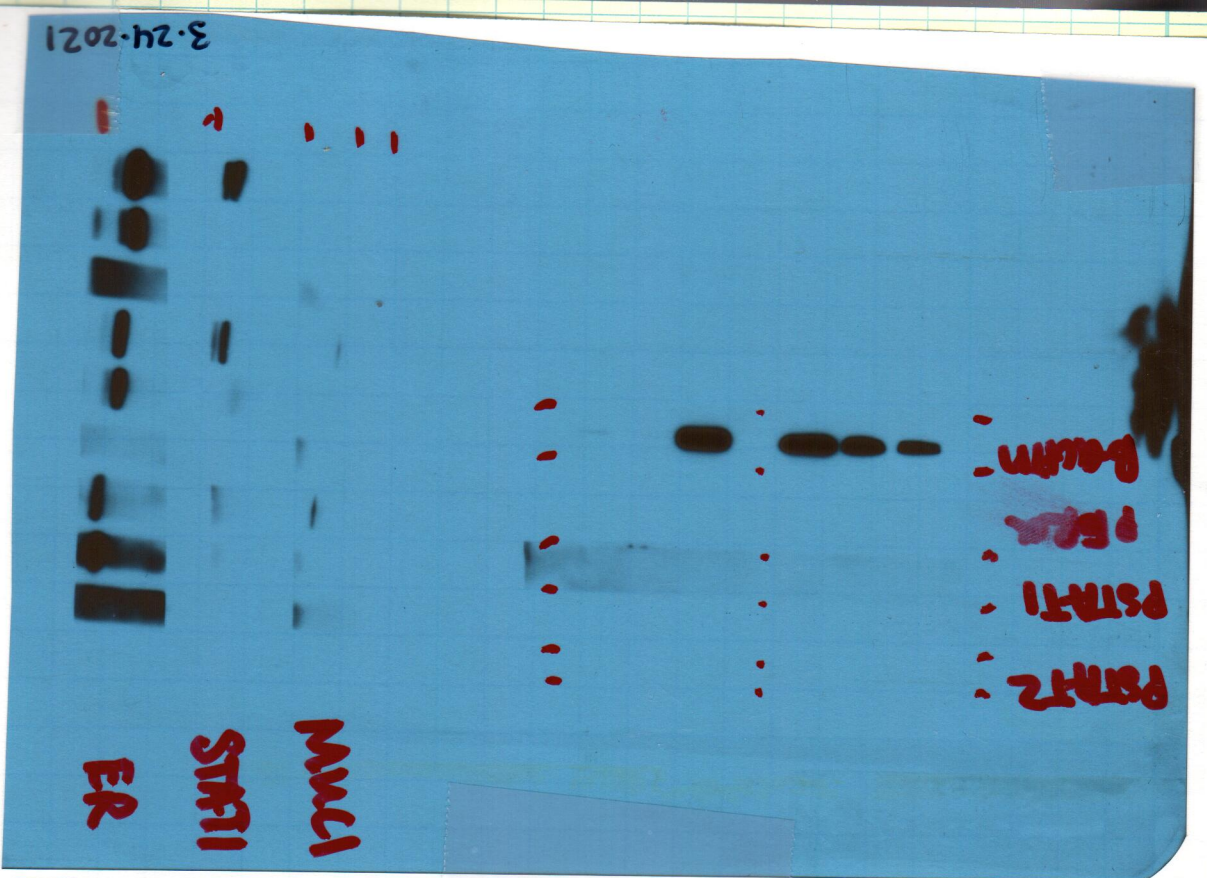

Supplement: Supplementary file 1 [file cancers-13-05130-s001.zip › cancers-1384109-supplementary/cancers-1384109-western blot/ER paper WBs/Western Scans - Lab Notebook 4/WB0012.pdf]

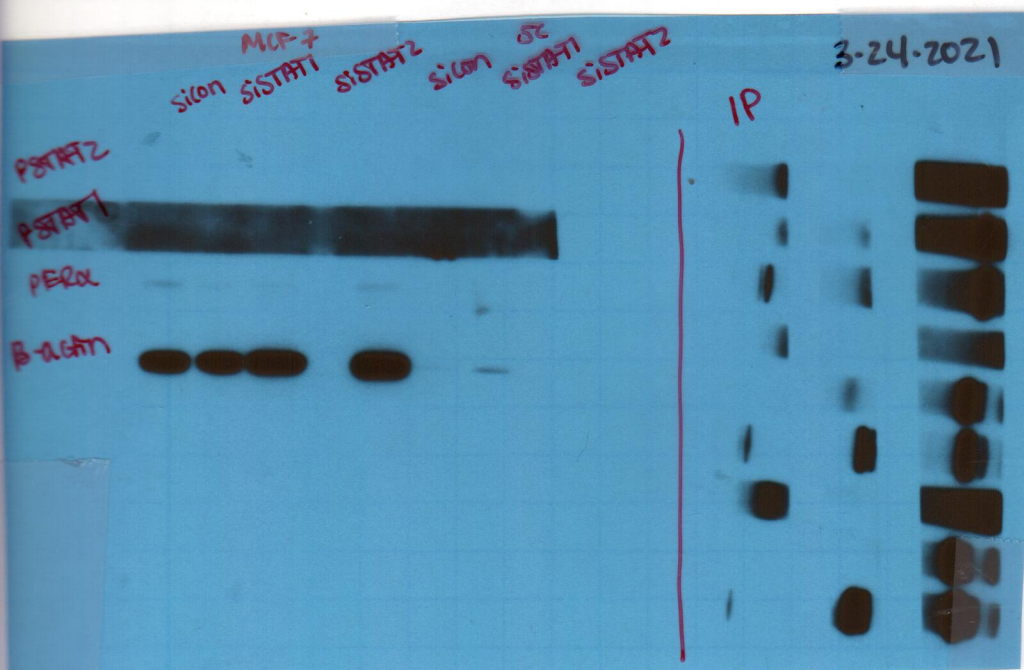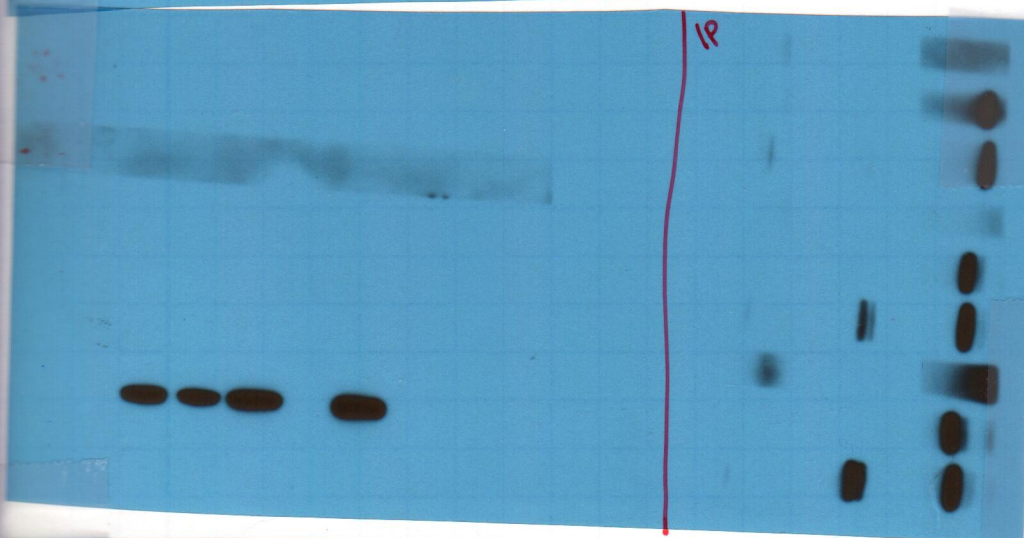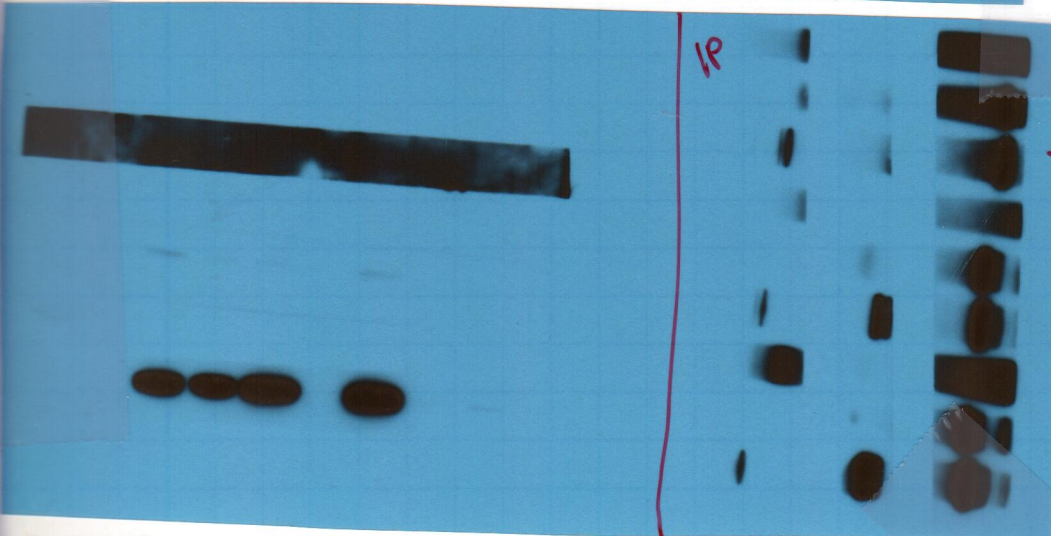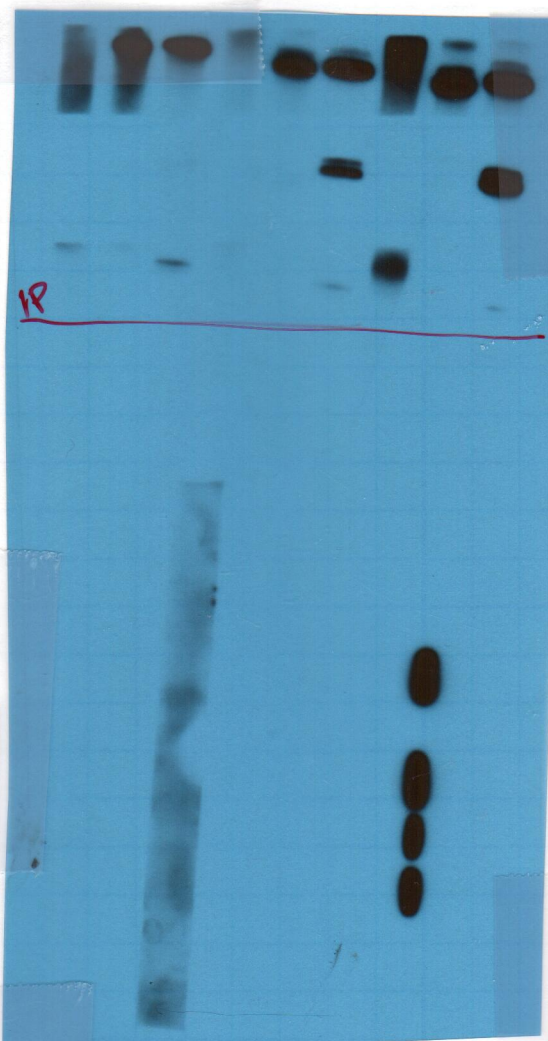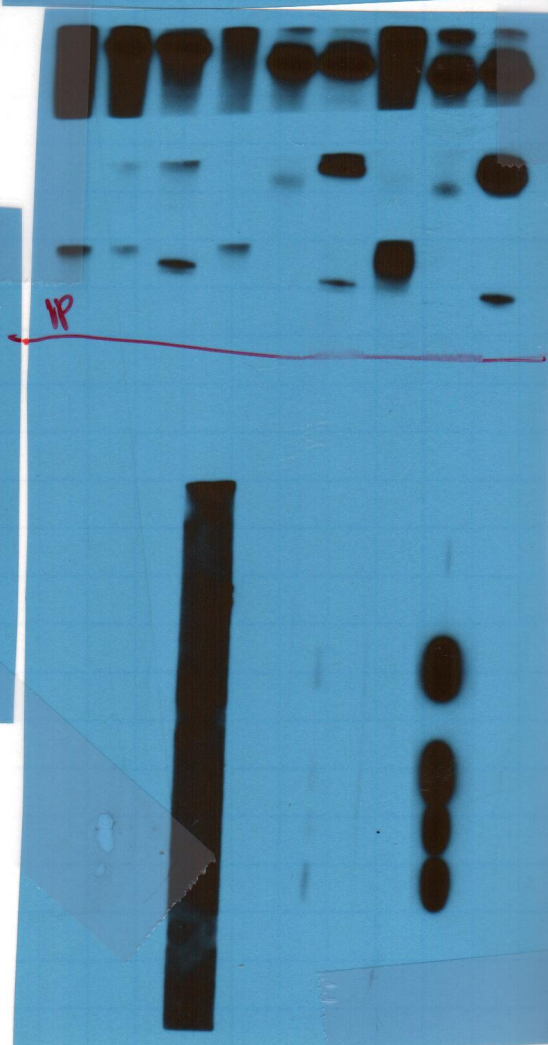

Supplement: Supplementary file 1 [file cancers-13-05130-s001.zip › cancers-1384109-supplementary/cancers-1384109-western blot/ER paper WBs/Western Scans - Lab Notebook 4/WB0013.pdf]

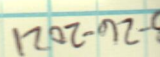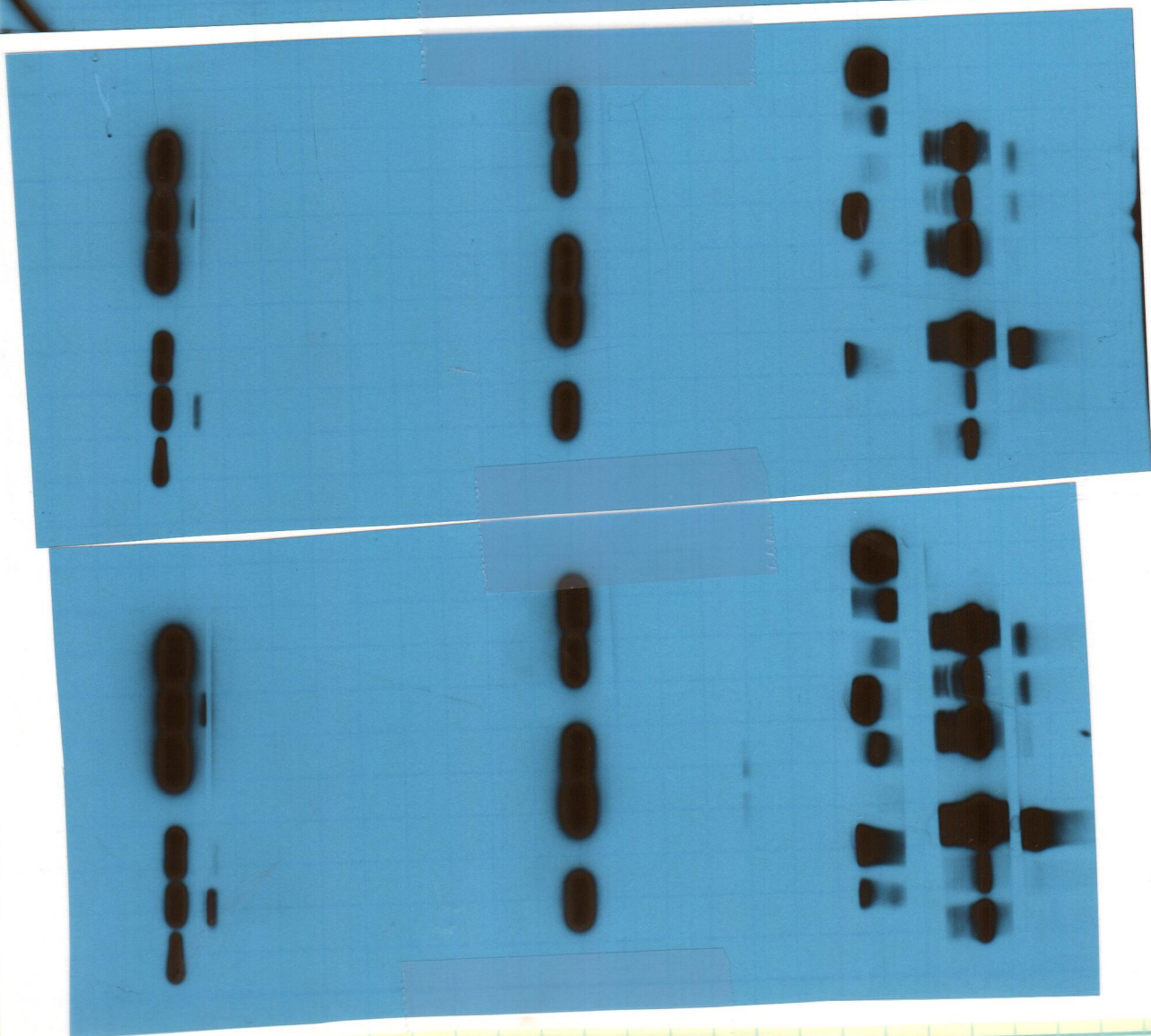

Supplement: Supplementary file 1 [file cancers-13-05130-s001.zip › cancers-1384109-supplementary/cancers-1384109-western blot/ER paper WBs/Western Scans - Lab Notebook 4/WB0015.pdf]

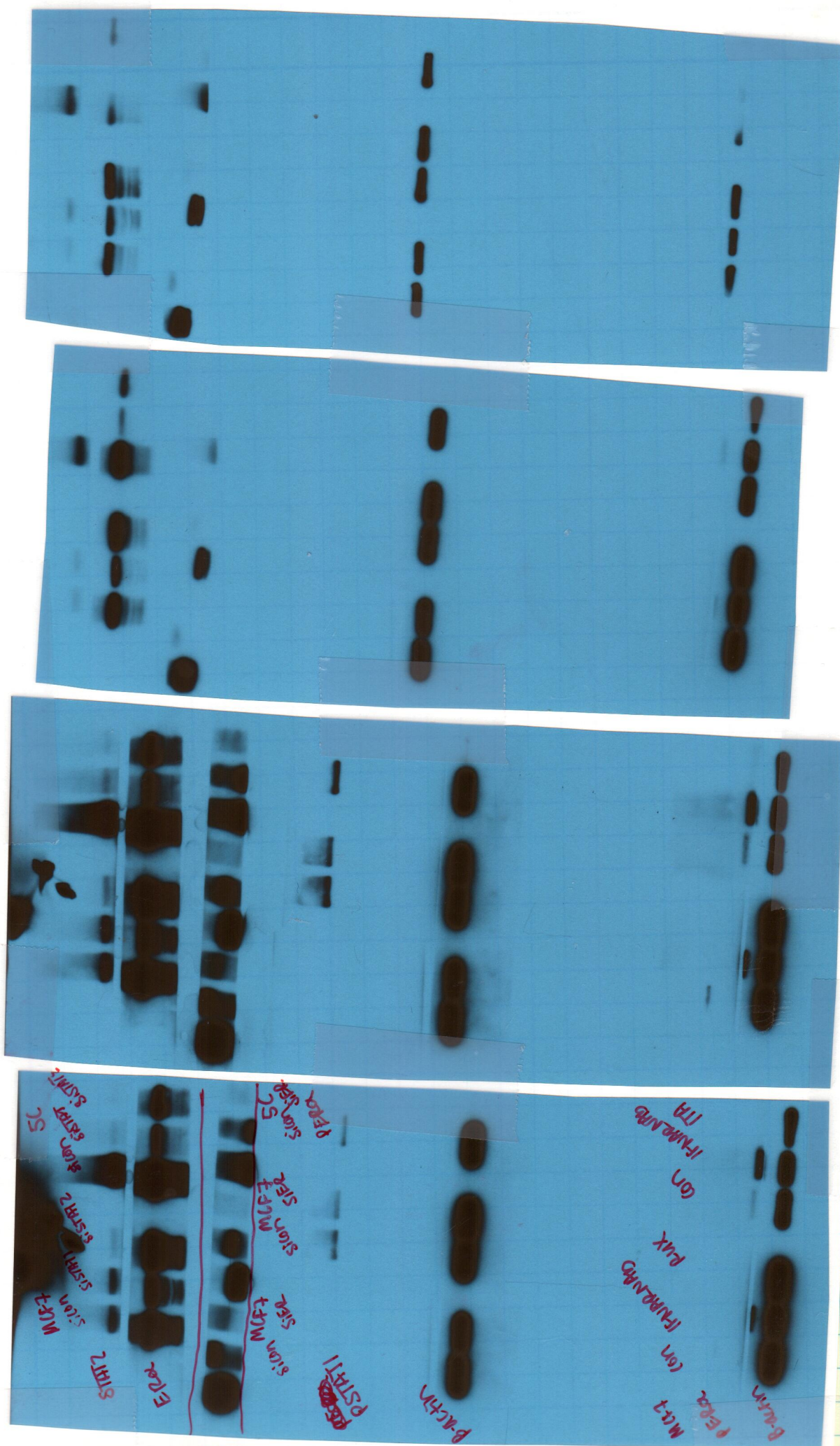

Supplement: Supplementary file 1 [file cancers-13-05130-s001.zip › cancers-1384109-supplementary/cancers-1384109-western blot/ER paper WBs/Western Scans - Lab Notebook 4/WB0016.pdf]

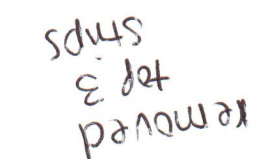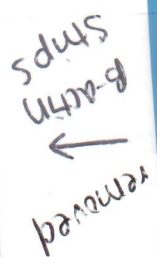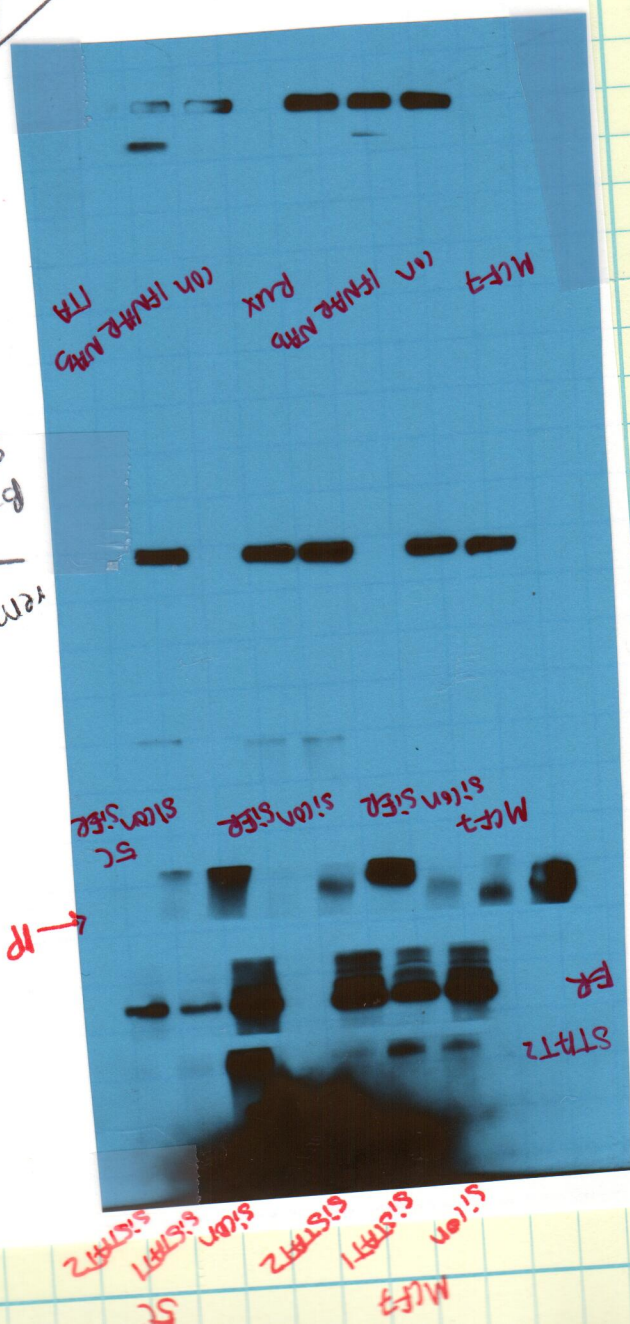

Supplement: Supplementary file 1 [file cancers-13-05130-s001.zip › cancers-1384109-supplementary/cancers-1384109-western blot/ER paper WBs/Western Scans - Lab Notebook 4/WB0017.pdf]

3-28-2021

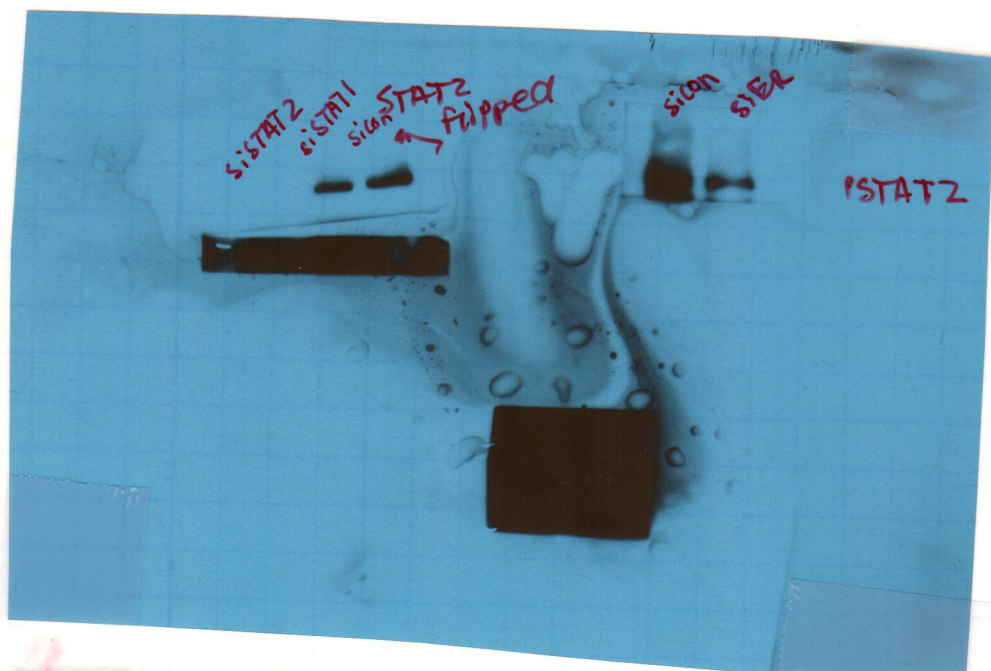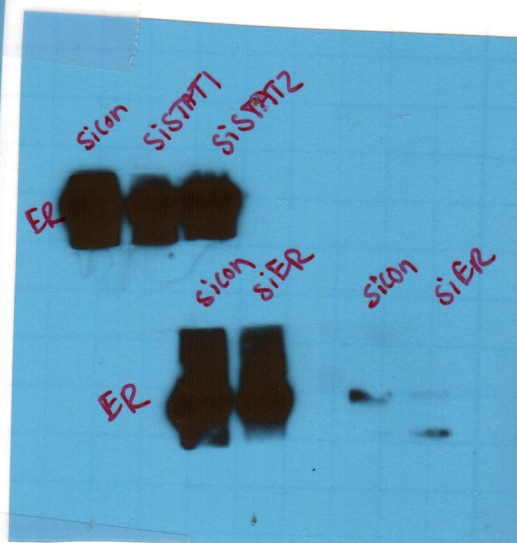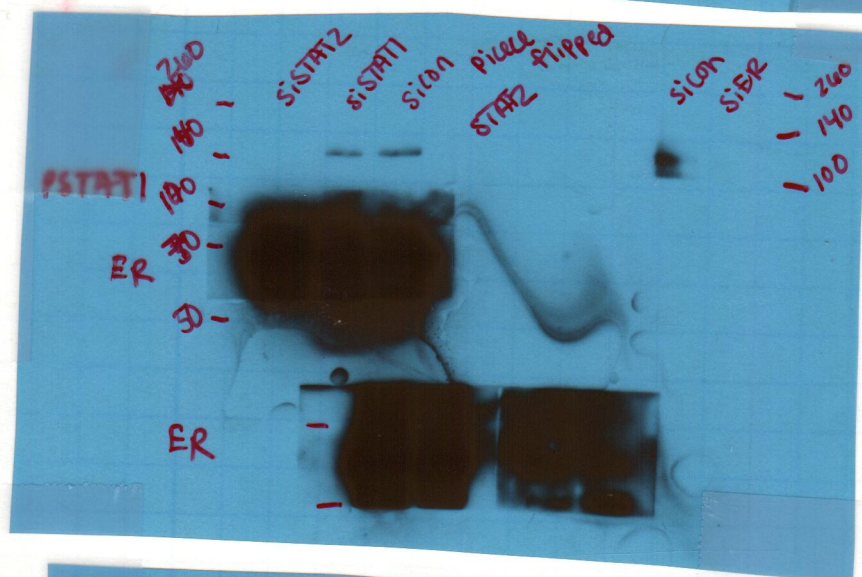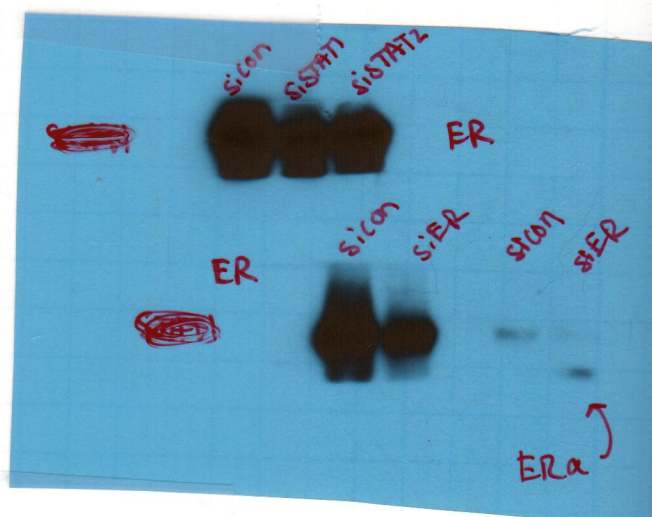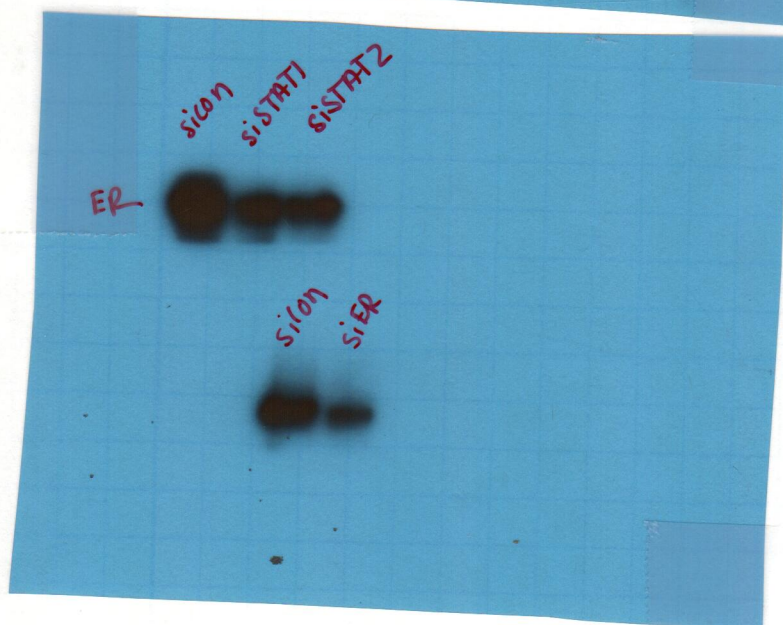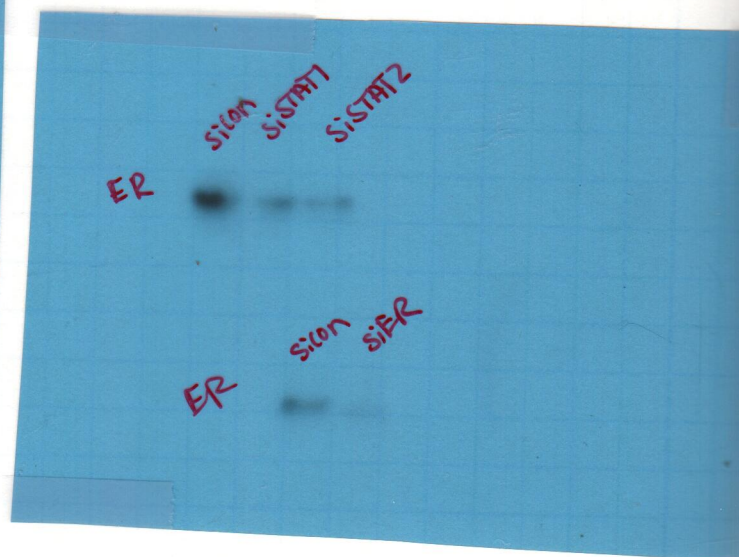

Supplement: Supplementary file 1 [file cancers-13-05130-s001.zip › cancers-1384109-supplementary/cancers-1384109-western blot/ER paper WBs/Western Scans - Lab Notebook 4/WB0018.pdf]

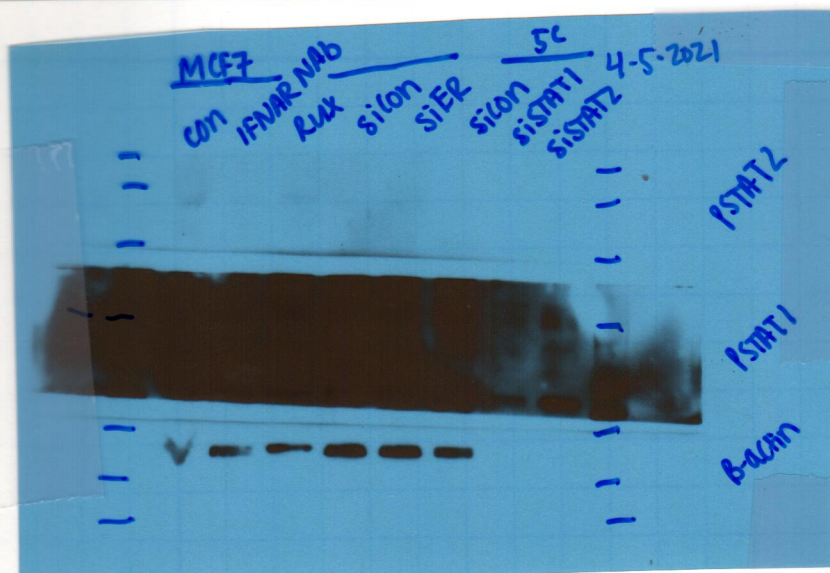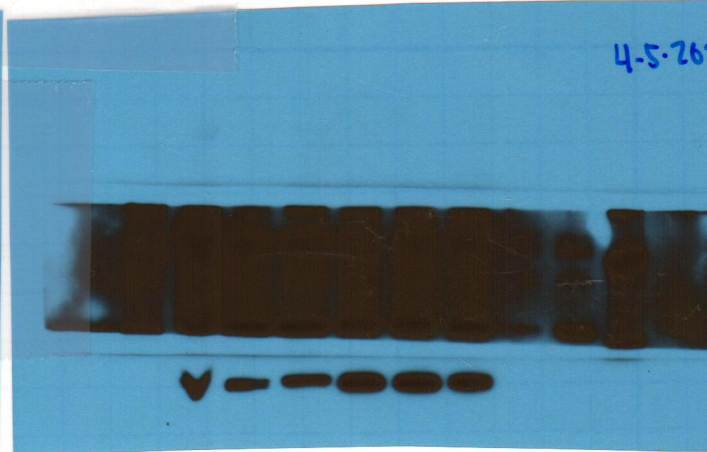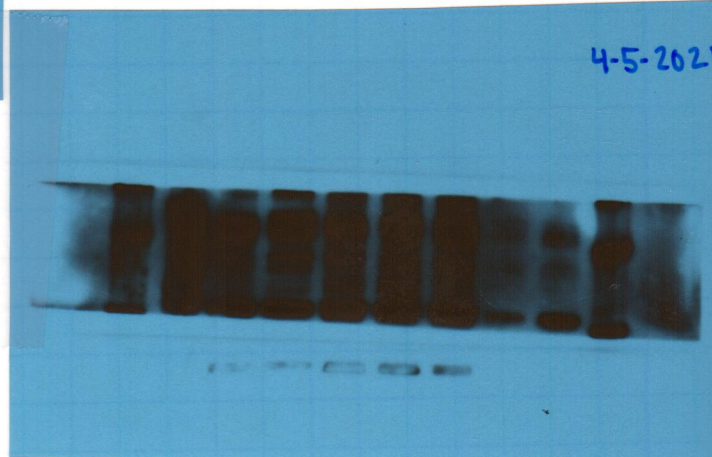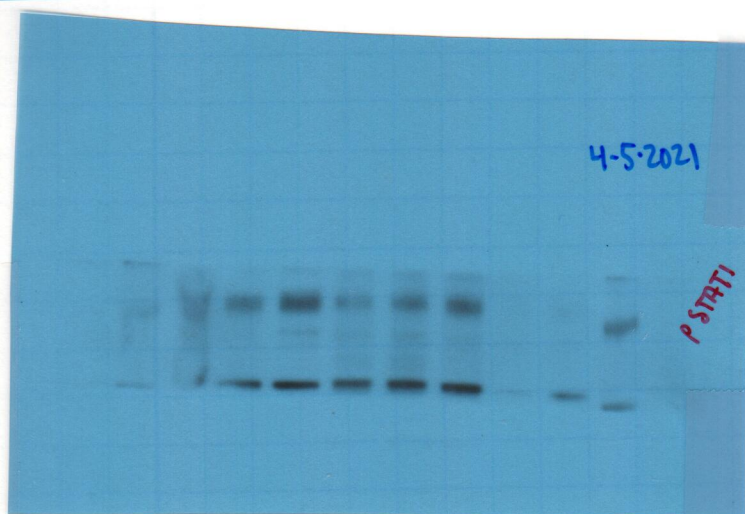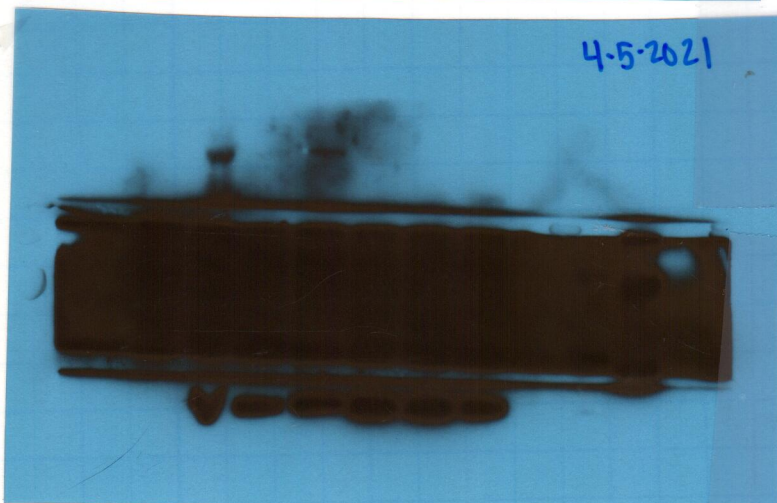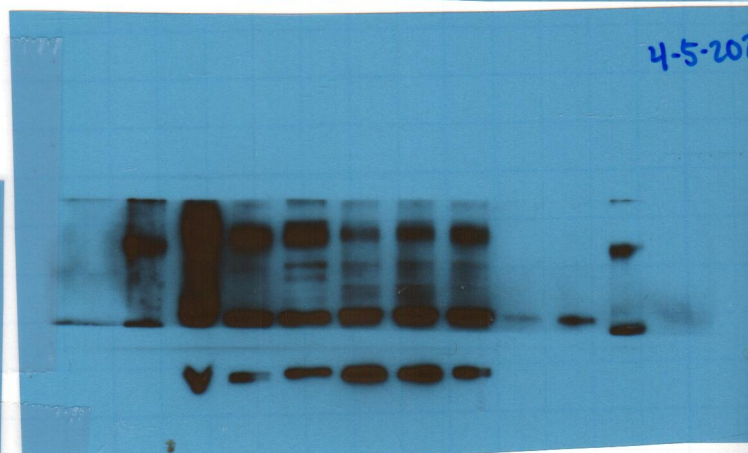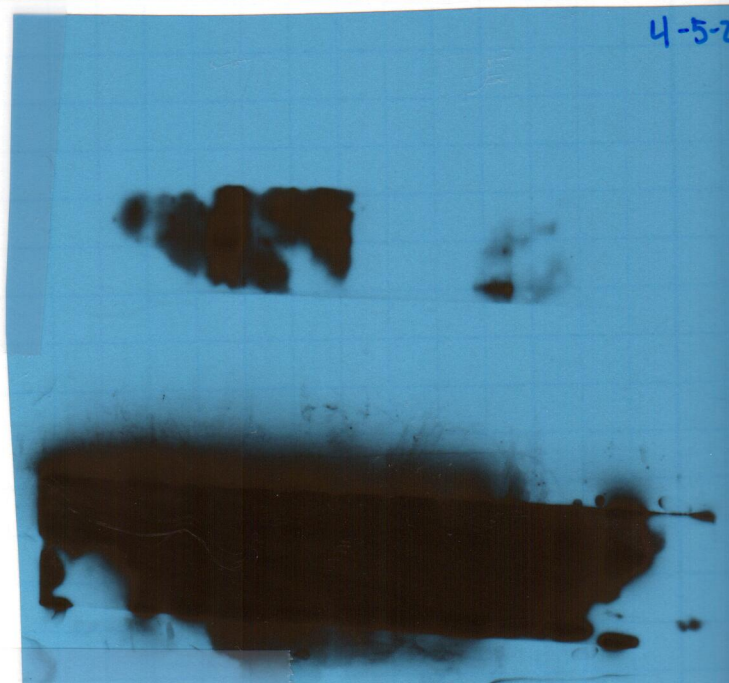

Supplement: Supplementary file 1 [file cancers-13-05130-s001.zip › cancers-1384109-supplementary/cancers-1384109-western blot/ER paper WBs/Western Scans - Lab Notebook 4/WB0019.pdf]

ordered ice /  
chip supplies / need  
other than kit.

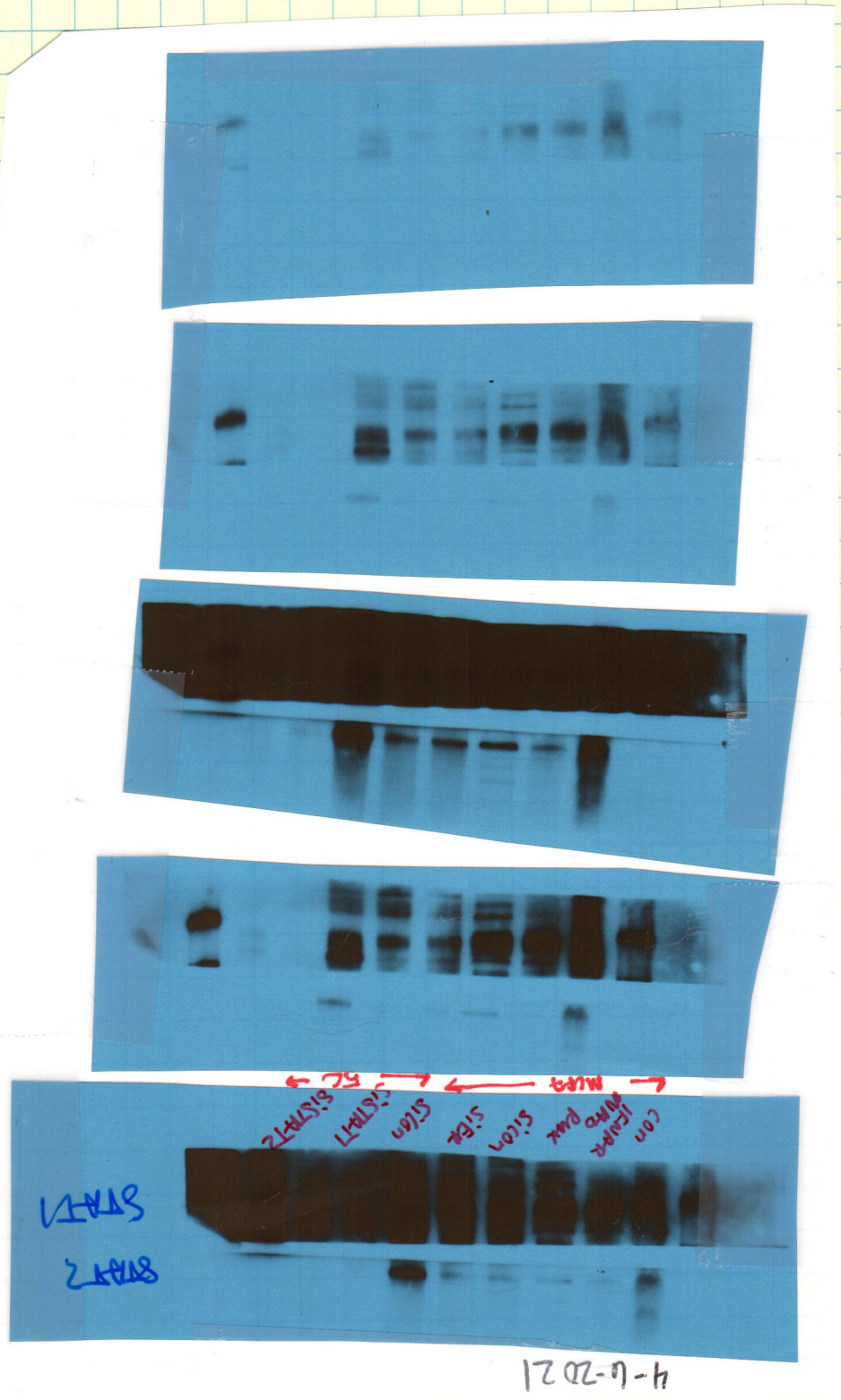

Supplement: Supplementary file 1 [file cancers-13-05130-s001.zip › cancers-1384109-supplementary/cancers-1384109-western blot/ER paper WBs/Western Scans - Lab Notebook 4/WB0020.pdf]

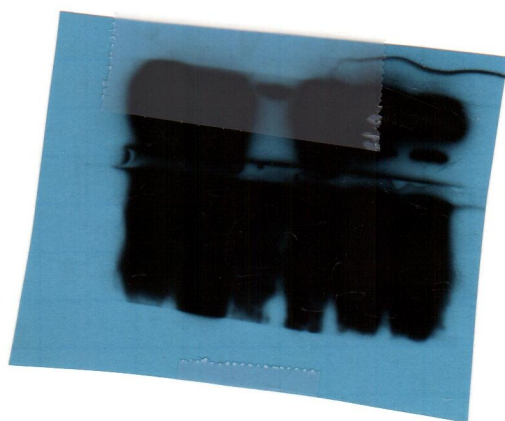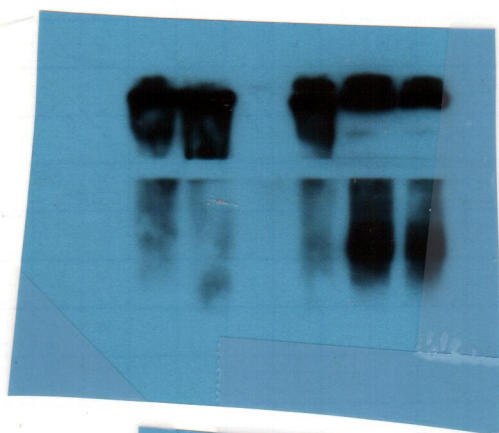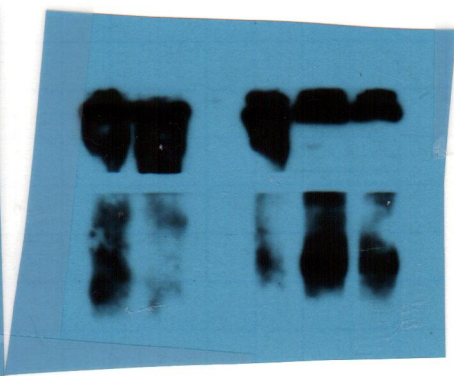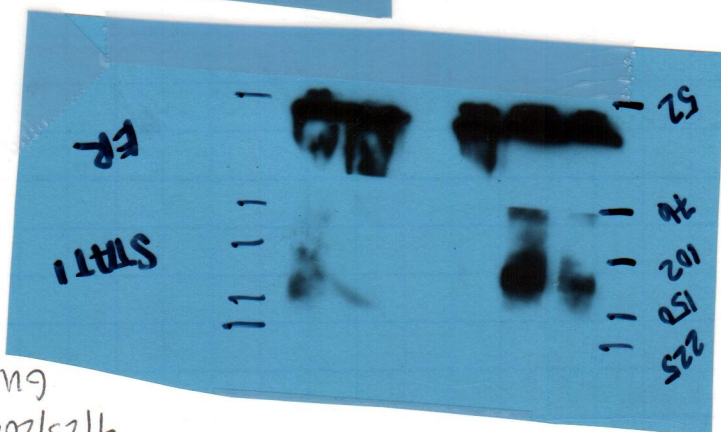

4/23/2021  
Gustavo  
bat

Supplement: Supplementary file 1 [file cancers-13-05130-s001.zip › cancers-1384109-supplementary/cancers-1384109-western blot/ER paper WBs/Western Scans - Lab Notebook 4/WB0022.pdf]

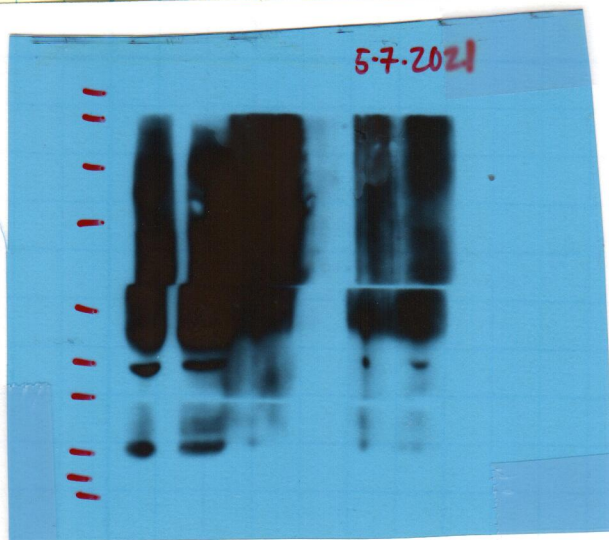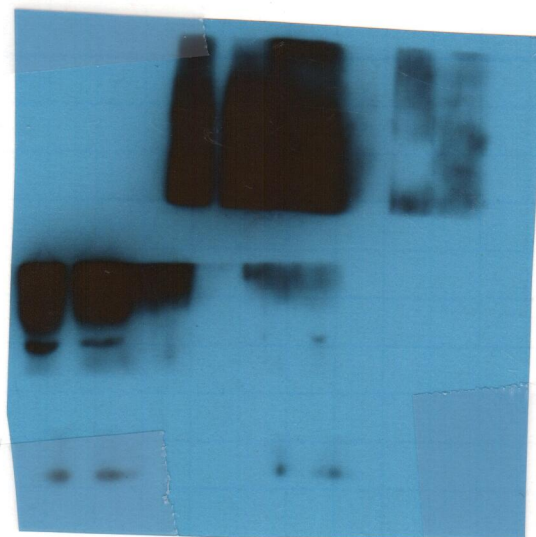

Gustavo  
blot

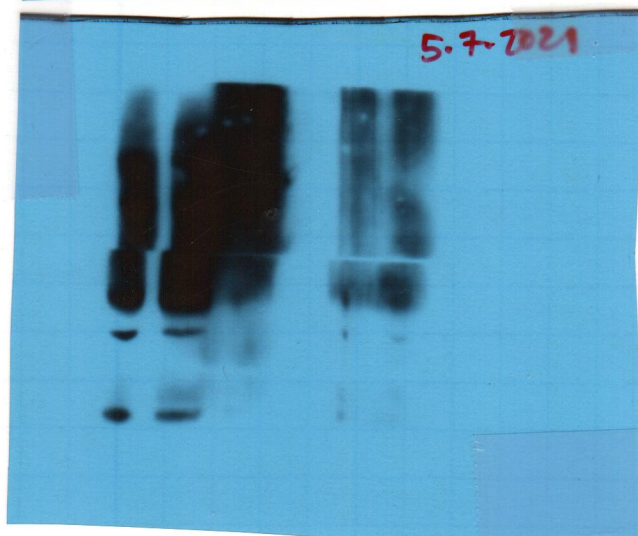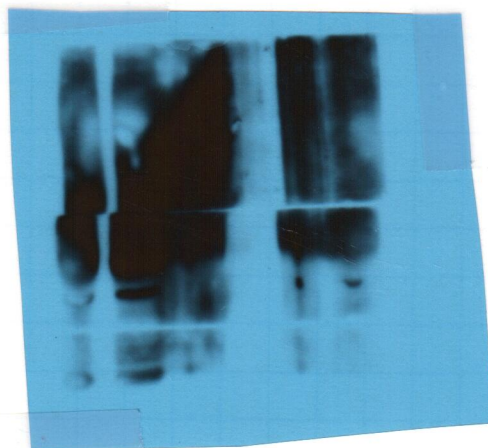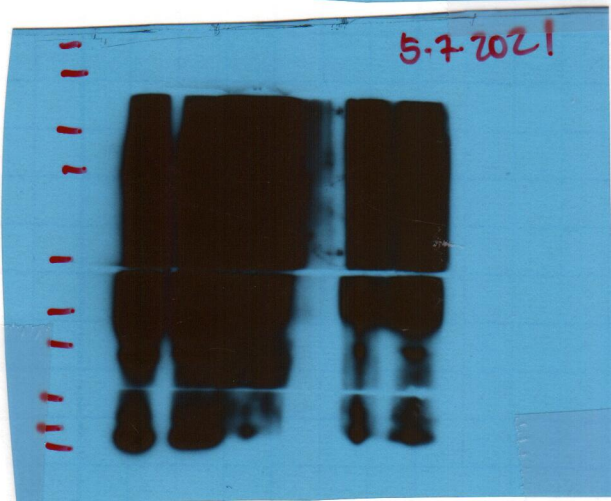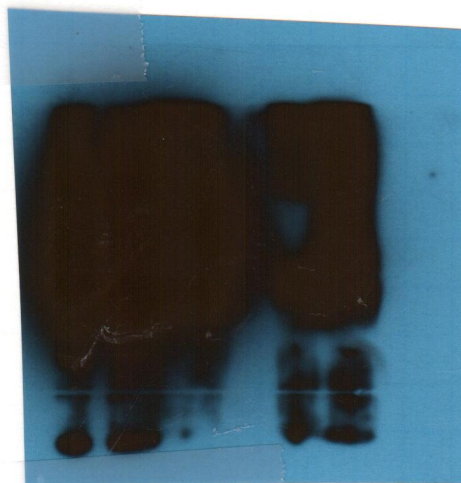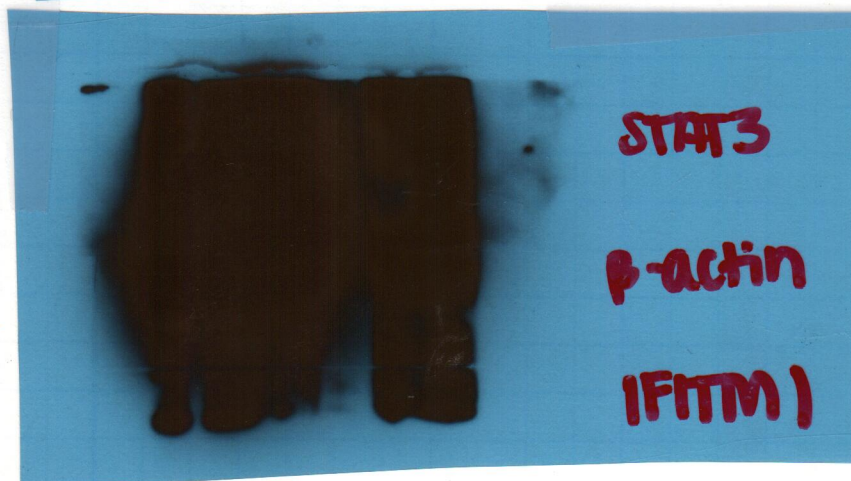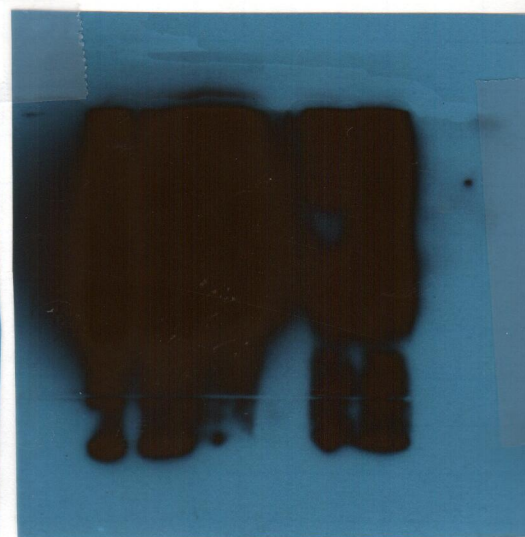

Supplement: Supplementary file 1 [file cancers-13-05130-s001.zip › cancers-1384109-supplementary/cancers-1384109-western blot/ER paper WBs/Western Scans - Lab Notebook 4/WB0023.pdf]

5-12-2021

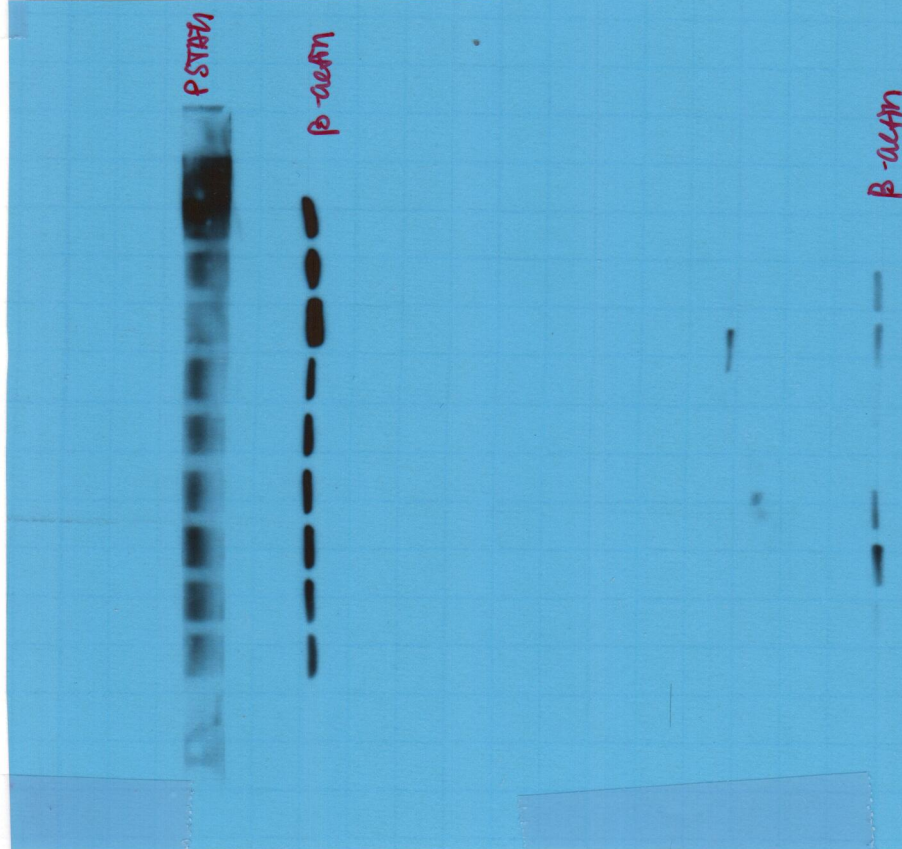

5-12-2021

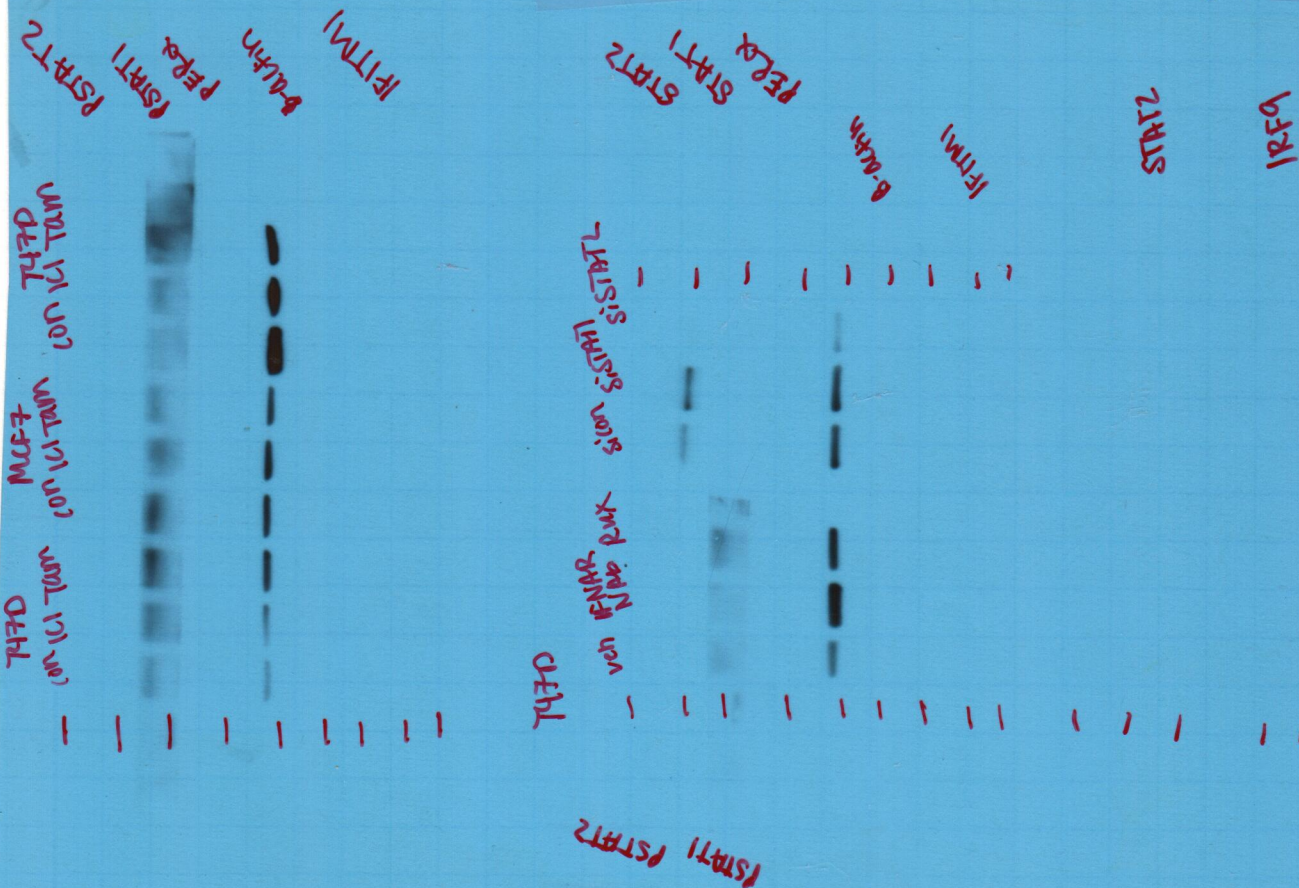

Supplement: Supplementary file 1 [file cancers-13-05130-s001.zip › cancers-1384109-supplementary/cancers-1384109-western blot/ER paper WBs/Western Scans - Lab Notebook 4/WB0025.pdf]

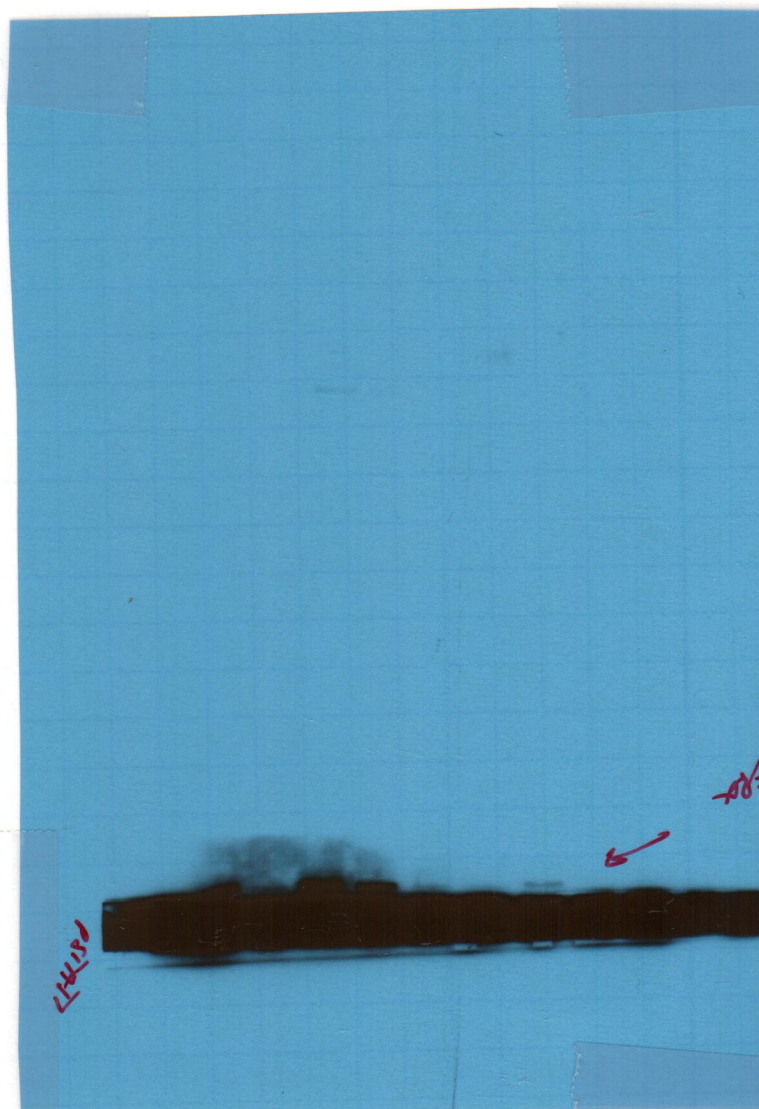

Supplement: Supplementary file 1 [file cancers-13-05130-s001.zip › cancers-1384109-supplementary/cancers-1384109-western blot/ER paper WBs/Western Scans - Lab Notebook 4/WB0026.pdf]

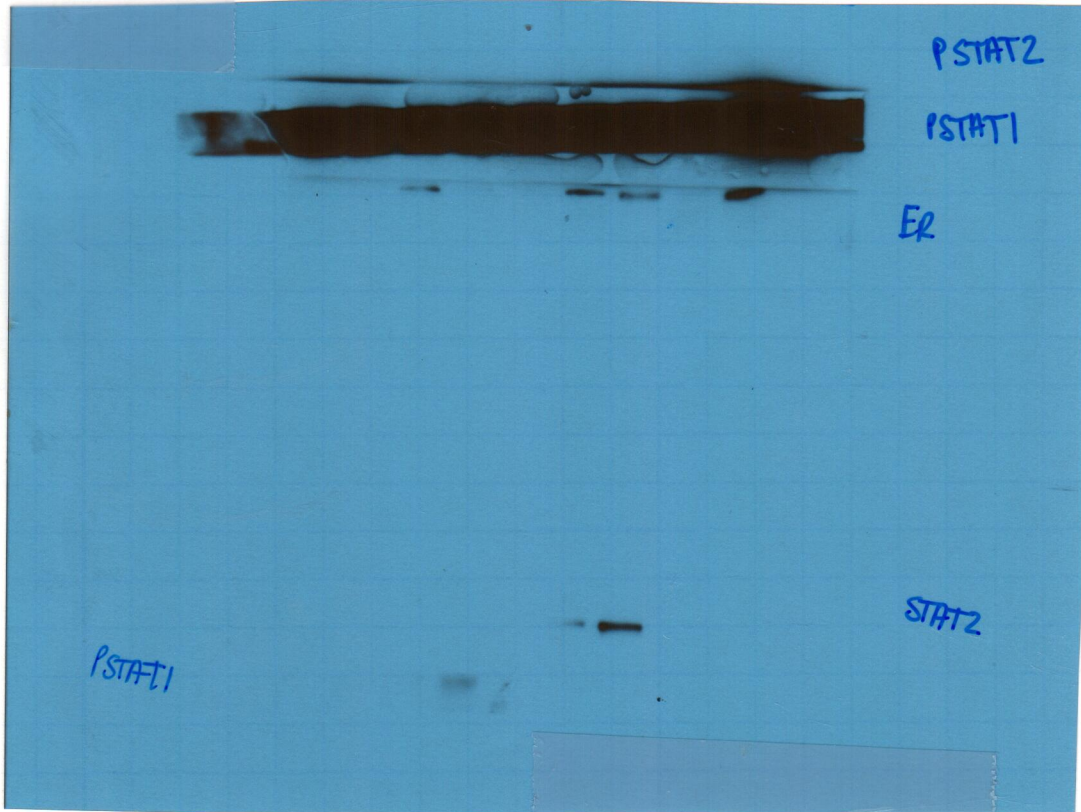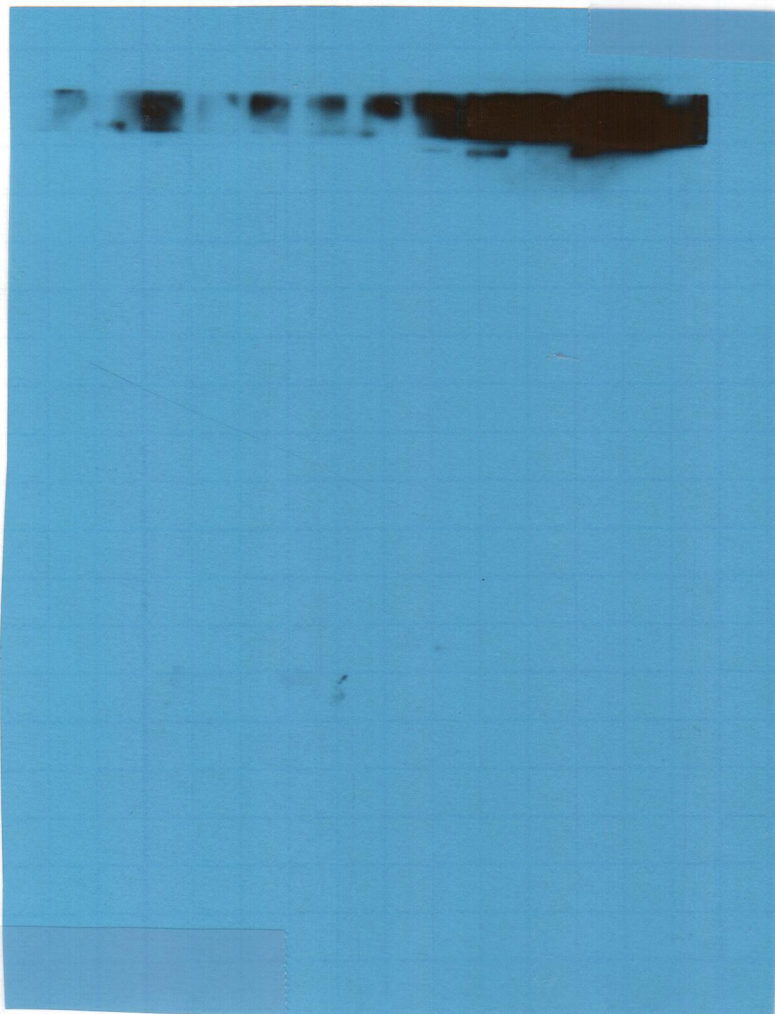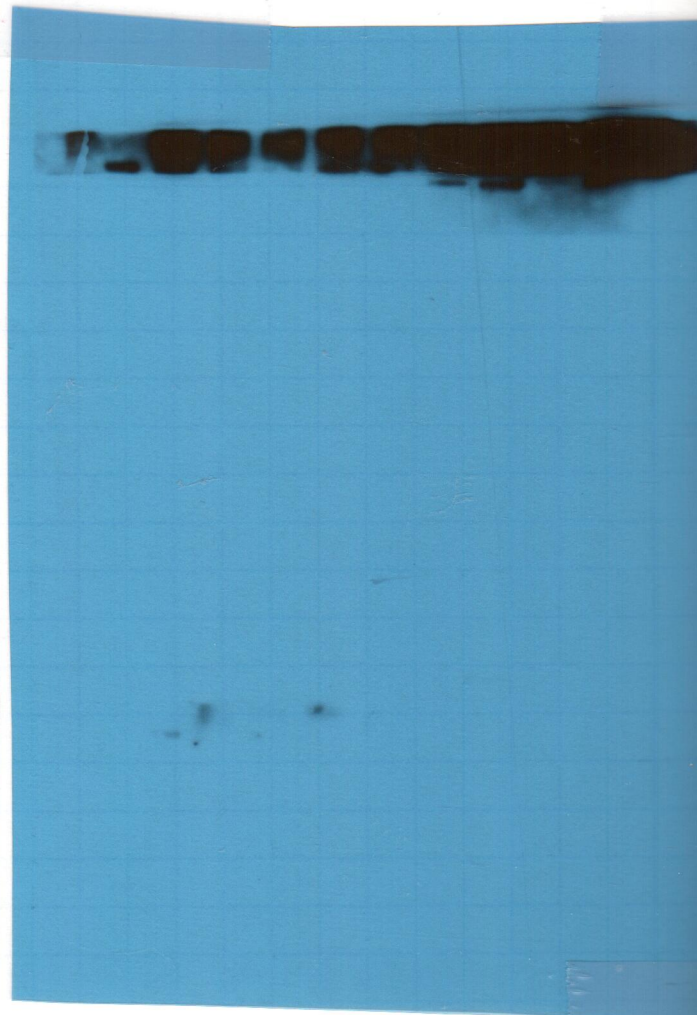

Supplement: Supplementary file 1 [file cancers-13-05130-s001.zip › cancers-1384109-supplementary/cancers-1384109-western blot/ER paper WBs/Western Scans - Lab Notebook 4/WB0027.pdf]

5-14-2021

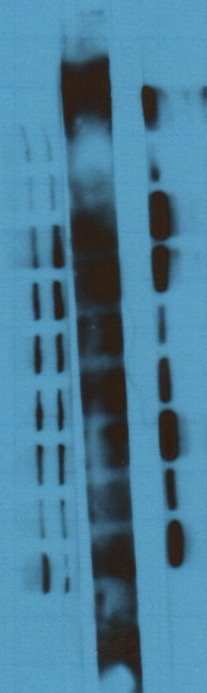

5-14-2021

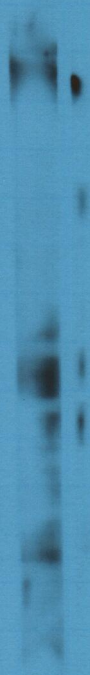

5-14-2021

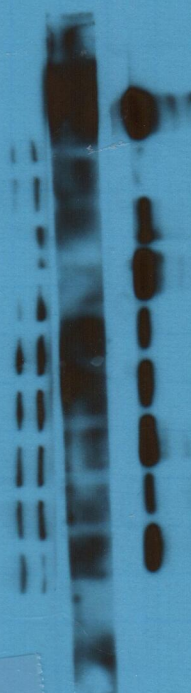

Supplement: Supplementary file 1 [file cancers-13-05130-s001.zip › cancers-1384109-supplementary/cancers-1384109-western blot/ER paper WBs/Western Scans - Lab Notebook 4/WB0029.pdf]

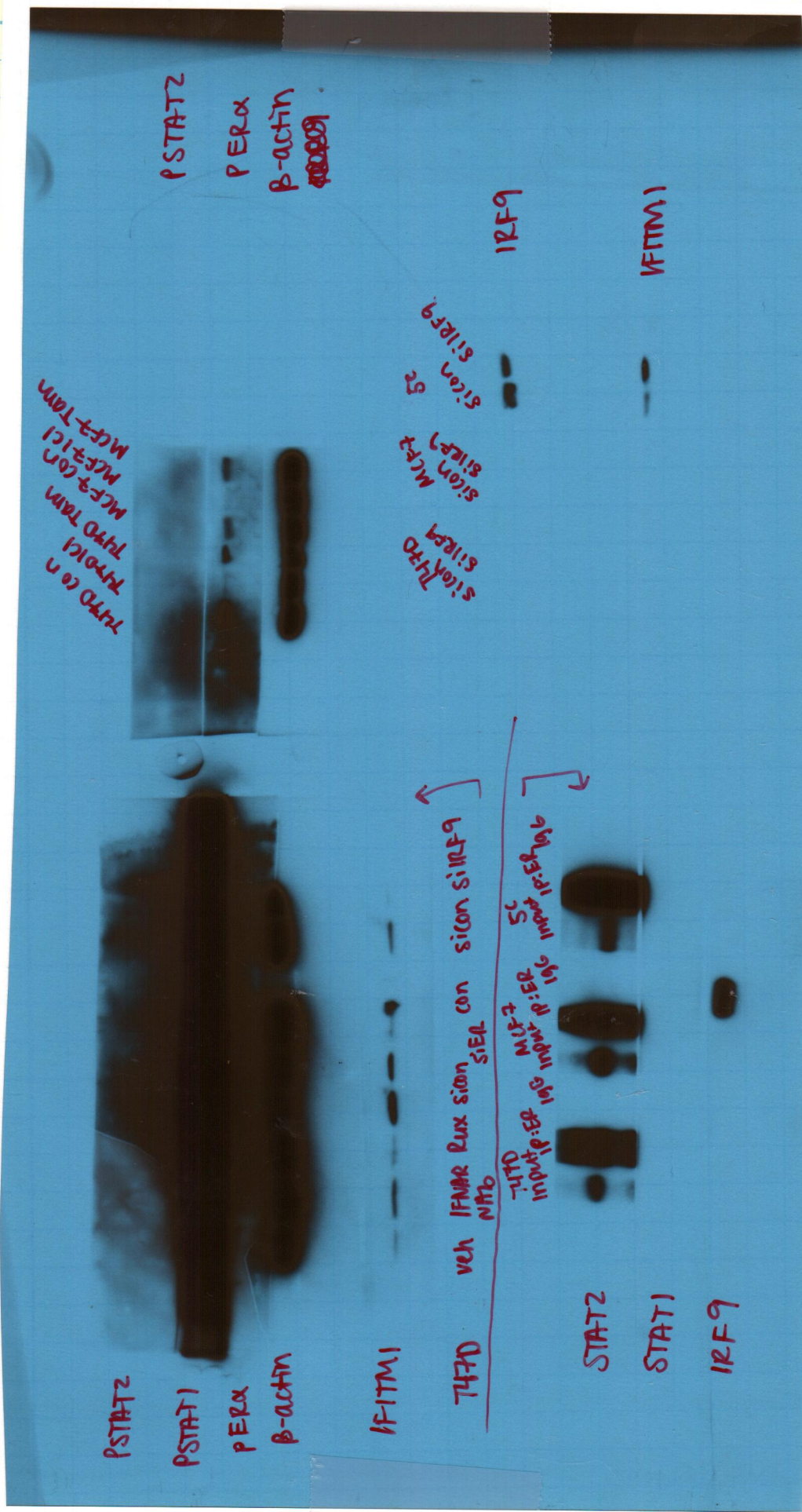

Supplement: Supplementary file 1 [file cancers-13-05130-s001.zip › cancers-1384109-supplementary/cancers-1384109-western blot/ER paper WBs/Western Scans - Lab Notebook 4/WB0031.pdf]

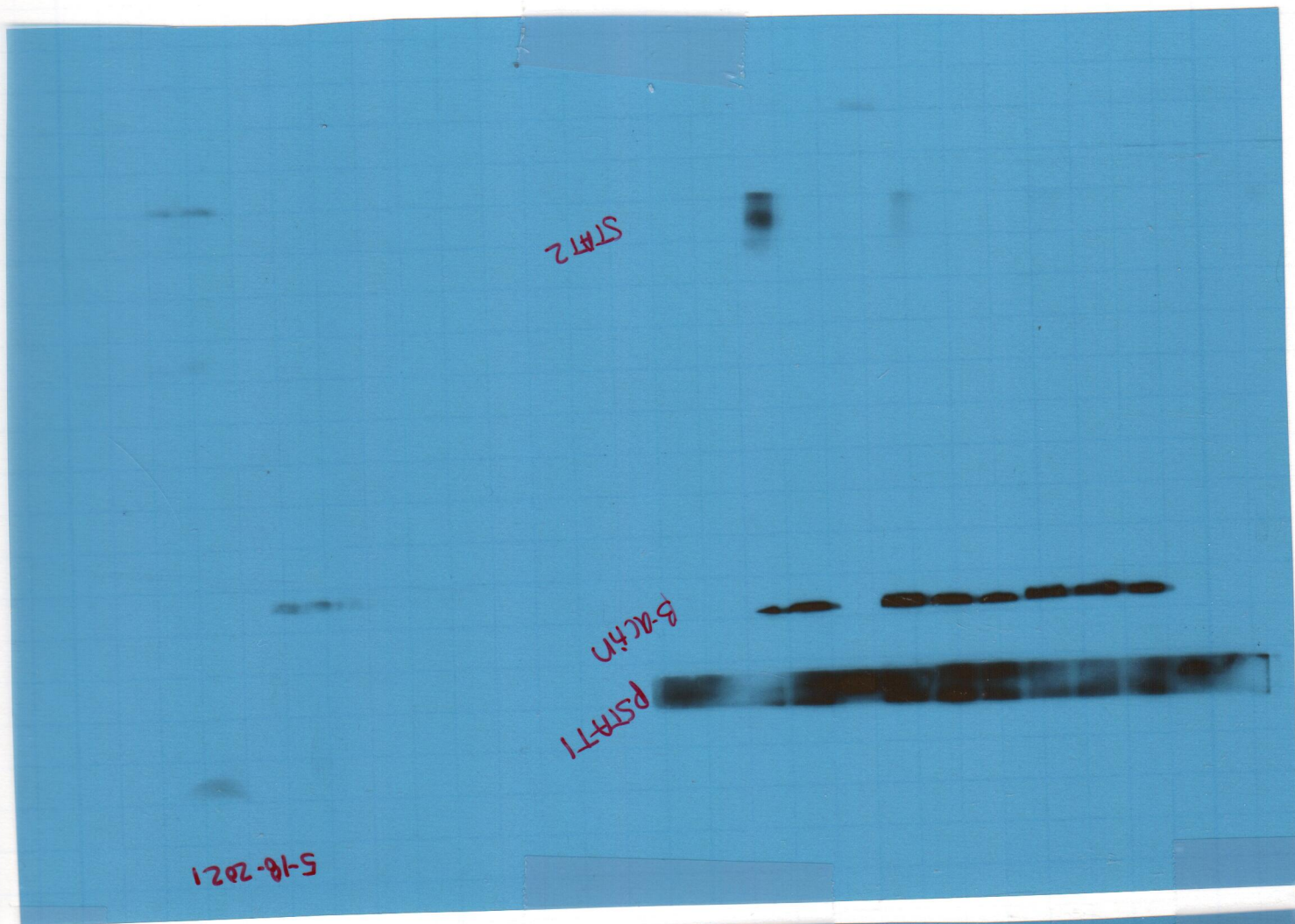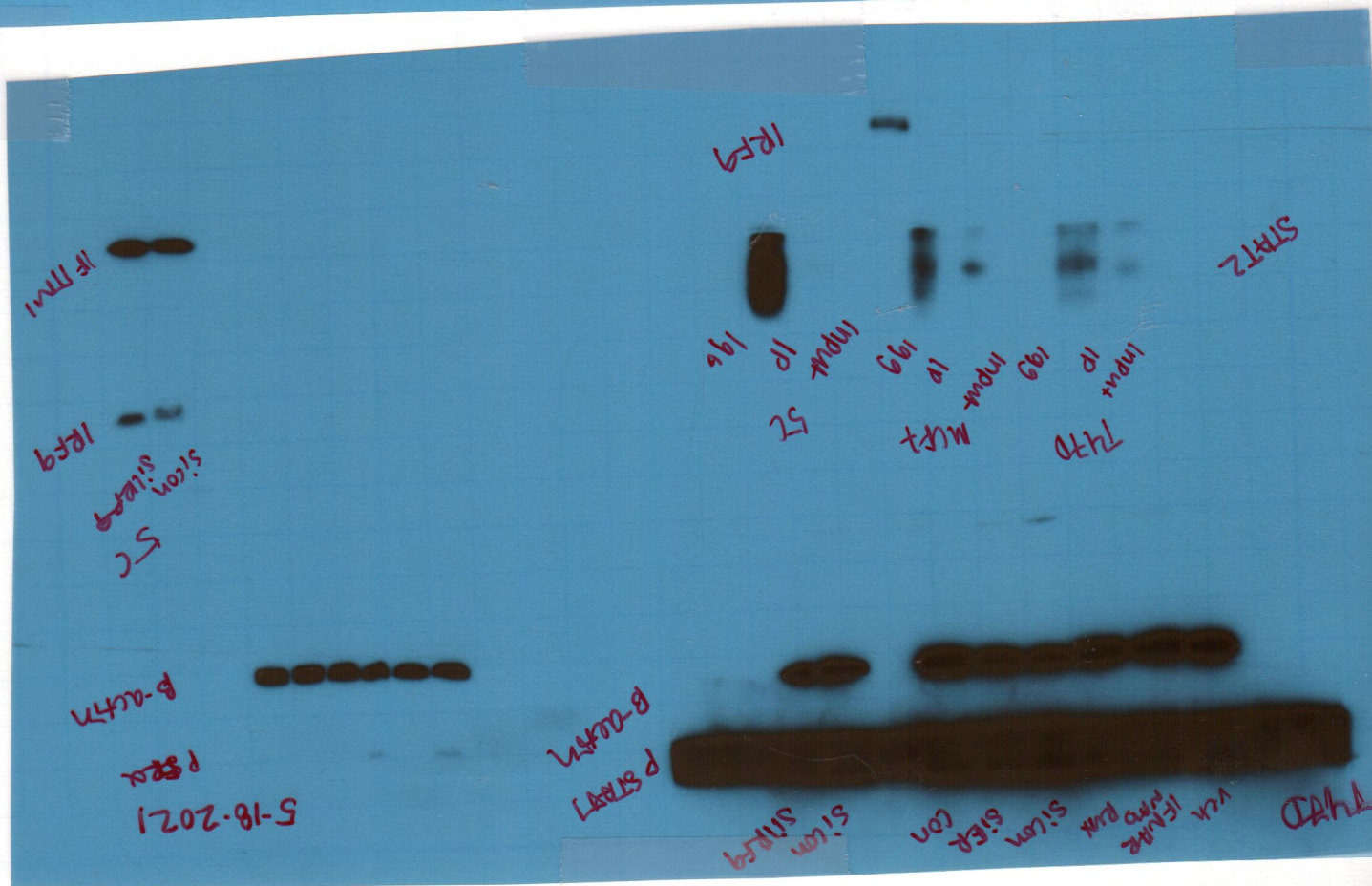

Supplement: Supplementary file 1 [file cancers-13-05130-s001.zip › cancers-1384109-supplementary/cancers-1384109-western blot/ER paper WBs/Western Scans - Lab Notebook 4/WB0032.pdf]

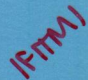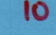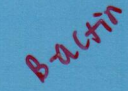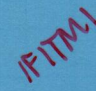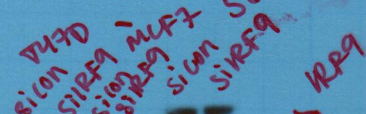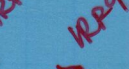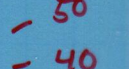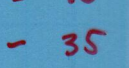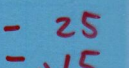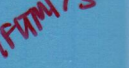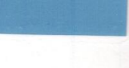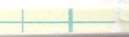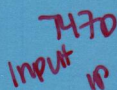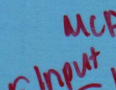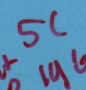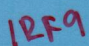

5-18-2021

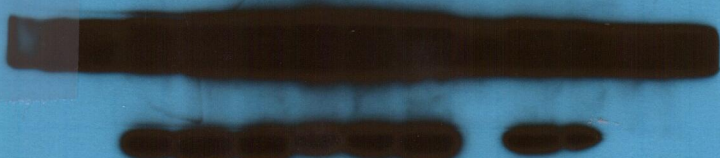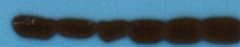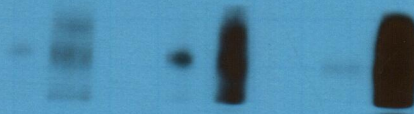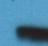

Supplement: Supplementary file 1 [file cancers-13-05130-s001.zip › cancers-1384109-supplementary/cancers-1384109-western blot/ER paper WBs/Western Scans - Lab Notebook 4/WB0033.pdf]

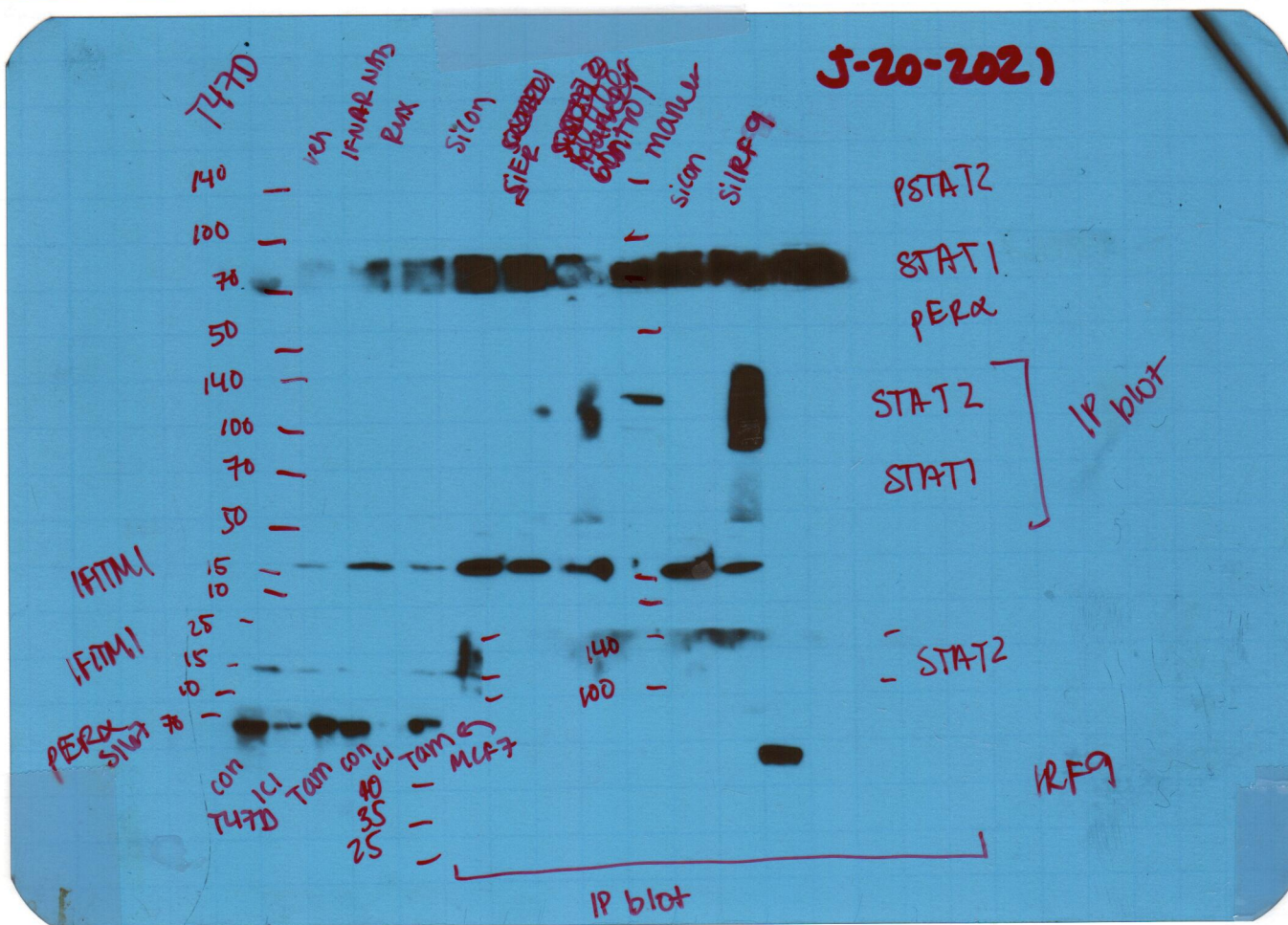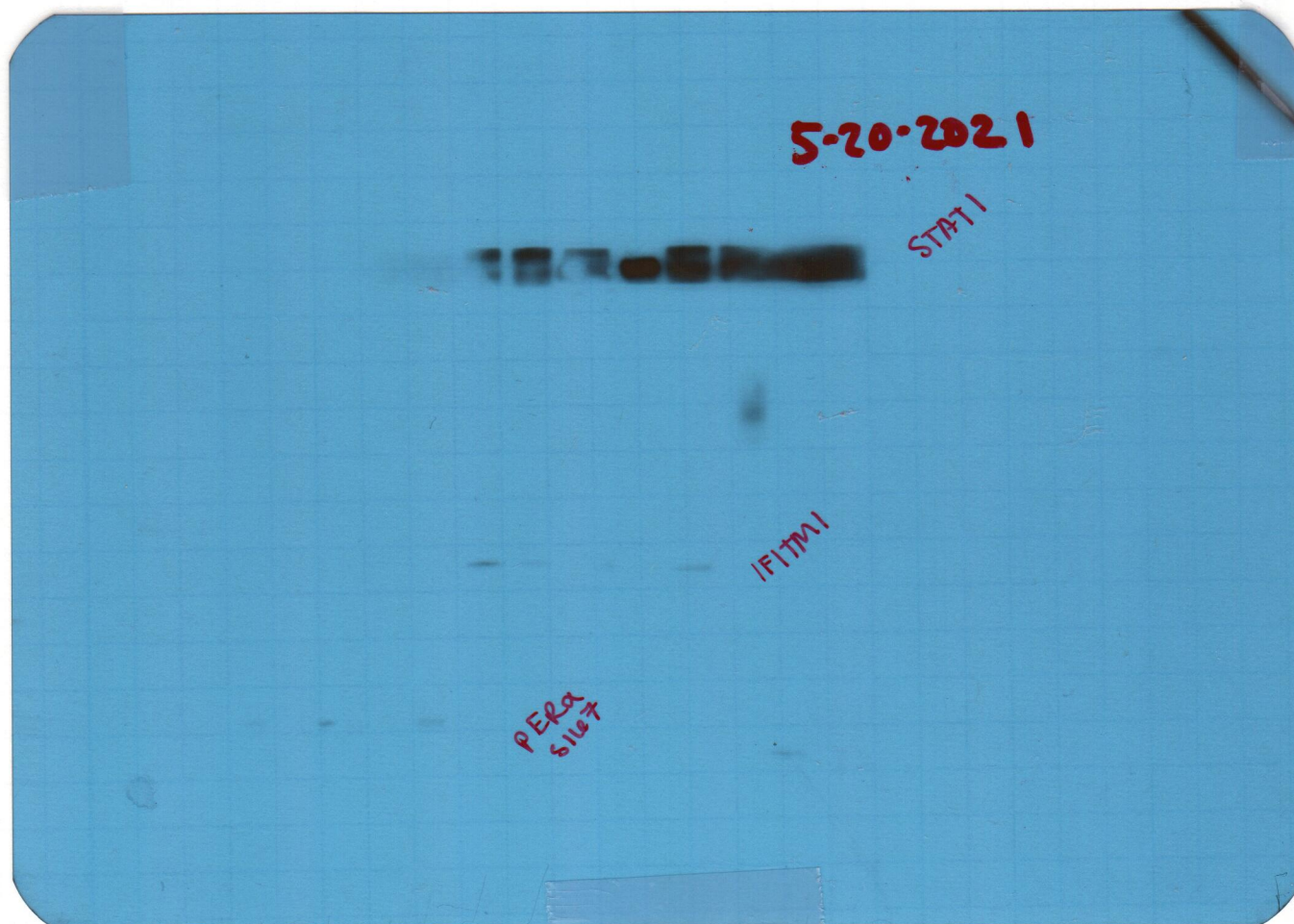

Supplement: Supplementary file 1 [file cancers-13-05130-s001.zip › cancers-1384109-supplementary/cancers-1384109-western blot/ER paper WBs/Western Scans - Lab Notebook 4/WB0034.pdf]

IP:  
ERInput TH7D  
IP

IgG

Input MCF7  
IP IgGInput 5C  
IP IgG

STAT2

STAT1

ER

IRS9

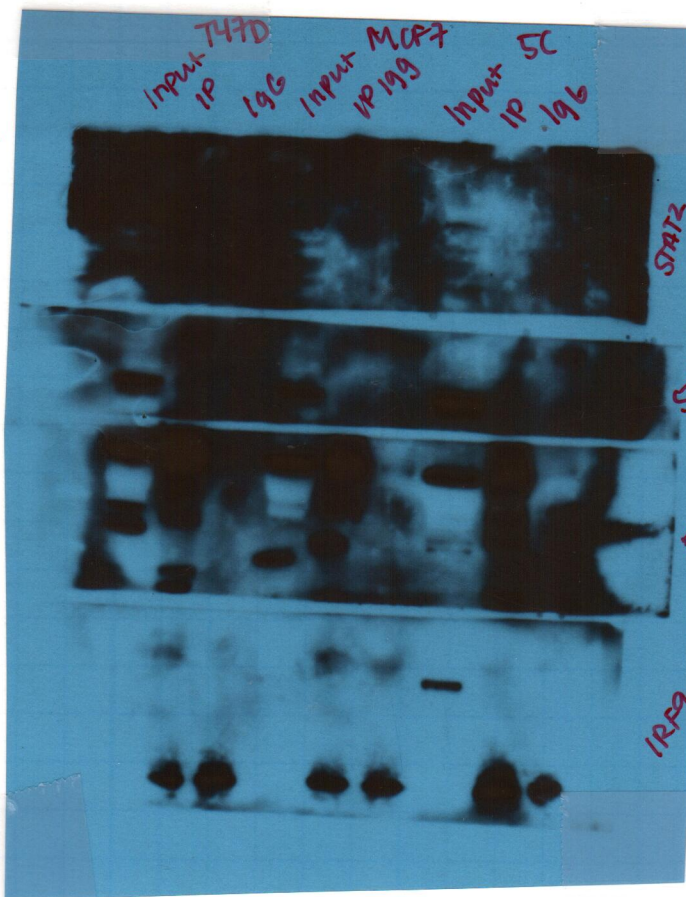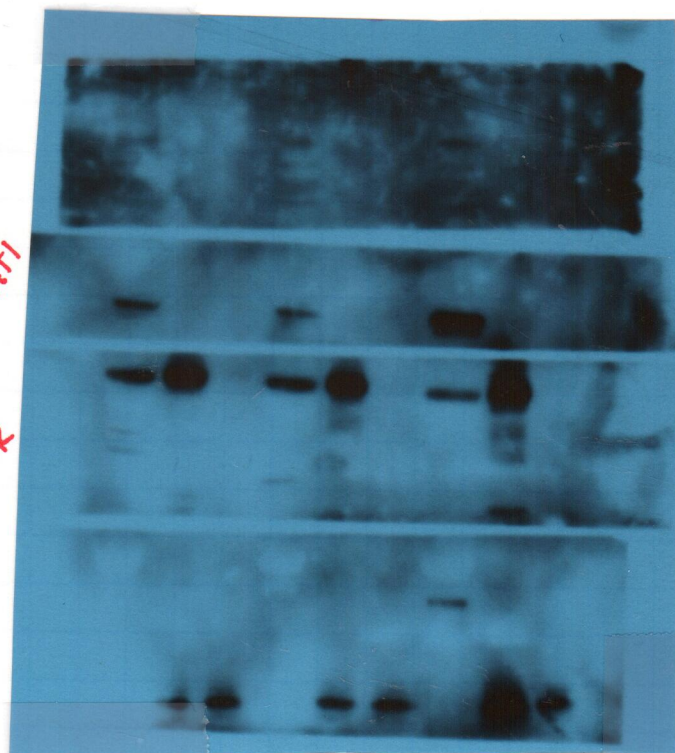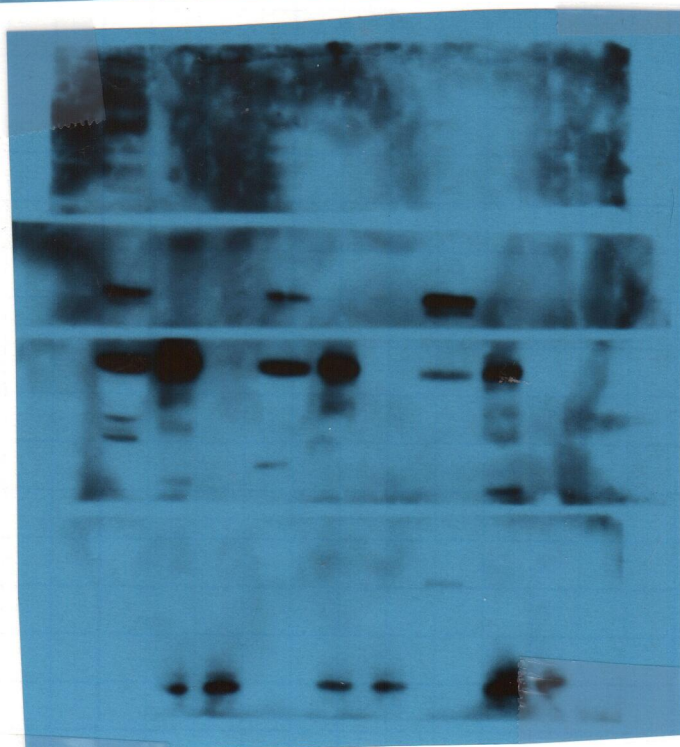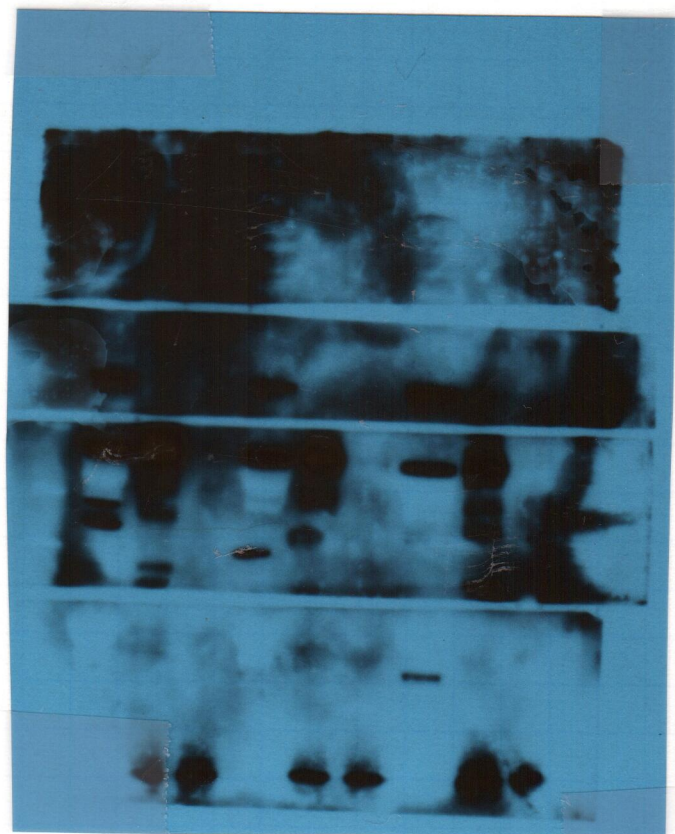

Supplement: Supplementary file 1 [file cancers-13-05130-s001.zip › cancers-1384109-supplementary/cancers-1384109-western blot/ER paper WBs/Western Scans - Lab Notebook 4/WB0036.pdf]

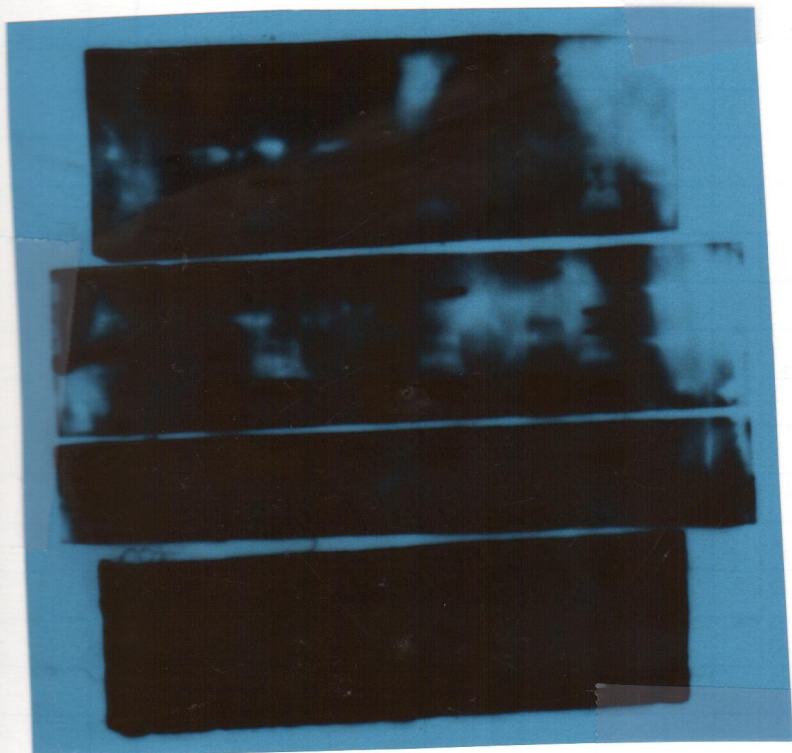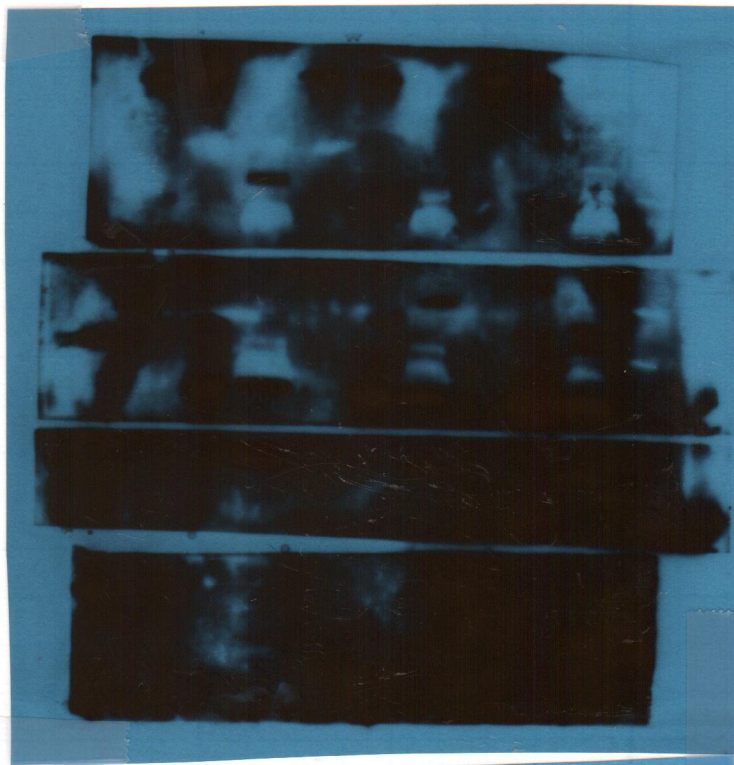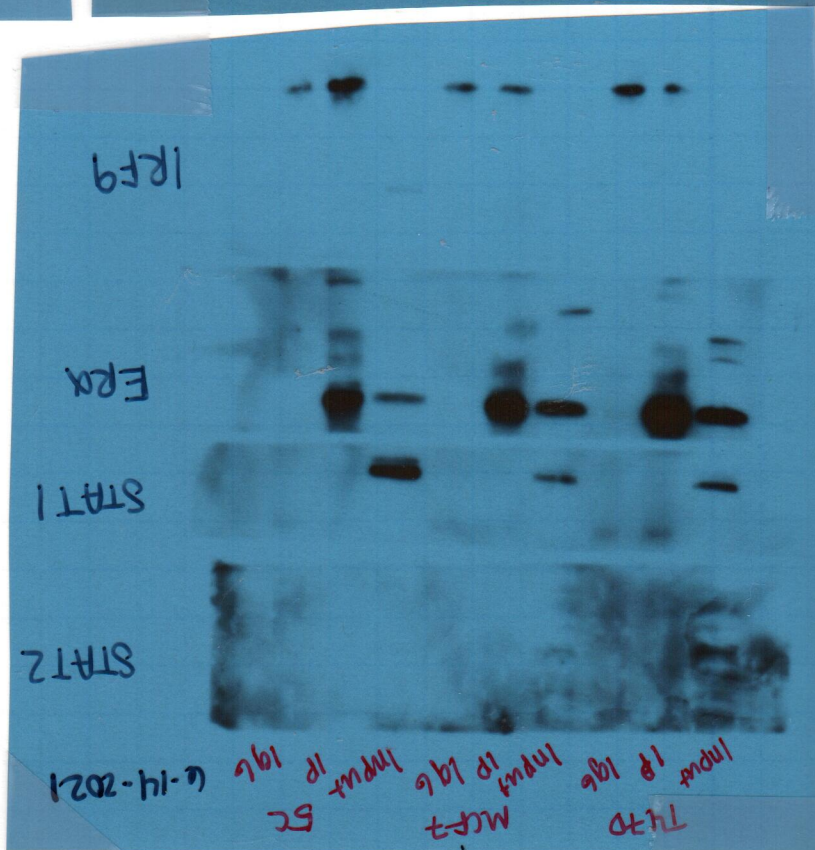

Supplement: Supplementary file 1 [file cancers-13-05130-s001.zip › cancers-1384109-supplementary/cancers-1384109-western blot/ER paper WBs/Western Scans - Lab Notebook 4/WB0037.pdf]

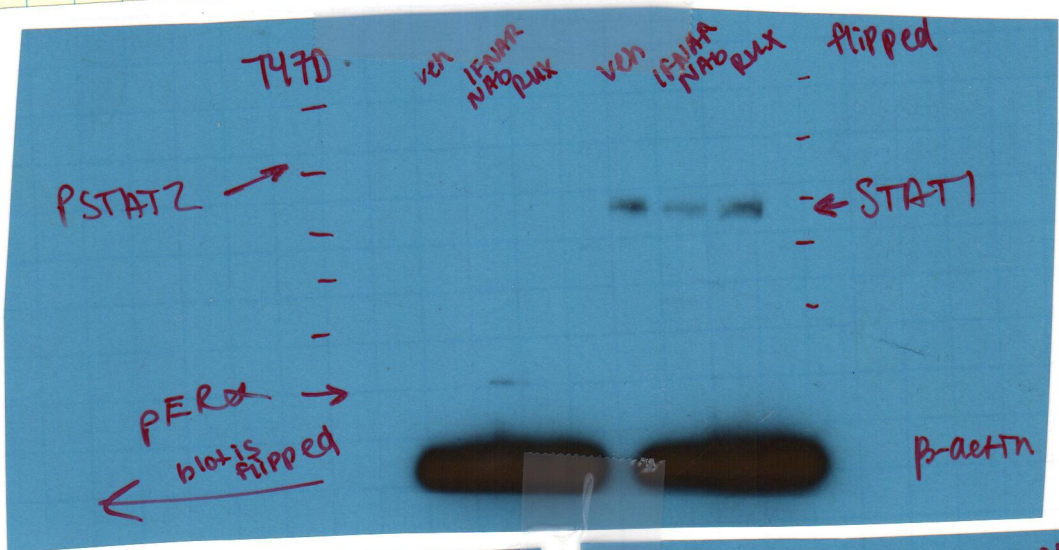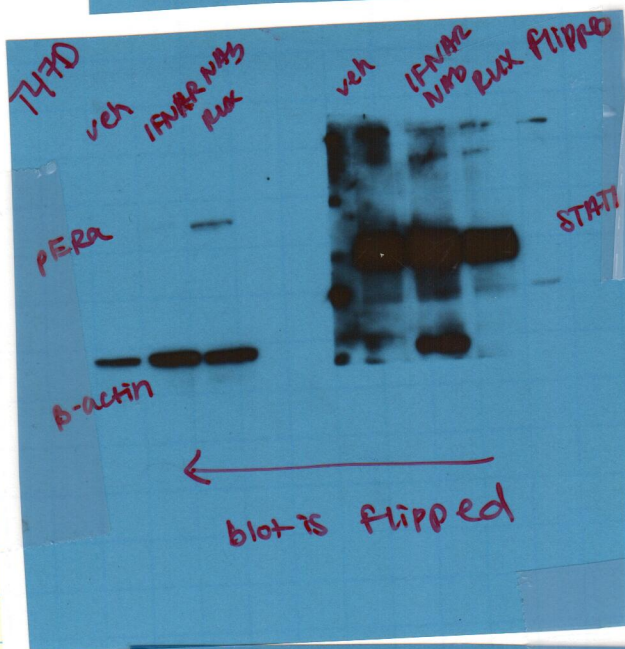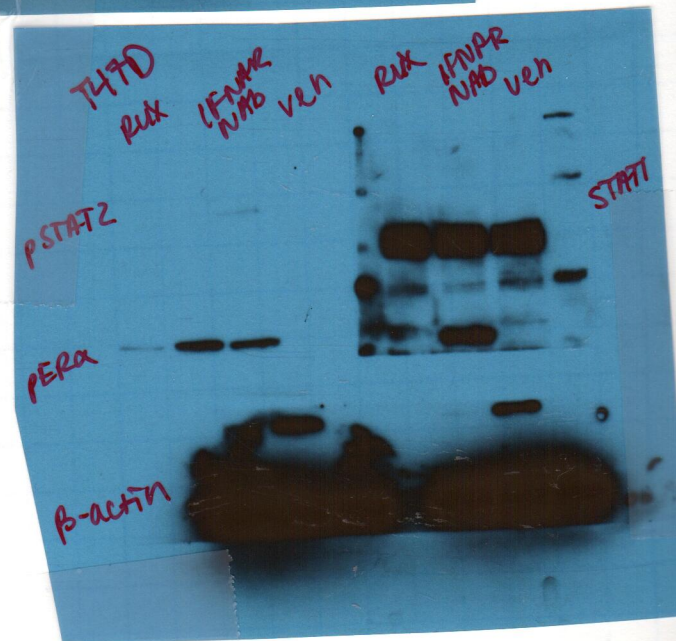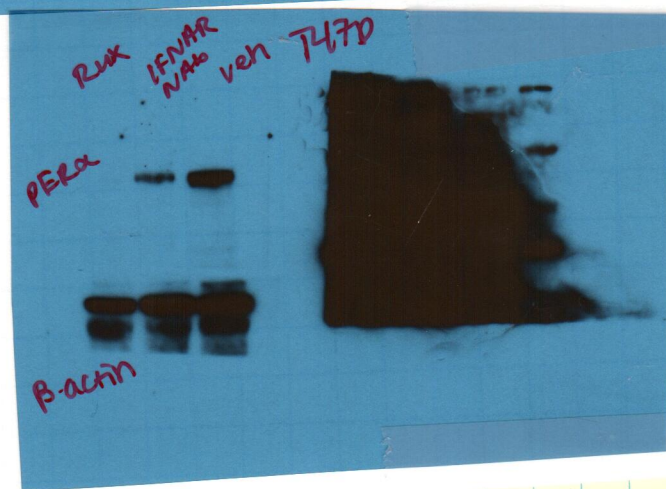

Supplement: Supplementary file 1 [file cancers-13-05130-s001.zip › cancers-1384109-supplementary/cancers-1384109-western blot/ER paper WBs/Western Scans - Lab Notebook 4/WB0038.pdf]

~~7470~~ Ran Western

marker

7470

MCF7

5C

7470  
siCon

siER

MCF7  
siCon

siER

5C  
siCon

siER

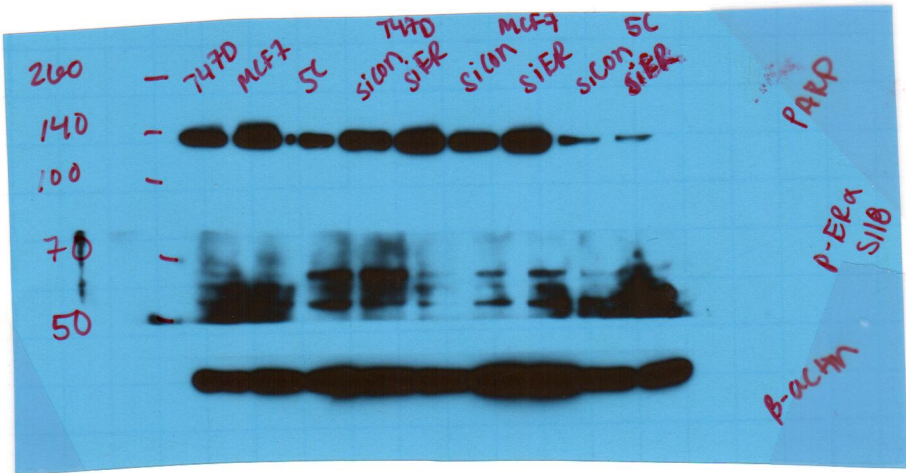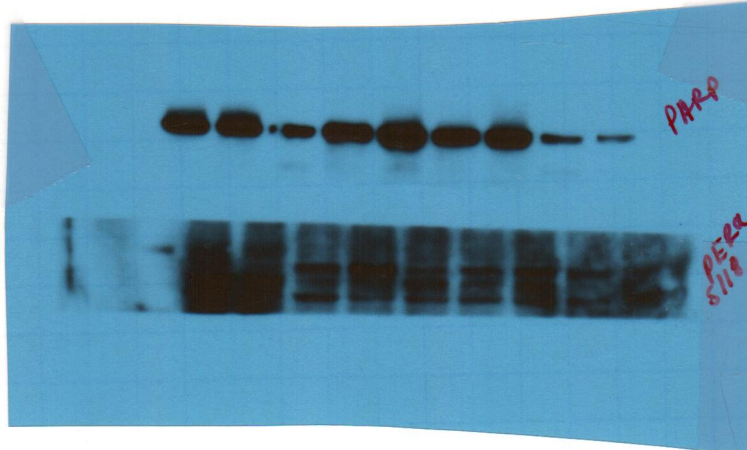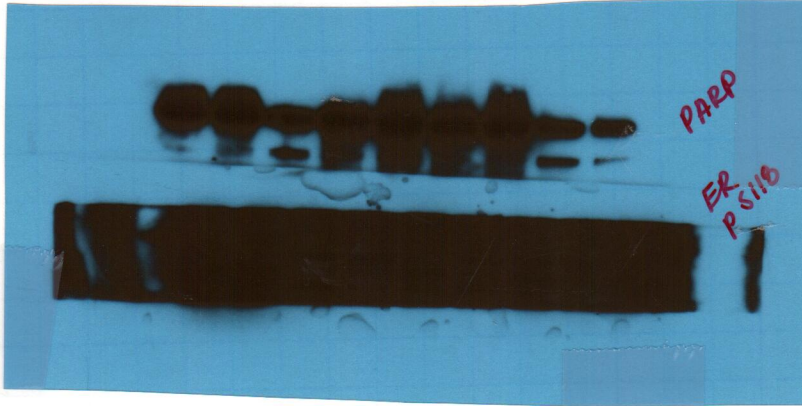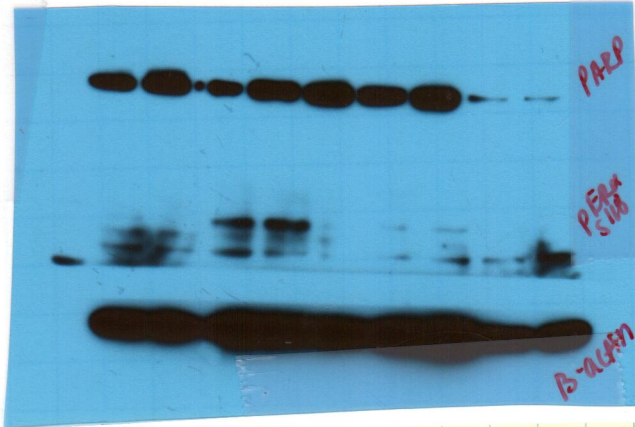

Supplement: Supplementary file 1 [file cancers-13-05130-s001.zip › cancers-1384109-supplementary/cancers-1384109-western blot/ER paper WBs/Western Scans - Lab Notebook 4/WB0039.pdf]

I think I cut off to close to show cleaved.

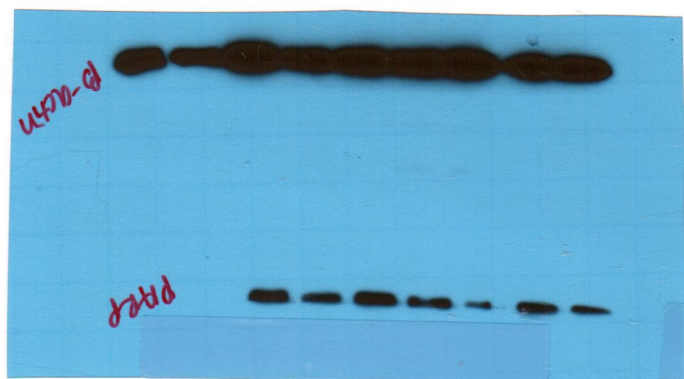

Supplement: Supplementary file 1 [file cancers-13-05130-s001.zip › cancers-1384109-supplementary/cancers-1384109-western blot/ER paper WBs/Western Scans - Lab Notebook 4/WB0040.pdf]

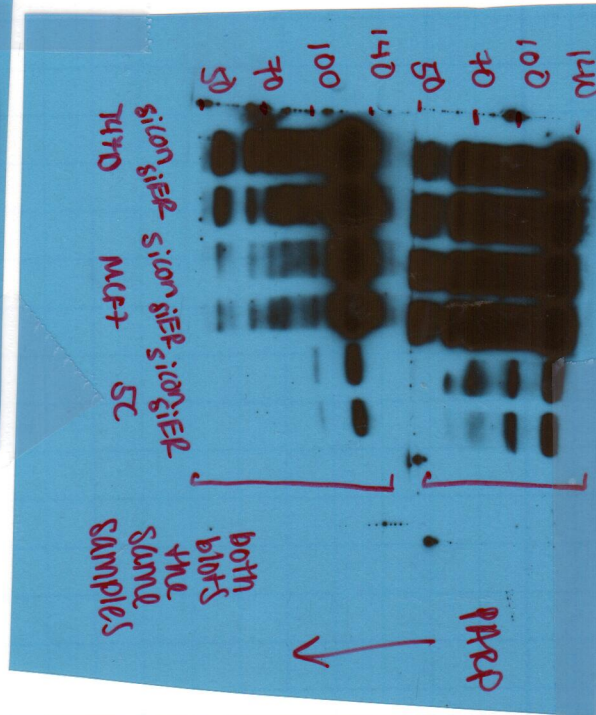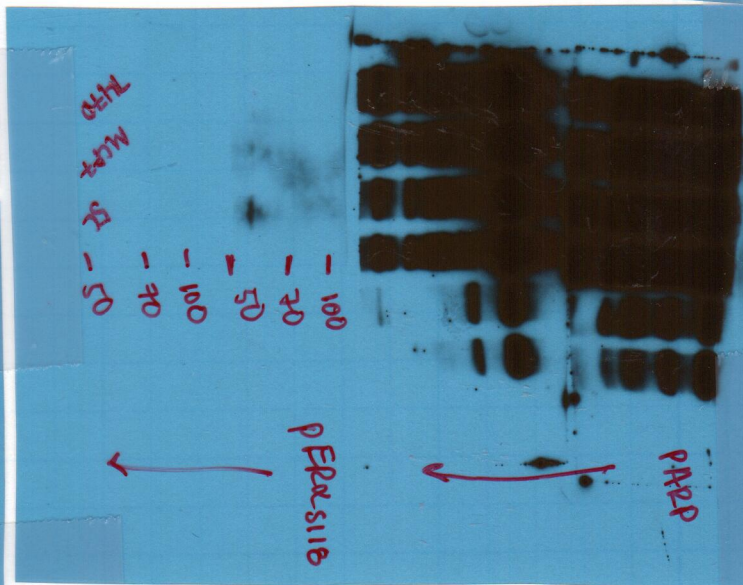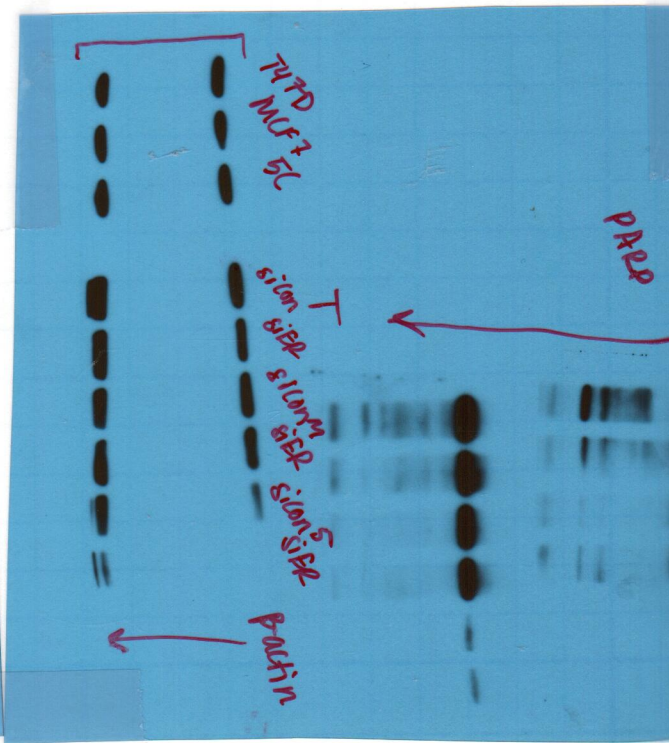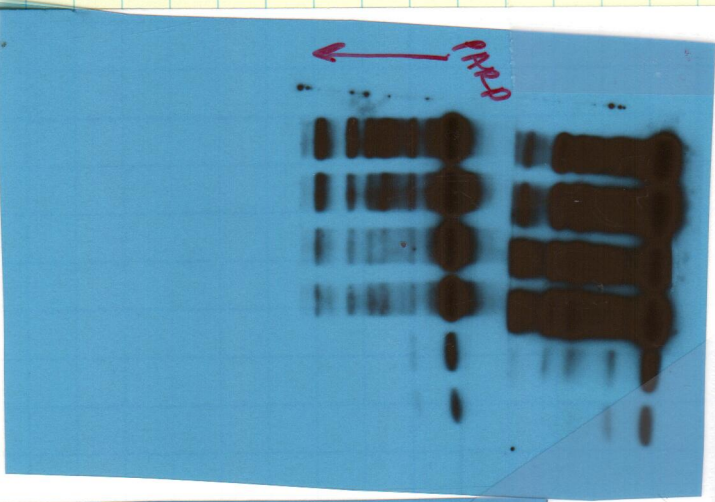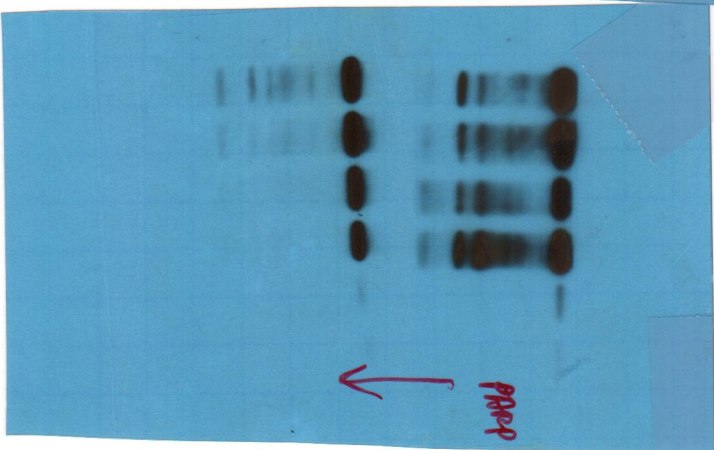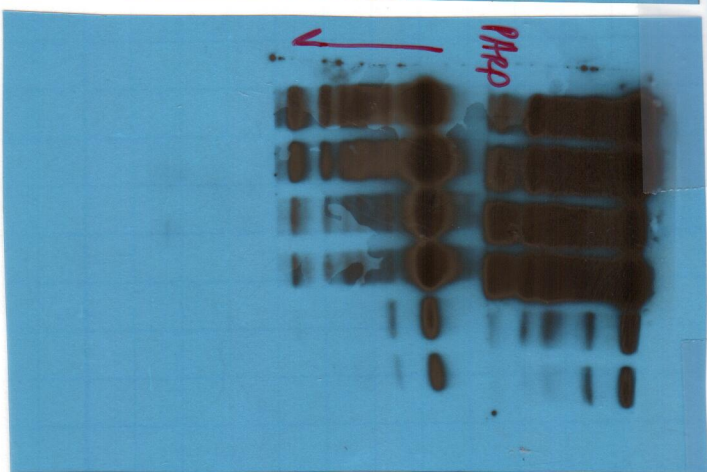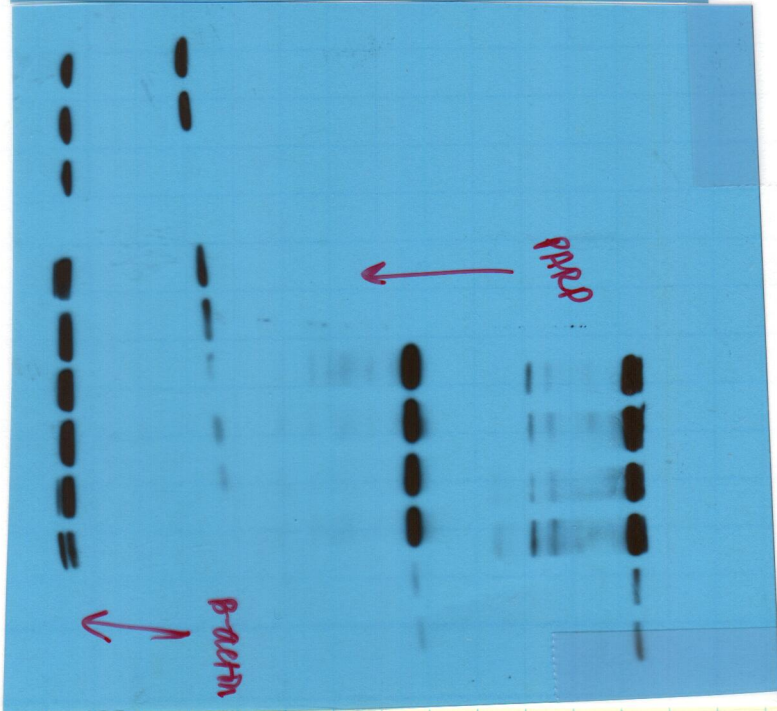

Supplement: Supplementary file 1 [file cancers-13-05130-s001.zip › cancers-1384109-supplementary/cancers-1384109-western blot/ER paper WBs/Western Scans - Lab Notebook 4/WB0041.pdf]

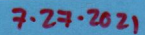

Supplement: Supplementary file 1 [file cancers-13-05130-s001.zip › cancers-1384109-supplementary/cancers-1384109-western blot/ER paper WBs/Western Scans - Lab Notebook 4/WBs for ER Paper/WB.pdf]

7.27.2021

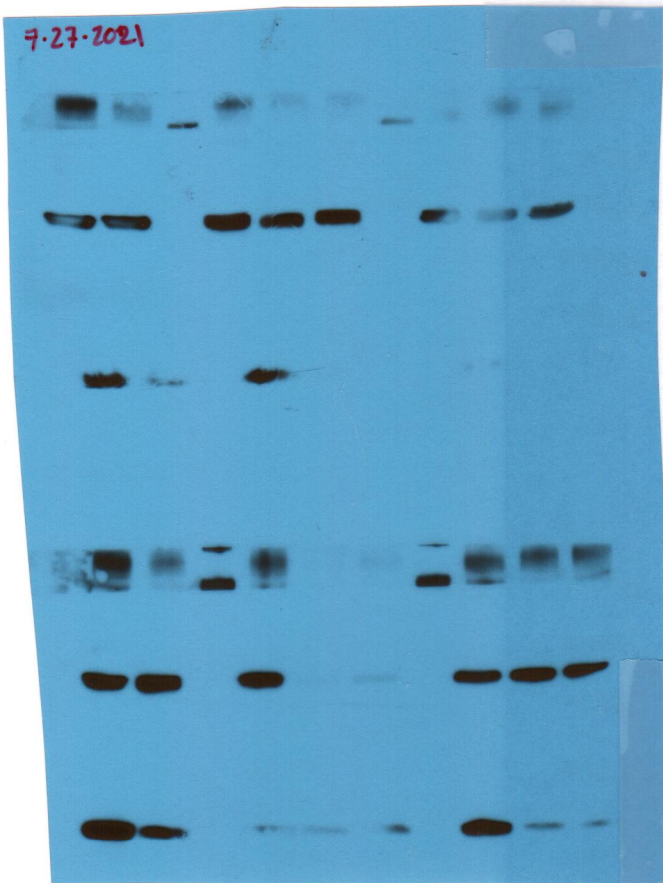

7.27.2021

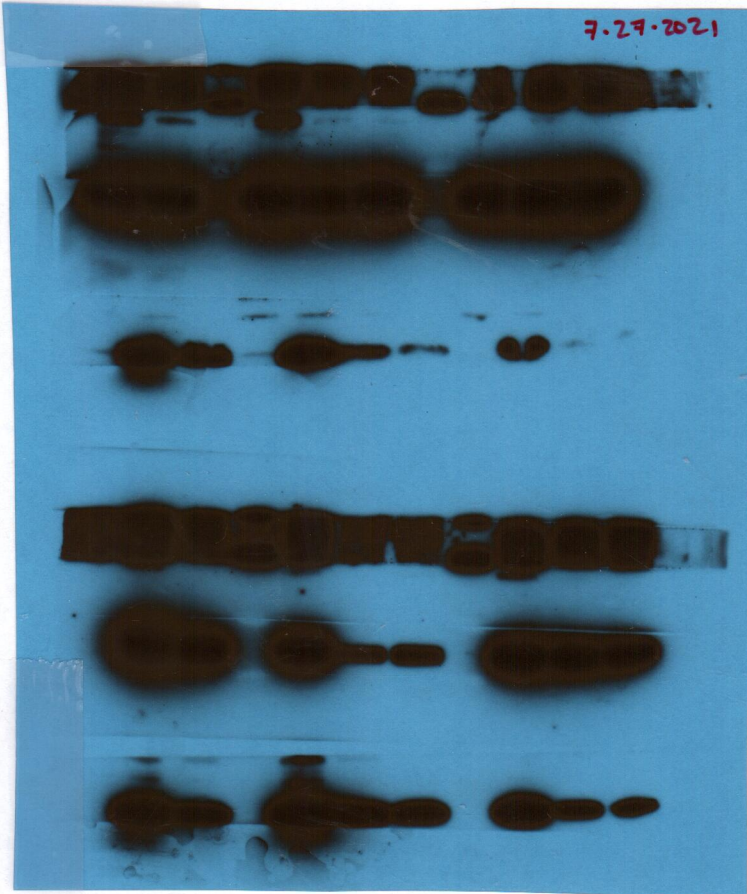

7.27.2021

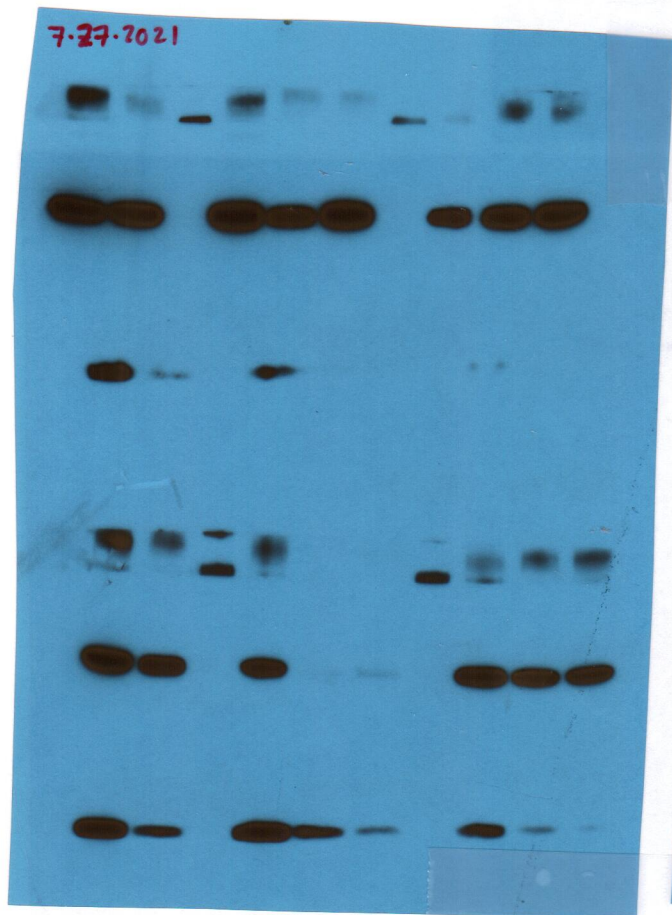

7.27.2021

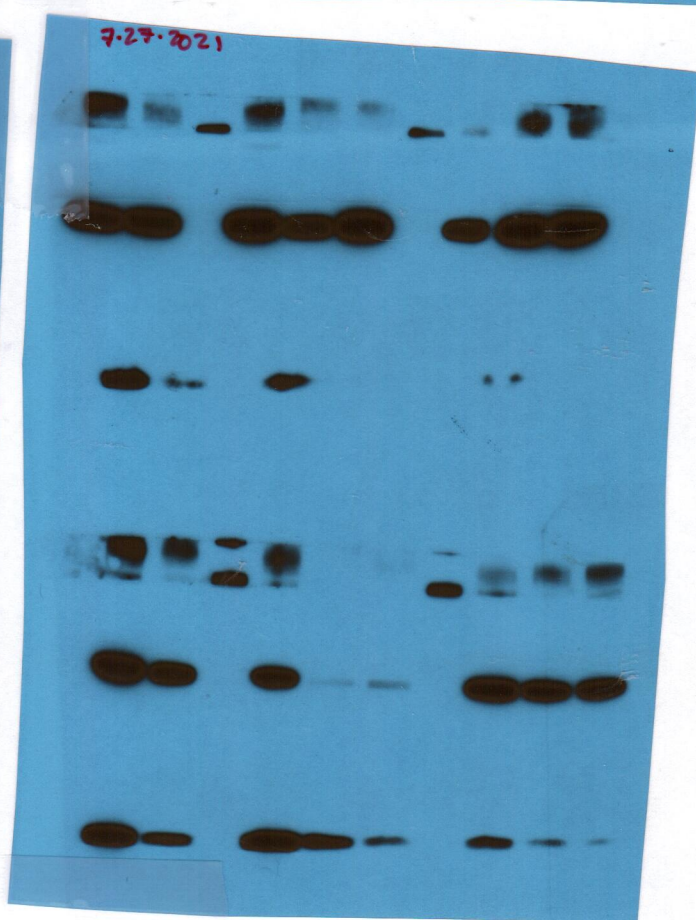

Supplement: Supplementary file 1 [file cancers-13-05130-s001.zip › cancers-1384109-supplementary/cancers-1384109-western blot/ER paper WBs/Western Scans - Lab Notebook 4/WBs for ER Paper/WB0001.pdf]

7.27.2021

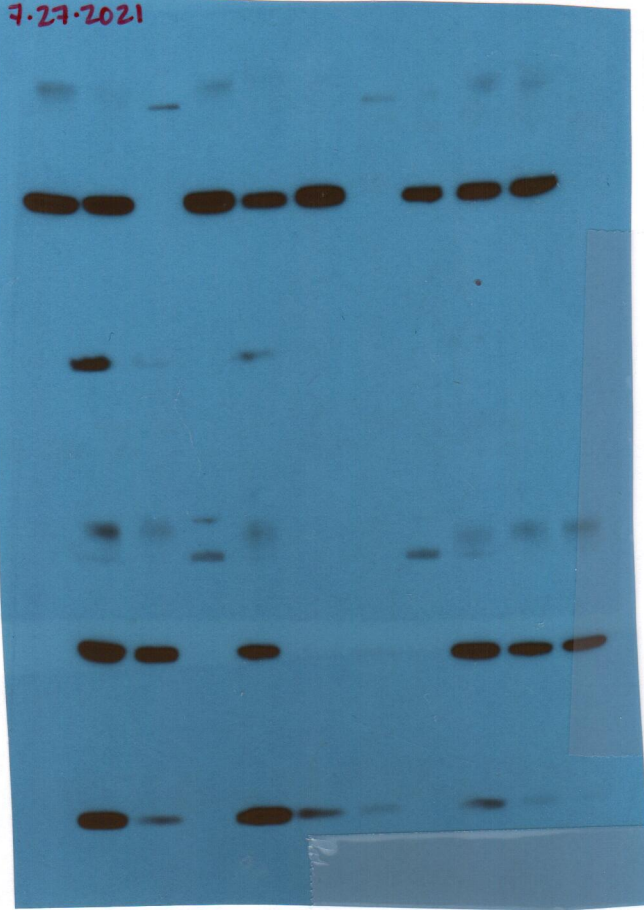

7.27.2021

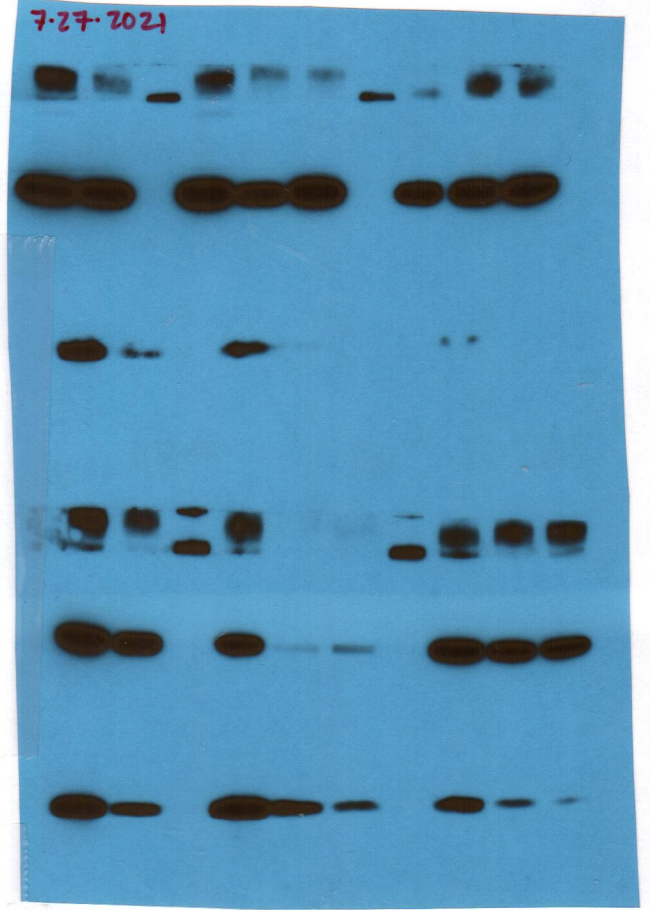

MCF7

7.28.2021

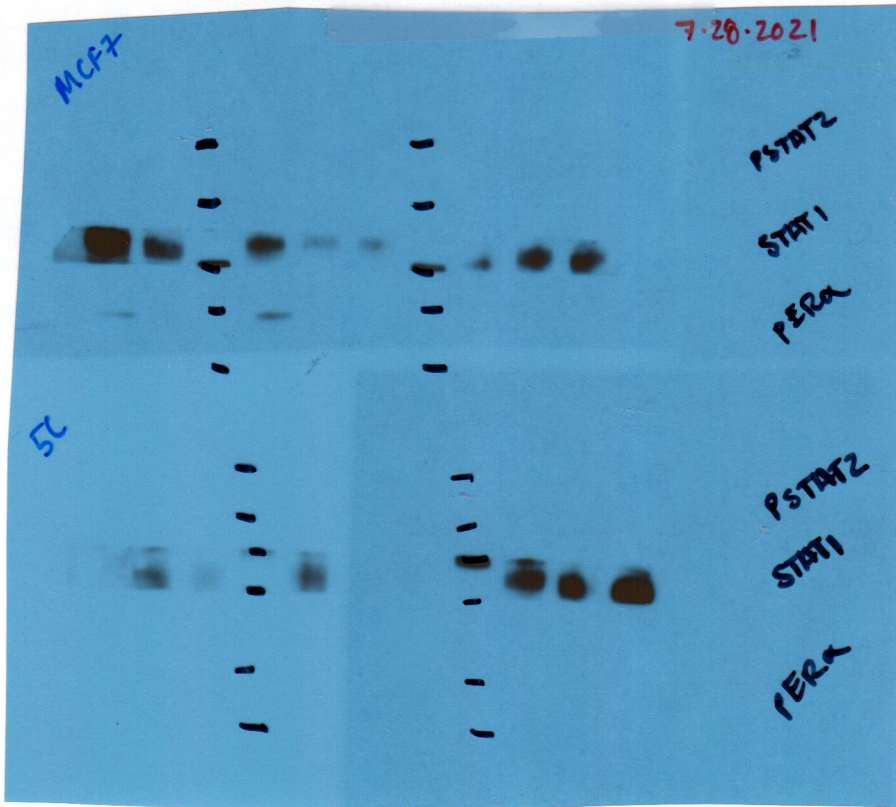

PSTAT2

STAT1

PERK

PSTAT2

STAT1

PERK

Supplement: Supplementary file 1 [file cancers-13-05130-s001.zip › cancers-1384109-supplementary/cancers-1384109-western blot/ER paper WBs/Western Scans - Lab Notebook 4/WBs for ER Paper/WB0002.pdf]

7.28.2021

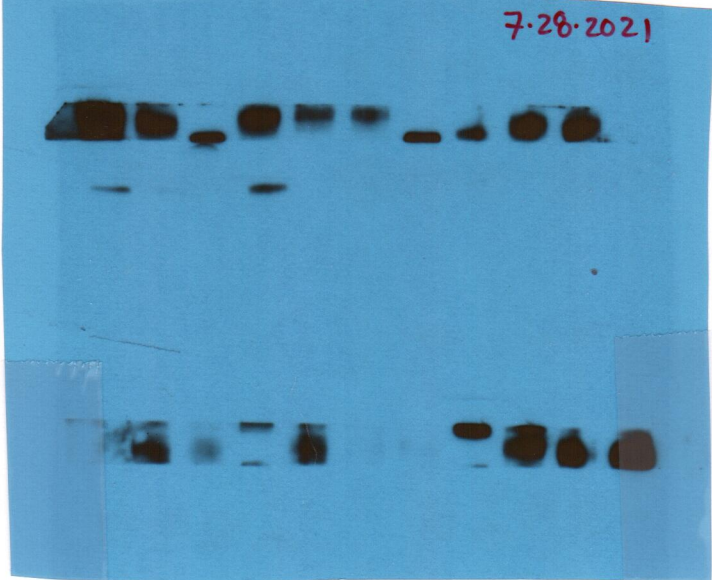

7.28.2021

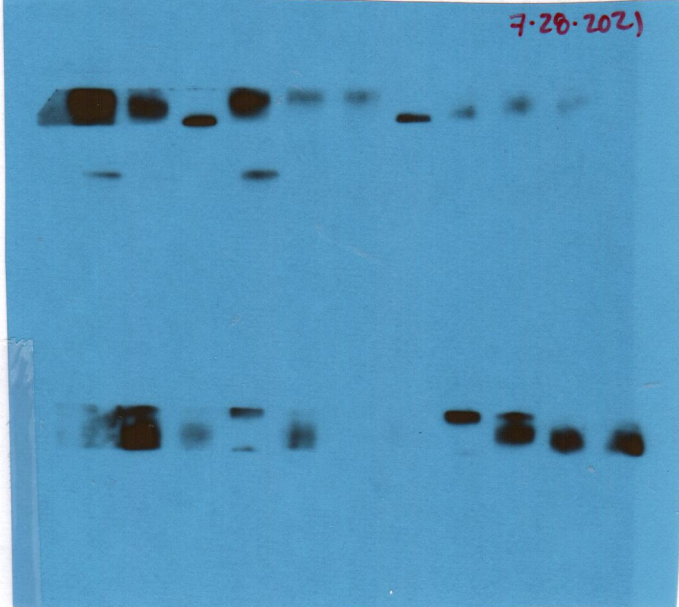

7.28.2021

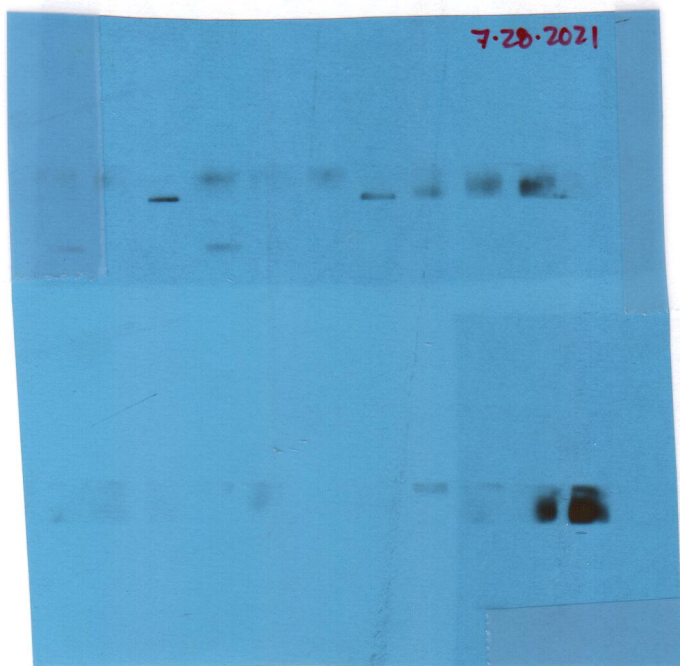

7.28.2021

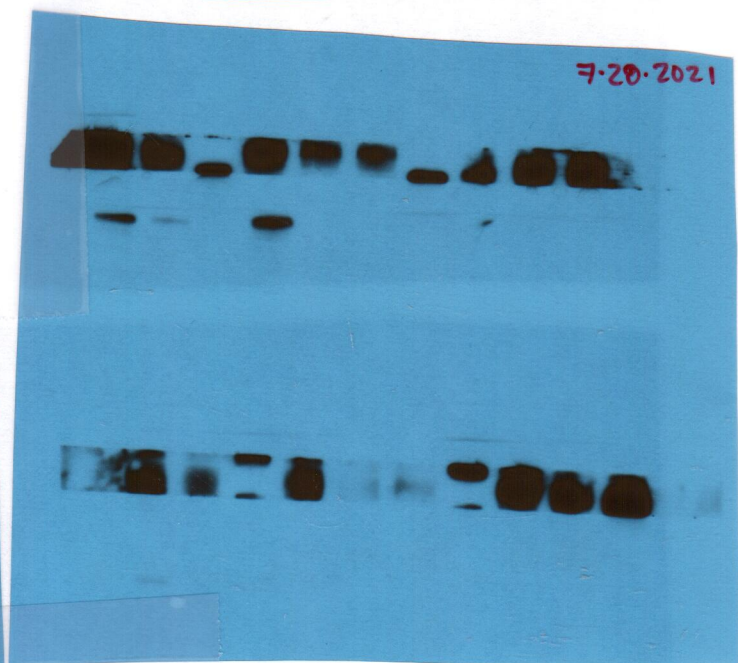

7.28.  
2021

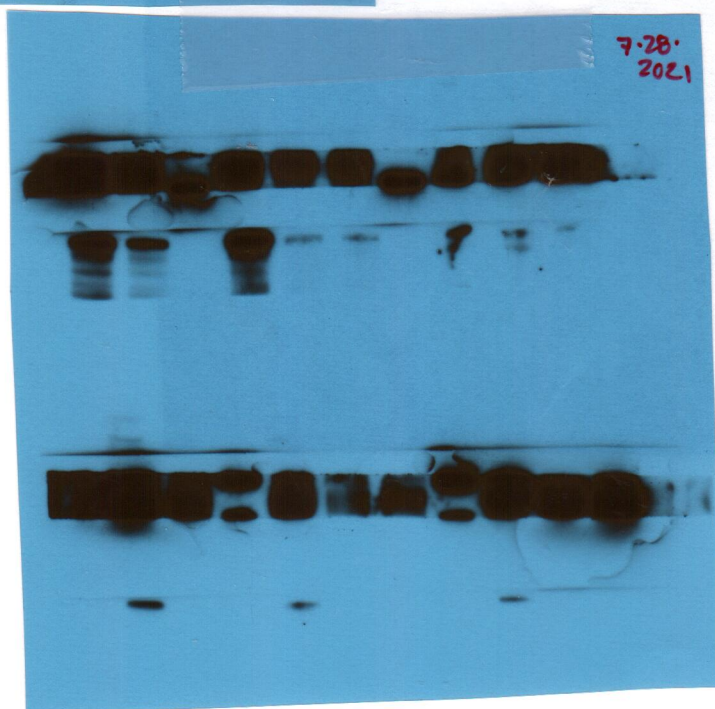

Supplement: Supplementary file 1 [file cancers-13-05130-s001.zip › cancers-1384109-supplementary/cancers-1384109-western blot/ER paper WBs/Western Scans - Lab Notebook 4/WBs for ER Paper/WB0003.pdf]

8-1-2021  
3m cels. #1

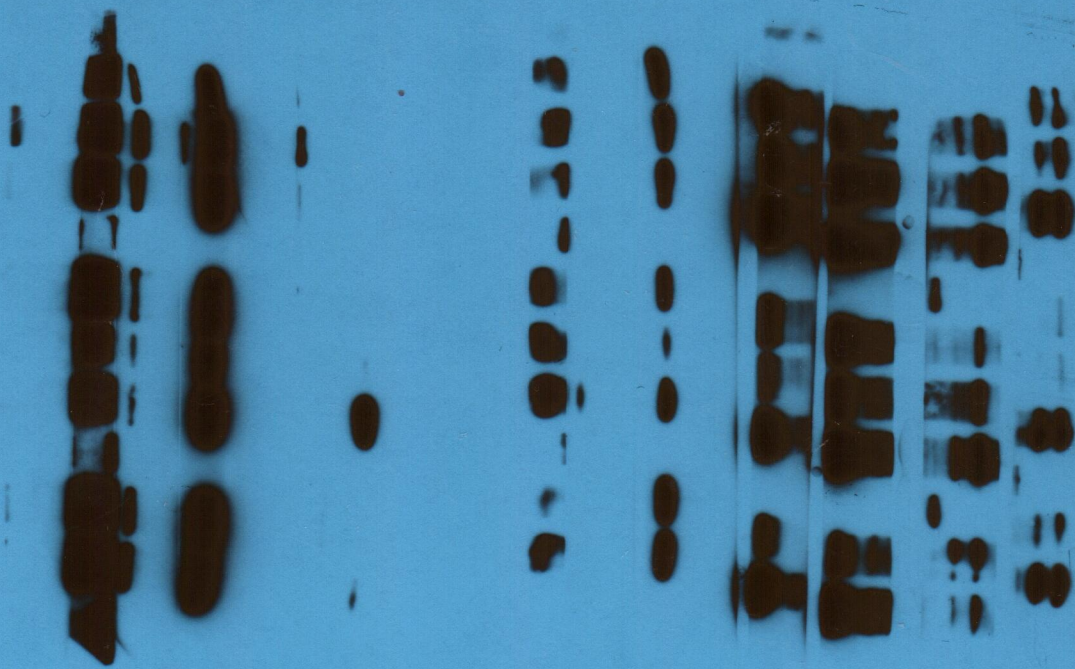

8-1-2021  
30s cels.

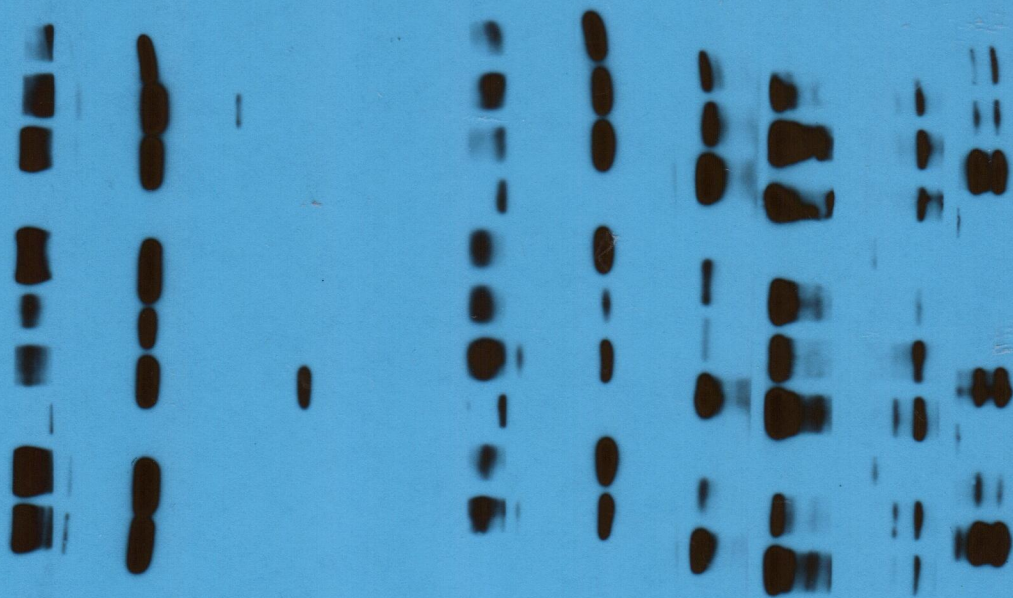

Supplement: Supplementary file 1 [file cancers-13-05130-s001.zip › cancers-1384109-supplementary/cancers-1384109-western blot/ER paper WBs/Western Scans - Lab Notebook 4/WBs for ER Paper/WB0004.pdf]

8-1-2021  
8-10-8 chcl.

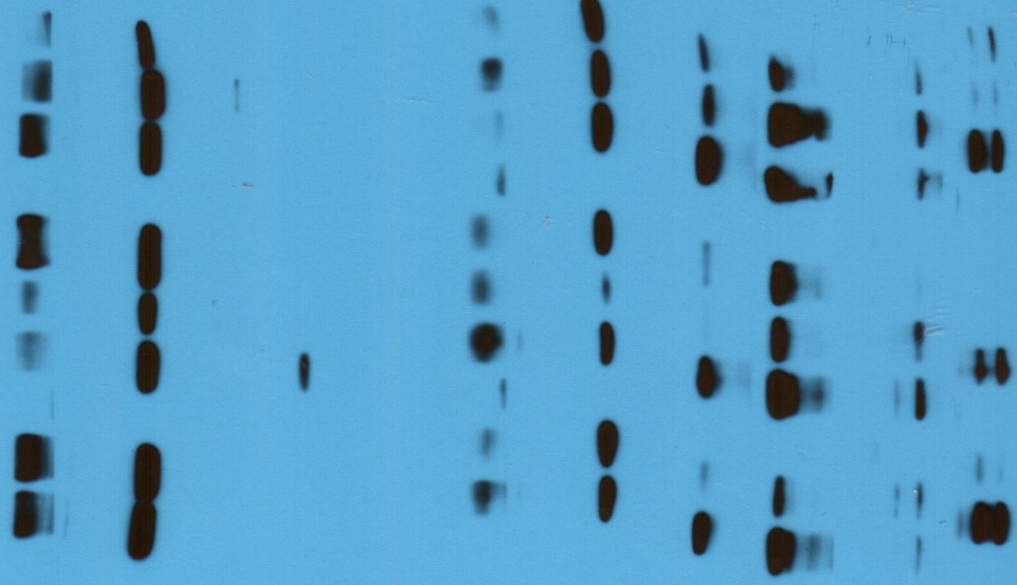

8-1-2021  
1 more.

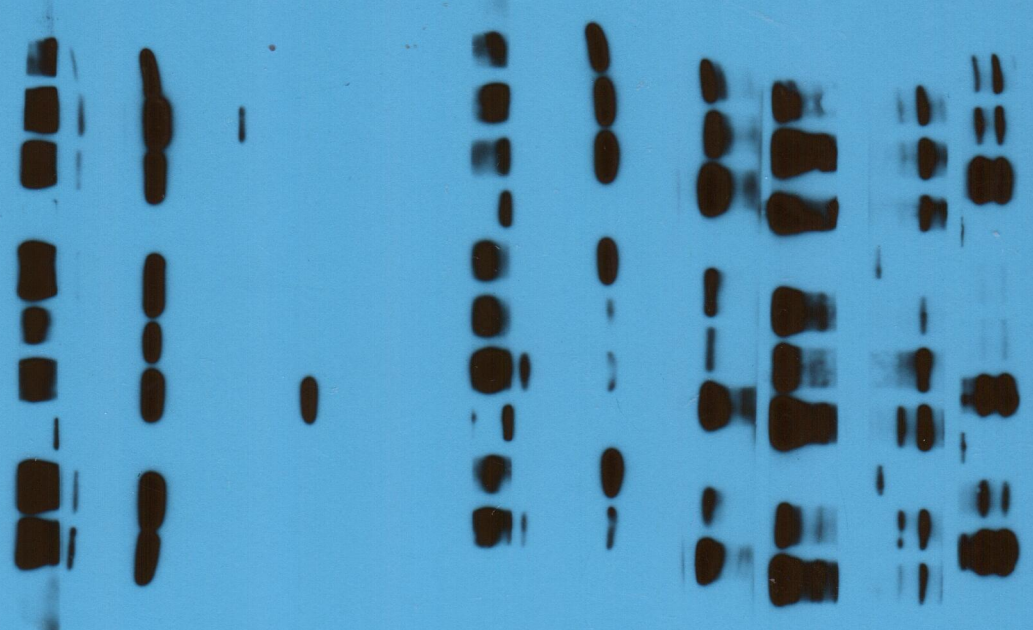

Supplement: Supplementary file 1 [file cancers-13-05130-s001.zip › cancers-1384109-supplementary/cancers-1384109-western blot/ER paper WBs/Western Scans - Lab Notebook 4/WBs for ER Paper/WB0007.pdf]

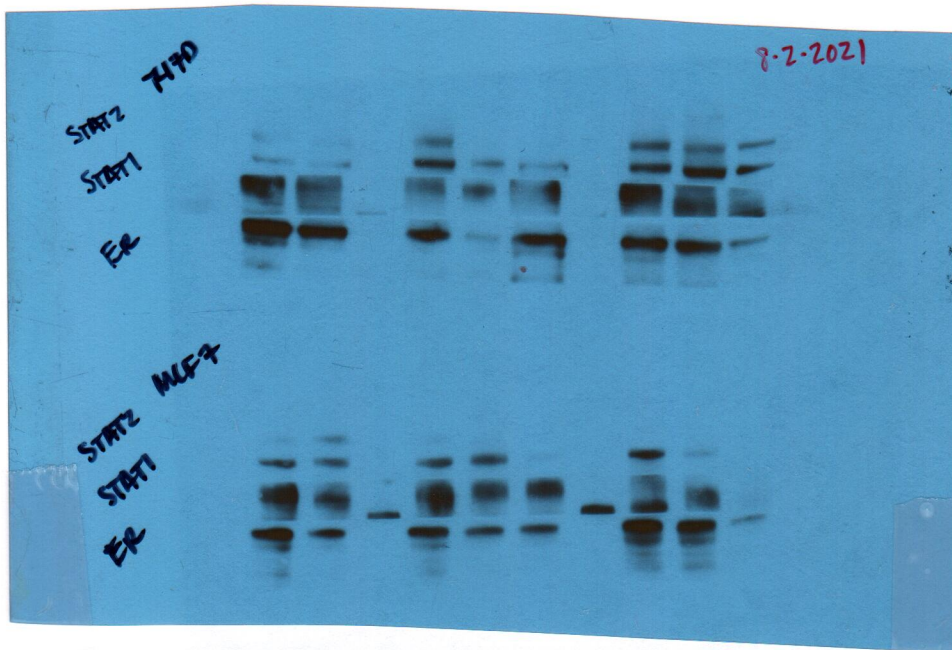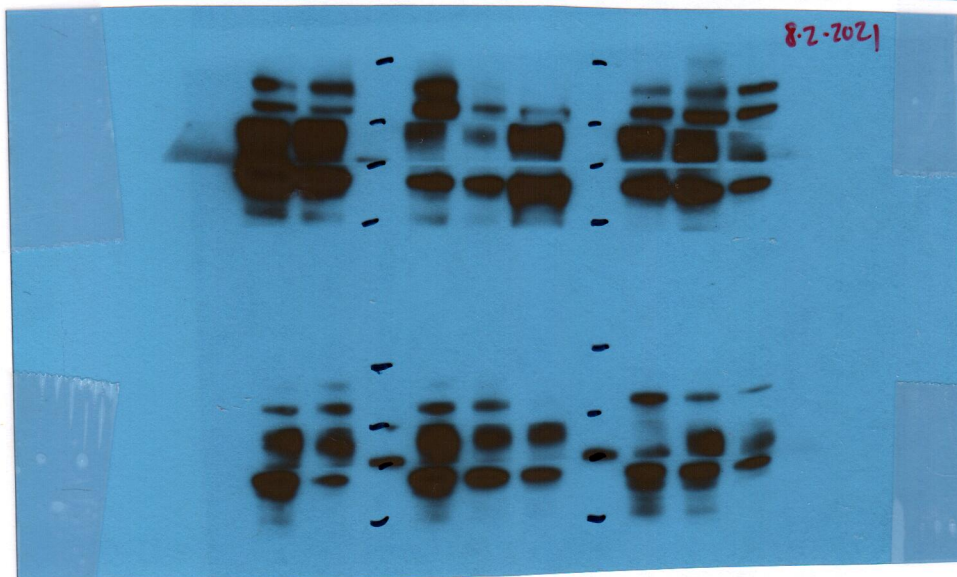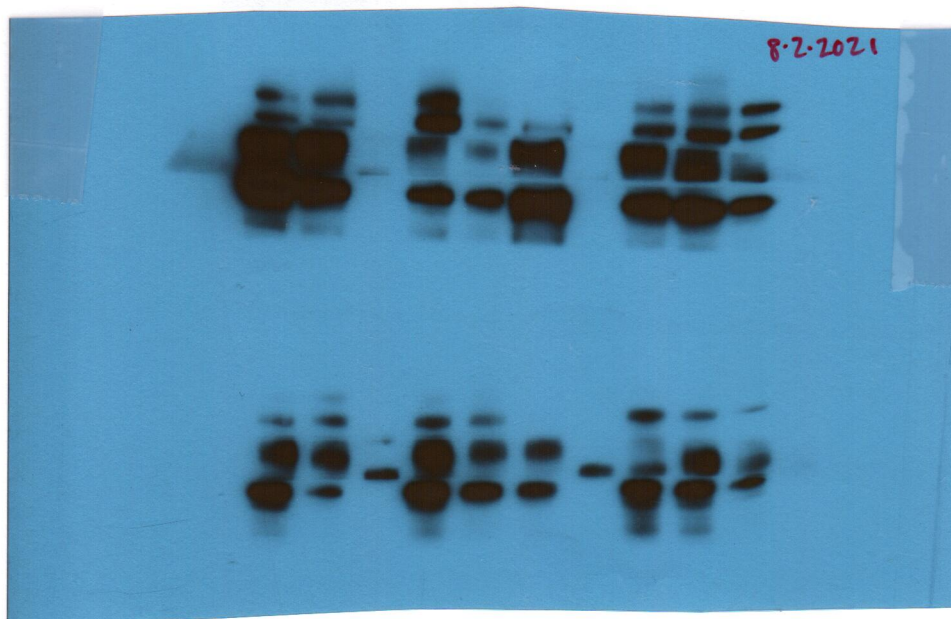

Supplement: Supplementary file 1 [file cancers-13-05130-s001.zip › cancers-1384109-supplementary/cancers-1384109-western blot/ER paper WBs/Western Scans - Lab Notebook 4/WBs for ER Paper/WB0008.pdf]

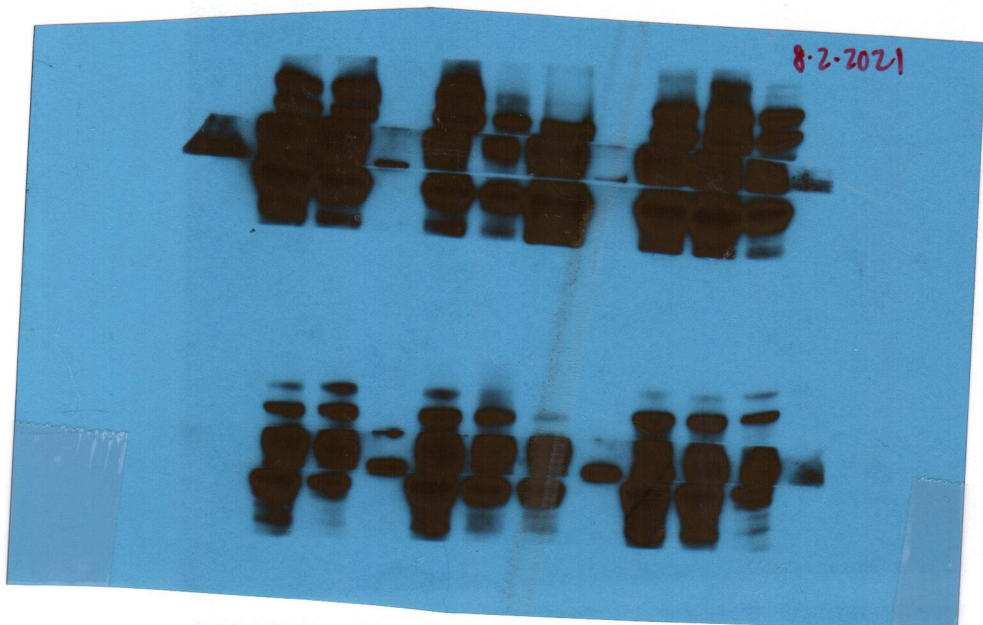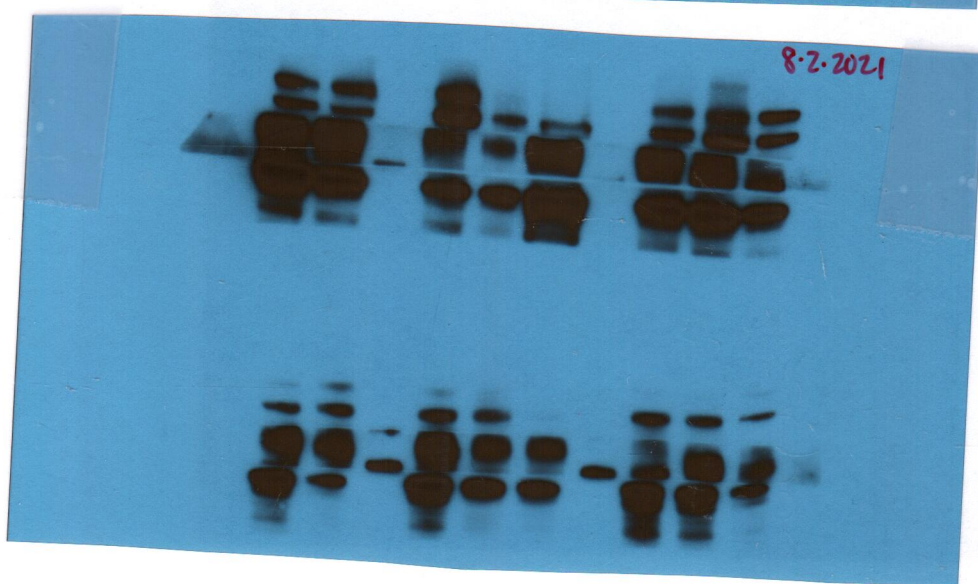

Supplement: Supplementary file 1 [file cancers-13-05130-s001.zip › cancers-1384109-supplementary/cancers-1384109-western blot/ER paper WBs/Western Scans - Lab Notebook 4/WBs for ER Paper/WB0009.pdf]
