# Supplementary material for: Enhanced IFNα Signaling Promotes Ligand-Independent Activation of ERα to Promote Aromatase Inhibitor Resistance in Breast Cancer
Source: Cancers (Basel). 2021 Oct 13;13(20):5130. doi: 10.3390/cancers13205130 (PMC8534010; doi:10.3390/cancers13205130)
Supplement: Supplementary file 1 [file WB.pdf › cancers-1384109-supplementary/cancers-1384109-western blot/ER paper WBs/WB.pdf]

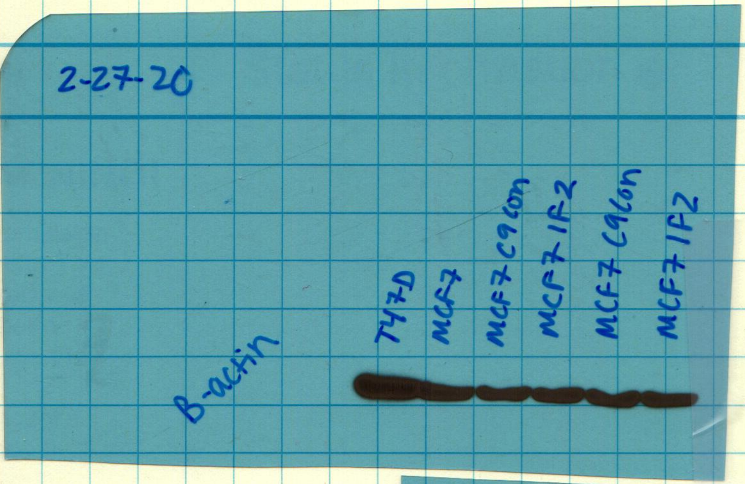

Not much IFITM1 here.

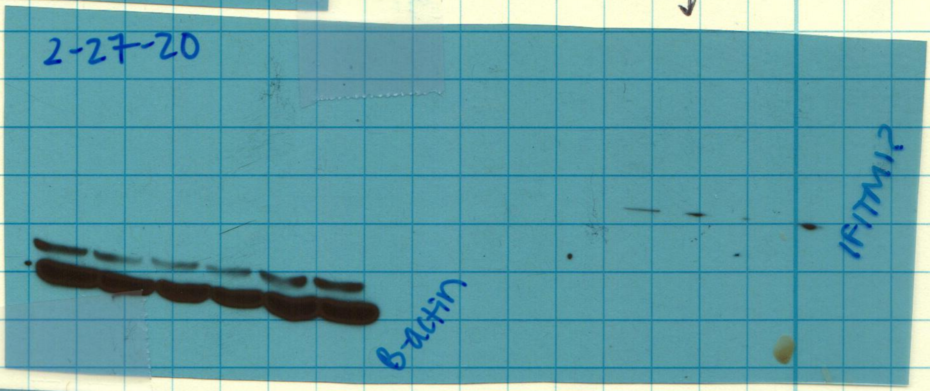

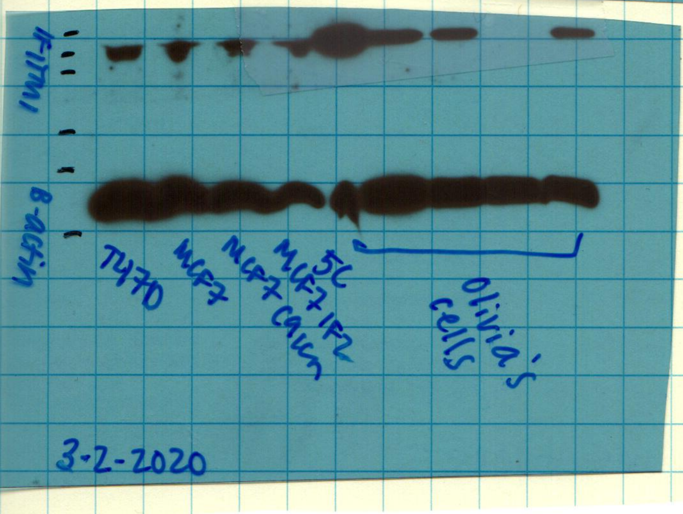

Might have loaded too much protein in my samples will retry once we get more gels in.

3-2020

3 ug/ul

3-20	Cell Line	Sample #	con. (ug/u	Sample B.	Protein	D.W.	Total
	T47D	1	13.2	10.00	***9.08***	***20.92***	40.00
	MCF-7	2	14.1	10.00	***8.52***	***21.48***	40.00
	MCF-7 C9Con	3	14.0	10.00	***8.55***	***21.45***	40.00
	MCF-7 IF2	4	14.2	10.00	***8.46***	***21.54***	40.00
	5C	5	7.9	10.00	***15.23***	***14.77***	40.00

2-2020
l Counting

10,000 cells/well

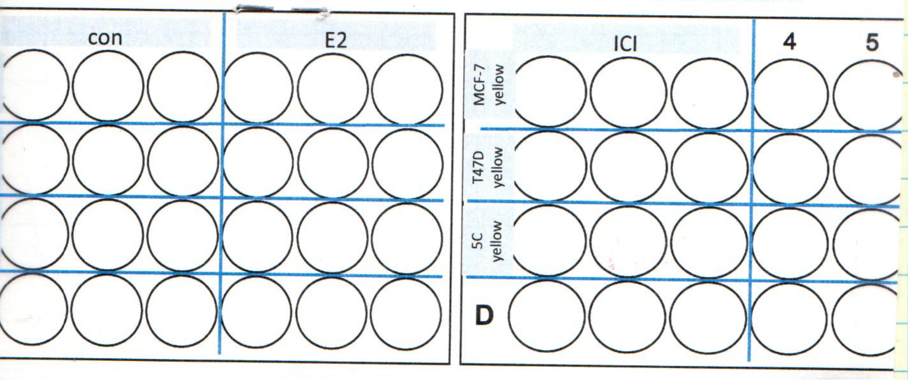

Passaged MCF7 red + yellow
T47D red + yellow
MCF7 C9 con
MCF7 IF2 cells

5C cells look like they are dying still.
