# Supplementary material for: Enhanced IFNα Signaling Promotes Ligand-Independent Activation of ERα to Promote Aromatase Inhibitor Resistance in Breast Cancer
Source: Cancers (Basel). 2021 Oct 13;13(20):5130. doi: 10.3390/cancers13205130 (PMC8534010; doi:10.3390/cancers13205130)
Supplement: Supplementary file 1 [file WB0012.pdf › cancers-1384109-supplementary/cancers-1384109-western blot/ER paper WBs/WB0012.pdf]

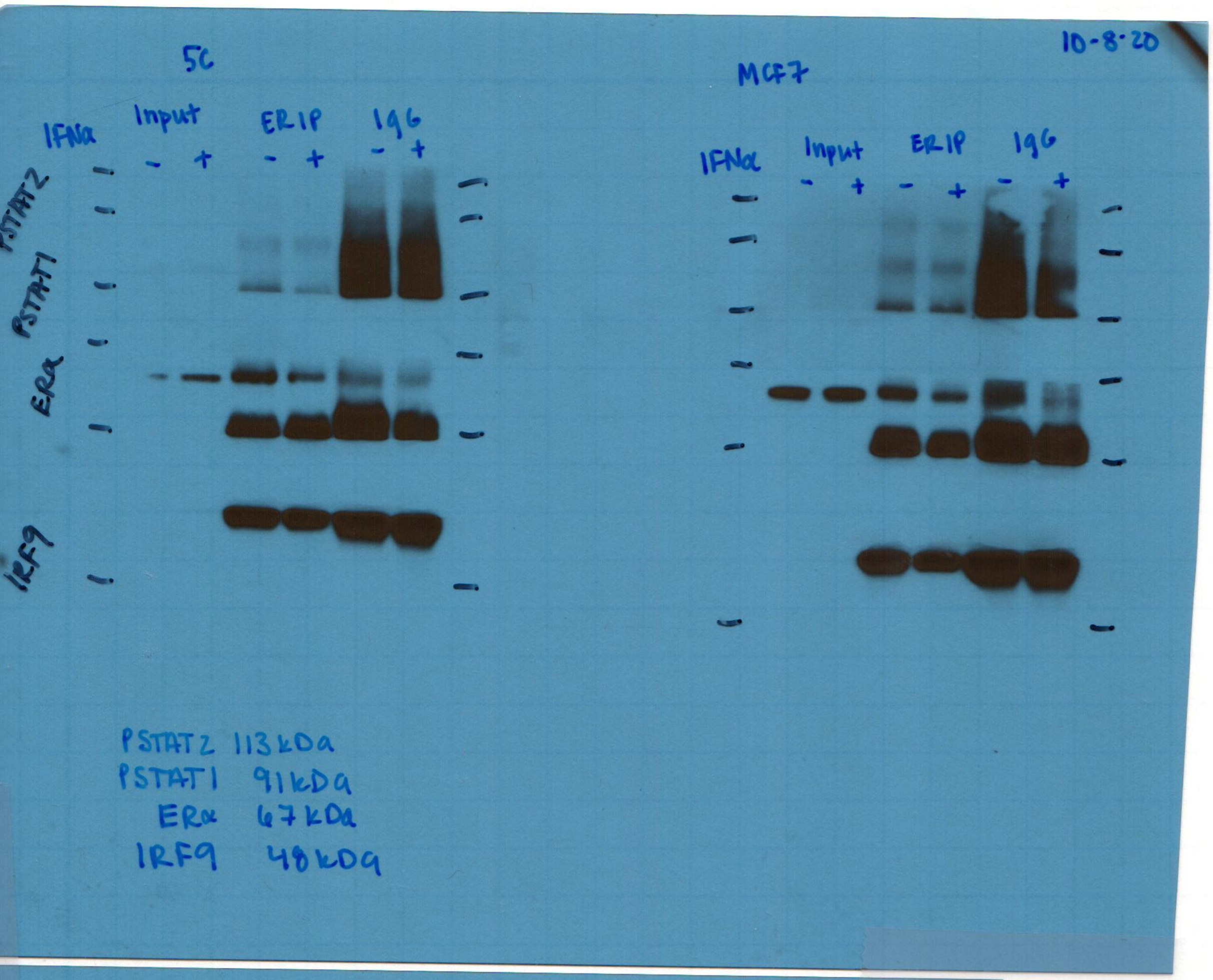
5C
MCF7
10-8-20
IFNα
Input
ERIP
IgG
- + - + - +
pSTAT2
pSTAT1
ERα
IRF9
pSTAT2 113kDa
pSTAT1 91kDa
ERα 67kDa
IRF9 48kDa

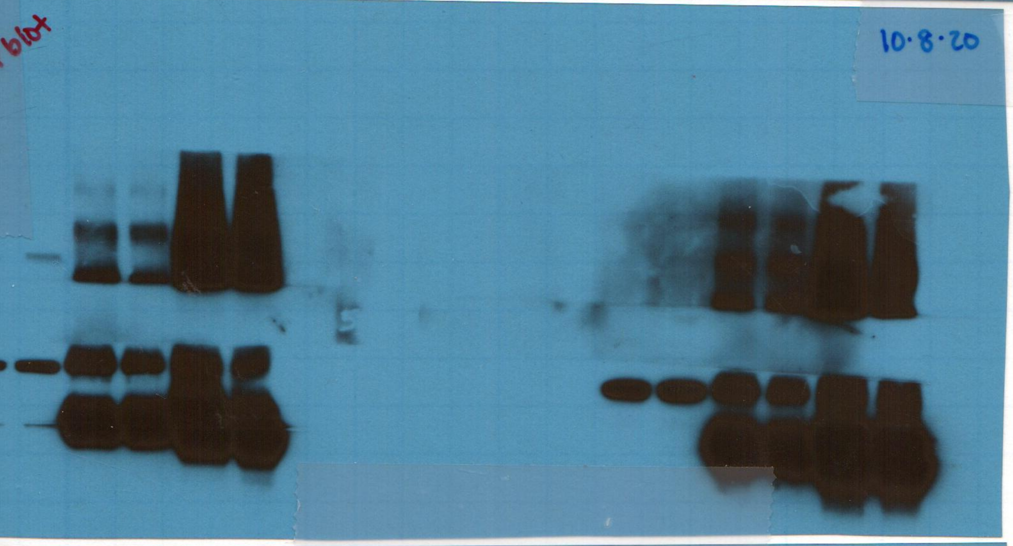
blot
10-8-20

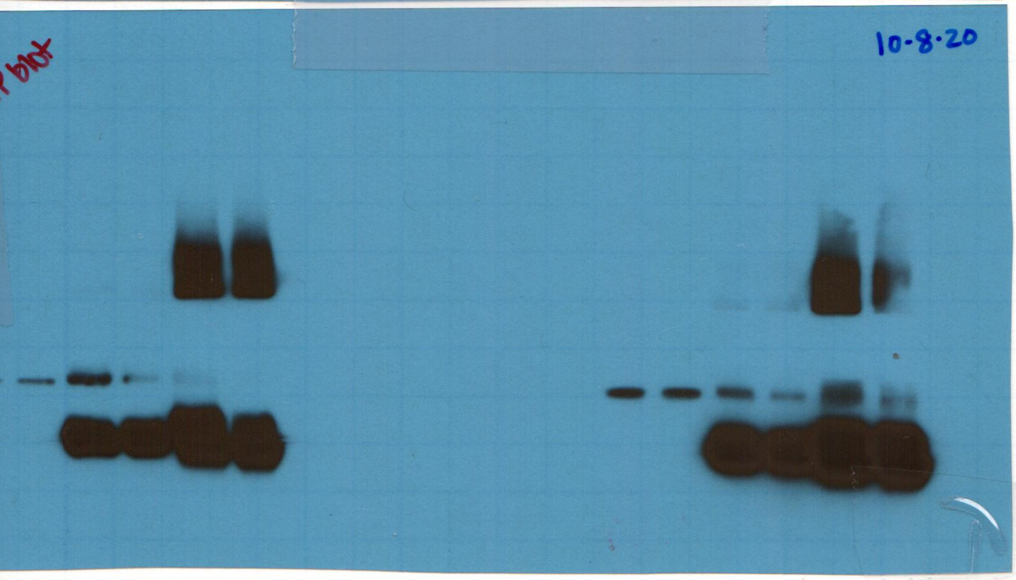
blot
10-8-20

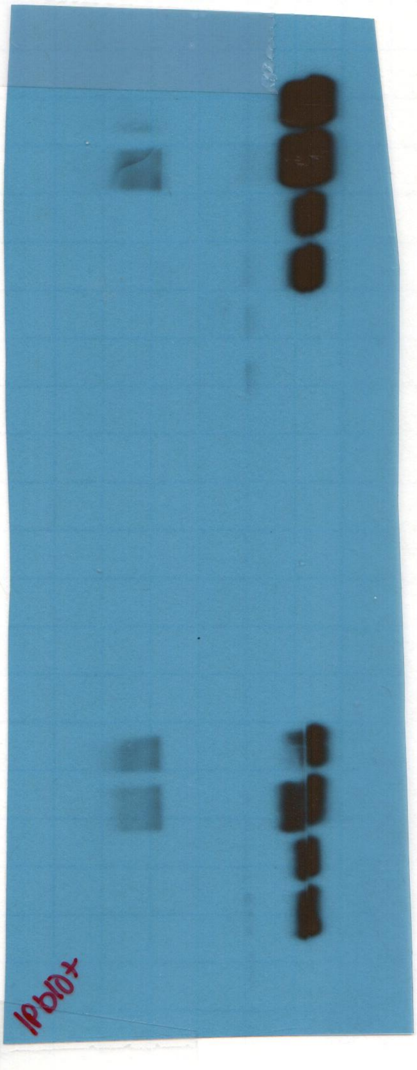
IP blot
