# Supplementary material for: Enhanced IFNα Signaling Promotes Ligand-Independent Activation of ERα to Promote Aromatase Inhibitor Resistance in Breast Cancer
Source: Cancers (Basel). 2021 Oct 13;13(20):5130. doi: 10.3390/cancers13205130 (PMC8534010; doi:10.3390/cancers13205130)
Supplement: Supplementary file 1 [file WB0013.pdf › cancers-1384109-supplementary/cancers-1384109-western blot/ER paper WBs/Western Scans - Lab Notebook 3 IN BINDER/WB0013.pdf]

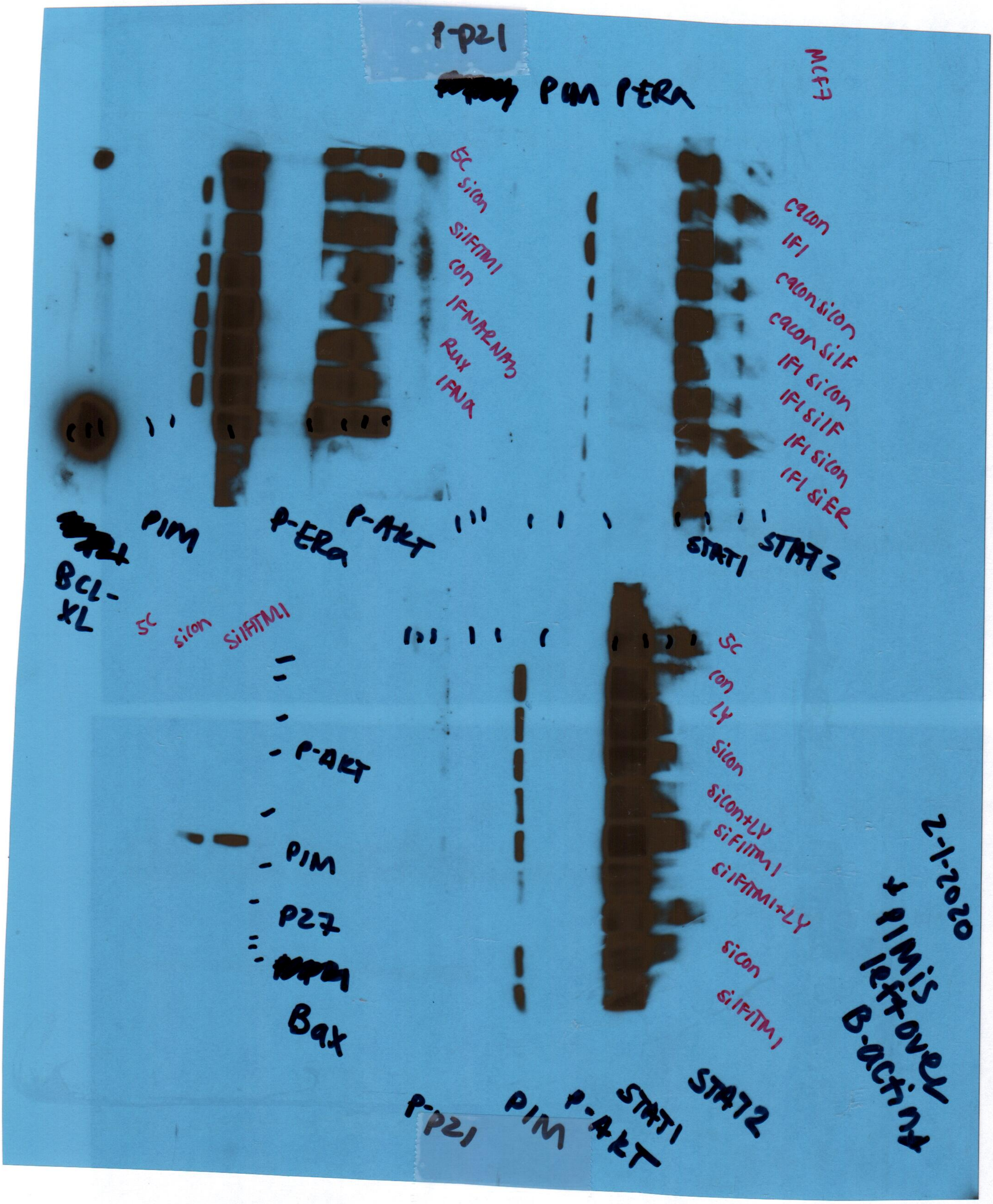

P-P21
PIM PERa
MCF7
SC
sicon
siIFITM1
con
IFNAR NA3
Rux
IFNa
cacon
IFI
cacon sicon
cacon siIF
IFI sicon
IFI siIF
IFI sicon
IFI siER
BCL-XL
PIM
P-ERa
P-AKT
STAT1
STAT2
SC
sicon
siIFITM1
P-AKT
PIM
P27
Bax
SC
con
LY
sicon
sicon+LY
siIFITM1
siIFITM1+LY
sicon
siIFITM1
2-1-2020
+ PIMs leftover
B-actin
STAT1
STAT2
P-P21
PIM
P-AKT
