# Supplementary material for: Enhanced IFNα Signaling Promotes Ligand-Independent Activation of ERα to Promote Aromatase Inhibitor Resistance in Breast Cancer
Source: Cancers (Basel). 2021 Oct 13;13(20):5130. doi: 10.3390/cancers13205130 (PMC8534010; doi:10.3390/cancers13205130)
Supplement: Supplementary file 1 [file WB0034.pdf › cancers-1384109-supplementary/cancers-1384109-western blot/ER paper WBs/Western Scans - Lab Notebook 3 IN BINDER/WB0034.pdf]

6·17·2020

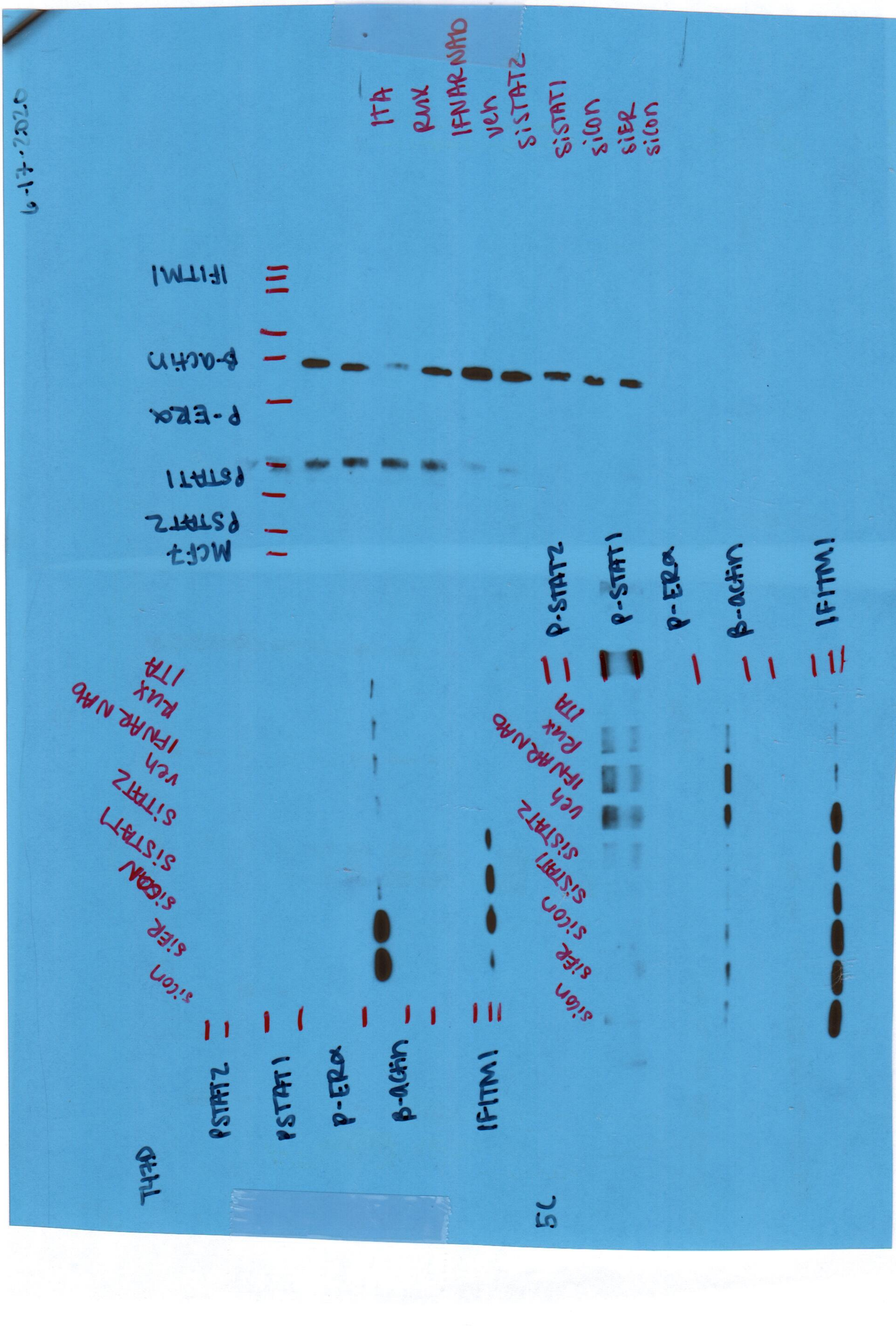
6-17-2020
MCF-7
pSTAT2
pSTAT1
p-ERα
β-actin
IFITM1
ITA
Rux
IFNAR-NAb
veh
siSTAT2
siSTAT1
sicon
siER
sicon
T47D
5C
p-STAT2
p-STAT1
p-ERα
β-actin
IFITM1
