# Supplementary material for: Enhanced IFNα Signaling Promotes Ligand-Independent Activation of ERα to Promote Aromatase Inhibitor Resistance in Breast Cancer
Source: Cancers (Basel). 2021 Oct 13;13(20):5130. doi: 10.3390/cancers13205130 (PMC8534010; doi:10.3390/cancers13205130)
Supplement: Supplementary file 1 [file WB0011.pdf › cancers-1384109-supplementary/cancers-1384109-western blot/ER paper WBs/Western Scans - Lab Notebook 4/WB0011.pdf]

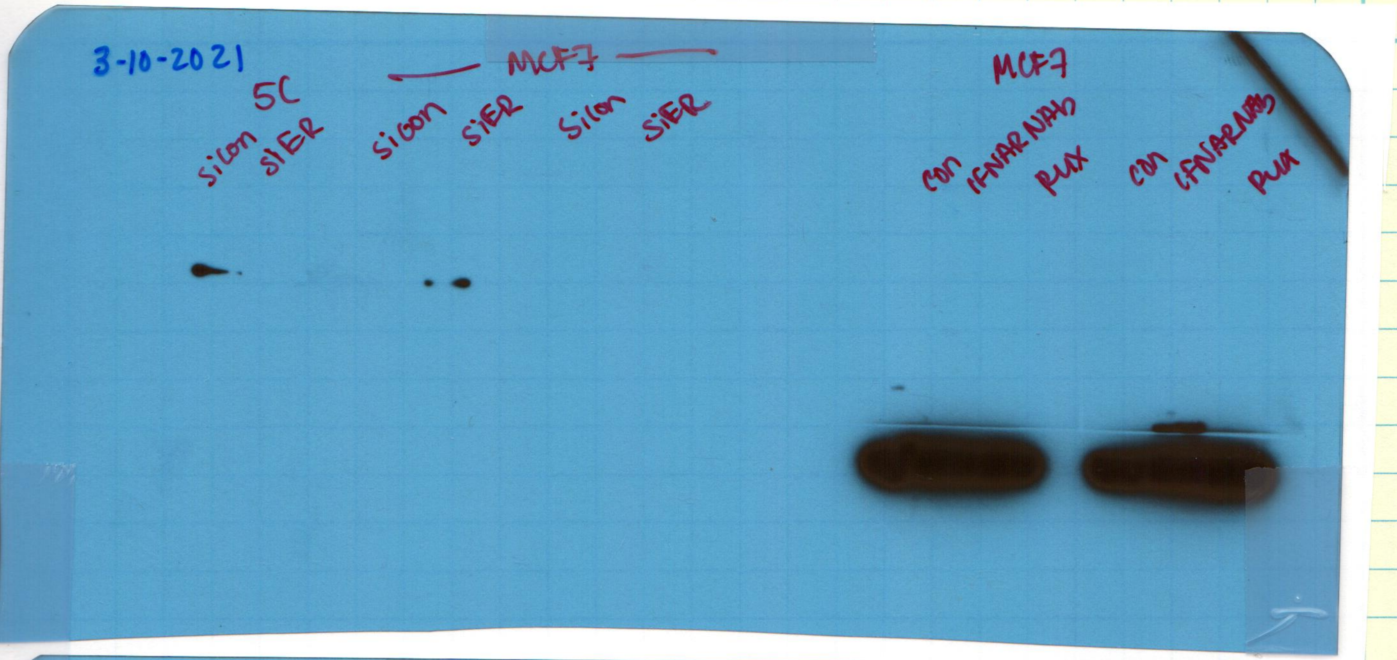
3-10-2021
MCF7
5C
Si con
Si ER
Si con
Si ER
Si con
Si ER
MCF7
con
IFNARNAb
Rux
con
IFNARNAb
Rux

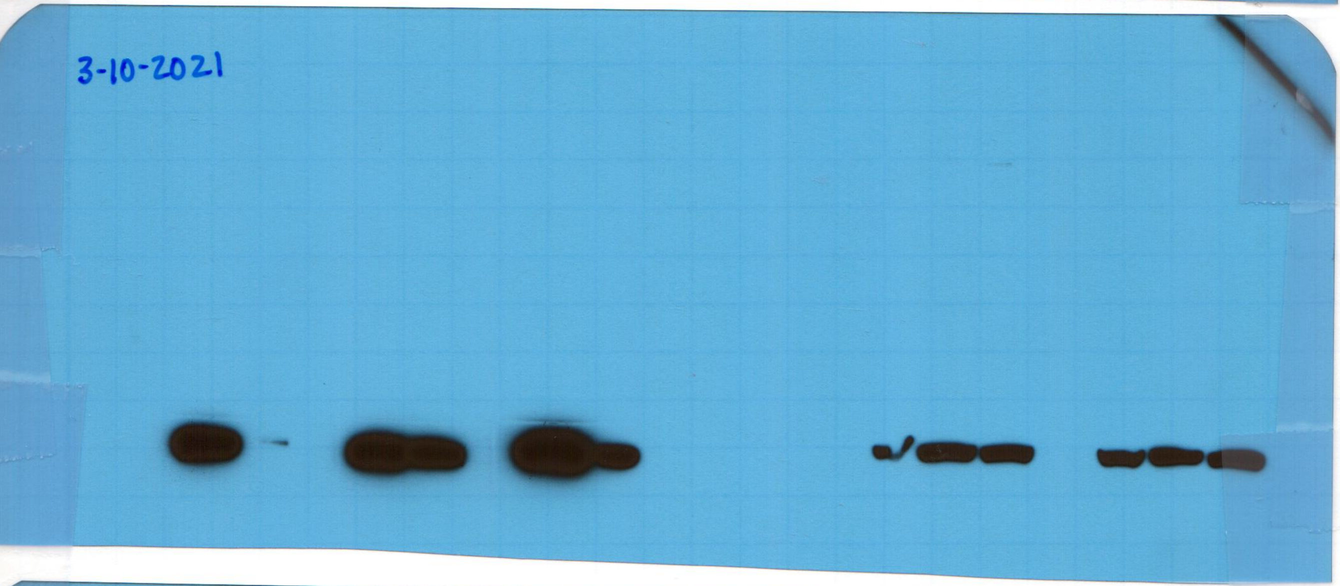
3-10-2021

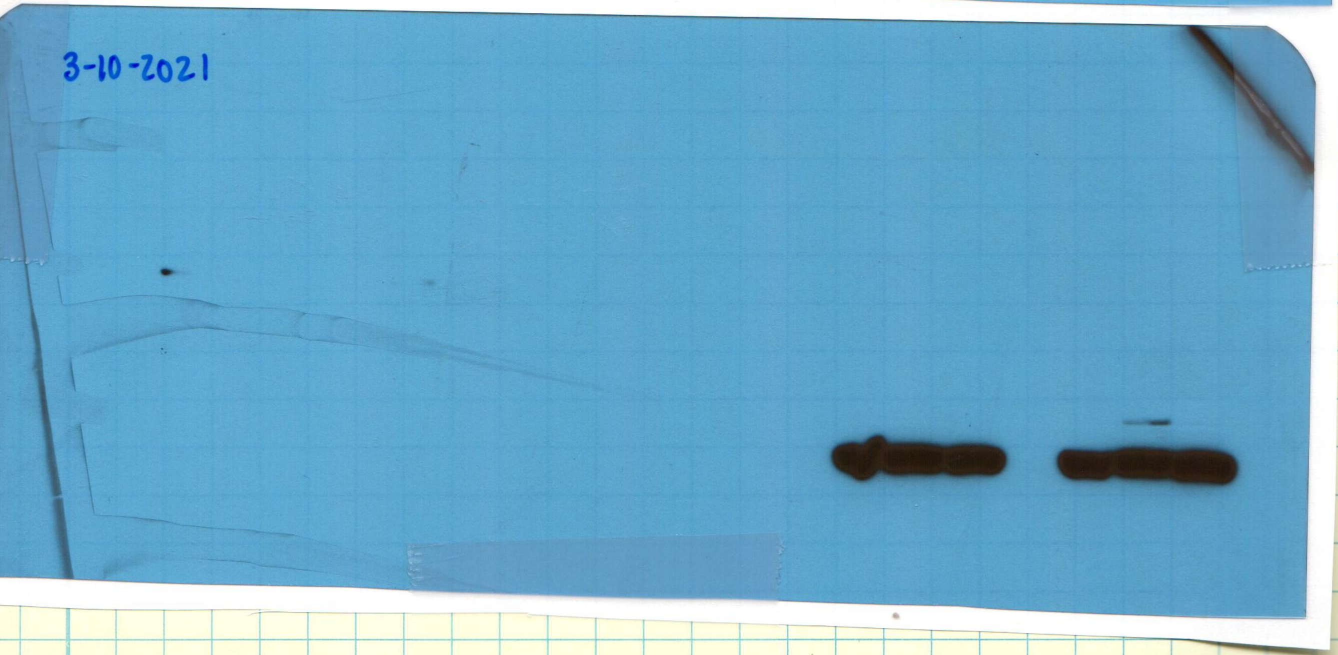
3-10-2021
