# Supplementary material for: Enhanced IFNα Signaling Promotes Ligand-Independent Activation of ERα to Promote Aromatase Inhibitor Resistance in Breast Cancer
Source: Cancers (Basel). 2021 Oct 13;13(20):5130. doi: 10.3390/cancers13205130 (PMC8534010; doi:10.3390/cancers13205130)
Supplement: Supplementary file 1 [file WB0028.pdf › cancers-1384109-supplementary/cancers-1384109-western blot/ER paper WBs/Western Scans - Lab Notebook 4/WB0028.pdf]

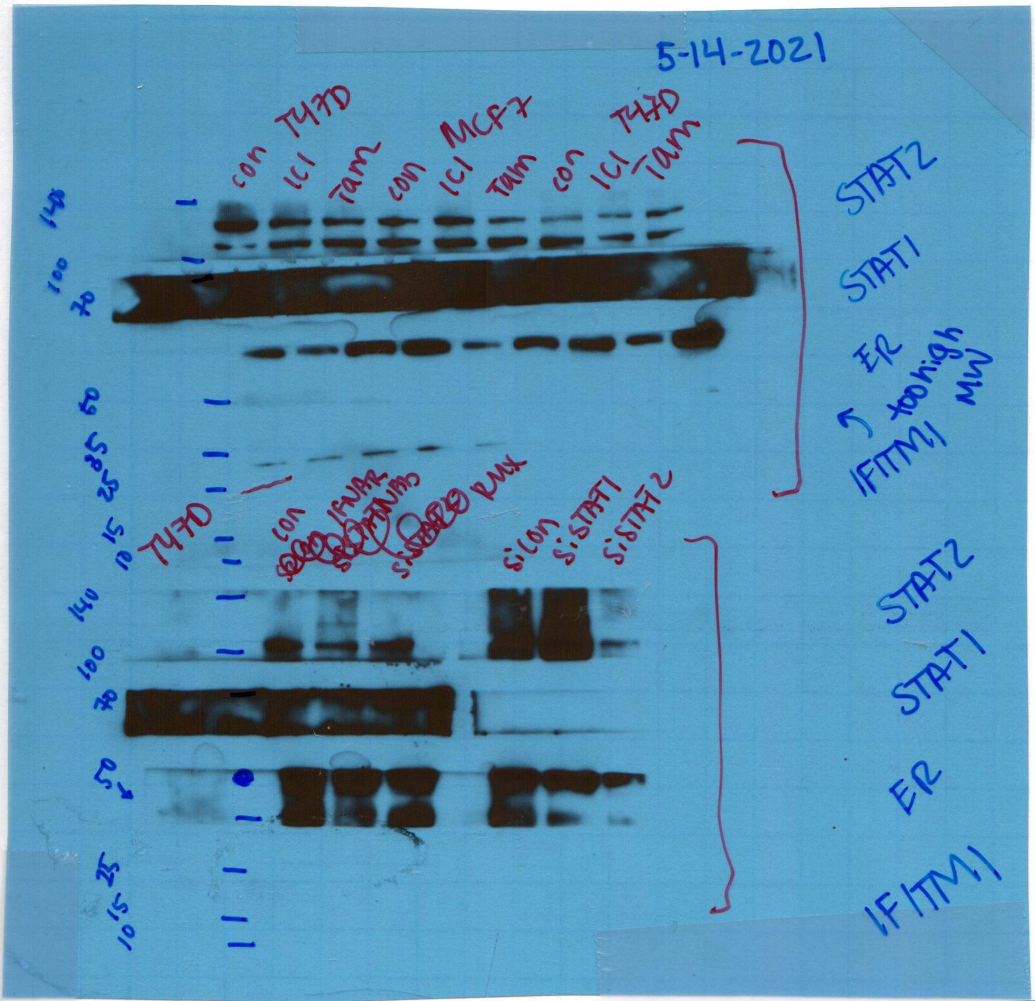
5-14-2021
T47D
MCF7
T47D
con
ICI
Tam
con
ICI
Tam
con
ICI
Tam
STAT2
STAT1
ER
IFITM1
too high MW
140
100
70
50
35
25
15
10
T47D
con
IFNAR
siSTAT1
siSTAT2
si con
si STAT1
si STAT2
RNX
STAT2
STAT1
ER
IFITM1

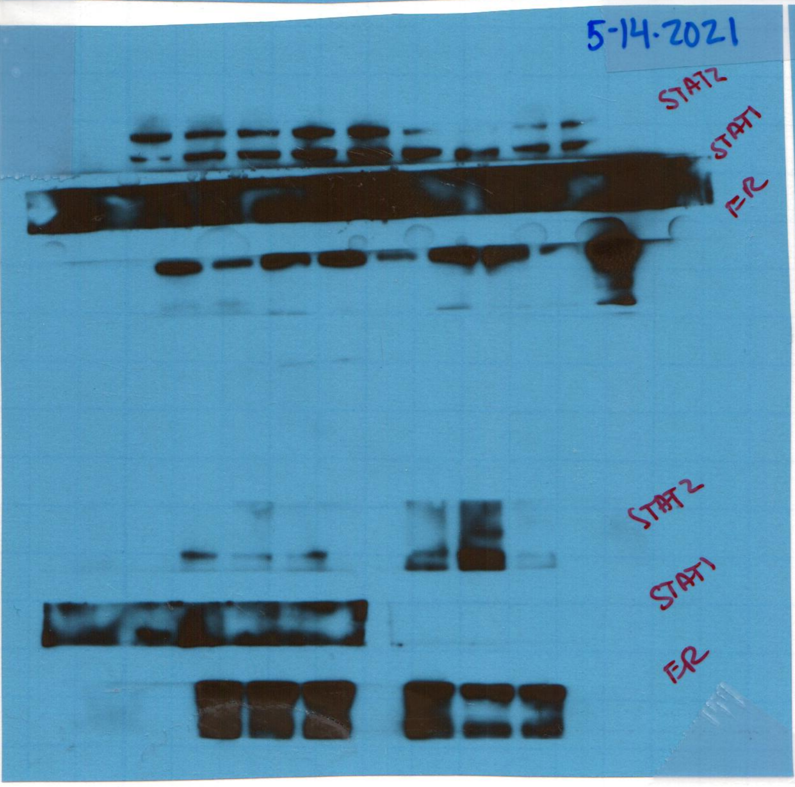
5-14-2021
STAT2
STAT1
ER
STAT2
STAT1
ER

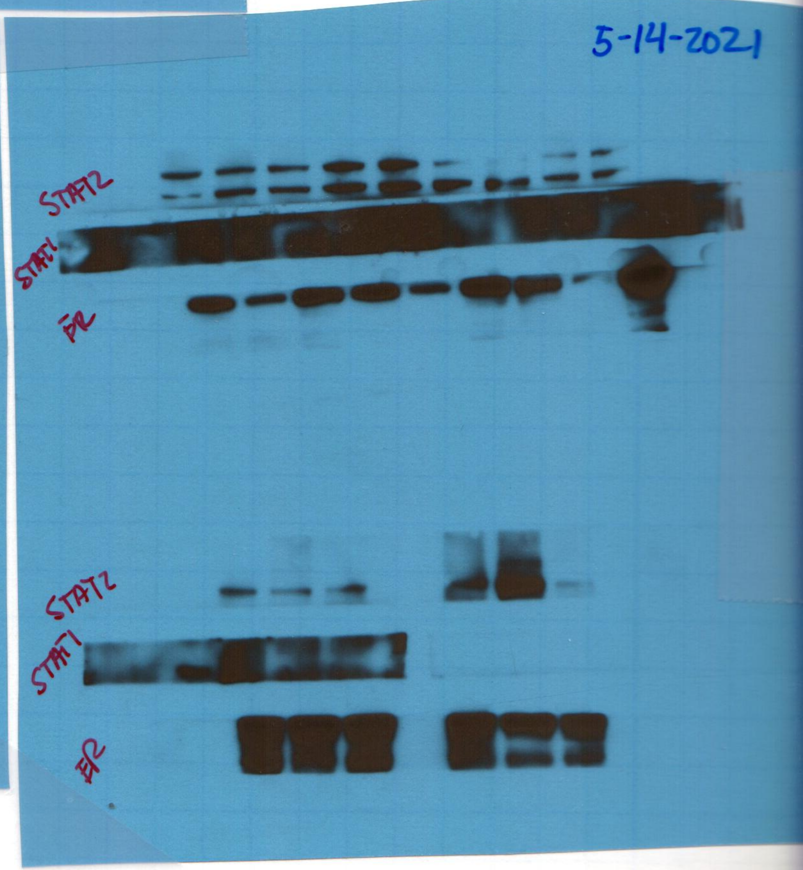
5-14-2021
STAT2
STAT1
ER
STAT2
STAT1
ER
