# Supplementary material for: Enhanced IFNα Signaling Promotes Ligand-Independent Activation of ERα to Promote Aromatase Inhibitor Resistance in Breast Cancer
Source: Cancers (Basel). 2021 Oct 13;13(20):5130. doi: 10.3390/cancers13205130 (PMC8534010; doi:10.3390/cancers13205130)

T47D Veh		T47D Rux			T47D IFNAR NAb			MCF7 Veh			MCF7 IFNAR NAb			MCF7 Rux			5C Veh			5C IFNAR NAb		
PUM1	PUM1	PUM1	PUM1	PUM1	PUM1	PUM1	PUM1	PUM1	PUM1	PUM1	PUM1	PUM1	PUM1	PUM1	PUM1	PUM1	PUM1	PUM1	PUM1	PUM1	PUM1	PUM1
IFITM1	IFITM1	IFITM1	IFITM1	IFITM1	IFITM1	IFITM1	IFITM1	IFITM1	IFITM1	IFITM1	IFITM1	IFITM1	IFITM1	IFITM1	IFITM1	IFITM1	IFITM1	IFITM1	IFITM1	IFITM1	IFITM1	IFITM1
STAT1	STAT1	STAT1	STAT1	STAT1	STAT1	STAT1	STAT1	STAT1	STAT1	STAT1	STAT1	STAT1	STAT1	STAT1	STAT1	STAT1	STAT1	STAT1	STAT1	STAT1	STAT1	STAT1
STAT2	STAT2	STAT2	STAT2	STAT2	STAT2	STAT2	STAT2	STAT2	STAT2	STAT2	STAT2	STAT2	STAT2	STAT2	STAT2	STAT2	STAT2	STAT2	STAT2	STAT2	STAT2	STAT2
IRF9	IRF9	IRF9	IRF9	IRF9	IRF9	IRF9	IRF9	IRF9	IRF9	IRF9	IRF9	IRF9	IRF9	IRF9	IRF9	IRF9	IRF9	IRF9	IRF9	IRF9	IRF9	IRF9

T47D siCon		T47D siSTAT1			T47D siSTAT2			MCF7 siCon			MCF7 siSTAT1			MCF7 siSTAT2		
PUM1	PUM1	PUM1	PUM1	PUM1	PUM1	PUM1	PUM1	PUM1	PUM1	PUM1	PUM1	PUM1	PUM1	PUM1	PUM1	PUM1
IFITM1	IFITM1	IFITM1	IFITM1	IFITM1	IFITM1	IFITM1	IFITM1	IFITM1	IFITM1	IFITM1	IFITM1	IFITM1	IFITM1	IFITM1	IFITM1	IFITM1
STAT1	STAT1	STAT1	STAT1	STAT1	STAT1	STAT1	STAT1	STAT1	STAT1	STAT1	STAT1	STAT1	STAT1	STAT1	STAT1	STAT1
STAT2	STAT2	STAT2	STAT2	STAT2	STAT2	STAT2	STAT2	STAT2	STAT2	STAT2	STAT2	STAT2	STAT2	STAT2	STAT2	STAT2
IRF9	IRF9	IRF9	IRF9	IRF9	IRF9	IRF9	IRF9	IRF9	IRF9	IRF9	IRF9	IRF9	IRF9	IRF9	IRF9	IRF9
											T47D Veh			T47D Rux		
CCND1	CCND1	CCND1	CCND1	CCND1	CCND1	CCND1	CCND1	CCND1	CCND1	CCND1	CCND1	CCND1	CCND1	CCND1	CCND1	CCND1
ps2	ps2	ps2	ps2	ps2	ps2	ps2	ps2	ps2	ps2	ps2	ps2	ps2	ps2	ps2	ps2	ps2
CTSD	CTSD	CTSD	CTSD	CTSD	CTSD	CTSD	CTSD	CTSD	CTSD	CTSD	CTSD	CTSD	CTSD	CTSD	CTSD	CTSD
FOXA1	FOXA1	FOXA1	FOXA1	FOXA1	FOXA1	FOXA1	FOXA1	FOXA1	FOXA1	FOXA1	FOXA1	FOXA1	FOXA1	FOXA1	FOXA1	FOXA1
c-myc	c-myc	c-myc	c-myc	c-myc	c-myc	c-myc	c-myc	c-myc	c-myc	c-myc	c-myc	c-myc	c-myc	c-myc	c-myc	c-myc
					T47D IFNAR NAb			T47D IFNAR NAb								
					ER	ER	ER	c-myc	c-myc	c-myc	ER	ER	ER	ER	ER	ER

T47D siER			MCF7 siER		
PUM1	PUM1	PUM1	PUM1	PUM1	PUM1
CCND1	CCND1	CCND1	CCND1	CCND1	CCND1
ps2	ps2	ps2	ps2	ps2	ps2
CTSD	CTSD	CTSD	CTSD	CTSD	CTSD
FOXA1	FOXA1	FOXA1	FOXA1	FOXA1	FOXA1
c-myc	c-myc	c-myc	c-myc	c-myc	c-myc
ER	ER	ER	ER	ER	ER
T47D IFNAR NAb			5C Rux		
CCND1	CCND1	CCND1	PUM1	PUM1	PUM1
ps2	ps2	ps2	STAT1	STAT1	STAT1
CTSD	CTSD	CTSD	STAT2	STAT2	STAT2
FOXA1	FOXA1	FOXA1	IRF9	IRF9	IRF9

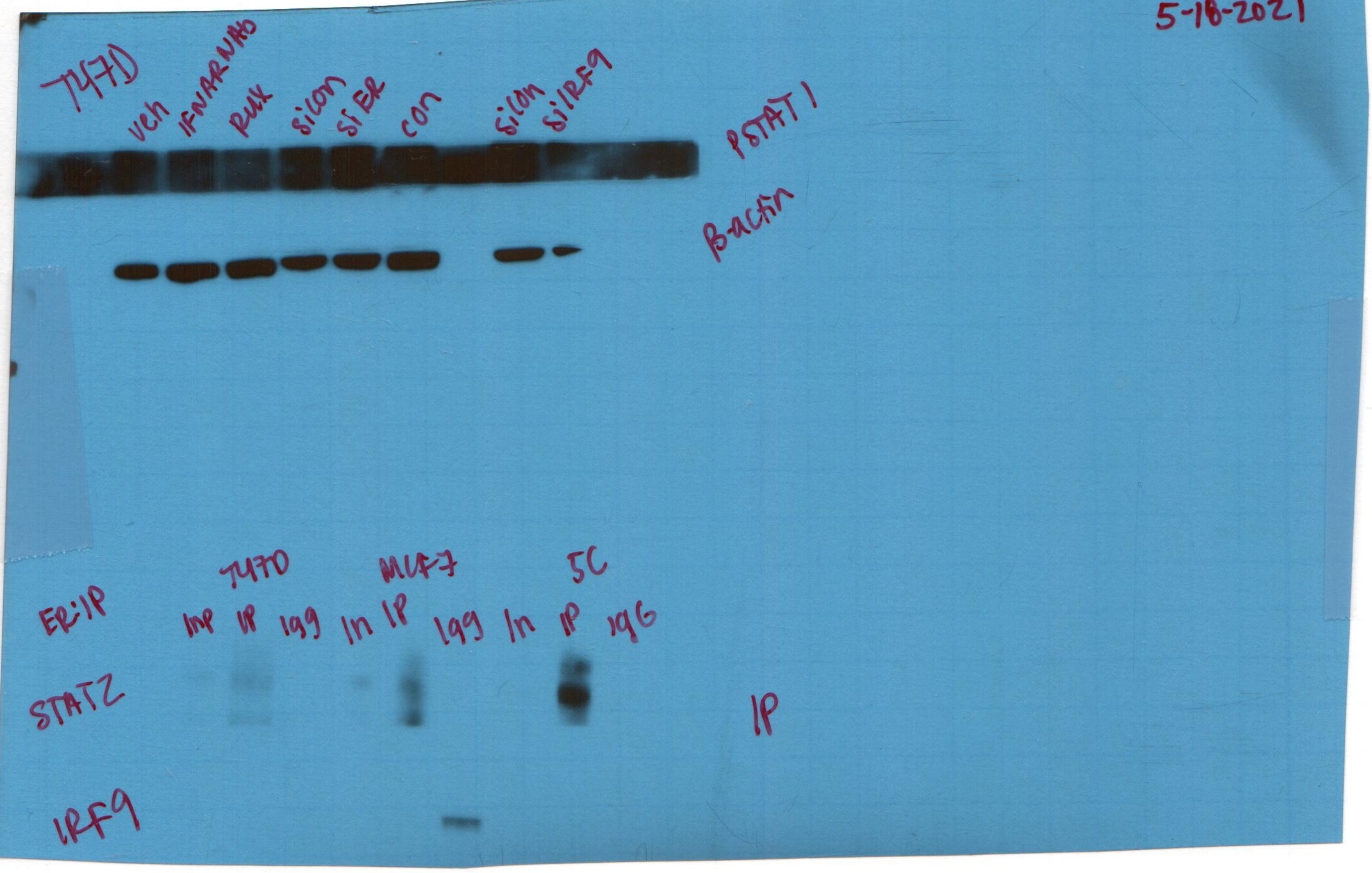

Supplement: Supplementary file 1 [file WB0030.pdf › cancers-1384109-supplementary/cancers-1384109-western blot/ER paper WBs/Western Scans - Lab Notebook 4/WB0030.pdf]
